# Multi-Phase US Spread and Habitat Switching of a Post-Columbian Invasive, *Sorghum halepense*

**DOI:** 10.1371/journal.pone.0164584

**Published:** 2016-10-18

**Authors:** U. Uzay Sezen, Jacob N. Barney, Daniel Z. Atwater, Gary A. Pederson, Jeffrey F. Pederson, J. Mike Chandler, T. Stan Cox, Sheila Cox, Peter Dotray, David Kopec, Steven E. Smith, Jill Schroeder, Steven D. Wright, Yuannian Jiao, Wenqian Kong, Valorie Goff, Susan Auckland, Lisa K. Rainville, Gary J. Pierce, Cornelia Lemke, Rosana Compton, Christine Phillips, Alexandra Kerr, Matthew Mettler, Andrew H. Paterson

**Affiliations:** 1 Plant Genome Mapping Lab., Athens, GA, United States of America; 2 Virginia Tech, Blacksburg, VA, United States of America; 3 USDA-ARS, PGRCU, Griffin, GA, United States of America; 4 USDA-ARS and University of Nebraska-Lincoln, Lincoln, NE, United States of America; 5 Texas A&M University, College Station, TX, United States of America; 6 The Land Institute, Salina, KS, United States of America; 7 Texas Tech University, Lubbock, TX, United States of America; 8 University of Arizona, Tucson, AZ, United States of America; 9 New Mexico State University, Las Cruces, NM, United States of America; 10 University of California Davis, Davis CA, United States of America; 11 State Key Laboratory of Systematic and Evolutionary Botany, Institute of Botany, China Academy of Sciences, Beijing, China; Queensland University of Technology, AUSTRALIA

## Abstract

Johnsongrass (*Sorghum halepense*) is a striking example of a post-Columbian founder event. This natural experiment within ecological time-scales provides a unique opportunity for understanding patterns of continent-wide genetic diversity following range expansion. Microsatellite markers were used for population genetic analyses including leaf-optimized Neighbor-Joining tree, pairwise FST, mismatch analysis, principle coordinate analysis, Tajima’s D, Fu’s F and Bayesian clusterings of population structure. Evidence indicates two geographically distant introductions of divergent genotypes, which spread across much of the US in <200 years. Based on geophylogeny, gene flow patterns can be inferred to have involved five phases. Centers of genetic diversity have shifted from two introduction sites separated by ~2000 miles toward the middle of the range, consistent with admixture between genotypes from the respective introductions. Genotyping provides evidence for a ‘habitat switch’ from agricultural to non-agricultural systems and may contribute to both Johnsongrass ubiquity and aggressiveness. Despite lower and more structured diversity at the invasion front, Johnsongrass continues to advance northward into cooler and drier habitats. Association genetic approaches may permit identification of alleles contributing to the habitat switch or other traits important to weed/invasive management and/or crop improvement.

## Introduction

From noble beginnings as a promising forage, Johnsongrass (*Sorghum halepense*) has become one of the most noxious agricultural weeds globally [1], costing US farmers tens of millions of dollars annually in management costs and yield losses [2]. In fact, the first US federal appropriation for weed control research targeted Johnsongrass (HB#121, 1900), yet its spread remains unchecked.

Johnsongrass (2n = 40) formed by natural hybridization between *S*. *bicolor*, (sorghum, 2n = 20) an annual native of Africa, and *S*. *propinquum* (2n = 20), a wild perennial native to wet subtropical habitats in southeast Asia that diverged from *S*. *bicolor* ~1–2 million years ago [3]. Johnsongrass is used as a forage crop in many countries, and for food (seed/flour) in some. However, it more commonly occurs as a weed, having spread from its west Asian center of diversity across much of Asia, Africa, Europe, North and South America, and Australia [1]. Its post-Columbian establishment in the US is likely typical of its spread on other continents, being introduced as a prospective forage and/or unintentionally as a contaminant of sorghum seedlots [4]. The economic impact of Johnsongrass is further increased by its frequent crossing with crop-sorghum (*S*. *bicolor*) [5], virtually precluding commercial use of transgenic sorghum [6] that has high potential to reduce crop losses to insects, diseases, weeds, and abiotic stresses improving food security particularly in low-rainfall agro-ecosystems.

Unlike most other agricultural weeds, Johnsongrass is also an aggressive invader of natural or minimally managed habitats. Johnsongrass is a state-listed noxious weed in 20 states and an invasive species in 16 states [7]. This tremendous success can be partially attributed to the ‘invasive syndrome’ of Johnsongrass including robust spreading rhizomes, shattering inflorescences, rapid growth rates, high seed dormancy, large and extensive annual seed production, impressive disturbance tolerance, potential allelochemicals, and associations with nitrogen-fixing bacterial endophytes [8–11]. Additionally, Johnsongrass has a very large climate niche, with favorable growing conditions occurring across all non-Antarctic continents [12]. In many invasive plants the size of invasive ranges is positively correlated with native range size [13], and Johnsongrass is no exception.

A compelling question is whether the spread of Johnsongrass is because it is a remarkably effective ‘general purpose genotype’ [14] or reflects rapid adaptation to new conditions that might also favor alleles of value for transfer to sorghum. Johnsongrass has now expanded into non-agricultural habitats from its initial populations that have become established in agricultural fields displaying genotypically and phenotypically distinct responses to these new environments [15]. Under replicated common garden conditions these non-agricultural accessions manifest fitness traits that are harbinger of climatic niche expansion for this most threatening invasive plant. Other examples exist of rapid evolution of exotic species following introduction to novel conditions [16, 17]; but rarely do exotic species become so successful across such broad geographies and habitats as Johnsongrass has in the US. Interestingly, as Prentis, Wilson [18] outline, the sources of the genetic variation underlying these evolutionary changes remain unknown in most invasive species.

Rapidly spreading populations contain demographic signatures due to serial founder effects. Global and large continent-wide genetic data from human and *Arabidopsis* populations have demonstrated reduced genetic diversity and increased linkage disequilibrium patterns as populations migrate away from their centers of origin. Range expansions from a limited genetic stock have significant levels of background relatedness, but have rarely been explored in invasive plants, which are modern rapid globetrotters [19].

As a foundation for investigating principles underlying the post-Colombian spread of Johnsongrass across the USA, we study Simple Sequence Repeat (SSR)-based genotypes to elucidate colonization and diversity patterns of geo-referenced populations sampling most of its continental United States range. This natural experiment provides a unique opportunity to track ‘blank slate’ colonization occurring within ecological time-scales, and provides a new perspective by broadening the timescale of invasion occurring over two centuries. A blank slate colonization may follow three trajectories: Founders may go extinct, maintain a restricted local population, or spread to all available habitats. These trajectories are shaped by the resultant vector of major co-acting evolutionary forces such as genetic drift, gene flow and natural selection. As a tetraploid with genomes inherited from two grasses respectively adapted to dry and tropical moist conditions, Johnsongrass is an outstanding experimental system in which to investigate aggressive invasion conforming to the third population trajectory.

DNA genotyping also provides a means to investigate whether patterns of genetic variation are related to the habitat or climate from which samples were collected. Findings complement and enhance historical information about the origins and spread of Johnsongrass, in particular indicating two independent invasions in very different climates separated by about 2000 miles. Finally, DNA fingerprint data provides the basis for selection and early characterization of a ‘diversity panel’ of US Johnsongrass, potentially suitable for use by association genetic approaches [20] to identify specific genes or small genomic regions contributing to spread of Johnsongrass and/or conferring adaptations of potential value for improvement of cultivated sorghum. These approaches are generalizable to investigating other weedy and/or invasive populations.

## Methods

A total of 599 genotypes were studied, with 1–32 from each of 70 different collection sites in 12 states (Figure A in S1 Fig, S2 Table). At most sites, seeds were sampled from plants at least 10 m apart to avoid clonal propagules [5]. Plants were grown in Watkinsville GA in 2012–13 to obtain tissue for DNA extraction [21]. Individuals were genotyped using 19 microsatellite markers designed to sit on arms of each of the 10 chromosomes [22] (S1 Table). PCR products were scored on 10% silver stained PAGE. Due to the tetraploidy 97 loci generated were treated as biallelic. The dissimilarity matrix and 100 bootstrapped N-J tree (S1 File) using one of the progenitor species *S*. *propinquum* as the out group were calculated by PowerMarker v3.25 [23]. N-J Tree was drawn by MEGA v4 [24]. Ordering of the leaves of the N-J tree were optimized based on the geographical context and a significance test with 1000 permutations was carried out using the software GenGIS [25]. The digital elevation model of the continental United States was downloaded from Oak Ridge National Labs’ Shuttle Radar Topography Mission (SRTM) records. A chi-squared contingency test of habitat by phase was carried out using R version 3.1.1 [26]. To test for sampling bias, two multinomial logistic regressions of habitat type against 1) annual mean temperature and annual precipitation; and 2) latitude and longitude were made. P values were estimated by Z-tests on the regression coefficients for “roadside” and “disturbed” with “agricultural” as the reference habitat. Principal Coordinate Analysis (PCoA) was done by DARWIN based on the calculated dissimilarity matrix [27]. Population genetic analysis (mismatch distribution, Tajima’s D, Fu’s F, pairwise Fst) were carried out by Arlequin 3.5 [28]. Tests for neutrality and linkage disequilibrium were performed after 10000 simulations using the program POPGENE[29]. Population genetic structure was analyzed by two programs employing Bayesian statistics STRUCTURE v2.3.3 [30] and BAPS6 [31]. A range of inferred cluster (K) values from 1 to 30 was explored using 15 replications with 80000 burn in and 800000 simulation steps. Results were plotted using STRUCTURE HARVESTER web server to visualize highest deltaK values corresponding to real population partitioning [32]. Output from STRUCTURE HARVESTER (indfile, popfile) were permuted in CLUMPP [33] and turned into a graphic using DISTRUCT [34]. Spatial structure analyses based on Voronoi tessellation were done by BAPS6 using individual and admixture models with the same range of inferred cluster values.

## Results

Neutrality test results after 10000 simulations have shown that most marker loci employed in the study are neutral. Only 7 out of 97 loci are found outside the 95% confidence intervals (Figure F in S1 Fig). Test for Linkage Disequilibrium for non-random association between pairs of loci in finite subdivided populations revealed a small total variance value (0.0847) indicating large human-assisted migration rates on a continental scale (among states) reducing LD (S3 Table, S4 Table).

Principal Coordinate Analysis (PCoA) funneled the 599 genotypes into 231 groupings (Figure A in S1 Fig, Figure B in S1 Fig). Some of these groupings consisted of individuals sampled from many locations and were not geographically distinct even after they were partitioned according to the 12 states of origin (Figure C in S1 Fig), presumably reflecting very high levels of intra-population diversity.

**Fig 1:**
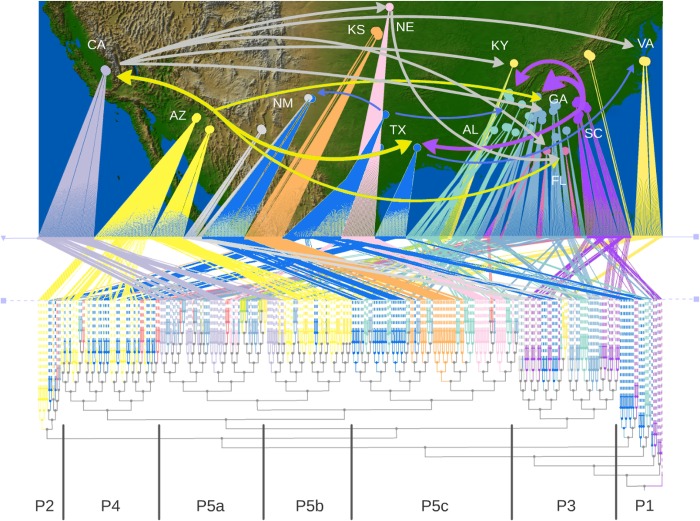
Map of sampling sites and N-J tree with an optimized leaf ordering along east–west geographical axis for *Sorghum halepense* genotypes. One of the progenitor species, *S*. *propinquum*, is used as outgroup. Colonization is outlined in 5 phases (P1-P5). Major gene flow pathways are shown using arrows in four colors (red, green, blue, gray). Initial colonization from southeastern US starting from SC (red arrows) are followed by the second introduction from AZ (S2, S4 green arrows). Gene flow from TX into NM, GA and VA (P3, blue arrows) happens concurrent with local gene flow among GA-AL-SC (P3 arrows not shown for clarity). From CA, there is a massive eastward radiation into NE, KY, FL and VA (P5a, gray arrows). While there is no detectable gene flow among KS, NE and TX (P5c) there is southbound gene flow from NE to FL (S5c, gray arrow) and KS into AL and GA (P5c, arrows not shown for clarity).

A Neighbor-Joining (N-J) tree with optimized leaf ordering visualized phylogeographic relations along the east–west axis (S1 File). The tree has its deepest branches in South Carolina (SC), providing genetic evidence for the US center of origin. The N-J tree clusters a portion of samples from southeastern states such as Georgia (GA) and Alabama (AL) with SC and extends into Texas (TX) within the first few basal nodes (Fig 1 Phase 1). Colonization from SC continued northward to at least Virginia (VA) and westward to at least New Mexico (NM) (Fig 1 Phase 3).

The N-J tree provides evidence for a second introduction, in Arizona (AZ). The founder cluster from AZ shows a very high correlation with the geographical axis. There are at least two distinct episodes of colonization from AZ into TX and then reaching to Florida (FL) and GA. (Fig 1 Phase 2, Phase 4).

The remainder of the N-J tree reflects admixture of genotypes following spread from AZ and SC, respectively. On this tree genotypes form distinct clusters for California (CA), TX, Nebraska (NE) and Kansas (KS) (Fig 1 Phases 5abc). The CA-dominated branch of the tree harbors genotypes from GA, FL, NE, Kentucky (KY) and VA on its leaves suggesting eastward gene flow from CA (Phase 5a). This pattern repeats in another clade of similar branch length (thus perhaps similar age) harboring genotypes from CA, AZ, VA, TX and GA (Phase 5b). Curious intercalation of AZ and VA genotypes may reflect long-distance gene flow between the two states after trans-continental railroad connection and eastward grain or livestock transportation. Concurrent with the rest of Phase 5, TX, KS and NE form their individual clades with no significant gene flow among themselves but occasionally flow into AL, GA and FL (Phase 5c).

Genotypes from FL, KY and VA appear at the ‘tips’ of branches indicating the most recent colonization events. As a peninsula FL has limited connectivity for gene flow. Besides, more than half of it is covered by swamps and provides fewer opportunities for population expansion with quite different agricultural practices. Indeed, FL appears to be a genetic sink rather than a source, being re-stocked at least six more times after the initial colonization (Fig 1 Phase 4, Phase 5a, Phase 5c).

Strikingly, 80% of the early colonist genotypes (Phases 1–3) were found in agricultural habitats. Genotypes from disturbed habitats were mostly found in the more recent Phase 4 and 5 clusters, and genotypes from roadside habitats were largely restricted to the Phase 5 clusters (Table 1). In these most distal clusters the agricultural habitat was underrepresented, suggesting that genetic differentiation in Johnsongrass accompanied a habitat switch away from agricultural habitats and toward disturbed and roadside habitats. Reintroductions of Johnsongrass to the Southeastern states reflect these changes in habitat; later colonizers were found primarily in disturbed and roadside habitats, while the more ancestral genotypes were found in agricultural habitats.

**Table 1:** Contingency table showing observed frequency of *Sorghum halepense* accessions belonging to each genetic cluster, found in each habitat type (Χ^2^ = 121.79; df = 12; P < 0.0001).

	Agricultural	Disturbed	Roadside
Phase 1	22	1	3
Phase 2	18	0	0
Phase 3	14	4	6
Phase 4	47	17	1
Phase 5a	24	10	19
Phase 5b	13	21	32
Phase 5c	0	12	26

These results suggest that initial expansion of Johnsongrass occurred primarily in agricultural habitats, with a subsequent transition of a cluster of closely related genotypes to non-agricultural habitat throughout the entire US range. This pattern was not influenced by sampling bias across geographic clines. Multinomial logistic regressions show that the three habitat types were equally represented along latitudinal, longitudinal, and rainfall clines (*P* > 0.213; multinomial logistic regression, *Z*-test), although agricultural habitat was less common in areas with high mean annual temperatures (*P* < 0.031). However, such sampling bias would have countered the trends we observed, as it would have caused us to be more, and not less, likely to sample non-agricultural habitat in the warmer ancestral populations.

Mismatch analysis [35], shows populations from all 12 states to exhibit demographic and spatial expansion with unimodal distributions (Figure C in S1 Fig, Figure D in S1 Fig). KY and FL showed a slowdown in expansion, with Harpending's raggedness index two orders of magnitudes larger than in GA and TX (Table 2). This slowdown may be explained in FL by geography and in KY may be an artifact of low sample number. Statistical tests of selective neutrality also suggested population expansion. Tajima’s D values were all positive indicating a past bottleneck but p-values were not significant (Table 1). Fu’s Fs, a more sensitive measure of population fluctuations, showed large negative values supported with significant p-values indicating a history of population expansion.

**Table 2:** Summary of sample sizes, number of polymorphic sites, Harpending’s raggedness index, Theta pi measure, Tajima’s D, Fu’s Fs values of *Sorghum halepense* populations according to the sampling locations (by state). The two progenitor genotypes *S*. *propinquum*, *S*. *bicolor* and the laboratory standard *S*. *halepense* are grouped as PBH.

	PBH	VA	KY	SC	GA	FL	AL	TX	NE	KS	NM	AZ	CA	mean	s.d.
**Sample Size**	3	29	12	42	85	22	54	127	32	36	17	86	54	46.08	35.15
**polymorphic site #**	47	89	73	85	96	84	88	95	83	84	75	87	83	82.2	12.41
**Raggedness**	na	0.01	0.017	0	0.0009	0.0079	0.0029	0.0005	0.0055	0.0043	0.007	0.0014	0.001	na	na
**Theta pi**	31.3	32.9	30.12	30	35.99	31.48	31.33	36.86	30.69	29.36	30	30.58	28.81	31.49	2.42
**Tajima's D**	0	1.72	1.14	1.87	2.94	1.47	2.18	3.54	1.84	1.66	1.48	2.54	2.03	1.88	0.86
**p-value**	0.32	0.93	0.88	0.95	0.98	0.92	0.96	0.99	0.95	0.93	0.93	0.97	0.95	0.9	0.17
**Fu's Fs**	2.32	-9.8	-1.8	-20.6	-24.08	-6.07	-24.15	-23.93	-12.41	-15.9	-3.85	-24.02	-24.13	-14.49	9.79
**p-values**	0.55	0	0.12	0	0.0003	0.014	0.0001	0.0034	0.0009	0.0004	0.038	0.0004	0	0.056	0.151

**Fig 2:**
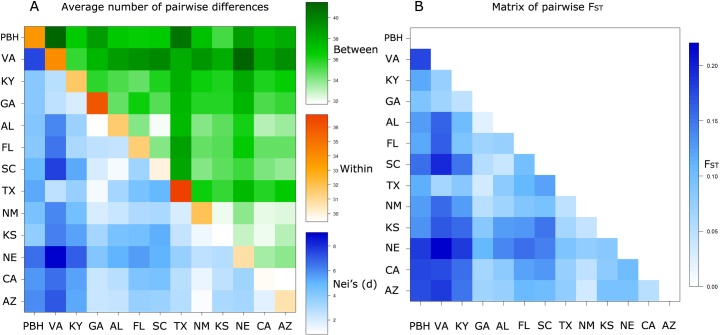
**(a)** Pairwise comparisons of Nei’s distances (net and raw distances) among (lower/upper diagonal) and within (along diagonal) *Sorghum halepense* populations. **(b)** Pairwise comparisons of Fst among populations. Populations diverge as they get farther away from GA and TX. The two progenitor genotypes *S*. *propinquum*, *S*. *bicolor* and the laboratory standard *S*. *halepense* are grouped as PBH.

Pairwise comparisons of allelic diversity within populations identify GA and TX as the states now harboring the greatest Johnsongrass diversity. SC, KS and CA harbored the least within-population diversity (Fig 2A). A pairwise Fst matrix detected patches of low population structure centered around GA and TX, with increases toward the edges of the continent-wide colonization. The most distinct state is VA, showing the highest Fst values in almost every pairwise comparison, the exceptions being with GA and TX. The pattern is mirrored at the other end of the continent where NE, CA and AZ show high Fst values when compared with VA and KY. High Fst’s drop when VA and KY are compared with GA and TX. On the other hand, TX/GA, AZ/NM and SC/AL have the lowest Fst values. These findings indicate TX/GA to represent the current center of diversity, but SC/AL and AZ/NM to have been centers of origins whose diversity are relatively lower (Fig 2B).

**Fig 3:**
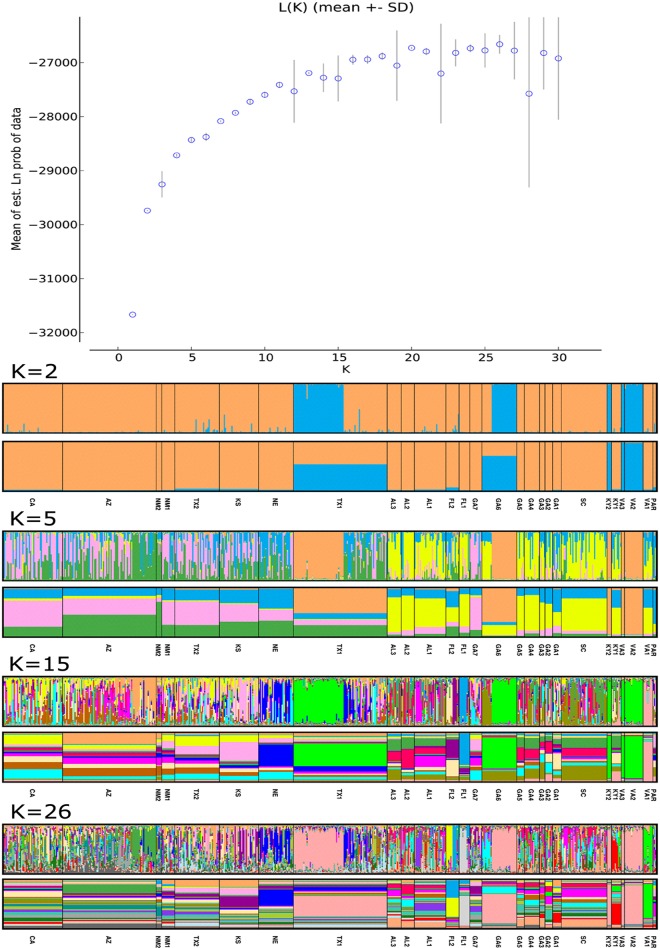
Saturation plot of *Sorghum halepense* genotypes after STRUCTURE runs based on Evanno Method (top). K values reach an asymptote between 15 and 26. DISTRUCT bar graph visualization of results after permuted by CLUMPP at four different K cluster assumptions (K = 2, 5, 15, 26). Clustering based on population averages (lower bars) and individual genotypes (upper bars). The two parental species *Sorghum bicolor* and *Sorghum propinquum* are labeled as PAR.

**Fig 4:**
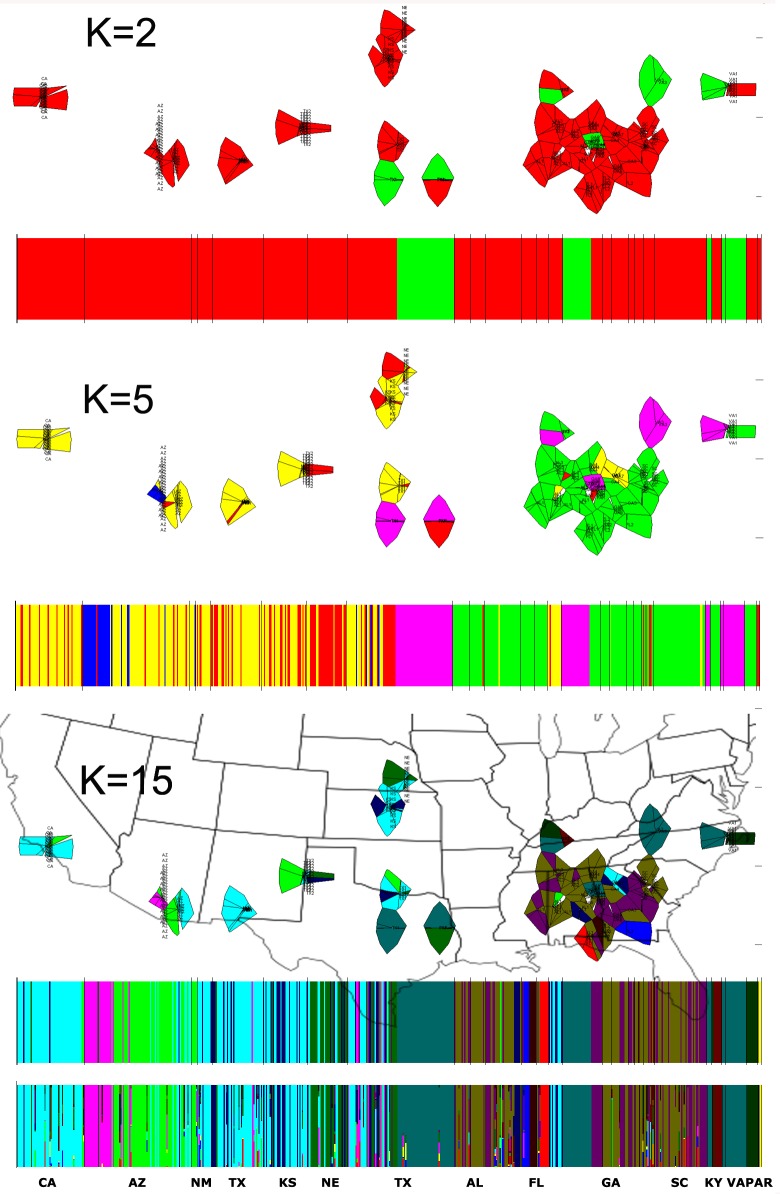
BAPS6 geographical clustering using Voronoi tesellations and bar representations of genotypes at three K values based on population averages (K = 2, 5, 15). State boundaries overlaid for K = 15 for visual guidance. Clustering based on individual genotypes (lower bars) is included for K = 15. The two parental species *Sorghum bicolor* and *Sorghum propinquum* are labeled as PAR.

Two Bayesian analysis programs, STRUCTURE [30] and BAPS6 [31], were used to investigate population structure and probe composition of ancestral subpopulation clusters. These programs assume that there are K clusters and the true value of K is estimated by a continuous sweep of replicated runs based on multilocus genotype data. Using the Evanno method, STRUCTURE detected between 15 and 26 population clusters [32] (Fig 3; Fig 4). Population cluster numbers remained bound within those values at very high K (K = 80, Figure E in S1 Fig). Further permutation of results using CLUMPP grouped most ancestral population blocks around southeastern states except one block from Arizona which represents a second separate introduction confirming the pattern painted by the N-J tree (Fig 1) [33]. Spatial analysis using BAPS6 also detected this ancestral population block starting from K = 5, predicting a maximum of 15 distinct populations at present (Fig 4).

## Discussion

Historical demography of a population is imprinted on present day genetic structure, and genetic trees can be quite informative to understand the past. Researchers have successfully reconstructed pre-Columbian dispersal of sweet potato (*Ipomea batatas*) into Oceania [36]. Similarly, prehistoric origins of cultivation and human-assisted dispersal of coconut palm (*Cocos nucifera*) around the World has been reconstructed [37]. Here in this study, a N-J tree of Johnsongrass genotypes with optimized leaf ordering demonstrates a correlation with geography. The most basal branches in this tree originate from SC and spread into GA, AL and TX (Fig 1, Phase 1). According to a historical account [38], John Means of SC introduced Johnsongrass in contaminated hemp seed from Egypt shortly after the Revolutionary War. His daughter married the ‎eponymous Colonel Johnson and moved to Alabama, where Col. Johnson wrote an 1847 letter resulting in the common name Johnsongrass [39]. The Arizona Gazette reports farmers complaints about Johnsongrass as early as 1890 [38] in concordance with a second introduction suggested by the N-J tree (Fig 1, Phase 2).

Johnsongrass is a striking example of continent-wide colonization following a post-Columbian founder event. This natural experiment in a largely self-pollinating perennial grass provides an opportunity to study population genetic dynamics operating at an ecological time-scale. Population genetic theory predicts that founders have limited genetic diversity with the potential for allele frequencies to drift erratically compared to their large panmictic source population [40]. Theory also predicts that initial sites of colonization should harbor the largest genetic diversity, with less diversity and more genetic structure at the margins. These predictions are based on the assumption of a single source for colonization. Here, multiple introductions from two ends of a geographical range appear to generate richer diversity in the middle ground than at the initial sites of colonization. At the same time, margins can still have high genetic structure in accordance with classic predictions. A review of 80 exotic animals, plants, and fungi found that multiple introductions and long post-introduction residence time are associated with increased gene flow and increased diversity, which is often lost in exotic species [41].

Pairwise comparisons of Fst and Nei’s distances of AZ and SC show reduced within-population and increased among-population variation (Fig 2). AZ poses exceptionally dry conditions that may impose selection for drought responsive traits, the *S*. *bicolor* progenitor being noted for drought tolerance although *S*. *propinquum* is native to wet habitats. Reduced within-population genetic diversity observed in inferred centers of introduction could be attributed to both founder effects and possible selection for characteristics optimal for conditions as different as dry AZ and humid SC. The observed increase in genetic diversity in TX and GA might be a result of admixture between divergent genotypes from the two different introductions.

Although useful to describe the present state of populations [42] an ordination method (PCoA) was insufficient to resolve a founding Johnsongrass population, most probably because multiple points of introduction create a diffusion pattern different from classic single source population expansions. Among 231 groupings (Figure A in S1 Fig), many were geographically indistinct, perhaps reflecting the remarkably high level of DNA polymorphism in Johnsongrass. Allelic richness of 182 genetically-mapped RFLP loci averaged 6.13 alleles per locus in 13 Johnsongrass and 5 *S*. *almum* [backcross derivatives of natural crosses between Johnsongrass and *S*. *bicolor*: [43]] accessions from the USA (8), Australia (2), Algeria, Argentina, Chile, India, Kazakhstan, New Zealand, South Africa, and the former USSR; versus 3.39 for a worldwide sample of 55 landrace and wild sorghum accessions; and 1.9 for 16 F1 hybrid sorghums from eight US commercial breeding programs [5]. However, PCoA provided a convenient first pass to choose a reduced sample of genotypes for further characterization as a ‘diversity panel’ of US *S*. *halepense*, potentially suitable for use by forward genetic approaches to identify alleles responsible for specific adaptations [20] (Figure A in S1 Fig, Figure B in S1 Fig).

The gradient observed in pairwise Fst comparisons of CA with other states is one of the most visually distinct features on the matrix, where Fst reaches its highest value in VA (Fig 2B) and shows a steady decline to a low at GA. In both pairwise comparisons VA stands out as the most divergent population, demonstrating peak values in comparisons with NE, SC and AZ. Range expansion leads to a reduction in genetic diversity with increasing distance from the origins of the expansion and we observe lowest within population genetic diversity at the edges of the sampled area such as Kansas and CA (Fig 2A).

Expansion of Johnsongrass throughout the Southeastern US corresponded to a striking habitat switch from agricultural (e.g., corn fields) to non-agricultural (i.e., roadside or disturbed systems) habitats in a multi-phase colonization process [15]. Ancestral genotypes of Johnsongrass are now found almost exclusively in agricultural habitats, and more derived genotypes (Phase 4 and 5) are found almost exclusively in non-agricultural habitats (Table 1). This suggests that initial expansion of Johnsongrass was in primarily agricultural habitats followed by a secondary phase of invasion that occurred when a family of non-agricultural specialists evolved and rapidly colonized non-agricultural habitats throughout the entire Southern US. This second phase of invasion may reflect a period of rapid railroad and highway expansion that occurred in the late-nineteenth and early-twentieth centuries. Concomitantly with transportation development was a dramatic shift in agricultural weed management that moved away from mechanical control (e.g., cultivation) to chemical control, which may have facilitated this putative ecotypic shift. If this habitat switch represents a niche expansion for Johnsongrass, it may help to explain the particular invasiveness demonstrated by Johnsongrass, which is nearly ubiquitous in the farms, fields, prairies, and ditches of the Southern U.S [15].

The finding that DNA genotyping is diagnostic of a habitat switch suggests that specific alleles or allele combinations may be responsible for the switch, and that their identification might lead to novel means of mitigating the spread of Johnsongrass, at least to non-agricultural habitats. The limited sampling of SSRs that was sufficient for this study is inadequate to scan the entire genome for associations with important Johnsongrass traits, but reduced-representation resequencing of a diverse subset of the accessions studied here may provide the required resolution. For example, extensive spread and admixture may have seeded diverse geographies with identical-by-descent alleles for herbicide resistance, that appear to comprise independent evolutions when subsequent conditions such as increased herbicide usage favor their increase in frequency [44]. Such a genome-wide association approach may make it possible to dissect the genetic basis of the habitat switch, however more specific traits that differentiate the respective habitat types will first need to be identified and quantified in this tetraploid weed [15].

Despite lower and more structured diversity at the invasion front, Johnsongrass continues to advance northward [45], with predictions of the damage niche (conditions in which a plant becomes an agricultural pest) to also move northward with climate change [46]. Other invaders evolve more rapidly on the leading edge than those in the interior of their distribution [47]; and Johnsongrass is poised to continue this advance with more habitats available to colonize.

The richness of alleles in Johnsongrass and its spread to environments beyond the reach of its progenitors could offer novel and valuable variants for improvement of sorghum for resistance to drought, cold, disease and other stresses. Our improved understanding of population structure, together with an incipient ‘diversity panel’ of US *S*. *halepense*, provides for association genetic approaches to identify alleles responsible for specific traits of importance to weed/invasive management, and/or to crop improvement [20].

## Supporting Information

S1 Fig **Figure A.** Map of sampling sites and Principle Coordinate Analysis (PCoA) distribution of samples. **Figure B.** Principle Coordinate Analysis and locations of five groups at two extremes shown with number of genotypes in each group. **Figure C.** Mismatch distribution profiles (demographic expansion) from 12. **Figure D.** Mismatch distribution profiles (spatial expansion) from 12 states. **Figure E.** STRUCTURE analysis of genotypes at K = 80. **Figure F.** Neutrality test results for genetic markers employed in the study after 10000 simulations.
(DOCX)

S1 File Newick format file of N-J tree with bootrap values. (NWK)

S1 Table Genetic marker information. (DOCX)

S2 Table GPS locations of samples. (CSV)

S3 Table Overall averages of variance components in test for Linkage Disequilibrium for non-random association between pairs of loci in finite subdivided populations. (DOCX)

S4 Table Variances for every pairwise comparison of loci in Linkage Disequilibrium test for non-random association between pairs of loci in finite subdivided populations. (DOCX)
