# Supplementary material for: Multi-Phase US Spread and Habitat Switching of a Post-Columbian Invasive, Sorghum halepense
Source: PLoS One. 2016 Oct 18;11(10):e0164584. doi: 10.1371/journal.pone.0164584 (PMC5068735; doi:10.1371/journal.pone.0164584)
Supplement: S1 Fig — Figure A. Map of sampling sites and Principle Coordinate Analysis (PCoA) distribution of samples. Figure B. Principle Coordinate Analysis and locations of five groups at two extremes shown with number of genotypes in each group. Figure C. Mismatch distribution profiles (demographic expansion) from 12. Figure D. Mismatch distribution profiles (spatial expansion) from 12 states. Figure E. STRUCTURE analysis of genotypes at K = 80. Figure F. Neutrality test results for genetic markers employed in the study after 10000 simulations. (DOCX) [file pone.0164584.s001.docx]

**Supporting Information**

**Sezen et al. 2016**

**Multi-phase US spread and habitat switching of a post-Columbian invasive,** ***Sorghum halepense.***

**Figure Legends**

**Fig. S1** Map of sampling sites (a) and PCoA distribution of samples grouped into 231 points (b). Same data grouped into states (c).

**Fig. S2** Principle Coordinate Analysis and locations of five groups at two extremes shown with number of genotypes in each group.

**Fig. S3** Mismatch distribution profiles (demographic expansion) from 12 states and parental genotypes (Sp-Sb) with *Sorghum halepense* laboratory reference genotype Gypsum9E (Sh).

**Fig. S4** Mismatch distribution profiles (spatial expansion) from 12 states and parental genotypes (Sp-Sb) with laboratory reference genotype Gypsum9E (Sh).

**Fig. S5** STRUCTURE analysis of genotypes at K=80.

**Fig, S6** Neutrality test results after 10000 simulations. Only 7 out of 97 loci are found outside the 95% confidence intervals.

**
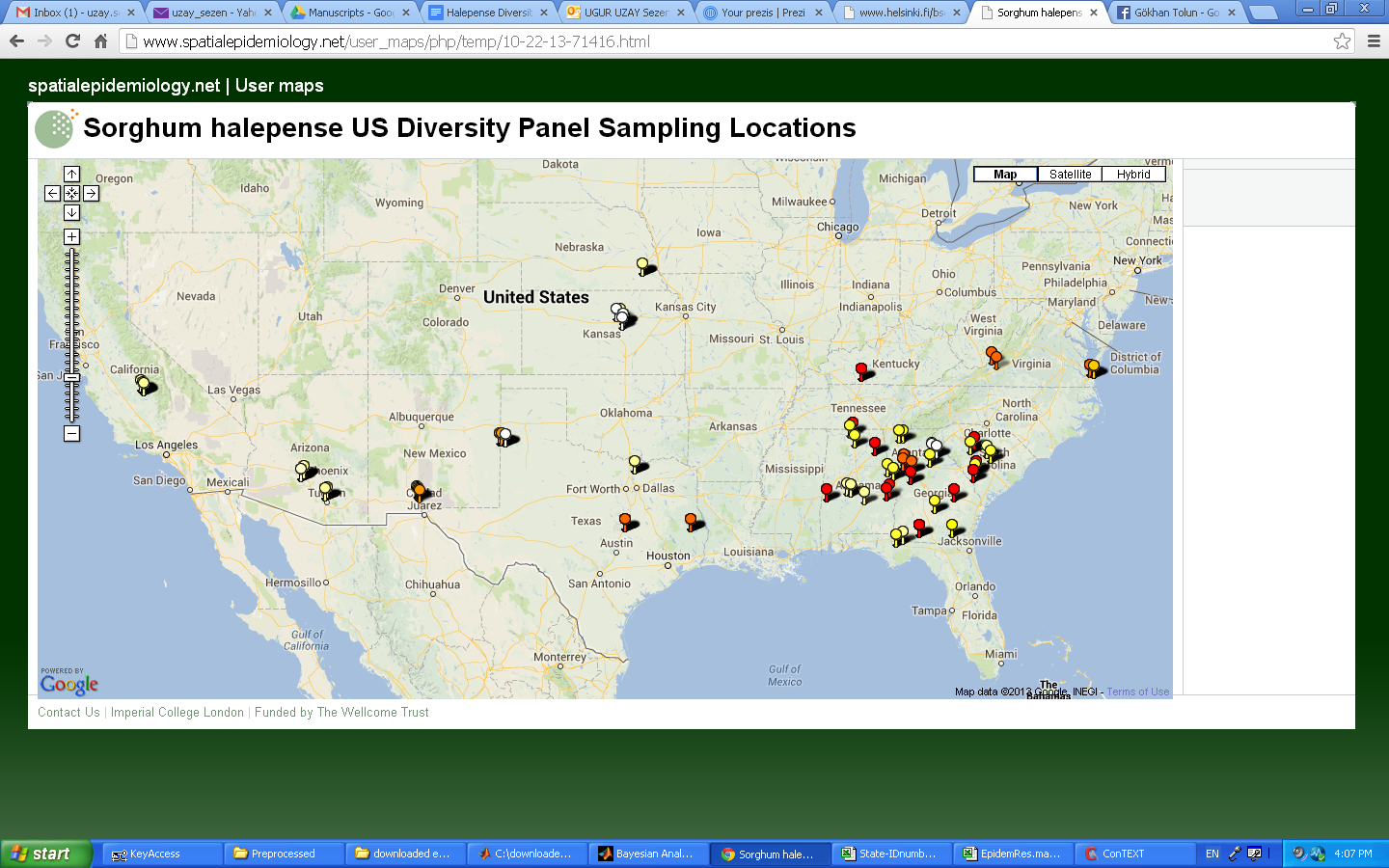
**

**(a)**


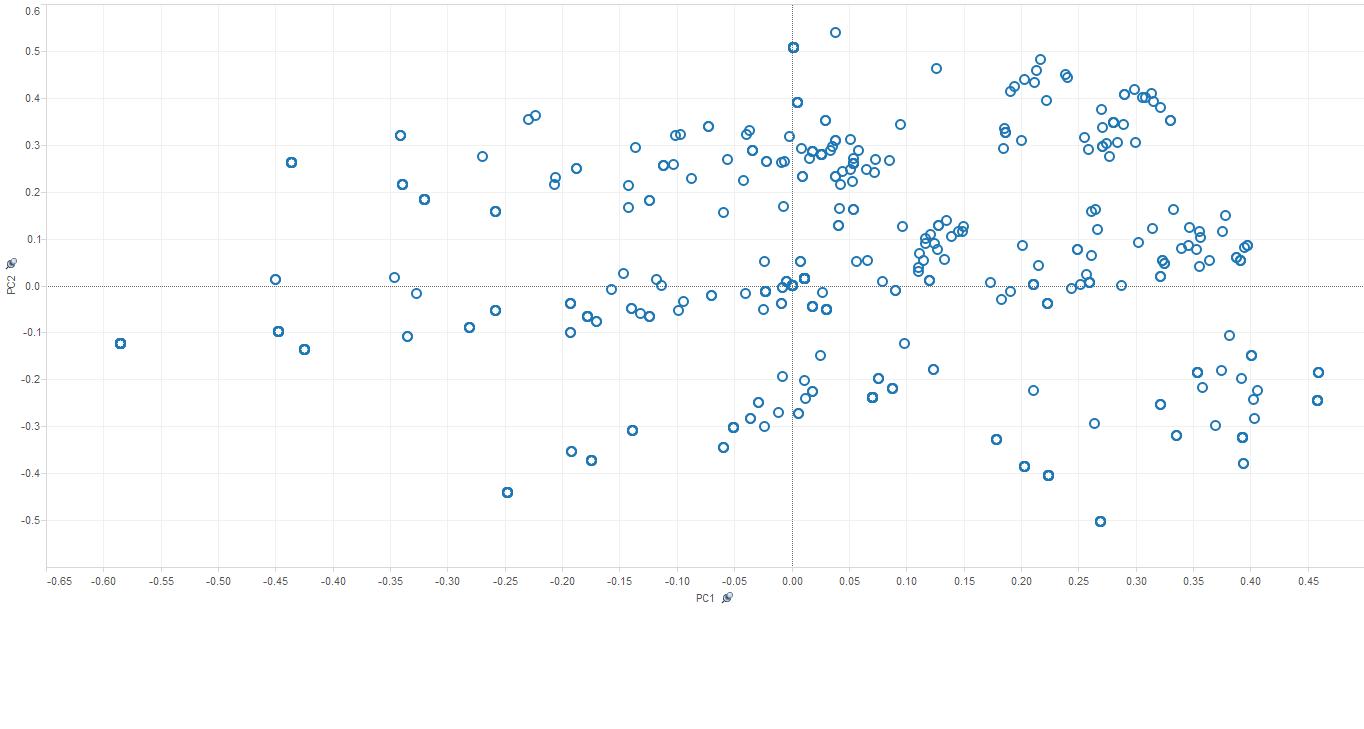
**(b)**

**
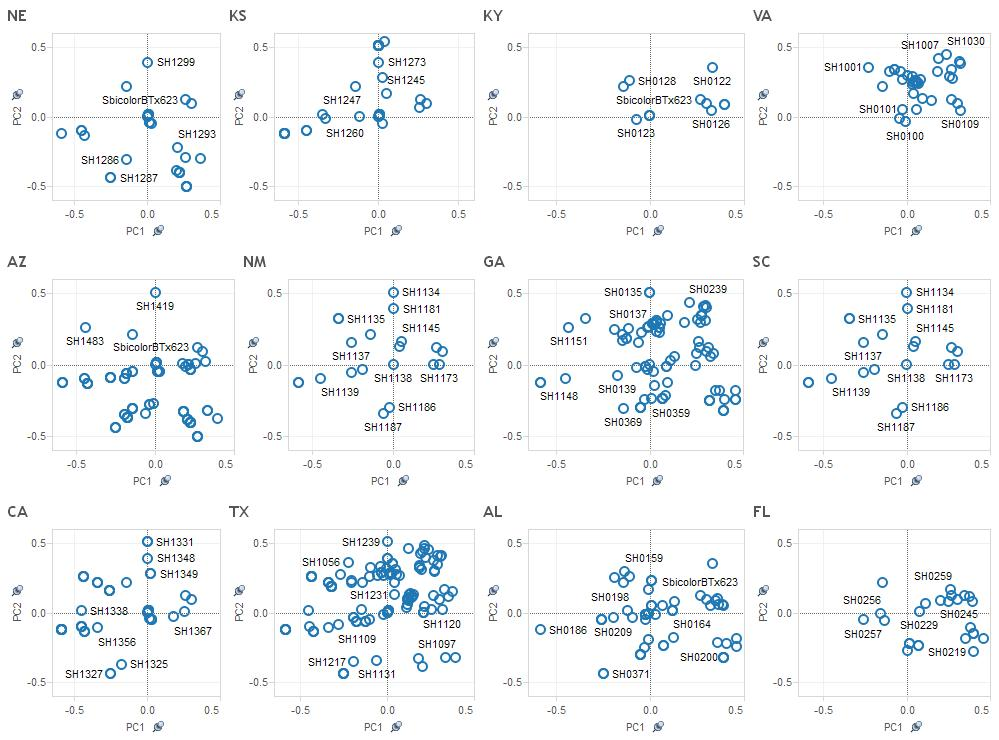
**

**(c)**

**Fig. S1** Map of sampling sites (a) and PCoA distribution of samples grouped into 231 points (b). Same data grouped into states (c).


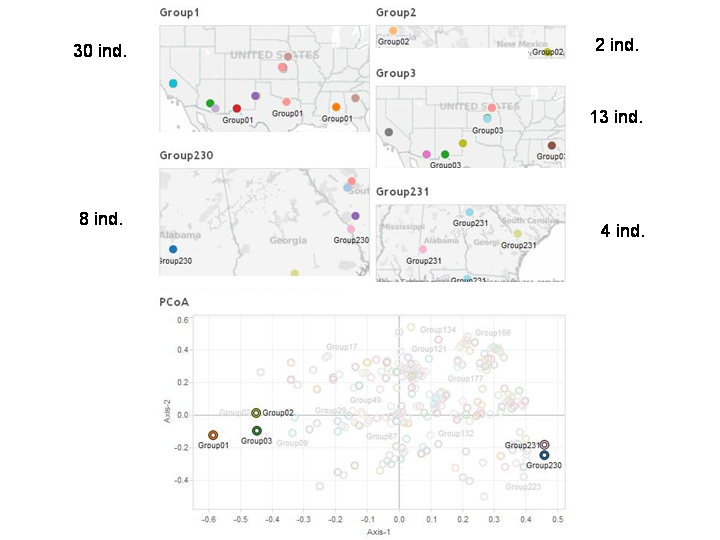


**Figure S2** Principle Coordinate Analysis (PCoA) identified 231 groupings. Locations of genotypes of five groups at two extremes are shown with number of individual genotypes in each group.


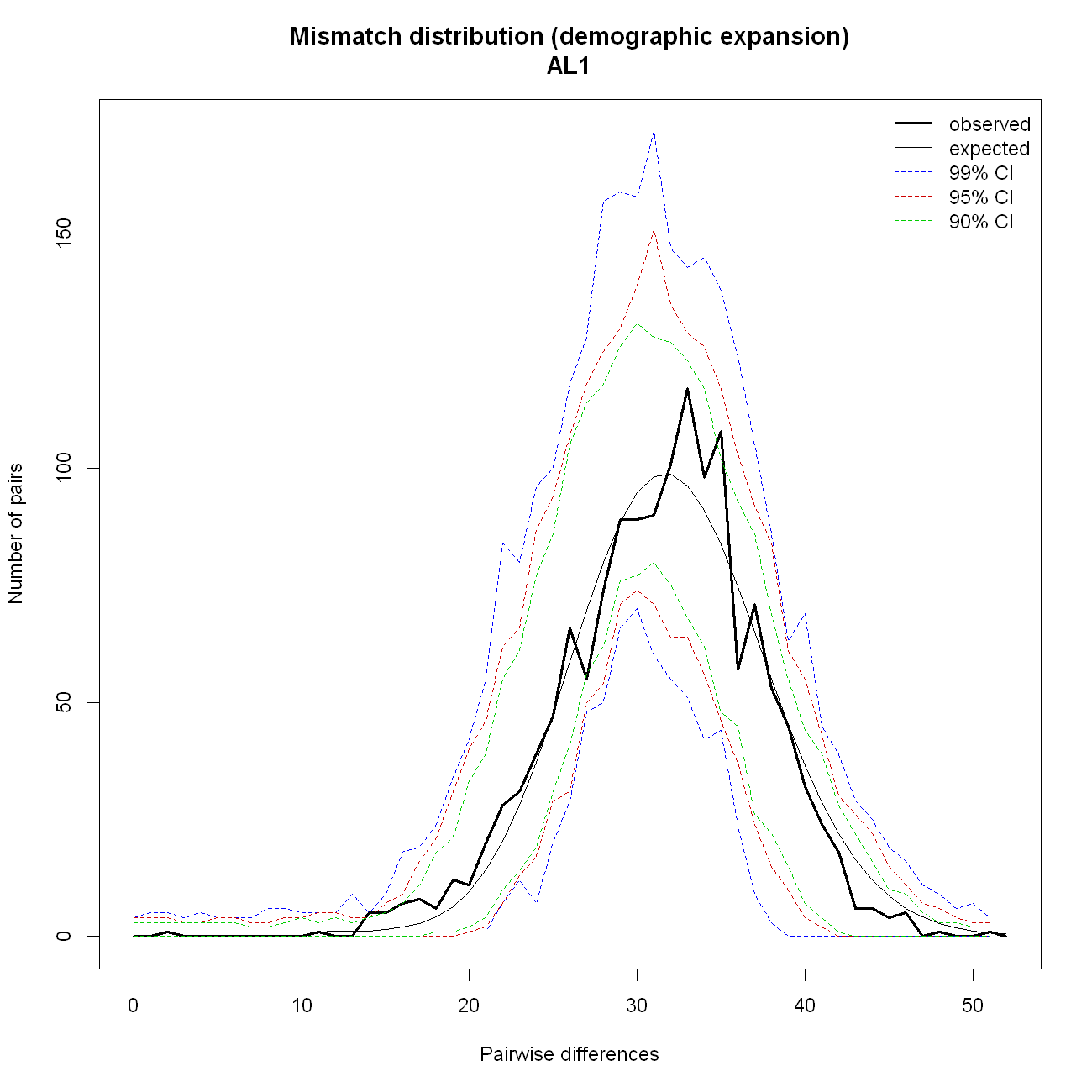

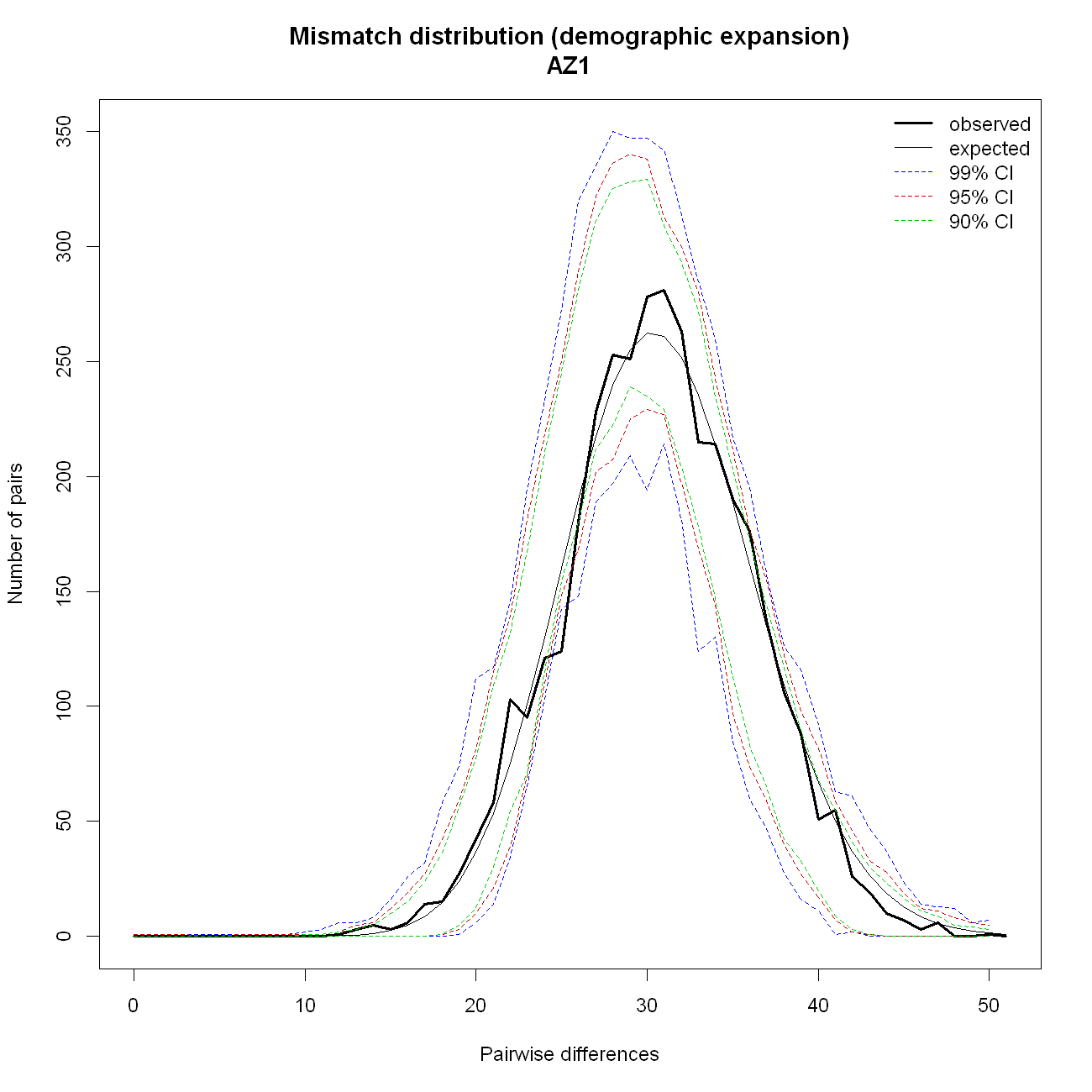


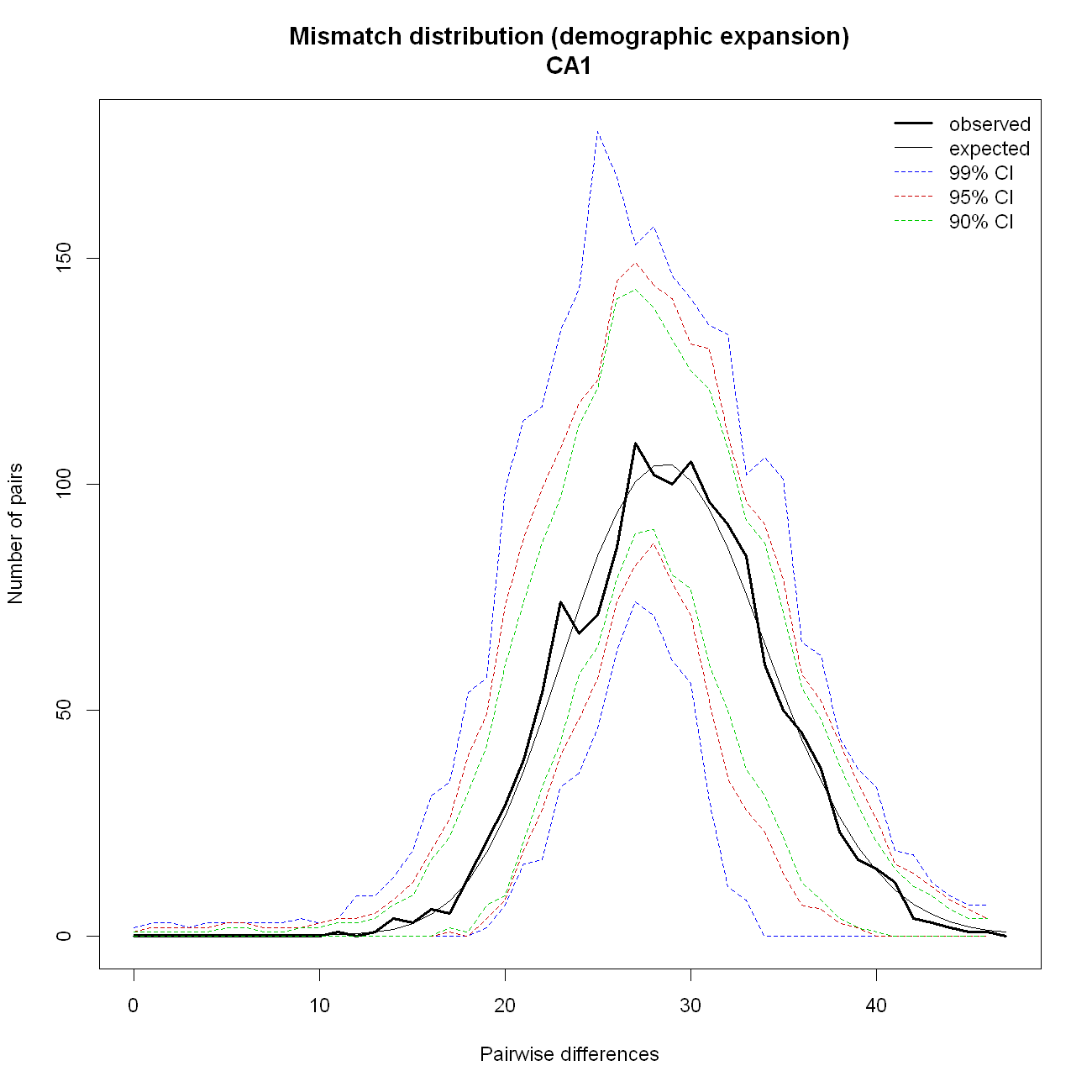

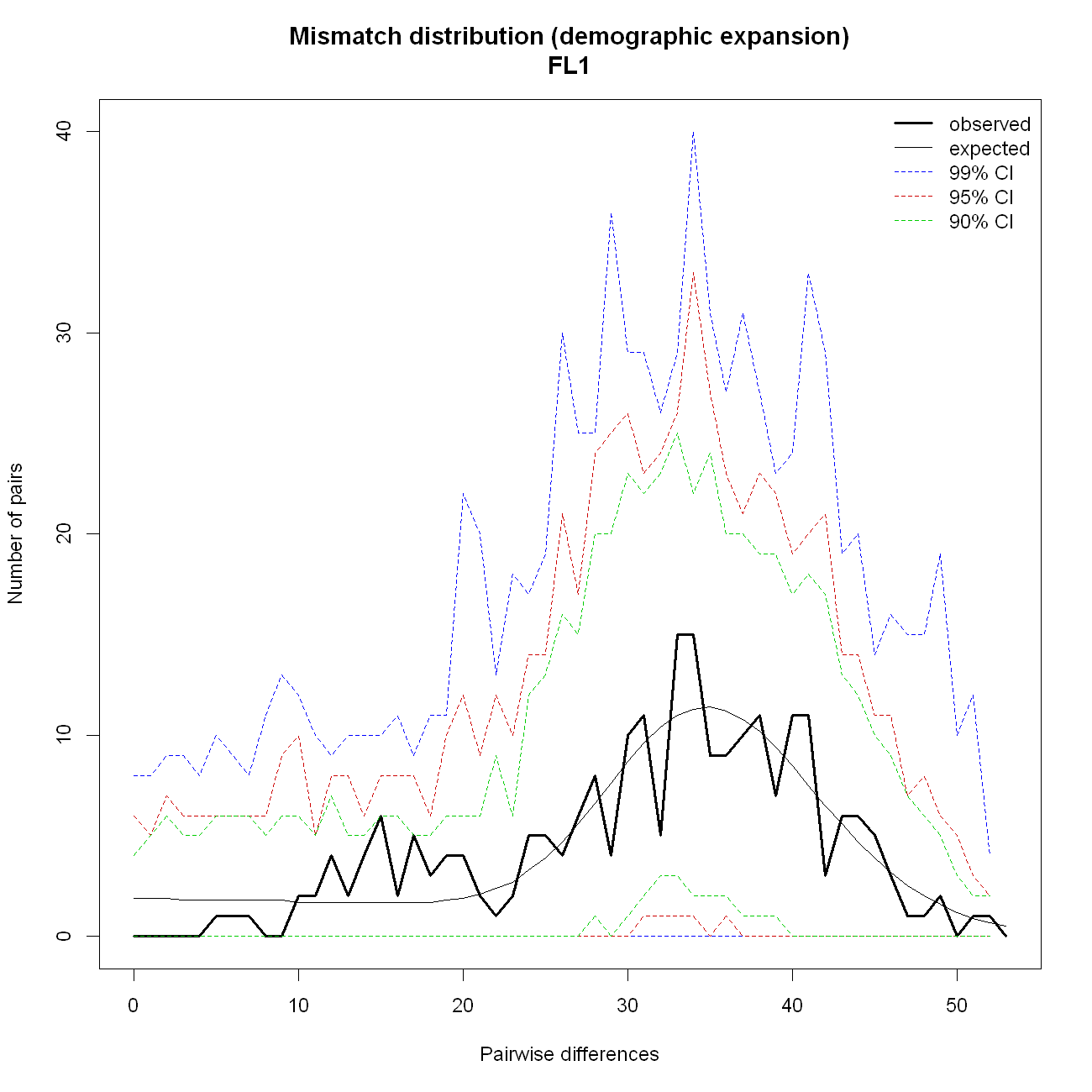

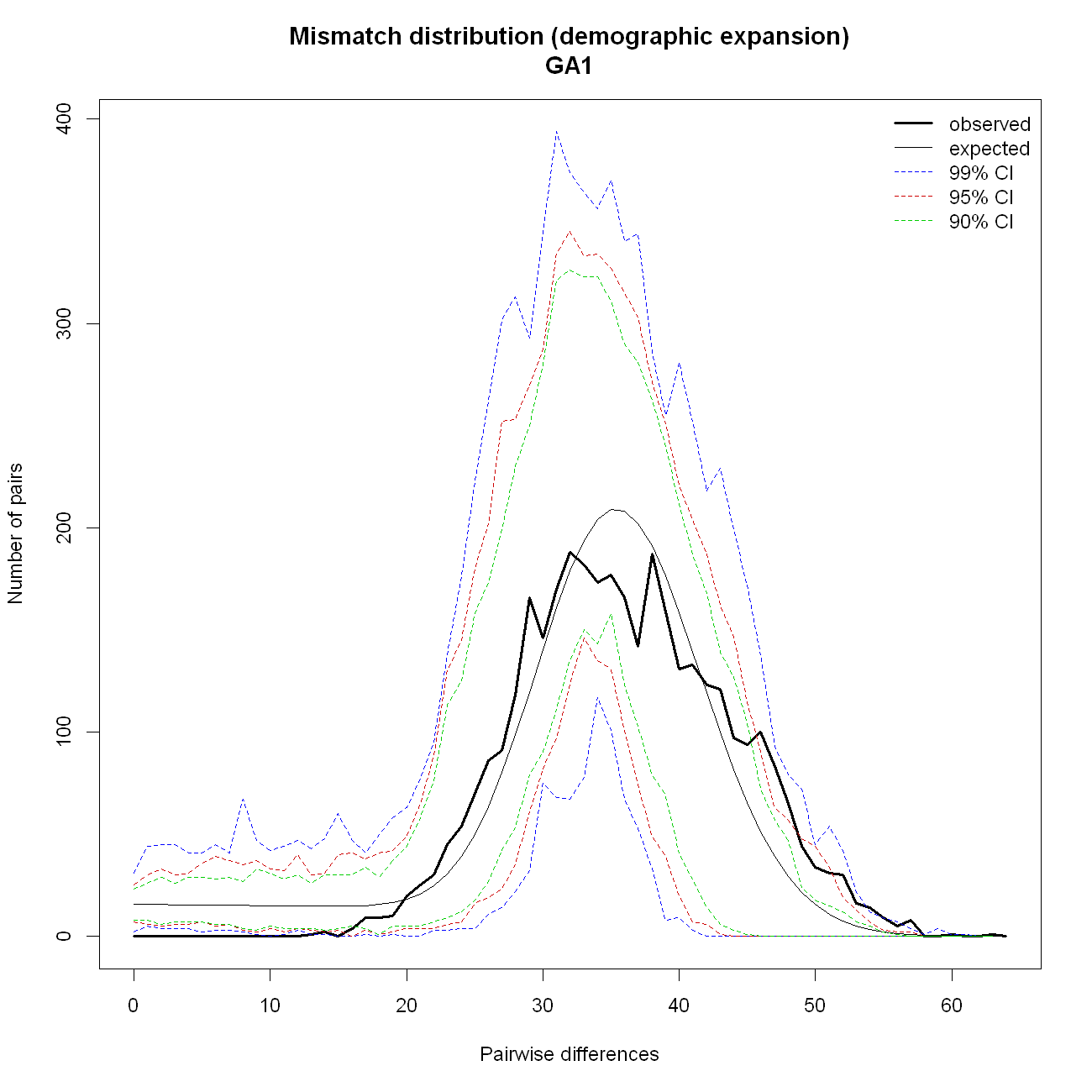

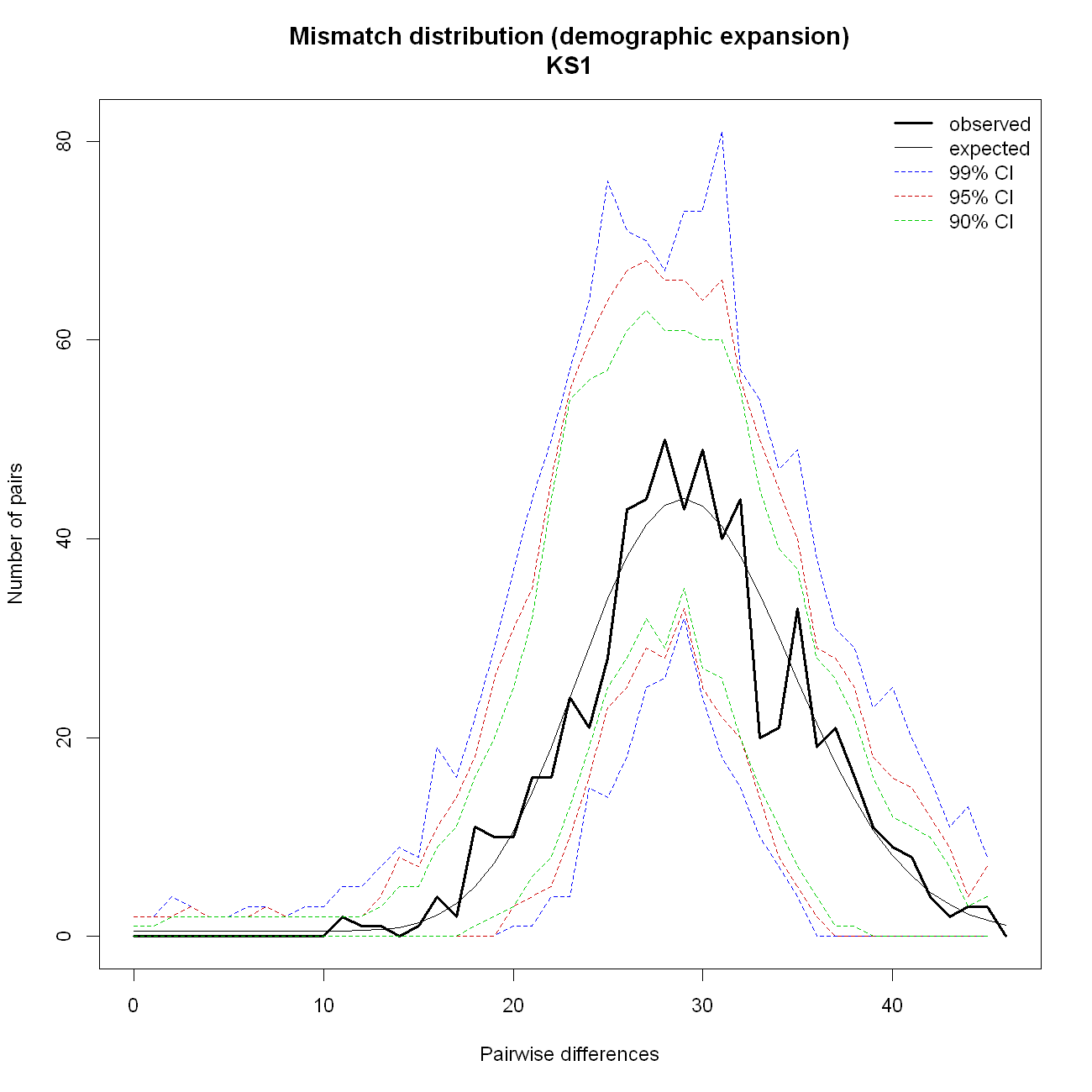

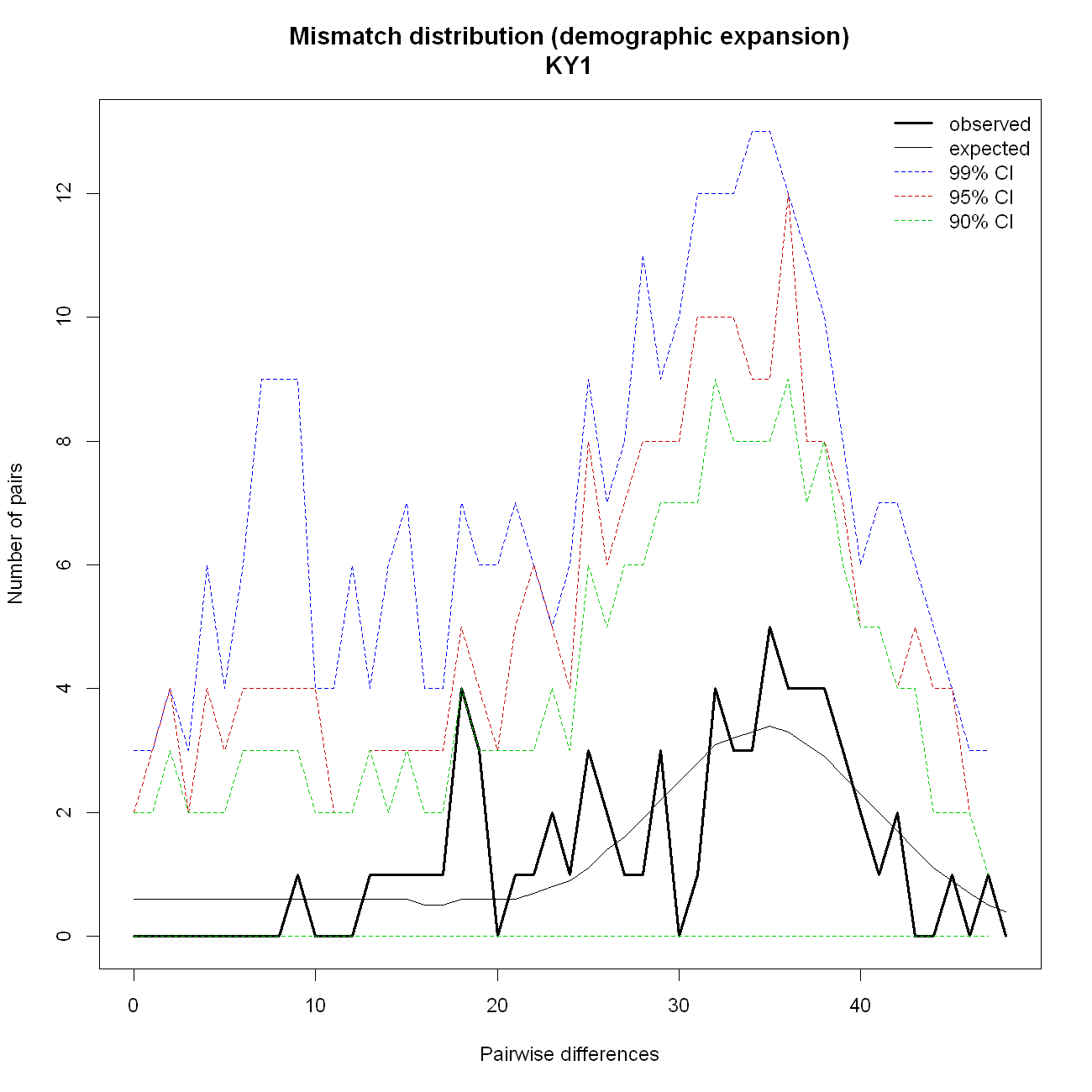

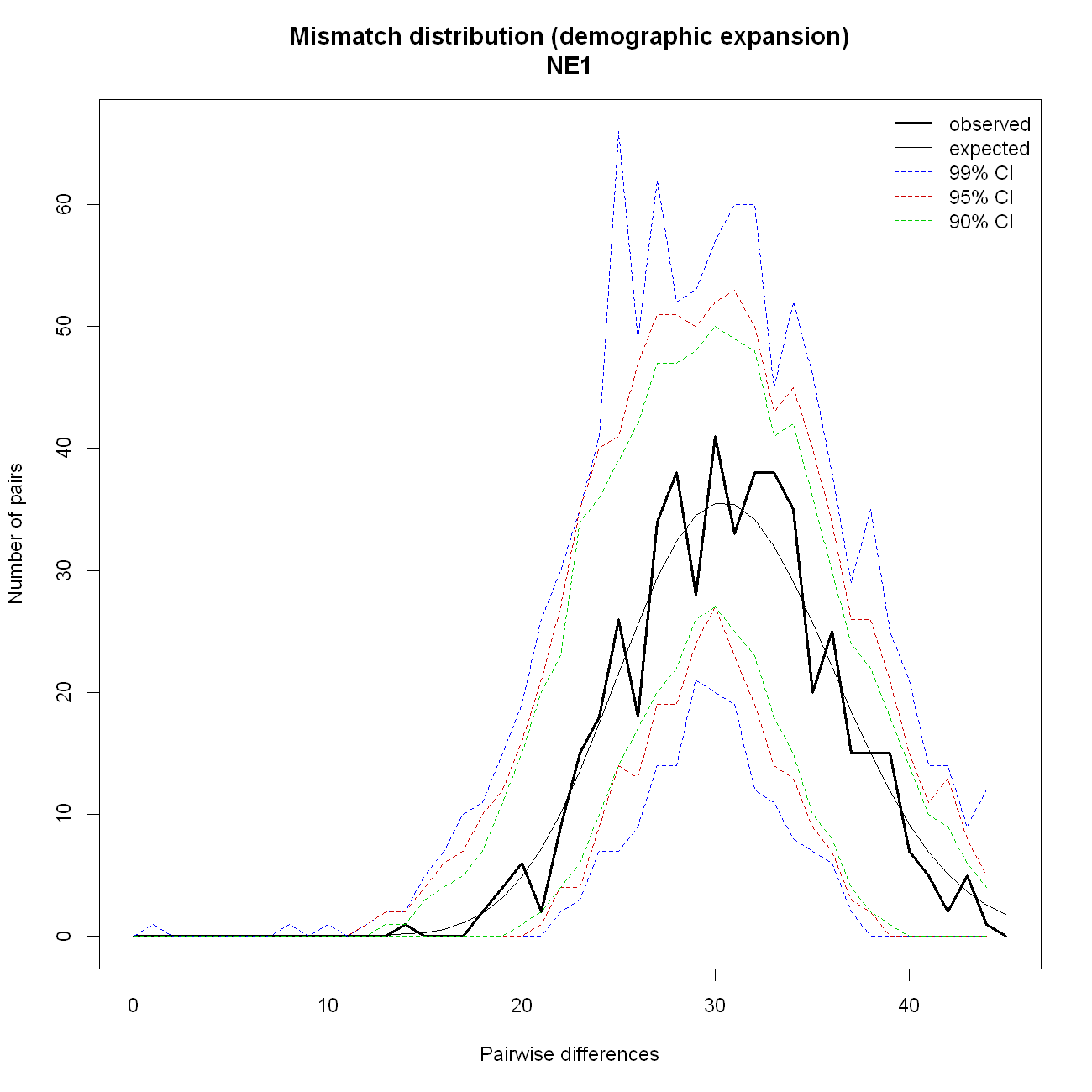

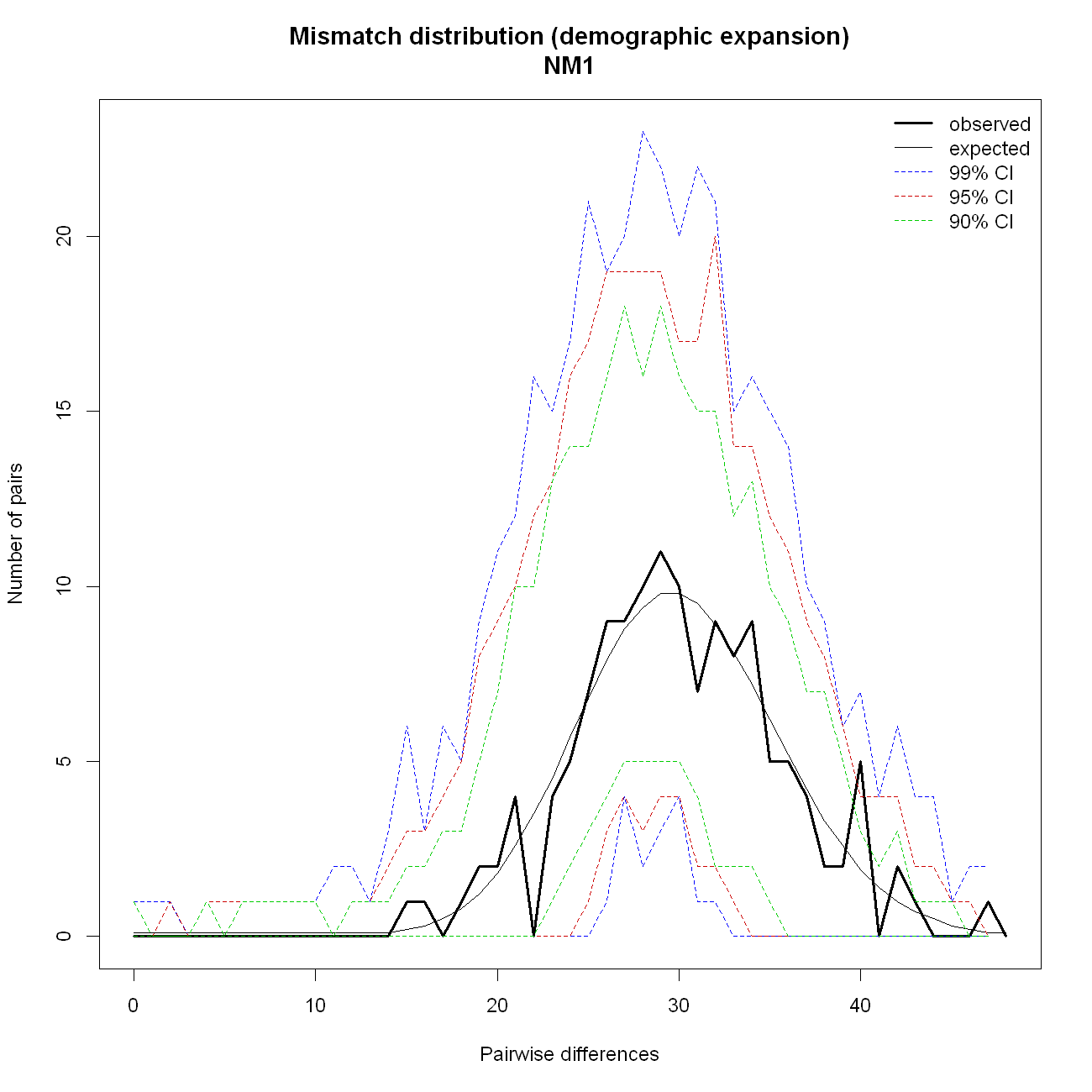

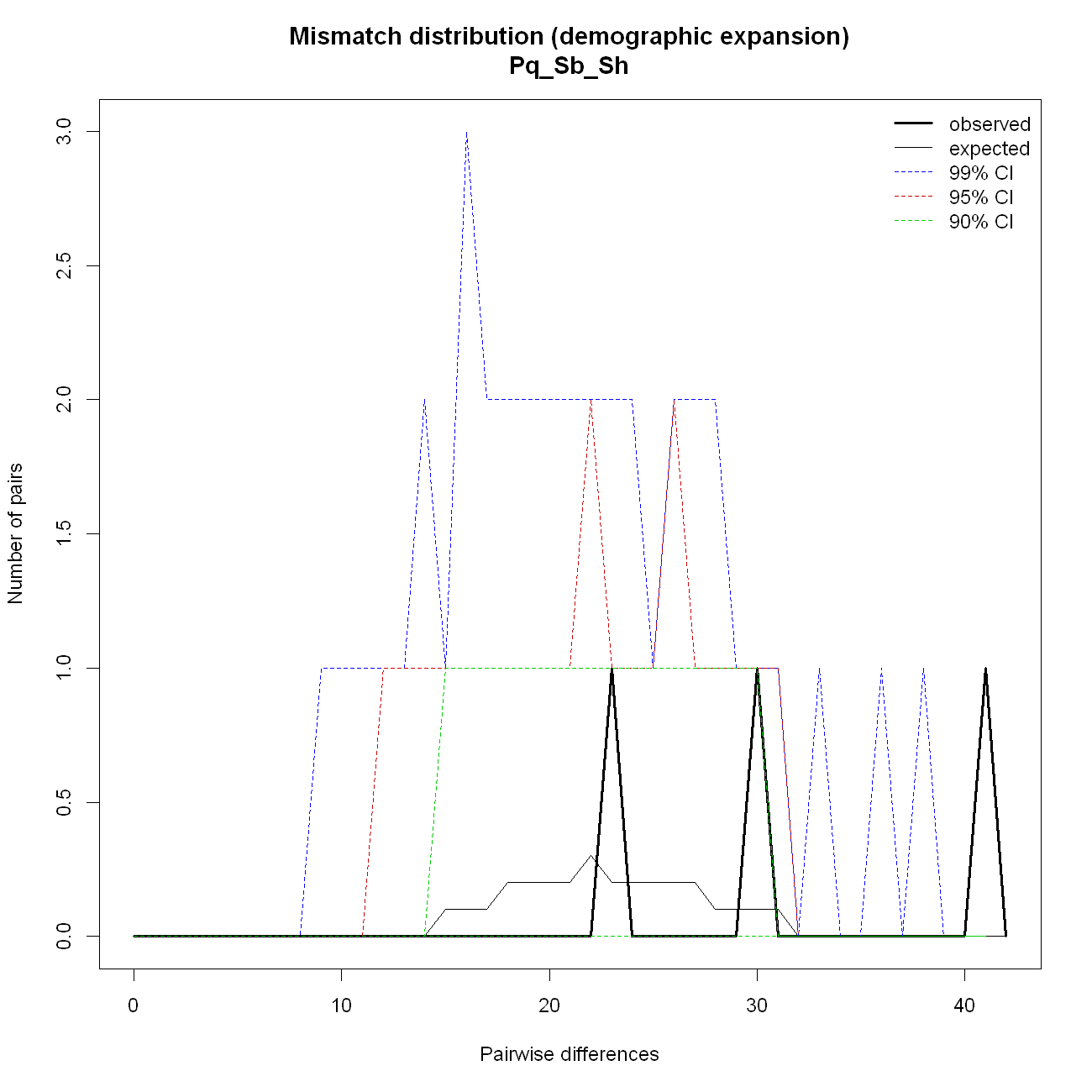

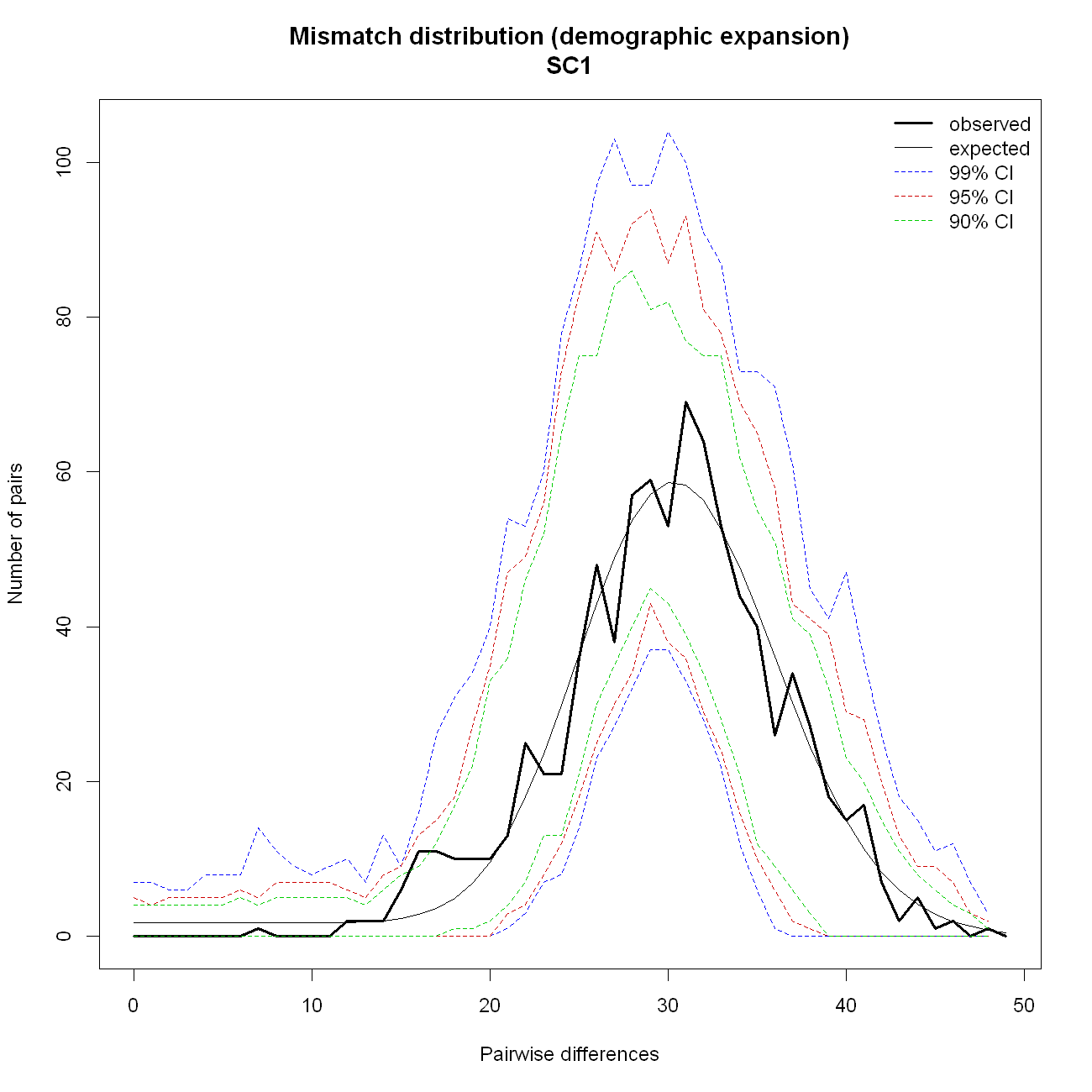

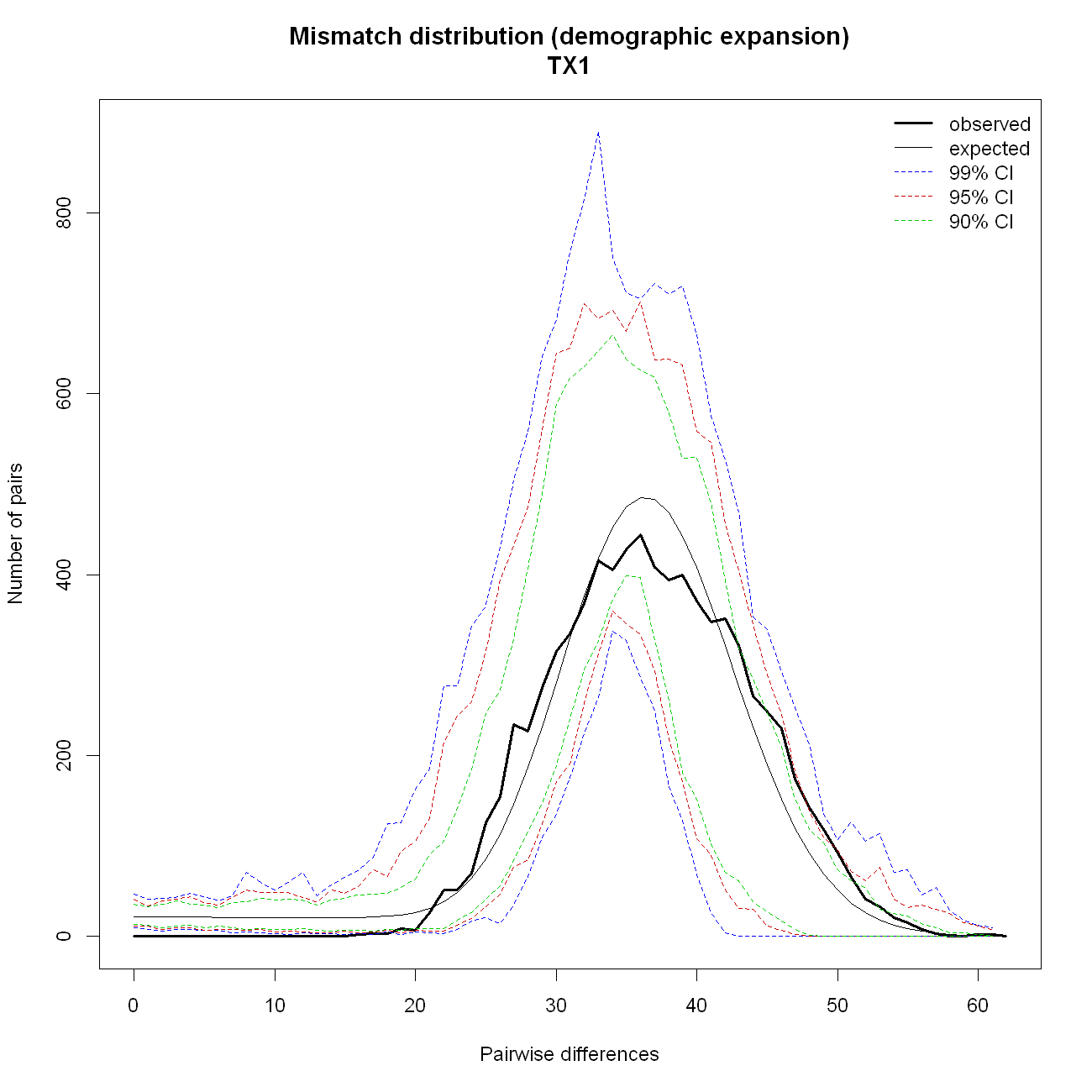

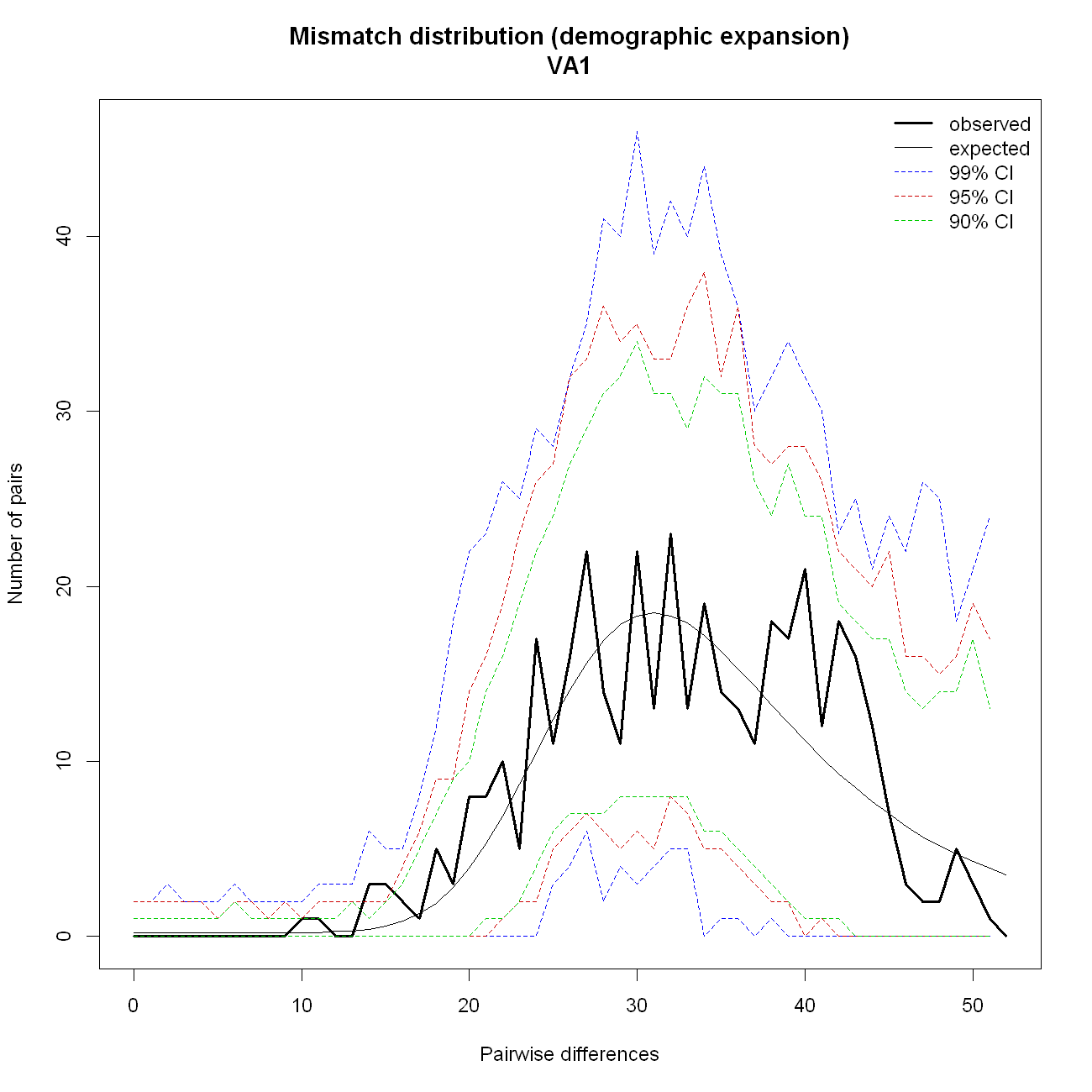


**Fig. S3** Mismatch distribution profiles (demographic expansion) from 12 states and parental genotypes (Sp-Sb) with laboratory reference genotype Gypsum9E (Sh).


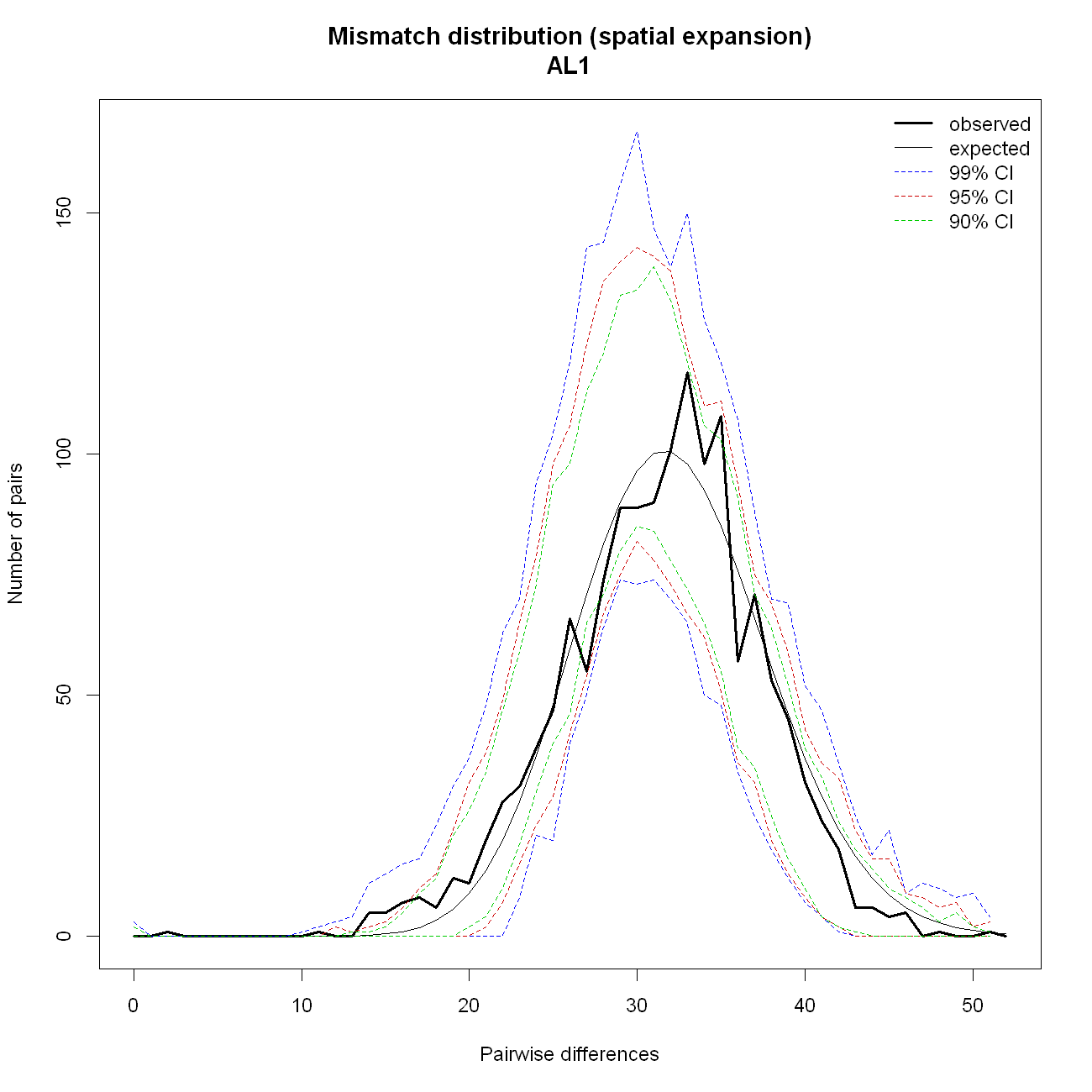

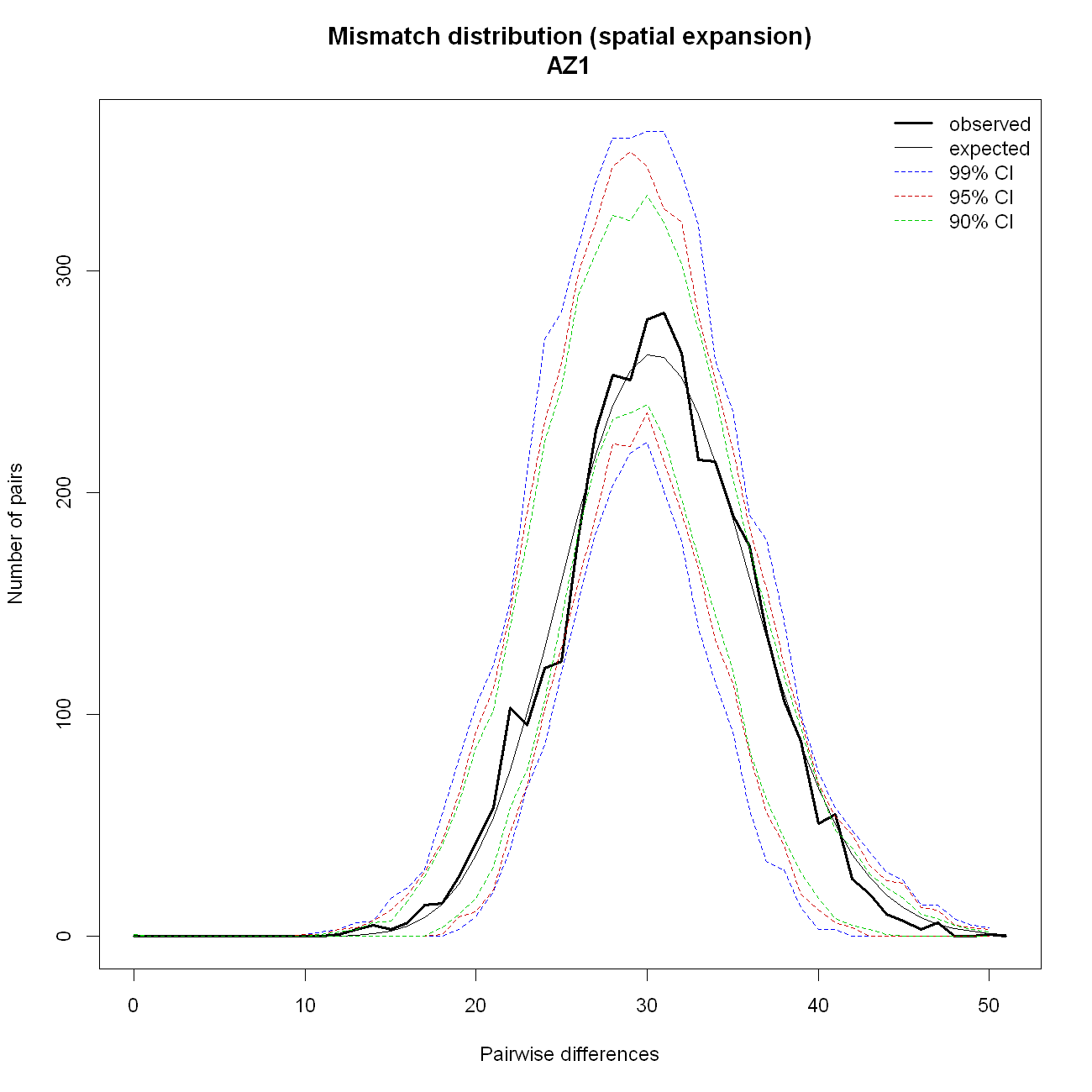

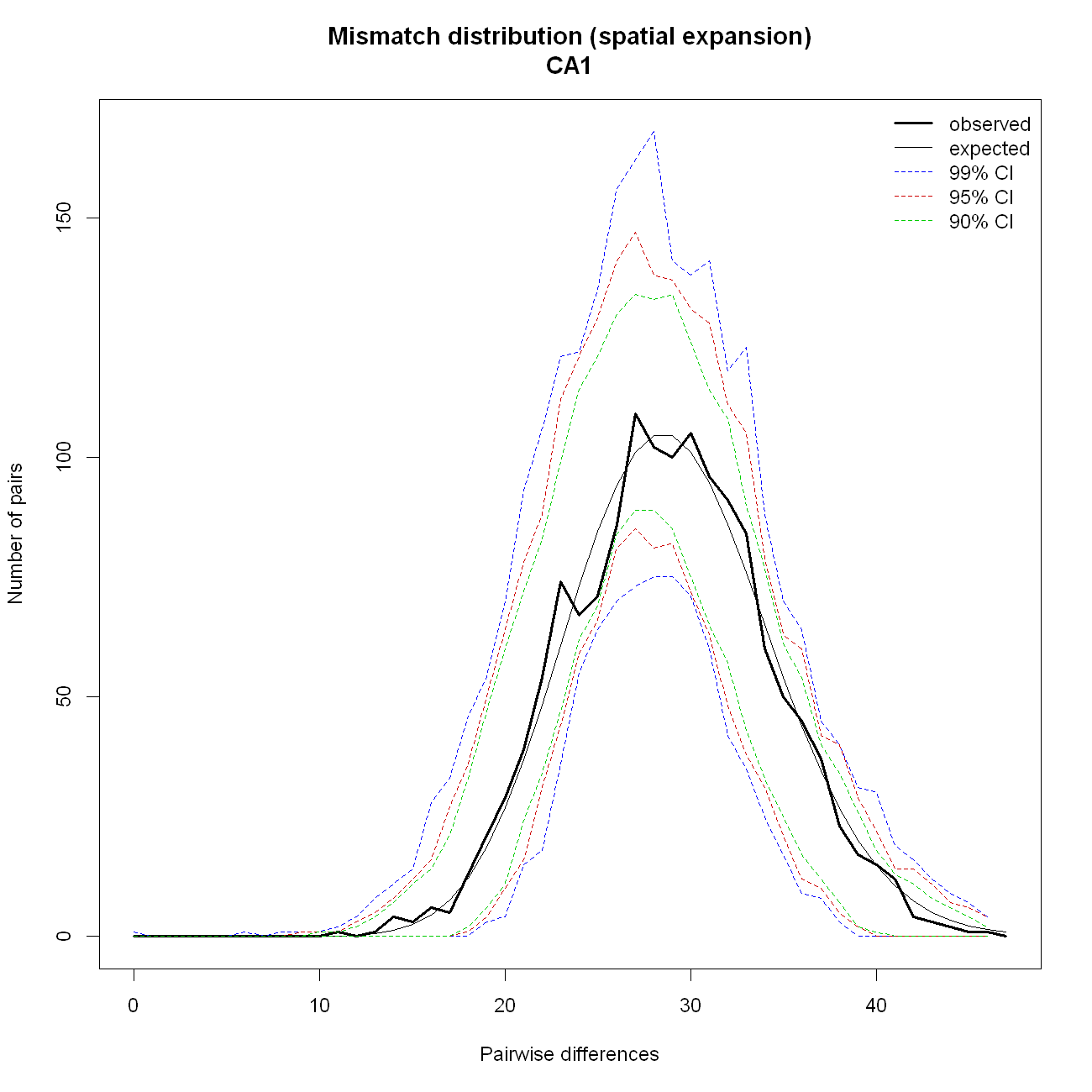

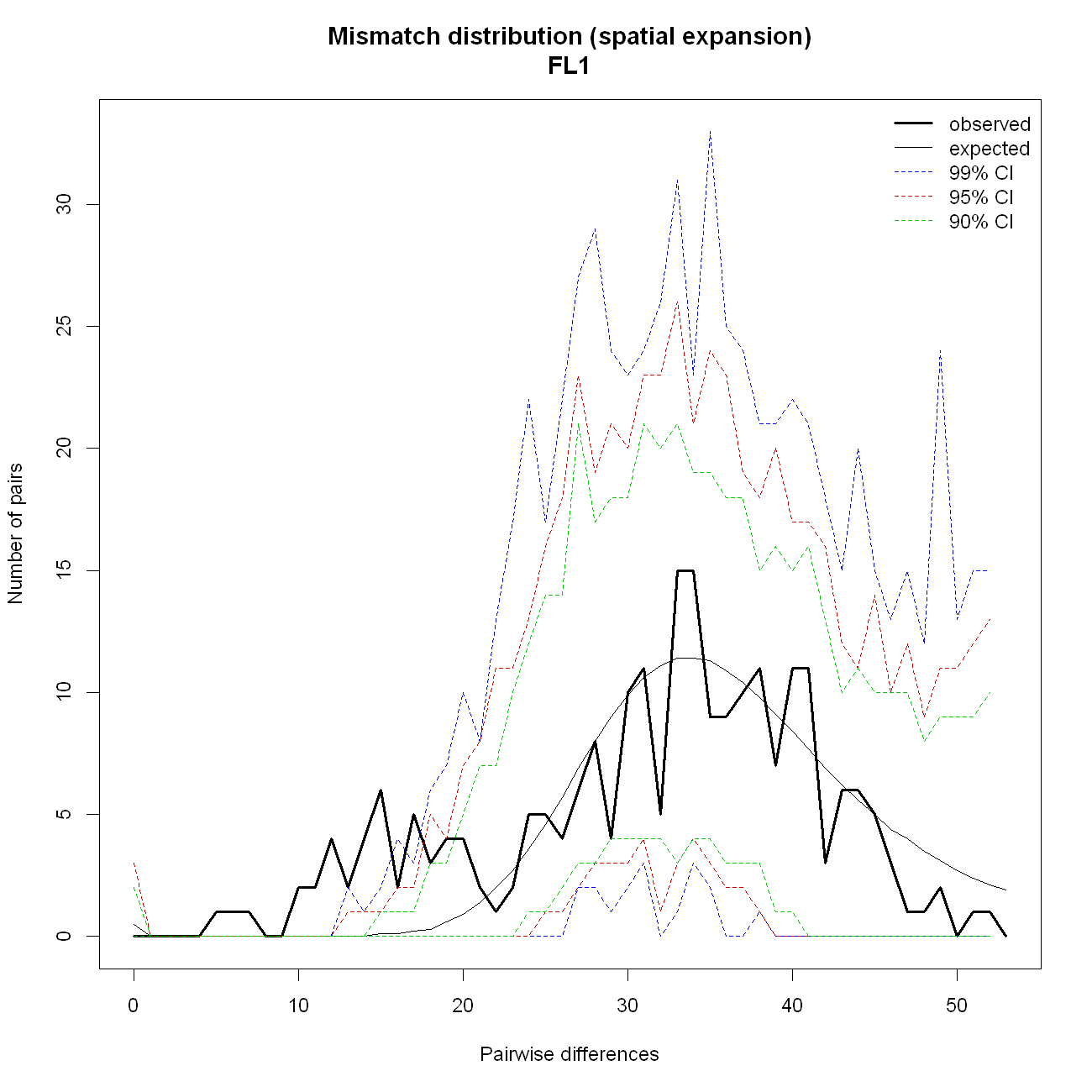

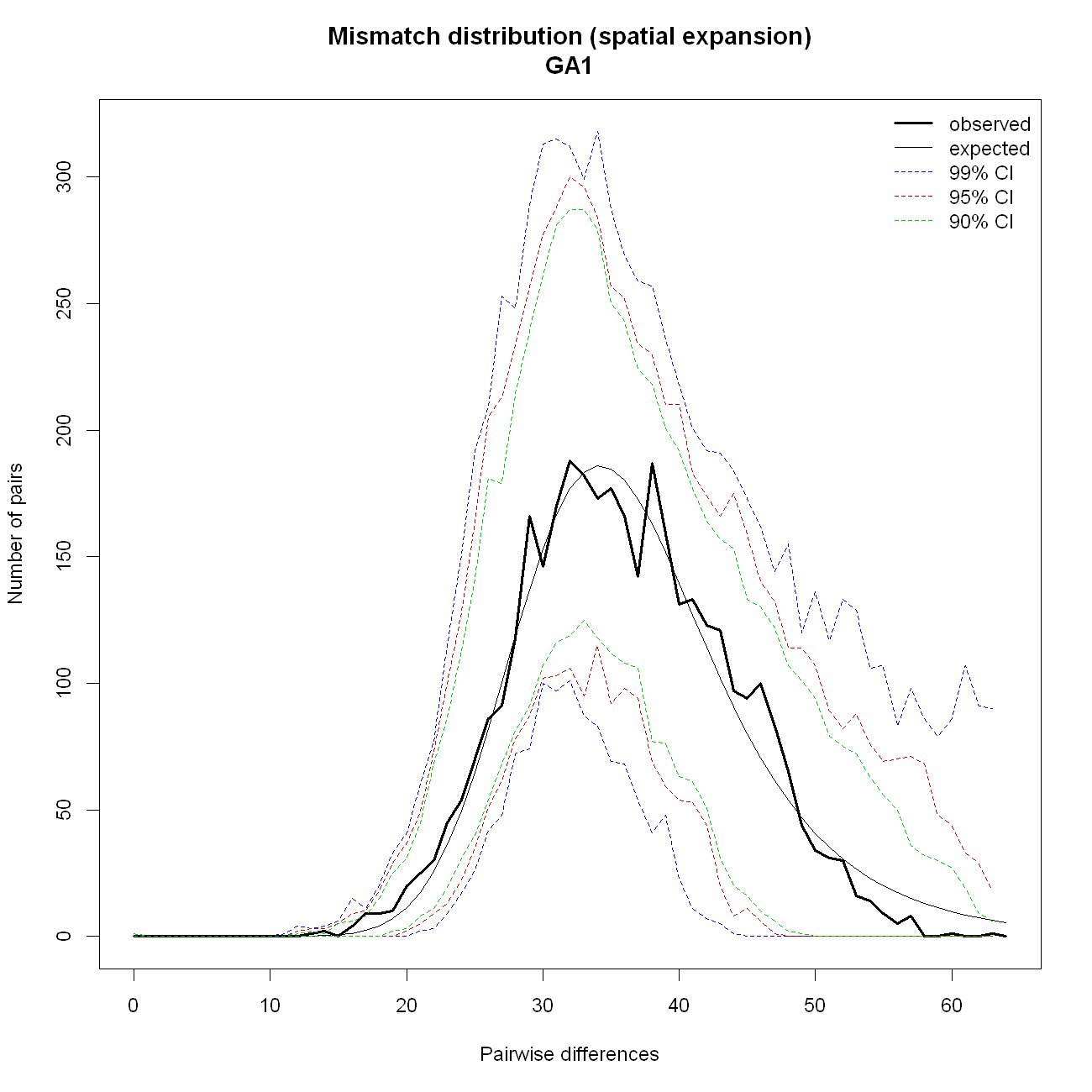

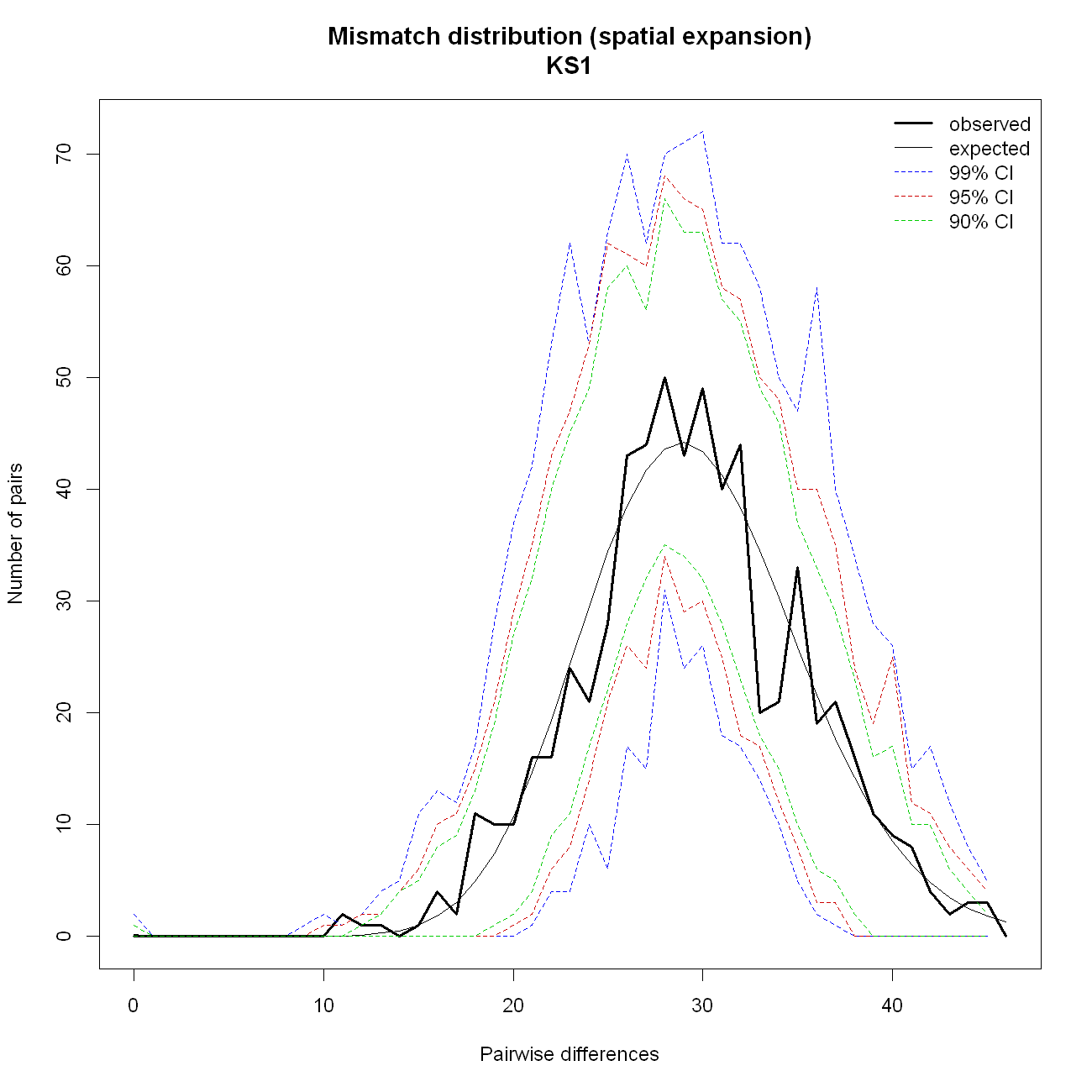

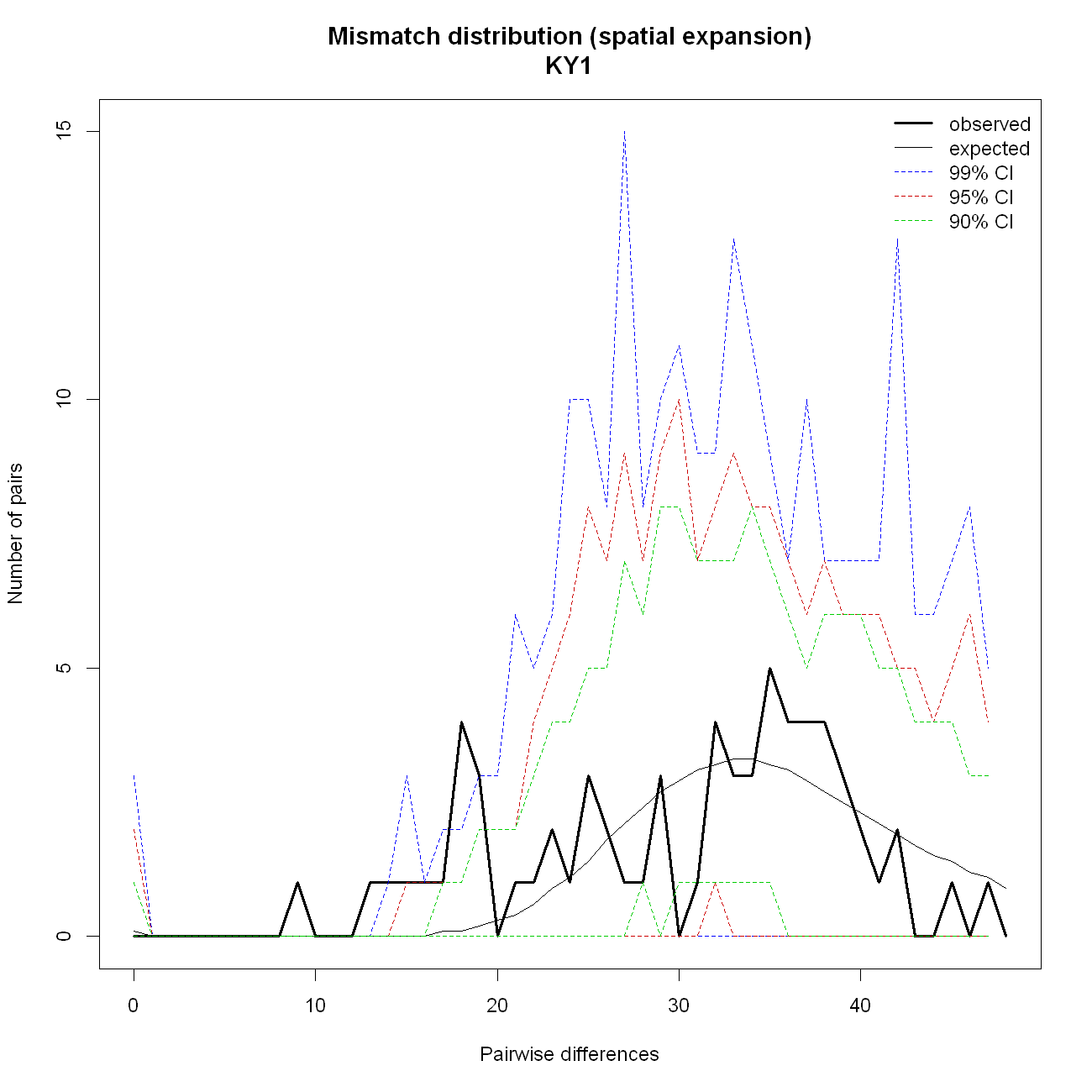

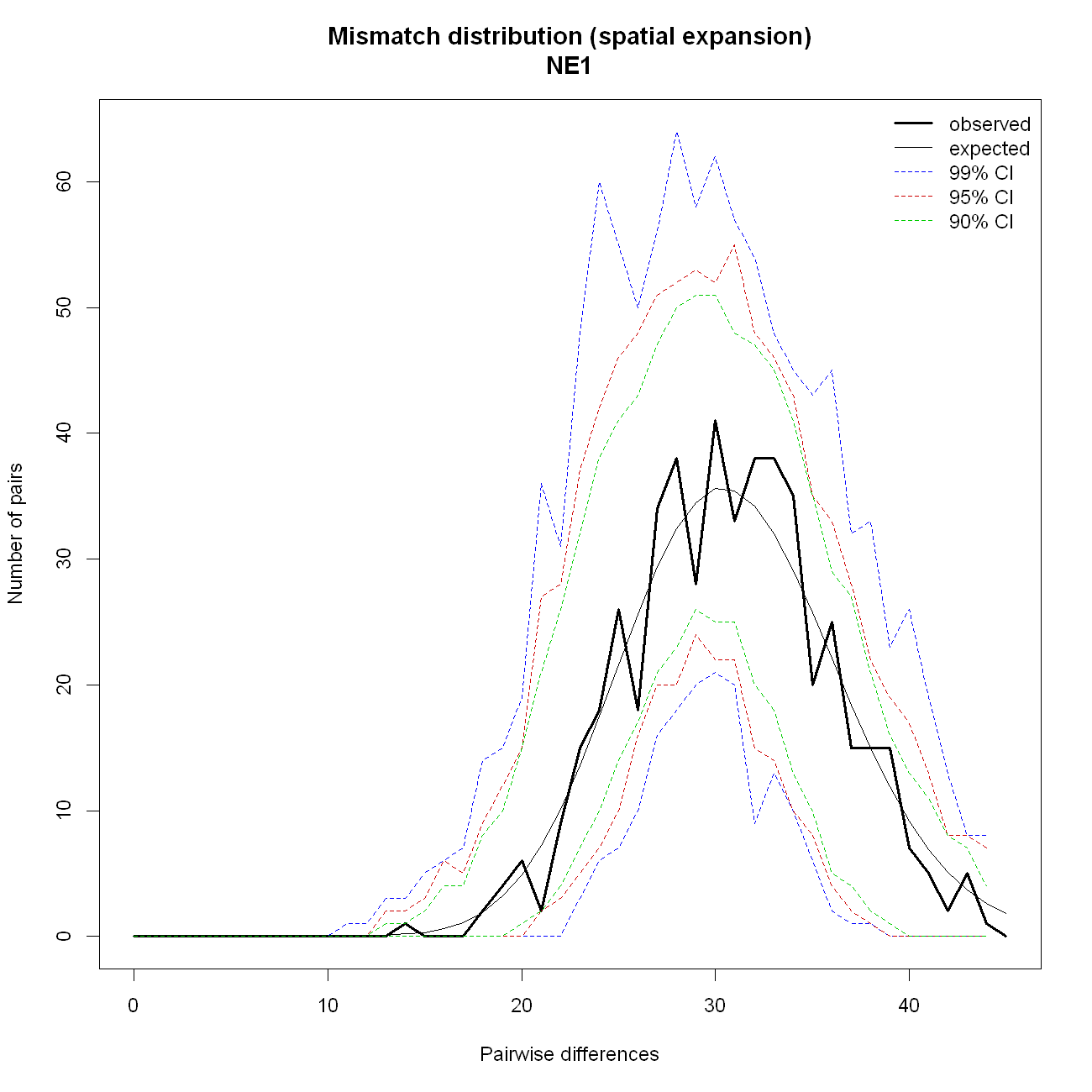

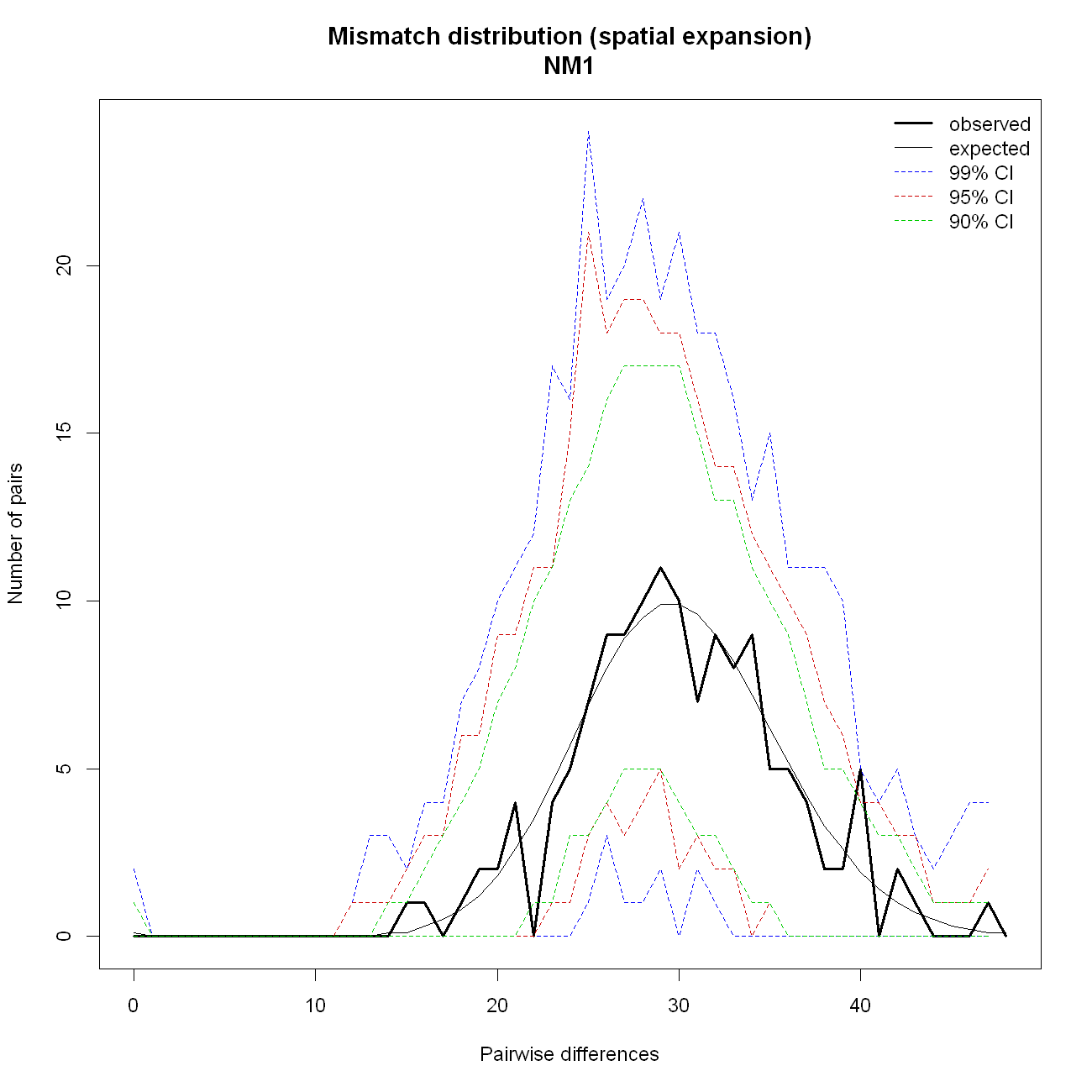

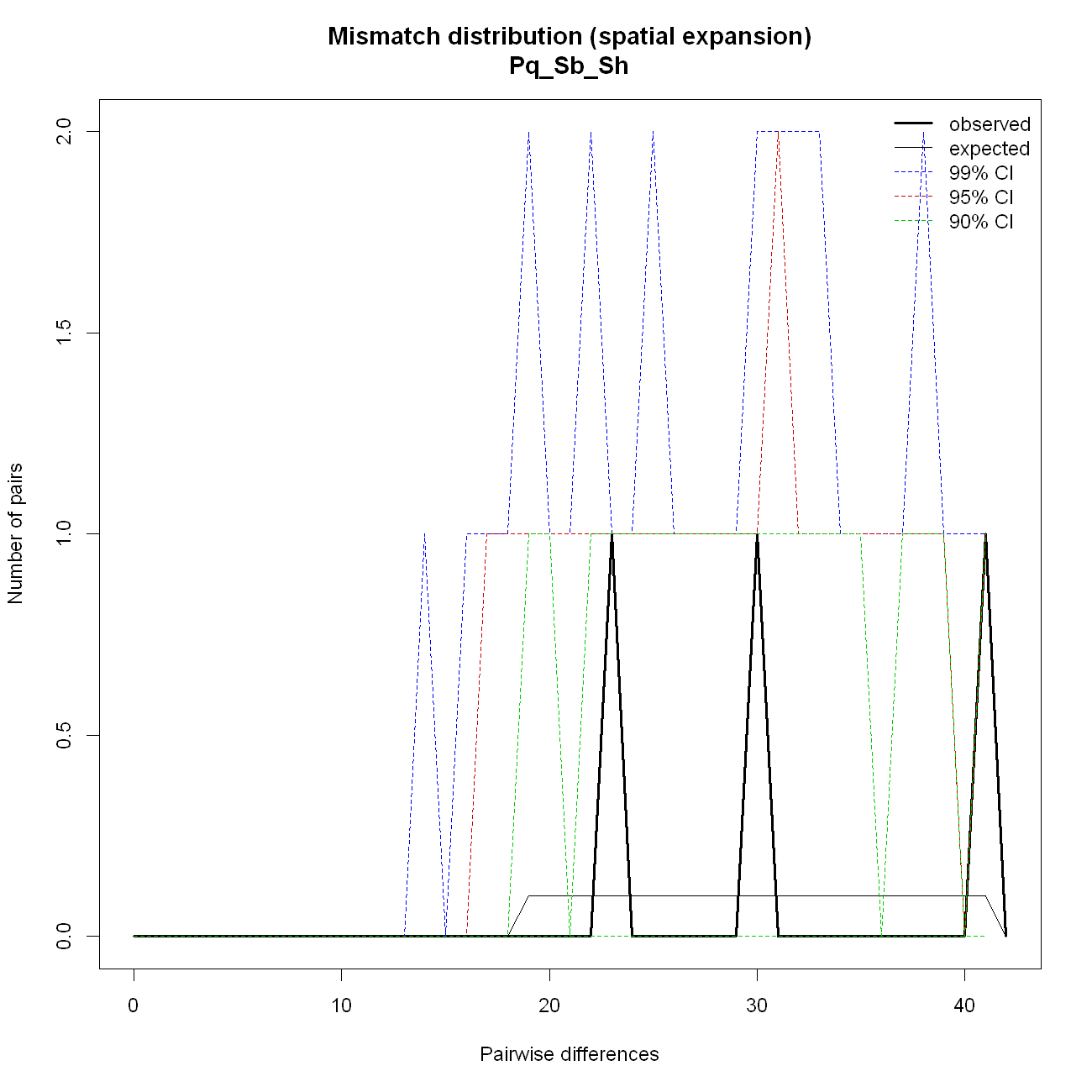

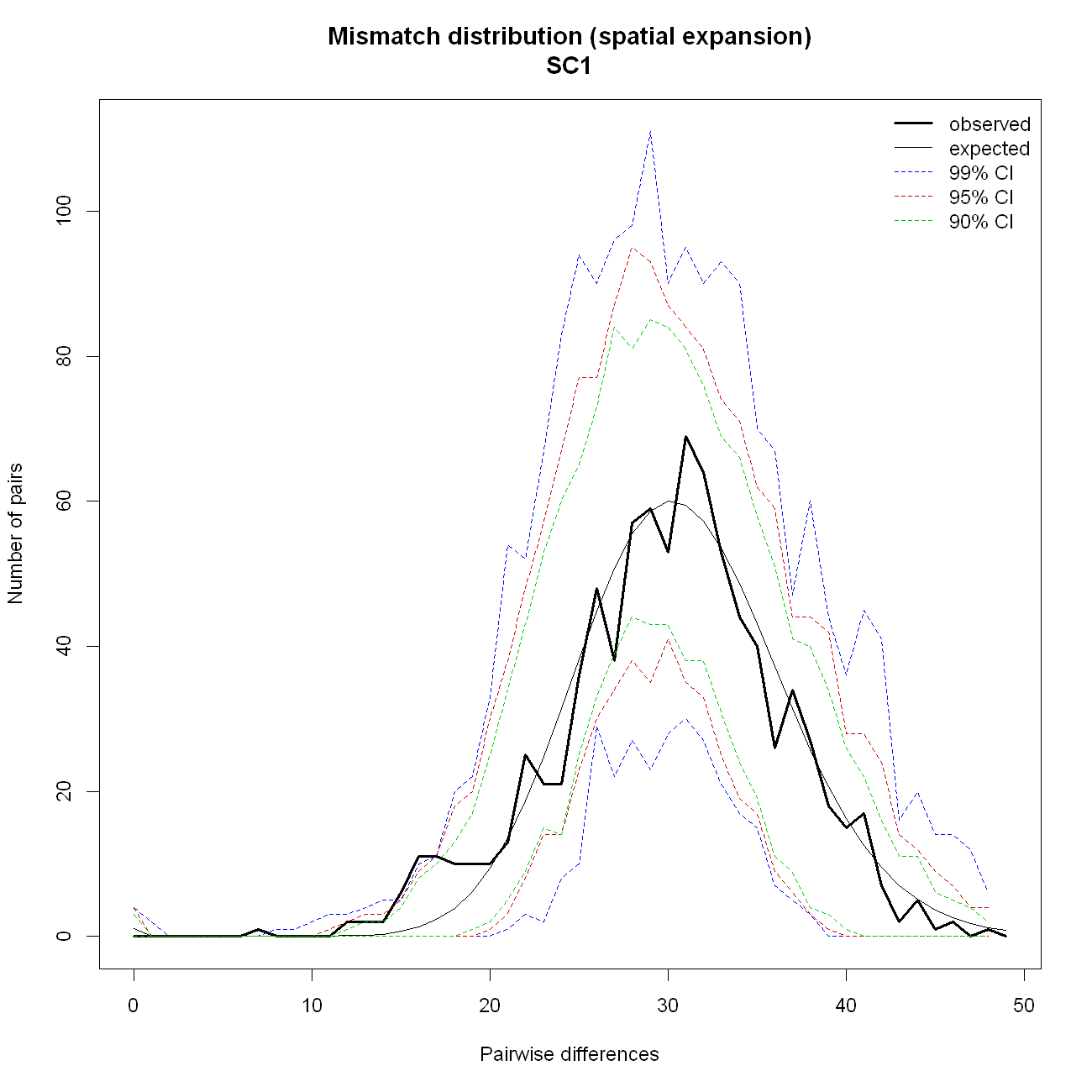

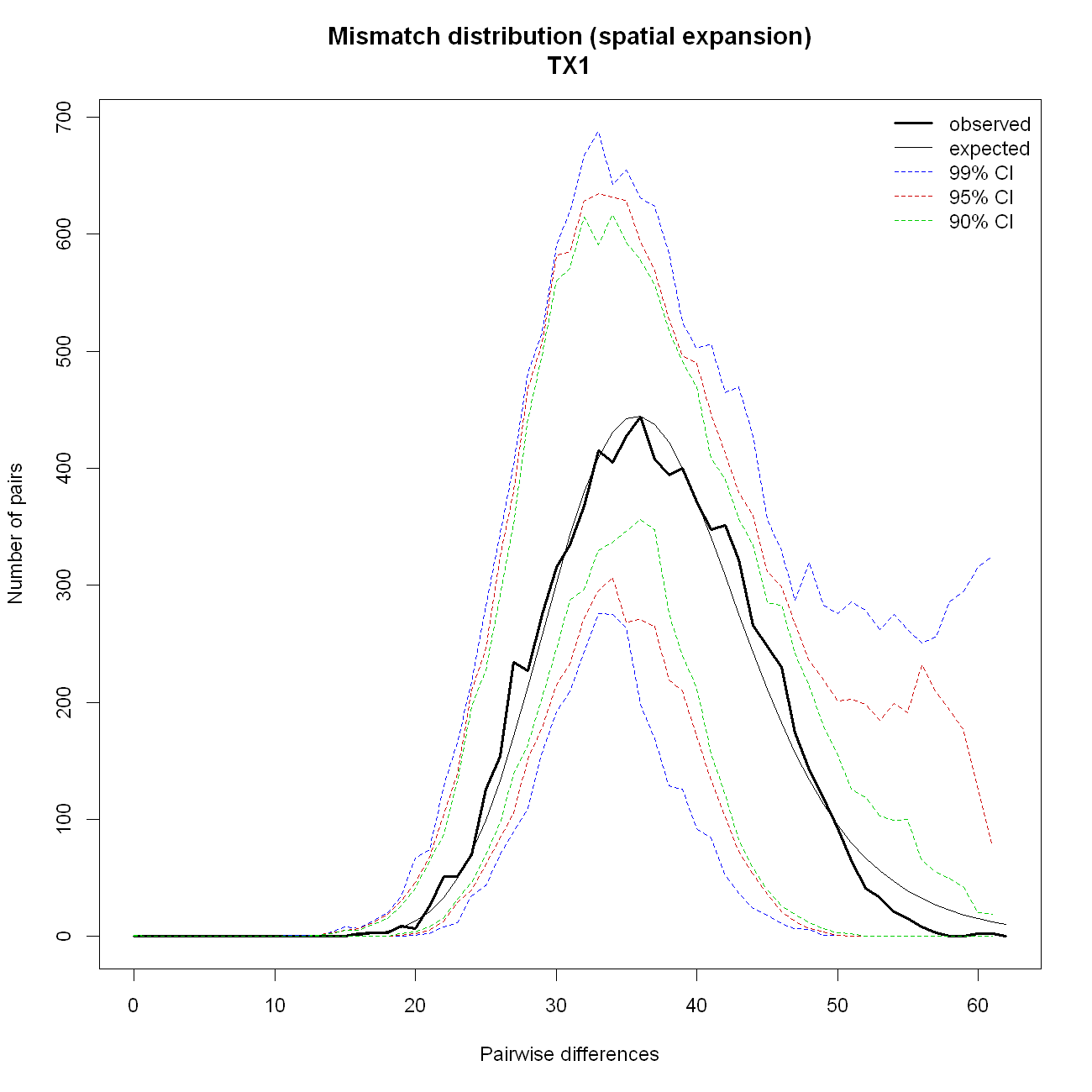

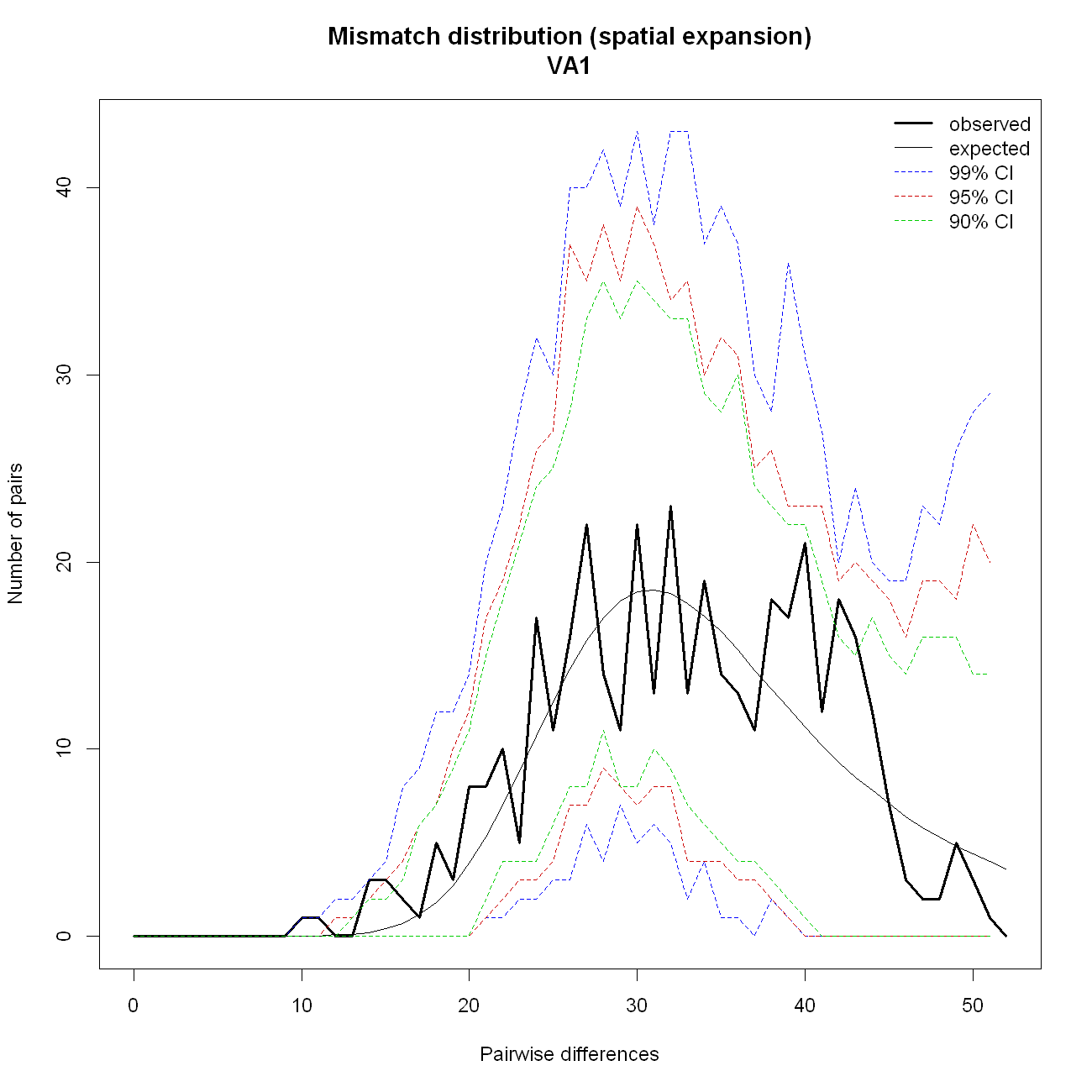


**Fig. S4** Mismatch distribution profiles (spatial expansion) from 12 states and parental genotypes (Sp-Sb) with laboratory reference genotype Gypsum9E (Sh).


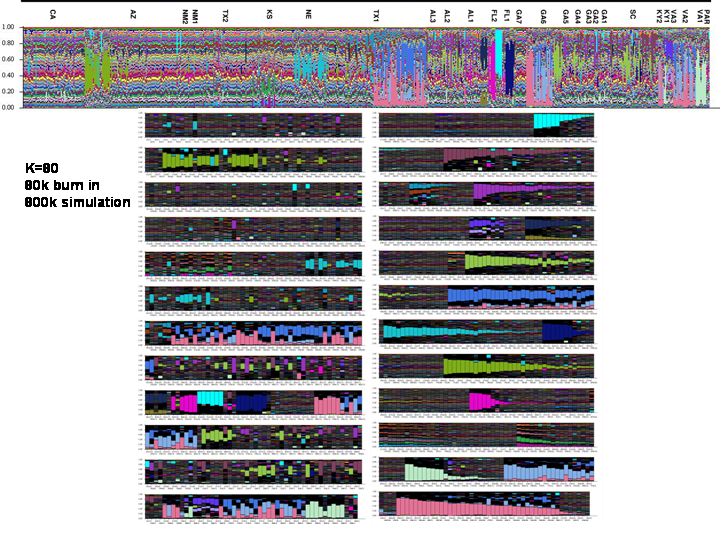


**Fig. S5** STRUCTURE analysis of genotypes at K=80. Bar plot showing 12 states (Top) and individual genotypes (Lower left) with matching colors. Same data reorganized according to their ancestral coefficient to form gradient (Lower right).

**Fig. S6** Neutrality test results after 10000 simulations. Only 7 out of 97 loci are found outside the 95% confidence intervals.
