# Supplementary material for: Multi-Phase US Spread and Habitat Switching of a Post-Columbian Invasive, Sorghum halepense
Source: PLoS One. 2016 Oct 18;11(10):e0164584. doi: 10.1371/journal.pone.0164584 (PMC5068735; doi:10.1371/journal.pone.0164584)
Supplement: S1 Table — (DOCX) [file pone.0164584.s003.docx]

**Table S1** Microsatellite markers developed by ([Casa *et al.*, 2005](#_ENREF_1)).

Chr1 Xgap256, Xcup44

Chr2 CA120676a, CA154181a

Chr3 CA207636a, CA146183a

Chr4 TC69322d, Xcup28

Chr5 mSbCIR329, CA100232a

Chr6 CA098760a,

Chr7 CA193820b, mSbCIR300

Chr8 Xtxp047, Xtxp210

Chr9 TC50663d, TC65153a

Chr10 CA217392a, Xcup43
