# Supplementary material for: Multi-Phase US Spread and Habitat Switching of a Post-Columbian Invasive, Sorghum halepense
Source: PLoS One. 2016 Oct 18;11(10):e0164584. doi: 10.1371/journal.pone.0164584 (PMC5068735; doi:10.1371/journal.pone.0164584)
Supplement: S3 Table — (DOCX) [file pone.0164584.s005.docx]

**Table S3** Test for Linkage Disequilibrium for non-random association between pairs of loci in finite subdivided populations (Ohta 1982):

(DIT)² (DIS)² (D'IS)² (DST)² (D'ST)²

Overall Average 0.08470 0.00815 0.07947 0.07660 0.00523

Total variance (DIT)² is small (0.0847) indicating large human-assisted migration rates on continental scale (among states) reducing LD and the variance components (D'IS)² and (D'ST)² are additive (0.07947 + 0.00523 = 0.0847). The fact that both relationships (D'IS)² > (D'ST)² and (DST)² > (DIS)² hold provide additional indication for reduced migration within populations in most of the 12 states .

Tabular description of every pairwise comparison between loci is presented in **Table S4**.
