# Supplementary material for: Multi-Phase US Spread and Habitat Switching of a Post-Columbian Invasive, Sorghum halepense
Source: PLoS One. 2016 Oct 18;11(10):e0164584. doi: 10.1371/journal.pone.0164584 (PMC5068735; doi:10.1371/journal.pone.0164584)
Supplement: S4 Table — (DOCX) [file pone.0164584.s006.docx]

**Table S4**: Tabular description of every pairwise comparison between loci summarized in **Table S3**.

***********************************************************************************

** **

** Ohta's two-locus analysis of population subdivision **

** [see Genetics 101:139-155 & PNAS 79:1940-1944] **

** **

***********************************************************************************

==========================================================================================

Locus_A - Locus_B (DIT)² (DIS)² (D'IS)² (DST)² (D'ST)²

==========================================================================================

M01 - M02 0.06396 0.03057 0.05943 0.03137 0.00454

M01 - M03 0.06636 0.02107 0.06511 0.04293 0.00125

M01 - M04 0.05834 0.02008 0.05725 0.03058 0.00109

M01 - M05 0.06857 0.00635 0.06309 0.05871 0.00547

M01 - M06 0.06739 0.00227 0.06426 0.06536 0.00313

M01 - M07 0.08275 0.00715 0.08207 0.07592 0.00068

M01 - M08 0.07554 0.00648 0.06974 0.06686 0.00580

M01 - M09 0.09428 0.00523 0.08927 0.08717 0.00501

M01 - M10 0.21788 0.00185 0.21157 0.21969 0.00631

M01 - M11 0.14339 0.02001 0.13338 0.11671 0.01000

M01 - M12 0.06659 0.00047 0.06549 0.06627 0.00109

M01 - M13 0.11745 0.00194 0.11301 0.11778 0.00444

M01 - M14 0.13592 0.00324 0.13203 0.13233 0.00388

M01 - M15 0.04186 0.00018 0.04031 0.04231 0.00155

M01 - M16 0.12761 0.00334 0.12354 0.12230 0.00407

M01 - M17 0.05217 0.00227 0.04827 0.04976 0.00390

M01 - M18 0.06039 0.00618 0.05884 0.05787 0.00155

M01 - M19 0.08252 0.01714 0.08014 0.06128 0.00238

M01 - M20 0.04380 0.00382 0.03942 0.04516 0.00438

M01 - M21 0.05292 0.01828 0.05035 0.03670 0.00257

M01 - M22 0.05569 0.00529 0.05407 0.05391 0.00162

M01 - M23 0.08479 0.00621 0.08110 0.07627 0.00370

M01 - M24 0.07638 0.01009 0.06822 0.06961 0.00816

M01 - M25 0.12162 0.00745 0.10964 0.11679 0.01198

M01 - M26 0.05088 0.00274 0.04831 0.04711 0.00257

M01 - M27 0.05509 0.01167 0.05307 0.04857 0.00201

M01 - M28 0.06760 0.01137 0.06200 0.06025 0.00560

M01 - M29 0.04473 0.00464 0.04270 0.04535 0.00203

M01 - M30 0.06927 0.01871 0.06514 0.04876 0.00413

M01 - M31 0.06539 0.01705 0.05933 0.04810 0.00606

M01 - M32 0.08782 0.01748 0.08236 0.07174 0.00546

M01 - M33 0.06058 0.01925 0.05813 0.04069 0.00244

M01 - M34 0.08209 0.00075 0.08067 0.07919 0.00142

M01 - M35 0.06957 0.00945 0.06756 0.06656 0.00201

M01 - M36 0.04974 0.00580 0.04803 0.04796 0.00171

M01 - M37 0.06463 0.00739 0.06293 0.06172 0.00169

M01 - M38 0.08647 0.01852 0.08382 0.06791 0.00265

M01 - M39 0.04028 0.00028 0.03913 0.04079 0.00115

M01 - M40 0.06016 0.01255 0.05367 0.04430 0.00649

M01 - M41 0.04617 0.00430 0.04082 0.04619 0.00535

M01 - M42 0.06675 0.01166 0.06370 0.05353 0.00305

M01 - M43 0.10866 0.01788 0.10535 0.08273 0.00332

M01 - M44 0.04857 0.01025 0.04685 0.03795 0.00172

M01 - M45 0.08411 0.00048 0.08260 0.08241 0.00152

M01 - M46 0.03801 0.00083 0.03724 0.03882 0.00077

M01 - M47 0.07522 0.00625 0.07010 0.07190 0.00512

M01 - M48 0.08478 0.00384 0.07845 0.08463 0.00633

M01 - M49 0.09209 0.00697 0.08729 0.08524 0.00480

M01 - M50 0.06742 0.00233 0.06671 0.06350 0.00071

M01 - M51 0.04474 0.02781 0.04278 0.02147 0.00196

M01 - M52 0.05857 0.03353 0.05489 0.03043 0.00368

M01 - M53 0.06756 0.00466 0.06344 0.06243 0.00412

M01 - M54 0.05037 0.00623 0.04846 0.04545 0.00191

M01 - M55 0.05636 0.02432 0.05358 0.03489 0.00278

M01 - M56 0.06559 0.02364 0.06323 0.03579 0.00236

M01 - M57 0.03638 0.00873 0.03555 0.03208 0.00084

M01 - M58 0.04501 0.00137 0.04243 0.04444 0.00258

M01 - M59 0.04696 0.00641 0.04358 0.04540 0.00338

M01 - M60 0.04954 0.00301 0.04810 0.04914 0.00144

M01 - M61 0.05753 0.00731 0.05254 0.05406 0.00499

M01 - M62 0.04858 0.00299 0.04759 0.04503 0.00099

M01 - M63 0.04685 0.00235 0.04585 0.04420 0.00100

M01 - M64 0.06474 0.01887 0.05954 0.04374 0.00520

M01 - M65 0.06653 0.00312 0.05974 0.06577 0.00678

M01 - M66 0.06680 0.00798 0.06575 0.06138 0.00105

M01 - M67 0.06593 0.00686 0.06477 0.06286 0.00116

M01 - M68 0.06193 0.02462 0.05053 0.03360 0.01141

M01 - M69 0.04644 0.00324 0.04458 0.04673 0.00186

M01 - M70 0.05775 0.01089 0.05348 0.05154 0.00427

M01 - M71 0.07054 0.02398 0.06579 0.04659 0.00475

M01 - M72 0.06065 0.02063 0.05968 0.03250 0.00097

M01 - M73 0.04453 0.00461 0.04149 0.04506 0.00304

M01 - M74 0.04631 0.00280 0.04550 0.04384 0.00081

M01 - M75 0.04275 0.00611 0.03795 0.04290 0.00480

M01 - M76 0.08139 0.00473 0.07900 0.07782 0.00239

M01 - M77 0.06855 0.02826 0.06056 0.03962 0.00799

M01 - M78 0.03914 0.00183 0.03795 0.04019 0.00120

M01 - M79 0.04518 0.00359 0.04333 0.04477 0.00185

M01 - M80 0.07099 0.03133 0.06347 0.03442 0.00752

M01 - M81 0.05219 0.00828 0.04870 0.04663 0.00348

M01 - M82 0.06355 0.00700 0.05776 0.05916 0.00579

M01 - M83 0.07383 0.00635 0.07088 0.07121 0.00295

M01 - M84 0.04585 0.00896 0.04415 0.04132 0.00171

M01 - M85 0.04364 0.00622 0.04131 0.04410 0.00234

M01 - M86 0.08334 0.01767 0.07576 0.06875 0.00759

M01 - M87 0.04427 0.00639 0.04273 0.04401 0.00153

M01 - M88 0.08151 0.01942 0.07768 0.06221 0.00383

M01 - M89 0.10357 0.01921 0.10162 0.08060 0.00196

M01 - M90 0.06332 0.00563 0.06003 0.05741 0.00330

M01 - M91 0.04772 0.00524 0.04597 0.03654 0.00175

M01 - M92 0.08265 0.01888 0.07625 0.05677 0.00641

M01 - M93 0.04839 0.00387 0.04703 0.04179 0.00135

M01 - M94 0.05325 0.00345 0.05101 0.05343 0.00223

M01 - M95 0.06986 0.01823 0.06454 0.05374 0.00532

M01 - M96 0.08042 0.02472 0.07690 0.05124 0.00352

M01 - M97 0.07173 0.01841 0.06667 0.05600 0.00505

M02 - M03 0.06848 0.03025 0.04969 0.03053 0.01879

M02 - M04 0.05357 0.02844 0.04060 0.01828 0.01296

M02 - M05 0.05497 0.01104 0.05065 0.04340 0.00432

M02 - M06 0.05407 0.00873 0.05097 0.04855 0.00311

M02 - M07 0.07757 0.01387 0.07587 0.06146 0.00170

M02 - M08 0.07127 0.01764 0.06860 0.05274 0.00267

M02 - M09 0.09412 0.01757 0.08592 0.07230 0.00820

M02 - M10 0.19848 0.00534 0.19263 0.19484 0.00585

M02 - M11 0.12213 0.02103 0.11269 0.09749 0.00944

M02 - M12 0.05713 0.00683 0.05668 0.05004 0.00046

M02 - M13 0.11210 0.00431 0.10812 0.10340 0.00398

M02 - M14 0.12231 0.00273 0.11954 0.11780 0.00277

M02 - M15 0.02411 0.00163 0.02362 0.02340 0.00049

M02 - M16 0.11006 0.00273 0.10679 0.10798 0.00327

M02 - M17 0.04232 0.00527 0.04012 0.03364 0.00220

M02 - M18 0.04803 0.00818 0.04698 0.04321 0.00105

M02 - M19 0.08363 0.02732 0.08281 0.04740 0.00083

M02 - M20 0.02573 0.00381 0.02481 0.02600 0.00092

M02 - M21 0.04671 0.01978 0.04467 0.02611 0.00204

M02 - M22 0.05028 0.00830 0.04917 0.04082 0.00111

M02 - M23 0.06898 0.00848 0.06608 0.05826 0.00290

M02 - M24 0.06254 0.00403 0.05544 0.05825 0.00711

M02 - M25 0.10518 0.00390 0.09356 0.09922 0.01162

M02 - M26 0.02815 0.00107 0.02673 0.02492 0.00142

M02 - M27 0.05738 0.02134 0.05480 0.03715 0.00258

M02 - M28 0.05541 0.00955 0.05047 0.04880 0.00494

M02 - M29 0.02909 0.00568 0.02875 0.02790 0.00034

M02 - M30 0.05932 0.02241 0.05436 0.03562 0.00496

M02 - M31 0.05597 0.01976 0.05142 0.03532 0.00455

M02 - M32 0.08552 0.02365 0.08139 0.05607 0.00413

M02 - M33 0.06178 0.02639 0.04898 0.02700 0.01279

M02 - M34 0.06440 0.00354 0.06362 0.06067 0.00078

M02 - M35 0.06629 0.01159 0.06513 0.05296 0.00116

M02 - M36 0.03621 0.00597 0.03544 0.03185 0.00077

M02 - M37 0.06107 0.00988 0.06053 0.04848 0.00054

M02 - M38 0.07409 0.01699 0.07233 0.05374 0.00176

M02 - M39 0.02440 0.00140 0.02352 0.02316 0.00088

M02 - M40 0.03510 0.01011 0.03225 0.03074 0.00285

M02 - M41 0.02566 0.00467 0.02451 0.02596 0.00115

M02 - M42 0.05659 0.01786 0.05219 0.04053 0.00440

M02 - M43 0.09453 0.02131 0.09133 0.06669 0.00320

M02 - M44 0.03188 0.00853 0.03043 0.02695 0.00146

M02 - M45 0.07309 0.00828 0.07215 0.06482 0.00094

M02 - M46 0.02252 0.00207 0.02232 0.02137 0.00019

M02 - M47 0.06393 0.00643 0.06117 0.05764 0.00276

M02 - M48 0.07144 0.00583 0.06942 0.06836 0.00202

M02 - M49 0.07431 0.00754 0.07020 0.06975 0.00411

M02 - M50 0.05986 0.01242 0.05937 0.04935 0.00050

M02 - M51 0.03611 0.02567 0.03545 0.01224 0.00066

M02 - M52 0.04400 0.02369 0.04283 0.01932 0.00117

M02 - M53 0.05959 0.00659 0.05379 0.05100 0.00580

M02 - M54 0.04406 0.00553 0.04238 0.03652 0.00167

M02 - M55 0.05383 0.02803 0.05342 0.02354 0.00041

M02 - M56 0.05108 0.02467 0.05066 0.02252 0.00042

M02 - M57 0.03645 0.01834 0.03584 0.02152 0.00061

M02 - M58 0.02930 0.00264 0.02818 0.02530 0.00112

M02 - M59 0.05643 0.02046 0.05340 0.03270 0.00302

M02 - M60 0.03976 0.00472 0.03876 0.03133 0.00100

M02 - M61 0.05823 0.01476 0.05348 0.03945 0.00474

M02 - M62 0.03131 0.00323 0.03081 0.02795 0.00051

M02 - M63 0.03106 0.00332 0.03059 0.02762 0.00048

M02 - M64 0.05250 0.01949 0.04767 0.03199 0.00482

M02 - M65 0.05410 0.00446 0.04829 0.04854 0.00581

M02 - M66 0.06239 0.01512 0.06173 0.05033 0.00066

M02 - M67 0.06231 0.01233 0.06186 0.04977 0.00045

M02 - M68 0.04769 0.02202 0.04660 0.02265 0.00109

M02 - M69 0.04535 0.01075 0.04264 0.03316 0.00270

M02 - M70 0.04913 0.00660 0.04543 0.03991 0.00369

M02 - M71 0.05822 0.02068 0.05516 0.03489 0.00306

M02 - M72 0.05365 0.02225 0.05147 0.02319 0.00218

M02 - M73 0.02778 0.00435 0.02746 0.02732 0.00032

M02 - M74 0.02980 0.00109 0.02960 0.02839 0.00019

M02 - M75 0.02763 0.00675 0.02688 0.02368 0.00074

M02 - M76 0.07079 0.01395 0.07025 0.05804 0.00054

M02 - M77 0.04468 0.02303 0.04325 0.02560 0.00143

M02 - M78 0.02935 0.00058 0.02858 0.02837 0.00077

M02 - M79 0.03712 0.00304 0.03690 0.03160 0.00022

M02 - M80 0.04267 0.02292 0.04064 0.02061 0.00203

M02 - M81 0.03844 0.00982 0.03620 0.03232 0.00224

M02 - M82 0.04939 0.00656 0.04648 0.04462 0.00291

M02 - M83 0.06578 0.00972 0.06457 0.05535 0.00121

M02 - M84 0.04181 0.01586 0.04111 0.02787 0.00070

M02 - M85 0.03265 0.00505 0.03180 0.03075 0.00086

M02 - M86 0.07065 0.01556 0.06568 0.05586 0.00497

M02 - M87 0.03402 0.00512 0.03321 0.03079 0.00081

M02 - M88 0.08302 0.02437 0.07617 0.05265 0.00684

M02 - M89 0.09670 0.02219 0.09284 0.06801 0.00387

M02 - M90 0.05019 0.00245 0.04714 0.04671 0.00306

M02 - M91 0.03867 0.01075 0.03683 0.02701 0.00185

M02 - M92 0.06747 0.01839 0.05915 0.04645 0.00833

M02 - M93 0.04404 0.01011 0.04192 0.03018 0.00211

M02 - M94 0.04649 0.00391 0.04515 0.04003 0.00134

M02 - M95 0.07205 0.02813 0.07128 0.03812 0.00077

M02 - M96 0.07976 0.03593 0.07504 0.03666 0.00473

M02 - M97 0.07221 0.02653 0.06897 0.04241 0.00325

M03 - M04 0.07525 0.03520 0.04452 0.02800 0.03073

M03 - M05 0.06419 0.01015 0.05795 0.05310 0.00624

M03 - M06 0.06643 0.00580 0.06120 0.06362 0.00523

M03 - M07 0.07344 0.00508 0.07050 0.07067 0.00294

M03 - M08 0.06686 0.00541 0.06093 0.06207 0.00593

M03 - M09 0.09583 0.00751 0.08525 0.08293 0.01058

M03 - M10 0.21435 0.00206 0.20634 0.21163 0.00801

M03 - M11 0.13748 0.01863 0.12680 0.11215 0.01068

M03 - M12 0.06195 0.00039 0.05968 0.06067 0.00227

M03 - M13 0.12420 0.01055 0.12004 0.11490 0.00416

M03 - M14 0.13996 0.00935 0.13467 0.12797 0.00529

M03 - M15 0.04042 0.00025 0.03915 0.03885 0.00127

M03 - M16 0.11942 0.00121 0.11554 0.11742 0.00388

M03 - M17 0.04668 0.00313 0.04398 0.04389 0.00270

M03 - M18 0.05672 0.00616 0.05608 0.05515 0.00064

M03 - M19 0.08394 0.01710 0.07991 0.05781 0.00402

M03 - M20 0.04119 0.00382 0.04010 0.04225 0.00109

M03 - M21 0.05685 0.02511 0.05282 0.03465 0.00403

M03 - M22 0.05532 0.00773 0.05441 0.05077 0.00091

M03 - M23 0.07482 0.00771 0.07153 0.06915 0.00328

M03 - M24 0.07362 0.00559 0.06588 0.06738 0.00774

M03 - M25 0.11568 0.00320 0.10401 0.11361 0.01167

M03 - M26 0.04109 0.00140 0.03923 0.04177 0.00186

M03 - M27 0.06379 0.01665 0.06222 0.04613 0.00157

M03 - M28 0.06835 0.01321 0.06284 0.05652 0.00552

M03 - M29 0.04002 0.00458 0.03905 0.04047 0.00097

M03 - M30 0.06081 0.01630 0.05880 0.04390 0.00201

M03 - M31 0.06429 0.01898 0.06160 0.04410 0.00269

M03 - M32 0.09298 0.01887 0.08821 0.06794 0.00477

M03 - M33 0.06280 0.01929 0.05543 0.03676 0.00736

M03 - M34 0.07413 0.00067 0.07204 0.07218 0.00209

M03 - M35 0.08711 0.01585 0.08585 0.06485 0.00126

M03 - M36 0.05202 0.00631 0.05054 0.04415 0.00148

M03 - M37 0.08872 0.02044 0.08770 0.05922 0.00102

M03 - M38 0.08573 0.02231 0.08377 0.06158 0.00196

M03 - M39 0.04049 0.00095 0.03942 0.03908 0.00106

M03 - M40 0.04293 0.00957 0.04024 0.04153 0.00269

M03 - M41 0.04471 0.00451 0.04364 0.04196 0.00107

M03 - M42 0.05355 0.00527 0.05268 0.05247 0.00088

M03 - M43 0.10263 0.01961 0.09742 0.07717 0.00521

M03 - M44 0.03853 0.01155 0.03733 0.03487 0.00120

M03 - M45 0.07626 0.00034 0.07379 0.07563 0.00247

M03 - M46 0.03746 0.00185 0.03694 0.03783 0.00052

M03 - M47 0.07378 0.00543 0.07247 0.06924 0.00131

M03 - M48 0.08616 0.00528 0.08386 0.08146 0.00231

M03 - M49 0.08857 0.00622 0.08526 0.08262 0.00331

M03 - M50 0.06561 0.00975 0.06491 0.05845 0.00070

M03 - M51 0.04336 0.02526 0.04271 0.01977 0.00065

M03 - M52 0.05279 0.02417 0.05115 0.02737 0.00165

M03 - M53 0.06978 0.00720 0.06730 0.05953 0.00248

M03 - M54 0.05171 0.00680 0.05138 0.04421 0.00032

M03 - M55 0.06353 0.02591 0.06265 0.03221 0.00088

M03 - M56 0.06357 0.02651 0.06199 0.03250 0.00158

M03 - M57 0.04061 0.01356 0.03894 0.02971 0.00168

M03 - M58 0.04041 0.00159 0.03886 0.03941 0.00156

M03 - M59 0.05254 0.00681 0.05035 0.04294 0.00218

M03 - M60 0.05278 0.00380 0.05145 0.04342 0.00132

M03 - M61 0.05790 0.00751 0.05350 0.05120 0.00440

M03 - M62 0.04190 0.00172 0.04096 0.04212 0.00094

M03 - M63 0.04237 0.00215 0.04150 0.04151 0.00087

M03 - M64 0.07112 0.02764 0.06114 0.04176 0.00999

M03 - M65 0.06673 0.00283 0.06142 0.06196 0.00531

M03 - M66 0.06989 0.00789 0.06933 0.05902 0.00056

M03 - M67 0.07473 0.00897 0.07311 0.06129 0.00162

M03 - M68 0.05758 0.02580 0.05581 0.03187 0.00177

M03 - M69 0.04470 0.00566 0.04317 0.04238 0.00153

M03 - M70 0.05116 0.00498 0.04702 0.04969 0.00414

M03 - M71 0.07408 0.02635 0.07001 0.04340 0.00408

M03 - M72 0.05575 0.01889 0.05390 0.03175 0.00185

M03 - M73 0.04015 0.00404 0.03949 0.04142 0.00066

M03 - M74 0.04245 0.00122 0.04189 0.04196 0.00056

M03 - M75 0.04351 0.00881 0.04209 0.03956 0.00141

M03 - M76 0.07214 0.00494 0.07145 0.06944 0.00069

M03 - M77 0.05714 0.02487 0.05596 0.03432 0.00118

M03 - M78 0.03932 0.00116 0.03778 0.03826 0.00154

M03 - M79 0.05291 0.00652 0.05206 0.04186 0.00085

M03 - M80 0.05225 0.02477 0.05163 0.02770 0.00062

M03 - M81 0.05284 0.00852 0.05106 0.04485 0.00177

M03 - M82 0.06637 0.01334 0.05694 0.05306 0.00943

M03 - M83 0.07457 0.00943 0.07262 0.06497 0.00195

M03 - M84 0.04701 0.01209 0.04451 0.03611 0.00251

M03 - M85 0.04469 0.00555 0.04305 0.04507 0.00164

M03 - M86 0.08292 0.01724 0.07567 0.07023 0.00724

M03 - M87 0.04244 0.00560 0.04113 0.04485 0.00131

M03 - M88 0.08329 0.01999 0.07937 0.06024 0.00392

M03 - M89 0.10940 0.02415 0.10340 0.07603 0.00600

M03 - M90 0.06360 0.01029 0.05967 0.05364 0.00393

M03 - M91 0.04358 0.00824 0.04170 0.03705 0.00188

M03 - M92 0.07607 0.01912 0.06708 0.05468 0.00899

M03 - M93 0.04228 0.00077 0.04014 0.04252 0.00214

M03 - M94 0.06162 0.00464 0.06074 0.05664 0.00087

M03 - M95 0.08173 0.02399 0.08059 0.05094 0.00114

M03 - M96 0.07491 0.02051 0.07029 0.04825 0.00462

M03 - M97 0.07446 0.02049 0.06765 0.05093 0.00682

M04 - M05 0.05290 0.01317 0.04576 0.04270 0.00714

M04 - M06 0.04641 0.00287 0.04368 0.04555 0.00273

M04 - M07 0.04987 0.00908 0.04484 0.04437 0.00503

M04 - M08 0.06204 0.01113 0.05771 0.05500 0.00433

M04 - M09 0.07506 0.01191 0.07032 0.08078 0.00474

M04 - M10 0.25076 0.00411 0.24433 0.23571 0.00644

M04 - M11 0.13364 0.02156 0.12351 0.10885 0.01013

M04 - M12 0.05561 0.00602 0.05474 0.05179 0.00087

M04 - M13 0.11807 0.00454 0.11340 0.11734 0.00468

M04 - M14 0.14207 0.00790 0.14052 0.14004 0.00155

M04 - M15 0.01487 0.00024 0.01423 0.01386 0.00064

M04 - M16 0.12870 0.00262 0.12489 0.12693 0.00381

M04 - M17 0.03245 0.00299 0.02986 0.02984 0.00259

M04 - M18 0.04882 0.00981 0.04546 0.04523 0.00336

M04 - M19 0.06626 0.01923 0.06161 0.05184 0.00465

M04 - M20 0.03186 0.00380 0.03094 0.02087 0.00092

M04 - M21 0.05399 0.02186 0.05164 0.02346 0.00235

M04 - M22 0.04607 0.00893 0.04572 0.03967 0.00035

M04 - M23 0.07252 0.00741 0.06938 0.06540 0.00314

M04 - M24 0.07042 0.00529 0.05908 0.06031 0.01134

M04 - M25 0.12346 0.00624 0.10820 0.11008 0.01526

M04 - M26 0.01998 0.00173 0.01809 0.01738 0.00190

M04 - M27 0.05769 0.01519 0.05611 0.03767 0.00158

M04 - M28 0.06431 0.01204 0.05851 0.05089 0.00580

M04 - M29 0.03401 0.00510 0.03390 0.02500 0.00011

M04 - M30 0.06354 0.01874 0.06105 0.03570 0.00248

M04 - M31 0.07198 0.02031 0.06379 0.03553 0.00819

M04 - M32 0.08564 0.02046 0.07845 0.05776 0.00719

M04 - M33 0.04341 0.01970 0.04050 0.02651 0.00290

M04 - M34 0.06583 0.00349 0.06446 0.06664 0.00138

M04 - M35 0.06143 0.00742 0.06031 0.05450 0.00112

M04 - M36 0.03699 0.00451 0.03625 0.02985 0.00074

M04 - M37 0.05652 0.00738 0.05614 0.05172 0.00038

M04 - M38 0.09478 0.01976 0.09098 0.05878 0.00380

M04 - M39 0.01388 0.00120 0.01379 0.01219 0.00009

M04 - M40 0.03547 0.00721 0.03353 0.02906 0.00193

M04 - M41 0.03313 0.00404 0.03229 0.02149 0.00083

M04 - M42 0.05455 0.00739 0.05268 0.04363 0.00186

M04 - M43 0.09335 0.01944 0.08819 0.07980 0.00516

M04 - M44 0.03538 0.01033 0.03425 0.02406 0.00113

M04 - M45 0.07464 0.00508 0.07310 0.07195 0.00154

M04 - M46 0.01054 0.00036 0.01042 0.01022 0.00012

M04 - M47 0.06114 0.00286 0.06023 0.05771 0.00091

M04 - M48 0.07473 0.00413 0.07299 0.07067 0.00174

M04 - M49 0.07533 0.00393 0.07252 0.07203 0.00282

M04 - M50 0.05530 0.00484 0.05512 0.04942 0.00018

M04 - M51 0.05283 0.03229 0.05252 0.01666 0.00031

M04 - M52 0.05740 0.02709 0.05627 0.02706 0.00113

M04 - M53 0.07187 0.01136 0.06946 0.06358 0.00242

M04 - M54 0.05592 0.01421 0.05364 0.04359 0.00228

M04 - M55 0.05665 0.02645 0.05101 0.03254 0.00565

M04 - M56 0.04255 0.02127 0.04220 0.03078 0.00035

M04 - M57 0.02711 0.00964 0.02687 0.01886 0.00024

M04 - M58 0.01618 0.00126 0.01499 0.01461 0.00119

M04 - M59 0.03859 0.01318 0.03690 0.02535 0.00169

M04 - M60 0.02937 0.00319 0.02839 0.02277 0.00098

M04 - M61 0.04477 0.01199 0.03927 0.03342 0.00549

M04 - M62 0.02125 0.00226 0.02067 0.01701 0.00058

M04 - M63 0.02011 0.00192 0.01949 0.01638 0.00061

M04 - M64 0.06182 0.01884 0.05900 0.03077 0.00282

M04 - M65 0.04333 0.00094 0.03825 0.04298 0.00508

M04 - M66 0.06499 0.00982 0.06419 0.05156 0.00080

M04 - M67 0.05814 0.01046 0.05683 0.04667 0.00131

M04 - M68 0.04998 0.01938 0.04745 0.02030 0.00252

M04 - M69 0.03477 0.00864 0.03216 0.02857 0.00261

M04 - M70 0.03772 0.00378 0.03404 0.03599 0.00367

M04 - M71 0.06161 0.02387 0.05842 0.03688 0.00320

M04 - M72 0.04177 0.02345 0.04125 0.02008 0.00052

M04 - M73 0.03191 0.00387 0.03174 0.02247 0.00017

M04 - M74 0.01993 0.00130 0.01987 0.01899 0.00006

M04 - M75 0.03224 0.00583 0.03096 0.01888 0.00128

M04 - M76 0.08219 0.00989 0.08199 0.06919 0.00020

M04 - M77 0.06808 0.02204 0.06415 0.03377 0.00393

M04 - M78 0.02228 0.00178 0.02196 0.02031 0.00032

M04 - M79 0.04406 0.00239 0.04321 0.04076 0.00086

M04 - M80 0.05283 0.02240 0.05024 0.02768 0.00259

M04 - M81 0.04192 0.00674 0.03938 0.03190 0.00254

M04 - M82 0.05294 0.00759 0.04912 0.04915 0.00382

M04 - M83 0.06916 0.00925 0.06802 0.06468 0.00114

M04 - M84 0.03812 0.01358 0.03598 0.02636 0.00214

M04 - M85 0.04173 0.00593 0.04063 0.02692 0.00110

M04 - M86 0.09932 0.01780 0.09174 0.05784 0.00757

M04 - M87 0.04100 0.00603 0.04010 0.02695 0.00089

M04 - M88 0.09163 0.02362 0.08552 0.05762 0.00612

M04 - M89 0.08367 0.01887 0.07890 0.07644 0.00476

M04 - M90 0.04900 0.00472 0.04581 0.04451 0.00319

M04 - M91 0.02282 0.00518 0.02088 0.01584 0.00194

M04 - M92 0.08321 0.01982 0.07194 0.04692 0.01127

M04 - M93 0.02182 0.00213 0.02043 0.02165 0.00139

M04 - M94 0.03278 0.00344 0.03229 0.03398 0.00049

M04 - M95 0.06317 0.01839 0.06218 0.03697 0.00099

M04 - M96 0.05968 0.02174 0.05955 0.03719 0.00013

M04 - M97 0.07755 0.02713 0.07571 0.04559 0.00184

M05 - M06 0.09451 0.01032 0.08438 0.08242 0.01013

M05 - M07 0.10036 0.02957 0.07588 0.06548 0.02448

M05 - M08 0.18487 0.07549 0.07292 0.09398 0.11195

M05 - M09 0.12588 0.00980 0.11651 0.10758 0.00937

M05 - M10 0.25954 0.00849 0.24936 0.25410 0.01018

M05 - M11 0.14009 0.00643 0.12667 0.13584 0.01342

M05 - M12 0.09753 0.01734 0.06777 0.08916 0.02975

M05 - M13 0.14389 0.00320 0.13493 0.14341 0.00896

M05 - M14 0.17989 0.01631 0.17361 0.16062 0.00628

M05 - M15 0.05712 0.00122 0.04468 0.06091 0.01244

M05 - M16 0.16208 0.01458 0.13783 0.15043 0.02426

M05 - M17 0.08030 0.01124 0.06028 0.07347 0.02001

M05 - M18 0.07173 0.00625 0.06468 0.06656 0.00706

M05 - M19 0.08509 0.01399 0.06297 0.07118 0.02212

M05 - M20 0.05524 0.00001 0.04942 0.05508 0.00582

M05 - M21 0.06321 0.01075 0.05822 0.04969 0.00500

M05 - M22 0.09802 0.02071 0.07298 0.07743 0.02504

M05 - M23 0.12247 0.02453 0.08889 0.09985 0.03358

M05 - M24 0.10341 0.00431 0.08815 0.09948 0.01526

M05 - M25 0.15774 0.00619 0.13615 0.14727 0.02158

M05 - M26 0.06839 0.00159 0.05896 0.06298 0.00943

M05 - M27 0.06823 0.00589 0.06247 0.06010 0.00576

M05 - M28 0.08863 0.00830 0.07819 0.07618 0.01044

M05 - M29 0.05950 0.00191 0.05398 0.05723 0.00552

M05 - M30 0.06634 0.00288 0.06056 0.06377 0.00578

M05 - M31 0.06831 0.00514 0.06202 0.06359 0.00629

M05 - M32 0.09195 0.00478 0.08306 0.08474 0.00889

M05 - M33 0.05830 0.00355 0.05217 0.05110 0.00613

M05 - M34 0.11768 0.02509 0.07865 0.10025 0.03903

M05 - M35 0.08360 0.00707 0.07847 0.07555 0.00514

M05 - M36 0.05659 0.00293 0.05170 0.05560 0.00489

M05 - M37 0.07701 0.00823 0.07259 0.07071 0.00443

M05 - M38 0.09036 0.00720 0.08492 0.08541 0.00544

M05 - M39 0.05713 0.00035 0.04863 0.05764 0.00850

M05 - M40 0.06043 0.00301 0.05360 0.05273 0.00683

M05 - M41 0.05378 0.00022 0.04879 0.05402 0.00498

M05 - M42 0.06995 0.00520 0.05830 0.06476 0.01165

M05 - M43 0.10532 0.01078 0.08380 0.09507 0.02152

M05 - M44 0.05570 0.00420 0.04707 0.04886 0.00863

M05 - M45 0.11920 0.02357 0.07992 0.10511 0.03928

M05 - M46 0.05200 0.00095 0.04405 0.05462 0.00795

M05 - M47 0.10103 0.00506 0.09341 0.09858 0.00762

M05 - M48 0.11891 0.00703 0.11127 0.11369 0.00764

M05 - M49 0.12041 0.00623 0.10469 0.11271 0.01572

M05 - M50 0.09865 0.00886 0.09337 0.08761 0.00528

M05 - M51 0.04980 0.01486 0.04414 0.03591 0.00566

M05 - M52 0.04714 0.00574 0.04304 0.04423 0.00410

M05 - M53 0.11355 0.01264 0.09264 0.09754 0.02091

M05 - M54 0.09974 0.01647 0.08984 0.07962 0.00989

M05 - M55 0.06243 0.00794 0.05507 0.05122 0.00735

M05 - M56 0.05657 0.00597 0.05224 0.04955 0.00433

M05 - M57 0.05274 0.01074 0.04807 0.04231 0.00467

M05 - M58 0.06202 0.00123 0.05330 0.06041 0.00872

M05 - M59 0.07933 0.01525 0.07015 0.06674 0.00917

M05 - M60 0.07540 0.00463 0.06928 0.06796 0.00612

M05 - M61 0.08663 0.01350 0.07171 0.07478 0.01491

M05 - M62 0.06046 0.00130 0.05512 0.06026 0.00533

M05 - M63 0.06012 0.00112 0.05443 0.05982 0.00568

M05 - M64 0.06429 0.01042 0.05994 0.05397 0.00436

M05 - M65 0.09034 0.00475 0.08062 0.08360 0.00972

M05 - M66 0.10426 0.01189 0.09956 0.08340 0.00470

M05 - M67 0.09930 0.01267 0.09483 0.08656 0.00447

M05 - M68 0.05383 0.00505 0.04880 0.04680 0.00503

M05 - M69 0.08077 0.00983 0.07481 0.06917 0.00597

M05 - M70 0.08112 0.00249 0.06725 0.07609 0.01387

M05 - M71 0.06174 0.00275 0.05609 0.05934 0.00565

M05 - M72 0.04911 0.00302 0.04433 0.04351 0.00478

M05 - M73 0.05612 0.00172 0.04811 0.05628 0.00801

M05 - M74 0.06426 0.00122 0.05509 0.06302 0.00918

M05 - M75 0.05555 0.00417 0.04999 0.05316 0.00557

M05 - M76 0.10865 0.02401 0.08026 0.09147 0.02839

M05 - M77 0.05985 0.00235 0.05540 0.05757 0.00445

M05 - M78 0.06552 0.00264 0.06044 0.06138 0.00507

M05 - M79 0.07876 0.00320 0.06952 0.07492 0.00924

M05 - M80 0.05378 0.00165 0.05020 0.05022 0.00358

M05 - M81 0.05484 0.00394 0.04942 0.05484 0.00543

M05 - M82 0.07760 0.00565 0.06905 0.06954 0.00855

M05 - M83 0.07959 0.00446 0.07396 0.07956 0.00562

M05 - M84 0.05580 0.00658 0.05032 0.04871 0.00548

M05 - M85 0.05923 0.00366 0.05441 0.05487 0.00482

M05 - M86 0.09563 0.00650 0.08763 0.08611 0.00800

M05 - M87 0.05950 0.00395 0.05407 0.05532 0.00543

M05 - M88 0.09559 0.01466 0.09048 0.08038 0.00511

M05 - M89 0.09824 0.00395 0.09165 0.09669 0.00659

M05 - M90 0.08422 0.00254 0.07445 0.08479 0.00977

M05 - M91 0.06414 0.00594 0.05612 0.06022 0.00802

M05 - M92 0.07908 0.00422 0.06890 0.07573 0.01018

M05 - M93 0.07185 0.00292 0.05912 0.06425 0.01273

M05 - M94 0.07128 0.00596 0.06486 0.07143 0.00642

M05 - M95 0.06574 0.00590 0.06164 0.06231 0.00409

M05 - M96 0.06556 0.00658 0.06110 0.06195 0.00446

M05 - M97 0.06861 0.00794 0.06382 0.06664 0.00479

M06 - M07 0.07999 0.00846 0.07562 0.06728 0.00437

M06 - M08 0.10449 0.01366 0.09989 0.09159 0.00460

M06 - M09 0.11246 0.00071 0.10500 0.11113 0.00746

M06 - M10 0.27823 0.00284 0.26899 0.27381 0.00925

M06 - M11 0.15128 0.00155 0.13703 0.14952 0.01424

M06 - M12 0.09471 0.00229 0.09072 0.09186 0.00399

M06 - M13 0.16437 0.00809 0.14404 0.15966 0.02033

M06 - M14 0.17001 0.00353 0.16502 0.16938 0.00499

M06 - M15 0.06945 0.00030 0.06315 0.06904 0.00630

M06 - M16 0.16974 0.00791 0.16137 0.16438 0.00837

M06 - M17 0.07743 0.00369 0.07167 0.07114 0.00576

M06 - M18 0.08205 0.00618 0.07577 0.07766 0.00628

M06 - M19 0.08525 0.00443 0.08192 0.08102 0.00333

M06 - M20 0.06551 0.00002 0.06189 0.06552 0.00362

M06 - M21 0.05812 0.00459 0.05495 0.05424 0.00317

M06 - M22 0.08849 0.00557 0.08444 0.07909 0.00406

M06 - M23 0.11053 0.00622 0.10160 0.10408 0.00892

M06 - M24 0.10770 0.00389 0.09142 0.10447 0.01628

M06 - M25 0.16704 0.00649 0.13717 0.16078 0.02987

M06 - M26 0.07713 0.00104 0.06809 0.07416 0.00904

M06 - M27 0.06497 0.00330 0.06147 0.06575 0.00349

M06 - M28 0.08152 0.00353 0.07181 0.08013 0.00971

M06 - M29 0.06123 0.00060 0.05835 0.06161 0.00288

M06 - M30 0.06702 0.00175 0.06156 0.06665 0.00546

M06 - M31 0.06673 0.00174 0.05983 0.06469 0.00691

M06 - M32 0.09690 0.00114 0.08535 0.09588 0.01154

M06 - M33 0.05935 0.00534 0.05674 0.05641 0.00260

M06 - M34 0.10800 0.00235 0.10349 0.10636 0.00451

M06 - M35 0.09041 0.00292 0.08706 0.08942 0.00335

M06 - M36 0.06769 0.00214 0.06502 0.06600 0.00267

M06 - M37 0.08834 0.00461 0.08398 0.08284 0.00437

M06 - M38 0.08030 0.00402 0.07694 0.08138 0.00336

M06 - M39 0.06874 0.00048 0.06332 0.06793 0.00542

M06 - M40 0.06706 0.00385 0.06185 0.06076 0.00521

M06 - M41 0.06275 0.00017 0.05956 0.06298 0.00318

M06 - M42 0.09007 0.01139 0.08702 0.07589 0.00305

M06 - M43 0.11183 0.00669 0.10635 0.10090 0.00548

M06 - M44 0.05643 0.00308 0.05262 0.05358 0.00381

M06 - M45 0.11067 0.00278 0.10565 0.10944 0.00502

M06 - M46 0.06404 0.00229 0.05483 0.06510 0.00921

M06 - M47 0.12274 0.00760 0.11888 0.11236 0.00386

M06 - M48 0.14330 0.00666 0.13873 0.13000 0.00457

M06 - M49 0.14254 0.00942 0.13686 0.12879 0.00567

M06 - M50 0.10293 0.00622 0.10028 0.09294 0.00265

M06 - M51 0.04671 0.00487 0.04374 0.03961 0.00297

M06 - M52 0.05388 0.00587 0.05122 0.04681 0.00266

M06 - M53 0.10612 0.00606 0.09848 0.10264 0.00763

M06 - M54 0.08502 0.00236 0.07998 0.08247 0.00505

M06 - M55 0.05683 0.00445 0.05161 0.05365 0.00523

M06 - M56 0.06272 0.00523 0.05842 0.05810 0.00430

M06 - M57 0.05373 0.00814 0.05034 0.04707 0.00340

M06 - M58 0.07186 0.00078 0.06510 0.06993 0.00676

M06 - M59 0.07785 0.00569 0.07219 0.07377 0.00567

M06 - M60 0.08160 0.00452 0.07726 0.07303 0.00434

M06 - M61 0.08876 0.00493 0.07716 0.08484 0.01159

M06 - M62 0.07518 0.00237 0.06630 0.07299 0.00888

M06 - M63 0.07471 0.00209 0.06712 0.07225 0.00759

M06 - M64 0.07314 0.01072 0.06912 0.06110 0.00402

M06 - M65 0.10166 0.00362 0.08694 0.09849 0.01472

M06 - M66 0.09643 0.00554 0.09074 0.09336 0.00569

M06 - M67 0.09998 0.00627 0.09588 0.09673 0.00410

M06 - M68 0.05591 0.00621 0.05163 0.04921 0.00428

M06 - M69 0.07346 0.00365 0.06724 0.06892 0.00622

M06 - M70 0.08554 0.00359 0.07279 0.08110 0.01274

M06 - M71 0.06940 0.00452 0.06340 0.06291 0.00599

M06 - M72 0.05949 0.00726 0.05608 0.04662 0.00341

M06 - M73 0.06777 0.00126 0.06321 0.06495 0.00457

M06 - M74 0.07086 0.00149 0.06511 0.07229 0.00575

M06 - M75 0.06201 0.00287 0.05614 0.05921 0.00588

M06 - M76 0.09591 0.00272 0.09292 0.09366 0.00299

M06 - M77 0.05687 0.00308 0.05333 0.05631 0.00353

M06 - M78 0.06871 0.00165 0.06591 0.06368 0.00279

M06 - M79 0.08869 0.00777 0.08518 0.08123 0.00351

M06 - M80 0.05103 0.00411 0.04794 0.04983 0.00310

M06 - M81 0.06614 0.00150 0.06233 0.06420 0.00381

M06 - M82 0.07831 0.00277 0.07321 0.07452 0.00510

M06 - M83 0.09965 0.00767 0.09454 0.09233 0.00511

M06 - M84 0.06476 0.00667 0.06020 0.05602 0.00457

M06 - M85 0.07233 0.00343 0.06812 0.06664 0.00421

M06 - M86 0.10809 0.00481 0.10159 0.10099 0.00650

M06 - M87 0.07326 0.00351 0.06919 0.06595 0.00407

M06 - M88 0.08346 0.00575 0.07706 0.07805 0.00641

M06 - M89 0.09711 0.00160 0.09445 0.09447 0.00265

M06 - M90 0.09672 0.00468 0.08877 0.08591 0.00794

M06 - M91 0.07923 0.00878 0.07502 0.06393 0.00421

M06 - M92 0.07980 0.00382 0.07273 0.07876 0.00707

M06 - M93 0.07775 0.00384 0.07235 0.07099 0.00540

M06 - M94 0.09411 0.01128 0.08783 0.08302 0.00629

M06 - M95 0.07722 0.00174 0.07341 0.07807 0.00381

M06 - M96 0.07840 0.00476 0.07490 0.07150 0.00350

M06 - M97 0.07782 0.00405 0.07467 0.07409 0.00315

M07 - M08 0.11680 0.04010 0.08749 0.07361 0.02931

M07 - M09 0.10688 0.02021 0.10233 0.10053 0.00455

M07 - M10 0.27258 0.00286 0.26648 0.27173 0.00610

M07 - M11 0.17033 0.00649 0.15457 0.17638 0.01576

M07 - M12 0.08270 0.00261 0.07965 0.08662 0.00305

M07 - M13 0.14954 0.00318 0.14397 0.14130 0.00557

M07 - M14 0.16965 0.00239 0.16853 0.16794 0.00112

M07 - M15 0.04949 0.00082 0.04733 0.04980 0.00216

M07 - M16 0.15305 0.01097 0.14850 0.14458 0.00456

M07 - M17 0.06769 0.00376 0.06196 0.06120 0.00573

M07 - M18 0.08276 0.02217 0.08221 0.06537 0.00055

M07 - M19 0.08005 0.00401 0.07544 0.07854 0.00462

M07 - M20 0.06493 0.01923 0.06003 0.06561 0.00490

M07 - M21 0.07949 0.00643 0.07832 0.07254 0.00117

M07 - M22 0.06450 0.00374 0.06169 0.06218 0.00282

M07 - M23 0.10130 0.00469 0.08888 0.09905 0.01242

M07 - M24 0.08613 0.00459 0.07767 0.08025 0.00846

M07 - M25 0.14137 0.00371 0.12712 0.13352 0.01425

M07 - M26 0.05348 0.00187 0.04894 0.05246 0.00455

M07 - M27 0.07633 0.02580 0.07576 0.05432 0.00057

M07 - M28 0.07779 0.02480 0.07388 0.05906 0.00390

M07 - M29 0.06094 0.02027 0.05636 0.06121 0.00458

M07 - M30 0.08744 0.00192 0.08390 0.08568 0.00354

M07 - M31 0.08738 0.00132 0.08197 0.08652 0.00541

M07 - M32 0.11829 0.00317 0.11038 0.10964 0.00791

M07 - M33 0.07163 0.00754 0.07107 0.05841 0.00056

M07 - M34 0.09780 0.00269 0.09484 0.10064 0.00296

M07 - M35 0.11454 0.02135 0.11261 0.09077 0.00193

M07 - M36 0.06814 0.02001 0.06611 0.05933 0.00203

M07 - M37 0.09326 0.02112 0.09280 0.07602 0.00046

M07 - M38 0.12148 0.00153 0.11720 0.11973 0.00428

M07 - M39 0.04627 0.00018 0.04567 0.04747 0.00060

M07 - M40 0.07225 0.02362 0.06349 0.05649 0.00875

M07 - M41 0.06576 0.01938 0.06119 0.06480 0.00456

M07 - M42 0.11202 0.02468 0.11041 0.07505 0.00161

M07 - M43 0.11596 0.00657 0.10920 0.11453 0.00675

M07 - M44 0.08205 0.02351 0.08148 0.05171 0.00057

M07 - M45 0.10210 0.00242 0.09752 0.10587 0.00458

M07 - M46 0.04411 0.00033 0.04376 0.04396 0.00035

M07 - M47 0.07673 0.00336 0.07490 0.07124 0.00183

M07 - M48 0.08520 0.00246 0.08313 0.08406 0.00207

M07 - M49 0.08578 0.00229 0.08155 0.08605 0.00423

M07 - M50 0.07270 0.00528 0.07233 0.06930 0.00036

M07 - M51 0.09262 0.01030 0.08562 0.08482 0.00700

M07 - M52 0.08000 0.00210 0.07503 0.07960 0.00498

M07 - M53 0.10369 0.00432 0.09762 0.09948 0.00606

M07 - M54 0.08371 0.00726 0.08038 0.07516 0.00332

M07 - M55 0.08852 0.00896 0.07601 0.07930 0.01250

M07 - M56 0.06382 0.00580 0.05891 0.05755 0.00491

M07 - M57 0.05640 0.02300 0.05580 0.03299 0.00060

M07 - M58 0.04695 0.00094 0.04537 0.04414 0.00158

M07 - M59 0.05862 0.01075 0.05637 0.04762 0.00226

M07 - M60 0.05291 0.00135 0.05210 0.05018 0.00081

M07 - M61 0.07631 0.01320 0.06748 0.05785 0.00884

M07 - M62 0.04840 0.00049 0.04767 0.04758 0.00073

M07 - M63 0.04803 0.00040 0.04727 0.04761 0.00076

M07 - M64 0.07779 0.00485 0.07574 0.07640 0.00204

M07 - M65 0.07041 0.00242 0.06541 0.06676 0.00500

M07 - M66 0.08814 0.01194 0.08737 0.07090 0.00077

M07 - M67 0.07573 0.01074 0.07348 0.06655 0.00225

M07 - M68 0.06666 0.00257 0.06407 0.06283 0.00259

M07 - M69 0.05765 0.00404 0.05608 0.05128 0.00157

M07 - M70 0.06380 0.00395 0.05670 0.05860 0.00710

M07 - M71 0.08342 0.00288 0.07934 0.08003 0.00408

M07 - M72 0.05705 0.00269 0.05658 0.05103 0.00047

M07 - M73 0.09975 0.01941 0.09879 0.06303 0.00096

M07 - M74 0.05052 0.00172 0.04951 0.04970 0.00102

M07 - M75 0.09178 0.01992 0.08969 0.05607 0.00209

M07 - M76 0.12748 0.02163 0.12701 0.09938 0.00047

M07 - M77 0.13276 0.00147 0.12261 0.12762 0.01015

M07 - M78 0.04694 0.00101 0.04642 0.04734 0.00052

M07 - M79 0.07084 0.00304 0.06503 0.06830 0.00582

M07 - M80 0.10352 0.00542 0.09836 0.09763 0.00516

M07 - M81 0.09577 0.02061 0.08670 0.06083 0.00907

M07 - M82 0.08429 0.02216 0.08275 0.06154 0.00155

M07 - M83 0.10625 0.02290 0.10401 0.08369 0.00224

M07 - M84 0.06186 0.02169 0.06037 0.04008 0.00149

M07 - M85 0.10557 0.02129 0.10318 0.06356 0.00239

M07 - M86 0.12768 0.00353 0.11954 0.12226 0.00814

M07 - M87 0.11111 0.02151 0.10889 0.06799 0.00222

M07 - M88 0.11116 0.00203 0.10775 0.10694 0.00341

M07 - M89 0.12228 0.00302 0.11928 0.12077 0.00301

M07 - M90 0.06449 0.00258 0.06183 0.06223 0.00266

M07 - M91 0.04317 0.00050 0.04177 0.04330 0.00140

M07 - M92 0.10284 0.00186 0.09439 0.09900 0.00844

M07 - M93 0.05790 0.00176 0.05640 0.05330 0.00150

M07 - M94 0.06122 0.00372 0.05967 0.05487 0.00155

M07 - M95 0.08592 0.00438 0.08456 0.08370 0.00136

M07 - M96 0.09619 0.01116 0.09369 0.08341 0.00250

M07 - M97 0.09268 0.00509 0.09066 0.09000 0.00202

M08 - M09 0.13995 0.01323 0.13198 0.11766 0.00797

M08 - M10 0.26105 0.00845 0.25356 0.25318 0.00749

M08 - M11 0.14539 0.00600 0.13296 0.14106 0.01243

M08 - M12 0.12008 0.02532 0.08929 0.10213 0.03079

M08 - M13 0.15444 0.00137 0.14860 0.15168 0.00585

M08 - M14 0.17370 0.00841 0.16826 0.16632 0.00543

M08 - M15 0.07330 0.00173 0.06478 0.07763 0.00852

M08 - M16 0.18129 0.02146 0.15039 0.15692 0.03091

M08 - M17 0.09377 0.01557 0.07431 0.08478 0.01946

M08 - M18 0.08852 0.00981 0.08051 0.07646 0.00801

M08 - M19 0.10912 0.02349 0.08240 0.08141 0.02672

M08 - M20 0.07284 0.00001 0.07011 0.07277 0.00273

M08 - M21 0.07165 0.01268 0.06648 0.05752 0.00517

M08 - M22 0.12039 0.02874 0.08548 0.09082 0.03491

M08 - M23 0.14218 0.03156 0.10262 0.10834 0.03956

M08 - M24 0.11231 0.00697 0.10304 0.10704 0.00927

M08 - M25 0.15137 0.00365 0.13745 0.15037 0.01392

M08 - M26 0.08083 0.00107 0.07532 0.07793 0.00551

M08 - M27 0.07541 0.00550 0.07243 0.06854 0.00298

M08 - M28 0.09268 0.00527 0.08482 0.08753 0.00786

M08 - M29 0.07631 0.00144 0.07404 0.07431 0.00227

M08 - M30 0.08271 0.00180 0.07939 0.07599 0.00332

M08 - M31 0.08499 0.00836 0.08086 0.07376 0.00414

M08 - M32 0.10587 0.00934 0.09949 0.09213 0.00638

M08 - M33 0.06761 0.00511 0.06521 0.06001 0.00240

M08 - M34 0.13551 0.02560 0.10055 0.11292 0.03496

M08 - M35 0.08760 0.00688 0.08430 0.08058 0.00330

M08 - M36 0.07115 0.00253 0.06877 0.06826 0.00238

M08 - M37 0.08160 0.00515 0.07916 0.07782 0.00244

M08 - M38 0.09150 0.00328 0.08871 0.09030 0.00279

M08 - M39 0.06992 0.00100 0.06425 0.07461 0.00567

M08 - M40 0.06945 0.00389 0.06560 0.06495 0.00386

M08 - M41 0.07122 0.00023 0.06893 0.07143 0.00230

M08 - M42 0.08491 0.00915 0.07652 0.07687 0.00839

M08 - M43 0.13489 0.02510 0.11421 0.10490 0.02068

M08 - M44 0.06881 0.01009 0.06008 0.05691 0.00874

M08 - M45 0.14610 0.03224 0.10585 0.11745 0.04025

M08 - M46 0.07094 0.00064 0.06771 0.07358 0.00323

M08 - M47 0.11345 0.00477 0.10926 0.10898 0.00419

M08 - M48 0.13080 0.00498 0.12531 0.12467 0.00549

M08 - M49 0.13369 0.00548 0.11966 0.12320 0.01404

M08 - M50 0.11627 0.01634 0.11250 0.09623 0.00377

M08 - M51 0.05588 0.00983 0.05173 0.04677 0.00415

M08 - M52 0.05676 0.00330 0.05478 0.05510 0.00198

M08 - M53 0.11277 0.00576 0.10041 0.10509 0.01236

M08 - M54 0.09810 0.00818 0.09591 0.08901 0.00219

M08 - M55 0.06684 0.00871 0.06448 0.05814 0.00235

M08 - M56 0.06913 0.00741 0.06658 0.05850 0.00254

M08 - M57 0.05675 0.00610 0.05410 0.05319 0.00265

M08 - M58 0.07746 0.00090 0.07175 0.07684 0.00570

M08 - M59 0.09031 0.01401 0.08488 0.07699 0.00543

M08 - M60 0.09395 0.00866 0.08895 0.08039 0.00501

M08 - M61 0.09333 0.01075 0.08325 0.08524 0.01007

M08 - M62 0.07948 0.00298 0.07593 0.07653 0.00355

M08 - M63 0.07916 0.00271 0.07550 0.07622 0.00366

M08 - M64 0.07343 0.00730 0.07014 0.06554 0.00329

M08 - M65 0.09704 0.00461 0.09030 0.09607 0.00674

M08 - M66 0.10910 0.01324 0.10712 0.09432 0.00198

M08 - M67 0.10456 0.01034 0.10174 0.09523 0.00282

M08 - M68 0.06529 0.00692 0.06281 0.05666 0.00247

M08 - M69 0.10200 0.01541 0.09841 0.08289 0.00359

M08 - M70 0.08668 0.00204 0.07781 0.08421 0.00888

M08 - M71 0.07111 0.00295 0.06636 0.06707 0.00474

M08 - M72 0.05923 0.00349 0.05696 0.05434 0.00227

M08 - M73 0.06924 0.00104 0.06539 0.07212 0.00385

M08 - M74 0.07917 0.00175 0.07310 0.07997 0.00607

M08 - M75 0.06371 0.00090 0.06048 0.06708 0.00322

M08 - M76 0.13347 0.03199 0.10119 0.10225 0.03228

M08 - M77 0.07430 0.00221 0.07138 0.07151 0.00292

M08 - M78 0.09068 0.00163 0.08740 0.08352 0.00327

M08 - M79 0.09212 0.00334 0.08526 0.08839 0.00686

M08 - M80 0.06602 0.00457 0.06178 0.06127 0.00424

M08 - M81 0.07216 0.00306 0.06920 0.07066 0.00297

M08 - M82 0.08493 0.00732 0.07735 0.07914 0.00758

M08 - M83 0.08910 0.00243 0.08599 0.08822 0.00311

M08 - M84 0.06744 0.00852 0.06200 0.06003 0.00544

M08 - M85 0.07583 0.00225 0.07310 0.07205 0.00272

M08 - M86 0.10920 0.00495 0.10324 0.09934 0.00596

M08 - M87 0.07307 0.00256 0.07020 0.07047 0.00287

M08 - M88 0.10550 0.01832 0.10229 0.08857 0.00322

M08 - M89 0.10430 0.00672 0.10186 0.10162 0.00244

M08 - M90 0.09511 0.00415 0.08836 0.09250 0.00675

M08 - M91 0.07736 0.00441 0.07251 0.07201 0.00485

M08 - M92 0.09525 0.00536 0.08868 0.08528 0.00657

M08 - M93 0.07707 0.00190 0.07160 0.07636 0.00547

M08 - M94 0.08618 0.00431 0.08208 0.08485 0.00411

M08 - M95 0.07648 0.00801 0.07457 0.07034 0.00191

M08 - M96 0.07517 0.01027 0.07249 0.06787 0.00269

M08 - M97 0.08447 0.00737 0.08096 0.07871 0.00351

M09 - M10 0.30965 0.00170 0.27823 0.31407 0.03141

M09 - M11 0.18950 0.00731 0.17592 0.18097 0.01358

M09 - M12 0.18738 0.01598 0.17696 0.14343 0.01042

M09 - M13 0.18556 0.00511 0.17592 0.18597 0.00964

M09 - M14 0.20489 0.00706 0.19497 0.21123 0.00993

M09 - M15 0.11391 0.00042 0.10568 0.11245 0.00823

M09 - M16 0.20497 0.00947 0.18981 0.18697 0.01516

M09 - M17 0.12042 0.00897 0.10786 0.09865 0.01256

M09 - M18 0.11004 0.01265 0.10359 0.10097 0.00645

M09 - M19 0.13644 0.01504 0.12838 0.11260 0.00806

M09 - M20 0.10450 0.00380 0.09808 0.11402 0.00642

M09 - M21 0.08882 0.00833 0.08446 0.08131 0.00437

M09 - M22 0.15260 0.01784 0.14019 0.11225 0.01241

M09 - M23 0.18835 0.01781 0.17866 0.14883 0.00969

M09 - M24 0.11780 0.00192 0.10563 0.11416 0.01216

M09 - M25 0.15509 0.00205 0.13934 0.15536 0.01575

M09 - M26 0.10567 0.00041 0.09891 0.10704 0.00676

M09 - M27 0.11533 0.01714 0.10995 0.09623 0.00538

M09 - M28 0.12722 0.01721 0.11800 0.11444 0.00923

M09 - M29 0.09351 0.00480 0.08855 0.10631 0.00496

M09 - M30 0.10491 0.00524 0.09839 0.09662 0.00651

M09 - M31 0.10713 0.00978 0.09963 0.09554 0.00750

M09 - M32 0.11840 0.01098 0.11009 0.11123 0.00831

M09 - M33 0.08616 0.00626 0.08026 0.08830 0.00589

M09 - M34 0.20119 0.01329 0.19091 0.15817 0.01028

M09 - M35 0.14910 0.01991 0.14391 0.11932 0.00519

M09 - M36 0.09324 0.00586 0.08645 0.09351 0.00678

M09 - M37 0.13504 0.01876 0.12940 0.11027 0.00565

M09 - M38 0.14085 0.00632 0.13558 0.12930 0.00527

M09 - M39 0.11942 0.00089 0.10945 0.11366 0.00997

M09 - M40 0.09090 0.00463 0.08511 0.09338 0.00578

M09 - M41 0.09936 0.00415 0.09310 0.10973 0.00625

M09 - M42 0.17214 0.02047 0.16161 0.12351 0.01054

M09 - M43 0.16431 0.01347 0.15724 0.14731 0.00707

M09 - M44 0.12499 0.02118 0.11503 0.08668 0.00996

M09 - M45 0.21221 0.01849 0.20184 0.16245 0.01037

M09 - M46 0.11173 0.00099 0.10419 0.11034 0.00753

M09 - M47 0.10917 0.00109 0.10399 0.10884 0.00518

M09 - M48 0.12247 0.00090 0.11648 0.12575 0.00599

M09 - M49 0.12568 0.00159 0.11840 0.12584 0.00729

M09 - M50 0.12191 0.01301 0.11613 0.10711 0.00578

M09 - M51 0.08500 0.02118 0.08061 0.05682 0.00439

M09 - M52 0.08677 0.02064 0.08103 0.06626 0.00574

M09 - M53 0.12150 0.01528 0.11592 0.10395 0.00558

M09 - M54 0.09182 0.01212 0.08889 0.08075 0.00293

M09 - M55 0.08946 0.02264 0.08438 0.07203 0.00508

M09 - M56 0.08604 0.02261 0.08157 0.07751 0.00447

M09 - M57 0.08807 0.01686 0.08305 0.07114 0.00503

M09 - M58 0.10507 0.00069 0.09762 0.10073 0.00745

M09 - M59 0.09728 0.00553 0.09081 0.08793 0.00647

M09 - M60 0.11214 0.00481 0.10549 0.10324 0.00665

M09 - M61 0.10627 0.00419 0.09616 0.09549 0.01011

M09 - M62 0.10538 0.00032 0.09791 0.10284 0.00746

M09 - M63 0.10448 0.00011 0.09723 0.10347 0.00725

M09 - M64 0.09805 0.00592 0.09309 0.08589 0.00496

M09 - M65 0.11203 0.00169 0.10301 0.11304 0.00902

M09 - M66 0.11594 0.00356 0.11075 0.11575 0.00519

M09 - M67 0.10191 0.00276 0.09610 0.09892 0.00581

M09 - M68 0.07748 0.00494 0.07103 0.07139 0.00645

M09 - M69 0.09726 0.01193 0.09200 0.09230 0.00527

M09 - M70 0.09586 0.00223 0.08759 0.09241 0.00827

M09 - M71 0.09834 0.00520 0.09196 0.09308 0.00637

M09 - M72 0.08283 0.01104 0.07784 0.07750 0.00499

M09 - M73 0.14860 0.01530 0.13658 0.10822 0.01202

M09 - M74 0.11156 0.00101 0.10286 0.11203 0.00870

M09 - M75 0.14665 0.01707 0.12896 0.10006 0.01769

M09 - M76 0.22155 0.03350 0.20638 0.14380 0.01517

M09 - M77 0.10926 0.02317 0.10193 0.07843 0.00733

M09 - M78 0.11116 0.00164 0.10424 0.11335 0.00692

M09 - M79 0.10398 0.00147 0.09629 0.09635 0.00769

M09 - M80 0.09596 0.01986 0.09157 0.07009 0.00438

M09 - M81 0.14100 0.01553 0.12581 0.10600 0.01519

M09 - M82 0.12636 0.01900 0.11635 0.10553 0.01001

M09 - M83 0.14513 0.01644 0.13975 0.12694 0.00538

M09 - M84 0.09972 0.02163 0.09068 0.07747 0.00904

M09 - M85 0.15403 0.01694 0.13734 0.10841 0.01669

M09 - M86 0.15311 0.00617 0.13715 0.13176 0.01596

M09 - M87 0.15693 0.01707 0.14040 0.11073 0.01653

M09 - M88 0.13605 0.01938 0.12885 0.10879 0.00720

M09 - M89 0.14485 0.00486 0.13567 0.14607 0.00917

M09 - M90 0.10342 0.00162 0.09457 0.10307 0.00885

M09 - M91 0.09366 0.00208 0.08552 0.09312 0.00814

M09 - M92 0.12762 0.00552 0.11647 0.11631 0.01115

M09 - M93 0.11691 0.00132 0.10576 0.10744 0.01115

M09 - M94 0.10760 0.00182 0.09691 0.10240 0.01070

M09 - M95 0.10432 0.00770 0.09965 0.09367 0.00467

M09 - M96 0.10318 0.00918 0.09690 0.09298 0.00628

M09 - M97 0.10599 0.00928 0.10149 0.09982 0.00451

M10 - M11 0.35696 0.00576 0.32317 0.34774 0.03379

M10 - M12 0.34702 0.00826 0.34103 0.34111 0.00599

M10 - M13 0.33971 0.00215 0.33051 0.33942 0.00921

M10 - M14 0.36556 0.00536 0.33000 0.35953 0.03556

M10 - M15 0.34442 0.00181 0.33643 0.33977 0.00798

M10 - M16 0.34122 0.00156 0.32109 0.34195 0.02014

M10 - M17 0.28540 0.00878 0.27524 0.27926 0.01016

M10 - M18 0.22991 0.00357 0.21811 0.22854 0.01181

M10 - M19 0.27356 0.00873 0.26728 0.26840 0.00628

M10 - M20 0.33914 0.00000 0.33062 0.33938 0.00852

M10 - M21 0.21136 0.00303 0.20347 0.21295 0.00789

M10 - M22 0.27523 0.00982 0.26924 0.27368 0.00598

M10 - M23 0.31893 0.00996 0.30789 0.31664 0.01104

M10 - M24 0.25687 0.00316 0.23772 0.25295 0.01915

M10 - M25 0.32243 0.00410 0.29393 0.31509 0.02850

M10 - M26 0.31541 0.00025 0.30389 0.31847 0.01151

M10 - M27 0.21763 0.00205 0.21114 0.21916 0.00649

M10 - M28 0.25620 0.00115 0.24314 0.25211 0.01306

M10 - M29 0.31172 0.00182 0.30242 0.30575 0.00930

M10 - M30 0.24684 0.00230 0.23948 0.24442 0.00735

M10 - M31 0.24416 0.00221 0.23485 0.23805 0.00931

M10 - M32 0.26562 0.00281 0.25152 0.25952 0.01410

M10 - M33 0.21615 0.00224 0.21031 0.21391 0.00585

M10 - M34 0.36146 0.01036 0.35448 0.35523 0.00698

M10 - M35 0.25611 0.00209 0.24351 0.25364 0.01260

M10 - M36 0.25747 0.00079 0.23810 0.25434 0.01937

M10 - M37 0.23991 0.00162 0.22579 0.23952 0.01412

M10 - M38 0.25698 0.00243 0.24066 0.25368 0.01632

M10 - M39 0.33607 0.00085 0.32802 0.33221 0.00805

M10 - M40 0.23754 0.00120 0.22782 0.24001 0.00972

M10 - M41 0.32383 0.00004 0.31513 0.32487 0.00870

M10 - M42 0.28753 0.00491 0.28026 0.28923 0.00727

M10 - M43 0.26998 0.00393 0.25891 0.27494 0.01107

M10 - M44 0.21791 0.00216 0.21190 0.21822 0.00601

M10 - M45 0.36409 0.01272 0.35636 0.35606 0.00773

M10 - M46 0.32984 0.00028 0.31993 0.33098 0.00991

M10 - M47 0.26710 0.00121 0.26012 0.26923 0.00698

M10 - M48 0.30015 0.00129 0.29228 0.30011 0.00787

M10 - M49 0.28717 0.00285 0.27864 0.28904 0.00853

M10 - M50 0.24594 0.00310 0.23942 0.24260 0.00652

M10 - M51 0.20325 0.00658 0.19664 0.19136 0.00661

M10 - M52 0.19842 0.00178 0.19151 0.19454 0.00690

M10 - M53 0.25716 0.00171 0.23792 0.25610 0.01924

M10 - M54 0.23235 0.00236 0.22661 0.22995 0.00574

M10 - M55 0.21304 0.00255 0.20600 0.20936 0.00705

M10 - M56 0.23332 0.00196 0.22757 0.23339 0.00574

M10 - M57 0.19583 0.00342 0.18981 0.19341 0.00602

M10 - M58 0.29174 0.00084 0.28268 0.29630 0.00906

M10 - M59 0.23425 0.00469 0.22577 0.23075 0.00848

M10 - M60 0.26335 0.00221 0.25649 0.26786 0.00686

M10 - M61 0.25091 0.00450 0.23780 0.24617 0.01311

M10 - M62 0.29500 0.00094 0.28559 0.29057 0.00941

M10 - M63 0.29784 0.00088 0.28828 0.29301 0.00957

M10 - M64 0.22605 0.00101 0.22018 0.22451 0.00588

M10 - M65 0.27517 0.00066 0.26047 0.27876 0.01470

M10 - M66 0.25924 0.00245 0.25136 0.25704 0.00788

M10 - M67 0.24984 0.00433 0.24391 0.24385 0.00593

M10 - M68 0.20929 0.00299 0.20272 0.20237 0.00658

M10 - M69 0.24092 0.00169 0.22986 0.24534 0.01106

M10 - M70 0.23805 0.00244 0.22544 0.23557 0.01261

M10 - M71 0.21496 0.00179 0.20607 0.21423 0.00889

M10 - M72 0.21961 0.00132 0.21338 0.21798 0.00623

M10 - M73 0.32608 0.00122 0.31813 0.32517 0.00795

M10 - M74 0.31585 0.00023 0.30453 0.31765 0.01132

M10 - M75 0.28589 0.00030 0.27753 0.28838 0.00835

M10 - M76 0.31907 0.00804 0.31231 0.31509 0.00676

M10 - M77 0.24530 0.00059 0.23838 0.24757 0.00693

M10 - M78 0.29611 0.00070 0.28857 0.30285 0.00753

M10 - M79 0.28789 0.00154 0.27744 0.28854 0.01046

M10 - M80 0.20176 0.00127 0.19632 0.20449 0.00544

M10 - M81 0.26379 0.00174 0.25059 0.25978 0.01321

M10 - M82 0.22747 0.00212 0.21592 0.22868 0.01156

M10 - M83 0.24800 0.00305 0.24109 0.24909 0.00690

M10 - M84 0.20929 0.00478 0.20283 0.19990 0.00646

M10 - M85 0.31928 0.00099 0.31111 0.31825 0.00816

M10 - M86 0.33775 0.00043 0.32764 0.33882 0.01011

M10 - M87 0.32322 0.00065 0.31548 0.32115 0.00774

M10 - M88 0.25610 0.00411 0.24125 0.24771 0.01485

M10 - M89 0.27707 0.00398 0.25995 0.27427 0.01712

M10 - M90 0.24731 0.00272 0.23115 0.23703 0.01616

M10 - M91 0.26972 0.00097 0.25914 0.26742 0.01058

M10 - M92 0.25904 0.00134 0.24965 0.26206 0.00939

M10 - M93 0.30828 0.00047 0.29494 0.30865 0.01335

M10 - M94 0.26827 0.00254 0.24860 0.26349 0.01967

M10 - M95 0.24529 0.00239 0.23960 0.23892 0.00569

M10 - M96 0.22534 0.00345 0.21945 0.22443 0.00588

M10 - M97 0.23851 0.00224 0.23098 0.23383 0.00753

M11 - M12 0.18039 0.00251 0.16974 0.17175 0.01065

M11 - M13 0.21475 0.00076 0.20191 0.21537 0.01283

M11 - M14 0.24113 0.00558 0.22884 0.23609 0.01229

M11 - M15 0.16242 0.00108 0.14794 0.15964 0.01448

M11 - M16 0.21473 0.00072 0.20206 0.21493 0.01268

M11 - M17 0.13990 0.00281 0.12617 0.13251 0.01373

M11 - M18 0.12784 0.00607 0.11705 0.12978 0.01079

M11 - M19 0.16624 0.01717 0.15366 0.14231 0.01258

M11 - M20 0.15200 0.00383 0.13761 0.16205 0.01439

M11 - M21 0.12538 0.01872 0.11518 0.10659 0.01020

M11 - M22 0.14782 0.00318 0.13733 0.13879 0.01049

M11 - M23 0.18259 0.00343 0.17038 0.17471 0.01221

M11 - M24 0.13945 0.00368 0.12297 0.13628 0.01648

M11 - M25 0.18569 0.00405 0.16398 0.18396 0.02170

M11 - M26 0.15609 0.00076 0.13968 0.15252 0.01641

M11 - M27 0.12667 0.00595 0.11443 0.11954 0.01224

M11 - M28 0.15522 0.00841 0.13976 0.13314 0.01547

M11 - M29 0.13341 0.00440 0.12255 0.14771 0.01085

M11 - M30 0.13166 0.01603 0.12148 0.12323 0.01018

M11 - M31 0.13731 0.01966 0.12605 0.12099 0.01126

M11 - M32 0.14360 0.01701 0.12933 0.13554 0.01426

M11 - M33 0.13687 0.01823 0.12753 0.11196 0.00933

M11 - M34 0.19634 0.00273 0.18583 0.18764 0.01051

M11 - M35 0.14646 0.00632 0.13559 0.14106 0.01087

M11 - M36 0.14206 0.00599 0.12429 0.13243 0.01777

M11 - M37 0.14305 0.00514 0.12712 0.13762 0.01593

M11 - M38 0.16455 0.01709 0.15307 0.14826 0.01147

M11 - M39 0.15970 0.00009 0.14242 0.16017 0.01728

M11 - M40 0.13896 0.01226 0.12815 0.12208 0.01081

M11 - M41 0.15279 0.00403 0.13758 0.15881 0.01521

M11 - M42 0.15110 0.00662 0.14117 0.15505 0.00993

M11 - M43 0.19205 0.01746 0.18031 0.17114 0.01173

M11 - M44 0.11936 0.00997 0.10964 0.11101 0.00971

M11 - M45 0.19415 0.00282 0.18392 0.18879 0.01023

M11 - M46 0.15930 0.00031 0.13989 0.15645 0.01941

M11 - M47 0.13718 0.00187 0.12674 0.14039 0.01044

M11 - M48 0.16047 0.00111 0.14780 0.16253 0.01266

M11 - M49 0.15717 0.00132 0.14245 0.16114 0.01473

M11 - M50 0.13038 0.00312 0.12116 0.12835 0.00922

M11 - M51 0.09371 0.02511 0.08287 0.08046 0.01085

M11 - M52 0.10952 0.02197 0.10192 0.08844 0.00760

M11 - M53 0.13630 0.00553 0.12579 0.13147 0.01051

M11 - M54 0.10976 0.00427 0.10277 0.10654 0.00699

M11 - M55 0.12582 0.02174 0.11595 0.09995 0.00986

M11 - M56 0.14153 0.02043 0.12875 0.10592 0.01278

M11 - M57 0.09985 0.00663 0.08908 0.09584 0.01078

M11 - M58 0.14814 0.00095 0.13091 0.14446 0.01723

M11 - M59 0.12986 0.01122 0.11761 0.11724 0.01225

M11 - M60 0.15158 0.00225 0.13585 0.13950 0.01572

M11 - M61 0.13235 0.00984 0.11879 0.12667 0.01356

M11 - M62 0.14467 0.00139 0.12804 0.14528 0.01664

M11 - M63 0.14444 0.00123 0.12814 0.14575 0.01630

M11 - M64 0.13463 0.01757 0.12539 0.11408 0.00925

M11 - M65 0.14835 0.00066 0.13163 0.14898 0.01672

M11 - M66 0.14730 0.00750 0.13720 0.13852 0.01010

M11 - M67 0.13846 0.00758 0.12827 0.12679 0.01019

M11 - M68 0.11781 0.01746 0.10825 0.09887 0.00956

M11 - M69 0.11691 0.00281 0.10625 0.11892 0.01066

M11 - M70 0.11870 0.00246 0.10583 0.12004 0.01287

M11 - M71 0.13984 0.01903 0.12953 0.11610 0.01031

M11 - M72 0.14495 0.01935 0.12838 0.10320 0.01658

M11 - M73 0.14428 0.00529 0.13353 0.15499 0.01075

M11 - M74 0.15312 0.00167 0.13928 0.15508 0.01383

M11 - M75 0.14031 0.00459 0.12774 0.14374 0.01257

M11 - M76 0.17097 0.00604 0.16171 0.17268 0.00926

M11 - M77 0.10778 0.02117 0.09972 0.10891 0.00806

M11 - M78 0.13687 0.00099 0.12425 0.14265 0.01262

M11 - M79 0.13624 0.00095 0.12433 0.13359 0.01191

M11 - M80 0.10907 0.02212 0.10070 0.09655 0.00837

M11 - M81 0.15115 0.00763 0.13467 0.14101 0.01648

M11 - M82 0.14255 0.00867 0.13042 0.13181 0.01213

M11 - M83 0.15359 0.00540 0.14213 0.14868 0.01146

M11 - M84 0.10680 0.00783 0.09596 0.10055 0.01085

M11 - M85 0.14562 0.00386 0.13191 0.15298 0.01372

M11 - M86 0.17208 0.01523 0.15751 0.17484 0.01457

M11 - M87 0.14882 0.00411 0.13502 0.15449 0.01380

M11 - M88 0.15629 0.01611 0.14600 0.14439 0.01028

M11 - M89 0.18807 0.01783 0.17830 0.16020 0.00976

M11 - M90 0.12632 0.00161 0.11323 0.12202 0.01310

M11 - M91 0.12172 0.00204 0.10927 0.12258 0.01245

M11 - M92 0.16195 0.01774 0.14742 0.14248 0.01453

M11 - M93 0.15646 0.00197 0.13686 0.14167 0.01959

M11 - M94 0.13958 0.00261 0.12623 0.14294 0.01336

M11 - M95 0.14644 0.01931 0.13743 0.12777 0.00902

M11 - M96 0.15146 0.02335 0.13983 0.12142 0.01162

M11 - M97 0.13955 0.01918 0.13019 0.12314 0.00935

M12 - M13 0.18681 0.00360 0.18292 0.18196 0.00389

M12 - M14 0.22432 0.01172 0.22162 0.20782 0.00269

M12 - M15 0.06354 0.00059 0.06019 0.06650 0.00334

M12 - M16 0.22028 0.02083 0.18696 0.20689 0.03333

M12 - M17 0.08273 0.01994 0.05857 0.08261 0.02415

M12 - M18 0.08513 0.00821 0.07762 0.07802 0.00751

M12 - M19 0.10325 0.02737 0.07572 0.09445 0.02753

M12 - M20 0.07211 0.00000 0.07124 0.07199 0.00087

M12 - M21 0.07962 0.01177 0.07778 0.06021 0.00183

M12 - M22 0.09162 0.03466 0.06240 0.08260 0.02922

M12 - M23 0.12745 0.03698 0.07834 0.11853 0.04912

M12 - M24 0.11488 0.00381 0.10592 0.11359 0.00896

M12 - M25 0.17665 0.00471 0.16223 0.16698 0.01442

M12 - M26 0.06404 0.00021 0.06121 0.06453 0.00283

M12 - M27 0.08213 0.00287 0.07677 0.07685 0.00536

M12 - M28 0.10276 0.00081 0.09581 0.10191 0.00695

M12 - M29 0.07361 0.00027 0.07330 0.07212 0.00031

M12 - M30 0.09278 0.00292 0.09062 0.08305 0.00216

M12 - M31 0.08848 0.00574 0.08573 0.07782 0.00275

M12 - M32 0.12450 0.00939 0.11921 0.10100 0.00529

M12 - M33 0.06694 0.00184 0.06659 0.06066 0.00034

M12 - M34 0.11497 0.03349 0.07381 0.12054 0.04116

M12 - M35 0.09604 0.00208 0.09448 0.09237 0.00156

M12 - M36 0.07594 0.00061 0.07530 0.07222 0.00065

M12 - M37 0.09359 0.00153 0.09069 0.09162 0.00290

M12 - M38 0.10453 0.00386 0.10236 0.10500 0.00217

M12 - M39 0.05223 0.00274 0.05013 0.06092 0.00210

M12 - M40 0.06332 0.00092 0.06037 0.06499 0.00295

M12 - M41 0.06839 0.00000 0.06749 0.06839 0.00090

M12 - M42 0.07698 0.00903 0.06891 0.08203 0.00807

M12 - M43 0.13525 0.01865 0.10230 0.12577 0.03295

M12 - M44 0.06260 0.00814 0.05445 0.06056 0.00815

M12 - M45 0.12643 0.04266 0.07692 0.12818 0.04951

M12 - M46 0.05840 0.00008 0.05778 0.05872 0.00061

M12 - M47 0.11636 0.00073 0.11317 0.11575 0.00319

M12 - M48 0.14102 0.00184 0.13457 0.13654 0.00645

M12 - M49 0.14337 0.00395 0.12868 0.13370 0.01469

M12 - M50 0.10896 0.00825 0.10834 0.10255 0.00062

M12 - M51 0.04679 0.00218 0.04601 0.04161 0.00078

M12 - M52 0.05129 0.00069 0.05017 0.05021 0.00111

M12 - M53 0.12669 0.00822 0.12182 0.11728 0.00487

M12 - M54 0.09467 0.00602 0.09437 0.08745 0.00030

M12 - M55 0.07129 0.00534 0.07067 0.06330 0.00062

M12 - M56 0.08245 0.00646 0.08182 0.06639 0.00063

M12 - M57 0.05181 0.00362 0.05128 0.04971 0.00053

M12 - M58 0.06094 0.00008 0.05820 0.06244 0.00275

M12 - M59 0.07055 0.00198 0.06754 0.06587 0.00301

M12 - M60 0.08427 0.00481 0.08289 0.06989 0.00138

M12 - M61 0.07560 0.00221 0.06825 0.07416 0.00734

M12 - M62 0.05988 0.00042 0.05876 0.06238 0.00112

M12 - M63 0.05966 0.00042 0.05847 0.06219 0.00119

M12 - M64 0.06809 0.00306 0.06756 0.06658 0.00053

M12 - M65 0.09625 0.00310 0.08979 0.09249 0.00646

M12 - M66 0.10224 0.00222 0.10182 0.10121 0.00043

M12 - M67 0.09287 0.00326 0.09162 0.09238 0.00125

M12 - M68 0.05373 0.00139 0.05303 0.05402 0.00070

M12 - M69 0.09132 0.00863 0.08988 0.07185 0.00144

M12 - M70 0.08483 0.00247 0.07710 0.08095 0.00773

M12 - M71 0.07793 0.00326 0.07165 0.07285 0.00628

M12 - M72 0.06061 0.00357 0.06007 0.05463 0.00054

M12 - M73 0.07130 0.00124 0.07055 0.07337 0.00075

M12 - M74 0.06150 0.00119 0.06067 0.06667 0.00083

M12 - M75 0.05643 0.00024 0.05553 0.05922 0.00090

M12 - M76 0.12415 0.03973 0.08724 0.12062 0.03690

M12 - M77 0.08319 0.00117 0.08152 0.07631 0.00167

M12 - M78 0.07255 0.00064 0.07202 0.06875 0.00053

M12 - M79 0.07609 0.00234 0.07446 0.08073 0.00163

M12 - M80 0.06330 0.00032 0.06292 0.06328 0.00037

M12 - M81 0.07488 0.00138 0.07080 0.07147 0.00408

M12 - M82 0.09888 0.00390 0.09169 0.09377 0.00719

M12 - M83 0.10785 0.00051 0.10125 0.10720 0.00660

M12 - M84 0.06495 0.00498 0.06086 0.06011 0.00409

M12 - M85 0.07108 0.00010 0.06987 0.07177 0.00121

M12 - M86 0.11549 0.00006 0.10813 0.11531 0.00736

M12 - M87 0.06945 0.00103 0.06861 0.07426 0.00084

M12 - M88 0.11980 0.01901 0.11788 0.10821 0.00192

M12 - M89 0.12822 0.00497 0.12746 0.12198 0.00076

M12 - M90 0.09478 0.00348 0.08674 0.09358 0.00804

M12 - M91 0.05393 0.00094 0.05158 0.05389 0.00236

M12 - M92 0.09535 0.00151 0.08752 0.08960 0.00783

M12 - M93 0.06286 0.00177 0.05914 0.06676 0.00372

M12 - M94 0.07493 0.00133 0.07417 0.07383 0.00076

M12 - M95 0.08172 0.00220 0.08124 0.07673 0.00048

M12 - M96 0.07387 0.00112 0.07356 0.07213 0.00031

M12 - M97 0.09299 0.00246 0.09100 0.08762 0.00200

M13 - M14 0.28956 0.02828 0.26561 0.24457 0.02395

M13 - M15 0.16272 0.00033 0.15639 0.16291 0.00633

M13 - M16 0.23886 0.01041 0.22709 0.22568 0.01177

M13 - M17 0.15566 0.00532 0.14972 0.14594 0.00594

M13 - M18 0.13817 0.00697 0.12673 0.13196 0.01144

M13 - M19 0.15957 0.00641 0.15366 0.14885 0.00592

M13 - M20 0.16571 0.00001 0.16065 0.16568 0.00505

M13 - M21 0.12365 0.00709 0.11996 0.11279 0.00368

M13 - M22 0.15989 0.00561 0.15604 0.14960 0.00385

M13 - M23 0.18911 0.00435 0.18223 0.17996 0.00688

M13 - M24 0.16141 0.00698 0.14205 0.15504 0.01936

M13 - M25 0.22546 0.00945 0.17902 0.21530 0.04645

M13 - M26 0.16266 0.00147 0.15250 0.15948 0.01016

M13 - M27 0.12159 0.00504 0.11433 0.11968 0.00726

M13 - M28 0.14706 0.00310 0.13852 0.14308 0.00855

M13 - M29 0.16096 0.00098 0.15568 0.15691 0.00528

M13 - M30 0.13274 0.00214 0.12779 0.13322 0.00495

M13 - M31 0.12435 0.00255 0.11676 0.12715 0.00759

M13 - M32 0.15688 0.00379 0.14604 0.15250 0.01085

M13 - M33 0.11699 0.00413 0.11335 0.11191 0.00364

M13 - M34 0.19916 0.00335 0.19483 0.19655 0.00433

M13 - M35 0.15363 0.00770 0.14911 0.14648 0.00453

M13 - M36 0.12573 0.00208 0.12195 0.12875 0.00378

M13 - M37 0.14687 0.00741 0.14004 0.13603 0.00683

M13 - M38 0.15217 0.00200 0.14771 0.14998 0.00446

M13 - M39 0.16107 0.00065 0.15551 0.15802 0.00556

M13 - M40 0.15082 0.00672 0.14245 0.13695 0.00836

M13 - M41 0.16559 0.00055 0.16009 0.16566 0.00550

M13 - M42 0.16002 0.00406 0.15601 0.15092 0.00401

M13 - M43 0.16663 0.00699 0.15947 0.16049 0.00716

M13 - M44 0.13418 0.00787 0.12889 0.11736 0.00529

M13 - M45 0.20575 0.00406 0.20139 0.20047 0.00435

M13 - M46 0.15778 0.00057 0.15066 0.16141 0.00712

M13 - M47 0.18135 0.00482 0.17668 0.16982 0.00467

M13 - M48 0.19011 0.00175 0.18468 0.19207 0.00543

M13 - M49 0.18718 0.00246 0.18067 0.18862 0.00651

M13 - M50 0.14200 0.00533 0.13752 0.14246 0.00448

M13 - M51 0.09860 0.00556 0.09428 0.09628 0.00431

M13 - M52 0.10964 0.00461 0.10577 0.10363 0.00387

M13 - M53 0.15465 0.00538 0.14799 0.15326 0.00666

M13 - M54 0.14721 0.00826 0.14230 0.13697 0.00491

M13 - M55 0.10517 0.00286 0.10062 0.10600 0.00455

M13 - M56 0.12020 0.00225 0.11493 0.11555 0.00527

M13 - M57 0.10659 0.00699 0.10303 0.10077 0.00356

M13 - M58 0.14965 0.00120 0.14166 0.15059 0.00800

M13 - M59 0.13437 0.00474 0.12270 0.12868 0.01167

M13 - M60 0.13912 0.00157 0.13372 0.13720 0.00541

M13 - M61 0.15450 0.00438 0.13707 0.14440 0.01743

M13 - M62 0.14938 0.00168 0.14057 0.15181 0.00880

M13 - M63 0.15149 0.00147 0.14320 0.15350 0.00829

M13 - M64 0.12913 0.00182 0.12461 0.12649 0.00453

M13 - M65 0.16724 0.00408 0.15562 0.16579 0.01162

M13 - M66 0.15565 0.00319 0.14620 0.15349 0.00945

M13 - M67 0.15005 0.00472 0.14554 0.14800 0.00451

M13 - M68 0.10618 0.00199 0.10234 0.10708 0.00384

M13 - M69 0.15060 0.00585 0.14528 0.13938 0.00531

M13 - M70 0.14583 0.00311 0.13569 0.13738 0.01013

M13 - M71 0.11645 0.00291 0.11168 0.11654 0.00477

M13 - M72 0.11047 0.00430 0.10659 0.10834 0.00388

M13 - M73 0.16433 0.00046 0.16036 0.16355 0.00397

M13 - M74 0.16732 0.00145 0.15723 0.16394 0.01008

M13 - M75 0.14556 0.00228 0.14151 0.13874 0.00405

M13 - M76 0.17939 0.00590 0.17279 0.17169 0.00660

M13 - M77 0.13345 0.00261 0.12814 0.12789 0.00531

M13 - M78 0.16158 0.00172 0.15369 0.15949 0.00788

M13 - M79 0.15402 0.00300 0.14407 0.15613 0.00995

M13 - M80 0.11401 0.00315 0.11085 0.10920 0.00316

M13 - M81 0.14286 0.00361 0.13869 0.13794 0.00418

M13 - M82 0.15366 0.01407 0.14822 0.13270 0.00544

M13 - M83 0.16007 0.00404 0.15607 0.15309 0.00400

M13 - M84 0.12450 0.01095 0.12089 0.11279 0.00361

M13 - M85 0.17156 0.00179 0.16562 0.16614 0.00594

M13 - M86 0.20144 0.00153 0.19453 0.19529 0.00691

M13 - M87 0.16619 0.00142 0.16098 0.16346 0.00521

M13 - M88 0.16393 0.00924 0.15250 0.14808 0.01143

M13 - M89 0.16737 0.00227 0.16124 0.16734 0.00613

M13 - M90 0.14613 0.00479 0.13976 0.14295 0.00637

M13 - M91 0.15067 0.00482 0.14613 0.14208 0.00454

M13 - M92 0.14864 0.00172 0.14165 0.14673 0.00699

M13 - M93 0.15757 0.00143 0.14998 0.15853 0.00759

M13 - M94 0.16522 0.00547 0.15193 0.14902 0.01329

M13 - M95 0.13156 0.00202 0.12730 0.12878 0.00427

M13 - M96 0.13273 0.00438 0.12922 0.12108 0.00350

M13 - M97 0.14100 0.00368 0.13702 0.13699 0.00398

M14 - M15 0.19760 0.00236 0.19691 0.18879 0.00069

M14 - M16 0.25701 0.00674 0.25322 0.24899 0.00379

M14 - M17 0.17725 0.00668 0.17470 0.16573 0.00256

M14 - M18 0.14959 0.00396 0.14817 0.14449 0.00142

M14 - M19 0.17493 0.00898 0.17397 0.16512 0.00097

M14 - M20 0.19553 0.00001 0.19264 0.19529 0.00289

M14 - M21 0.13983 0.00996 0.13822 0.13023 0.00161

M14 - M22 0.18158 0.01275 0.18040 0.16675 0.00118

M14 - M23 0.22227 0.01585 0.21355 0.20490 0.00873

M14 - M24 0.18834 0.00848 0.17826 0.17501 0.01008

M14 - M25 0.24314 0.00778 0.23125 0.22896 0.01189

M14 - M26 0.18031 0.00026 0.17862 0.18402 0.00168

M14 - M27 0.13850 0.00457 0.13380 0.13738 0.00471

M14 - M28 0.16881 0.00201 0.16251 0.16093 0.00630

M14 - M29 0.18055 0.00070 0.17823 0.17893 0.00232

M14 - M30 0.14521 0.00259 0.14312 0.15047 0.00209

M14 - M31 0.14063 0.00342 0.13784 0.14364 0.00278

M14 - M32 0.15741 0.00359 0.15315 0.15951 0.00427

M14 - M33 0.13592 0.00319 0.13536 0.12835 0.00056

M14 - M34 0.24134 0.01658 0.23760 0.21958 0.00375

M14 - M35 0.17507 0.00893 0.16612 0.16617 0.00895

M14 - M36 0.14023 0.00163 0.13278 0.14486 0.00745

M14 - M37 0.17193 0.01043 0.15467 0.15252 0.01726

M14 - M38 0.17985 0.00466 0.17793 0.17062 0.00192

M14 - M39 0.18810 0.00049 0.18760 0.18580 0.00050

M14 - M40 0.17427 0.01261 0.17199 0.15123 0.00228

M14 - M41 0.19505 0.00046 0.19243 0.19302 0.00262

M14 - M42 0.18069 0.00375 0.17985 0.17396 0.00083

M14 - M43 0.19396 0.00834 0.18877 0.19021 0.00519

M14 - M44 0.15856 0.01510 0.15732 0.13203 0.00123

M14 - M45 0.23981 0.01491 0.23457 0.22333 0.00524

M14 - M46 0.18919 0.00061 0.18862 0.18707 0.00057

M14 - M47 0.18478 0.00432 0.18100 0.18275 0.00377

M14 - M48 0.20399 0.00096 0.19724 0.20509 0.00675

M14 - M49 0.19446 0.00239 0.18654 0.20051 0.00793

M14 - M50 0.16574 0.01048 0.16532 0.16153 0.00042

M14 - M51 0.11518 0.00686 0.11432 0.11109 0.00086

M14 - M52 0.12263 0.00236 0.12128 0.11552 0.00135

M14 - M53 0.18398 0.00672 0.18076 0.17908 0.00322

M14 - M54 0.16574 0.01005 0.16450 0.15245 0.00124

M14 - M55 0.12974 0.00364 0.12902 0.12960 0.00072

M14 - M56 0.14348 0.00110 0.14283 0.14464 0.00065

M14 - M57 0.12461 0.00895 0.12297 0.11505 0.00164

M14 - M58 0.16479 0.00089 0.16326 0.16471 0.00153

M14 - M59 0.13928 0.00338 0.13671 0.13999 0.00257

M14 - M60 0.14976 0.00216 0.14800 0.15097 0.00176

M14 - M61 0.15944 0.00409 0.15512 0.15266 0.00431

M14 - M62 0.17048 0.00142 0.16961 0.16790 0.00087

M14 - M63 0.17340 0.00133 0.17241 0.17077 0.00099

M14 - M64 0.14841 0.00383 0.14699 0.13987 0.00142

M14 - M65 0.16970 0.00248 0.16483 0.17063 0.00487

M14 - M66 0.18312 0.00726 0.18252 0.17039 0.00060

M14 - M67 0.16049 0.00558 0.15919 0.15605 0.00129

M14 - M68 0.12760 0.00215 0.12411 0.12378 0.00349

M14 - M69 0.15245 0.00224 0.15111 0.15311 0.00134

M14 - M70 0.15819 0.00402 0.15399 0.15013 0.00421

M14 - M71 0.13753 0.00276 0.13513 0.13525 0.00240

M14 - M72 0.12771 0.00343 0.12619 0.12375 0.00153

M14 - M73 0.18904 0.00075 0.18728 0.18429 0.00177

M14 - M74 0.17420 0.00038 0.17385 0.17542 0.00035

M14 - M75 0.16001 0.00148 0.15672 0.15858 0.00329

M14 - M76 0.20505 0.01252 0.20445 0.19210 0.00061

M14 - M77 0.15805 0.00268 0.15158 0.14522 0.00647

M14 - M78 0.17505 0.00296 0.17415 0.18165 0.00090

M14 - M79 0.17611 0.00351 0.17553 0.16487 0.00057

M14 - M80 0.12727 0.00230 0.12407 0.12263 0.00320

M14 - M81 0.15499 0.00263 0.15132 0.15435 0.00366

M14 - M82 0.17048 0.01546 0.16745 0.14755 0.00303

M14 - M83 0.17239 0.00183 0.16486 0.17116 0.00752

M14 - M84 0.14252 0.01530 0.14074 0.12336 0.00178

M14 - M85 0.18856 0.00171 0.18638 0.18624 0.00219

M14 - M86 0.22486 0.00152 0.21647 0.22102 0.00839

M14 - M87 0.19010 0.00182 0.18870 0.18678 0.00141

M14 - M88 0.18471 0.01030 0.18320 0.16216 0.00151

M14 - M89 0.19822 0.00493 0.19199 0.19148 0.00623

M14 - M90 0.16413 0.00350 0.16104 0.15981 0.00309

M14 - M91 0.17568 0.00473 0.17398 0.16273 0.00170

M14 - M92 0.16803 0.00255 0.15770 0.16886 0.01033

M14 - M93 0.19536 0.00240 0.19395 0.18968 0.00141

M14 - M94 0.16729 0.00355 0.16636 0.15933 0.00092

M14 - M95 0.14414 0.00354 0.14219 0.13957 0.00195

M14 - M96 0.13563 0.00281 0.13508 0.13574 0.00054

M14 - M97 0.15671 0.00410 0.15156 0.15456 0.00515

M15 - M16 0.18516 0.00085 0.17543 0.18272 0.00973

M15 - M17 0.04070 0.00186 0.03216 0.04324 0.00854

M15 - M18 0.06277 0.00097 0.06237 0.05977 0.00040

M15 - M19 0.06350 0.00170 0.05882 0.06809 0.00468

M15 - M20 0.02093 0.00000 0.02008 0.02093 0.00085

M15 - M21 0.03120 0.00059 0.03085 0.03024 0.00035

M15 - M22 0.04663 0.00215 0.04263 0.05266 0.00400

M15 - M23 0.07662 0.00300 0.06350 0.08462 0.01312

M15 - M24 0.09423 0.00092 0.08168 0.08996 0.01255

M15 - M25 0.17181 0.00187 0.15203 0.16288 0.01978

M15 - M26 0.01963 0.00007 0.01622 0.01909 0.00341

M15 - M27 0.04976 0.00011 0.04794 0.05064 0.00182

M15 - M28 0.07393 0.00038 0.06531 0.07209 0.00862

M15 - M29 0.02726 0.00003 0.02694 0.02696 0.00032

M15 - M30 0.05216 0.00094 0.04999 0.04982 0.00217

M15 - M31 0.05349 0.00162 0.04936 0.04882 0.00413

M15 - M32 0.09111 0.00130 0.08439 0.08729 0.00672

M15 - M33 0.03532 0.00051 0.03498 0.03346 0.00034

M15 - M34 0.07415 0.00299 0.06700 0.08413 0.00716

M15 - M35 0.07919 0.00180 0.07790 0.07479 0.00129

M15 - M36 0.04380 0.00049 0.04349 0.04169 0.00031

M15 - M37 0.07508 0.00166 0.07425 0.07043 0.00082

M15 - M38 0.08163 0.00095 0.08045 0.07716 0.00118

M15 - M39 0.01631 0.00043 0.01590 0.01328 0.00041

M15 - M40 0.03485 0.00029 0.03134 0.03306 0.00350

M15 - M41 0.01999 0.00028 0.01922 0.01996 0.00077

M15 - M42 0.05041 0.00088 0.04961 0.05172 0.00080

M15 - M43 0.09677 0.00091 0.08766 0.10258 0.00911

M15 - M44 0.03374 0.00043 0.03196 0.03030 0.00178

M15 - M45 0.08180 0.00369 0.07358 0.09225 0.00822

M15 - M46 0.00819 0.00017 0.00768 0.00887 0.00050

M15 - M47 0.09106 0.00070 0.08969 0.09019 0.00137

M15 - M48 0.10928 0.00042 0.10647 0.11003 0.00281

M15 - M49 0.11055 0.00070 0.10420 0.11094 0.00635

M15 - M50 0.07600 0.00174 0.07563 0.07517 0.00037

M15 - M51 0.01317 0.00069 0.01268 0.01275 0.00049

M15 - M52 0.02720 0.00046 0.02693 0.02548 0.00026

M15 - M53 0.08909 0.00023 0.08449 0.08973 0.00461

M15 - M54 0.06000 0.00018 0.05967 0.06090 0.00033

M15 - M55 0.03573 0.00017 0.03469 0.03552 0.00104

M15 - M56 0.03676 0.00147 0.03624 0.03272 0.00052

M15 - M57 0.02383 0.00055 0.02309 0.02314 0.00074

M15 - M58 0.02213 0.00021 0.01988 0.02097 0.00225

M15 - M59 0.04254 0.00087 0.03976 0.03995 0.00277

M15 - M60 0.04059 0.00146 0.03872 0.03584 0.00187

M15 - M61 0.05215 0.00044 0.04454 0.05062 0.00761

M15 - M62 0.02309 0.00010 0.02192 0.02282 0.00117

M15 - M63 0.02196 0.00010 0.02076 0.02161 0.00120

M15 - M64 0.03871 0.00022 0.03840 0.03787 0.00030

M15 - M65 0.06863 0.00074 0.05870 0.06695 0.00993

M15 - M66 0.07721 0.00249 0.07692 0.07248 0.00029

M15 - M67 0.07403 0.00030 0.07324 0.07411 0.00079

M15 - M68 0.02576 0.00026 0.02502 0.02600 0.00073

M15 - M69 0.03909 0.00030 0.03718 0.03882 0.00191

M15 - M70 0.05488 0.00123 0.04596 0.05464 0.00892

M15 - M71 0.04452 0.00013 0.04198 0.04589 0.00254

M15 - M72 0.02600 0.00050 0.02533 0.02433 0.00066

M15 - M73 0.02405 0.00074 0.02316 0.02656 0.00089

M15 - M74 0.02108 0.00103 0.01968 0.02394 0.00140

M15 - M75 0.02033 0.00081 0.01954 0.02177 0.00079

M15 - M76 0.08045 0.00299 0.07545 0.09015 0.00500

M15 - M77 0.03750 0.00033 0.03622 0.03866 0.00128

M15 - M78 0.02278 0.00036 0.02229 0.02178 0.00049

M15 - M79 0.04576 0.00064 0.04514 0.04675 0.00062

M15 - M80 0.02977 0.00055 0.02933 0.03080 0.00044

M15 - M81 0.03659 0.00030 0.03426 0.03753 0.00232

M15 - M82 0.06850 0.00032 0.06217 0.06771 0.00633

M15 - M83 0.08204 0.00021 0.08003 0.08236 0.00201

M15 - M84 0.03389 0.00104 0.03078 0.03435 0.00312

M15 - M85 0.02835 0.00013 0.02734 0.02905 0.00100

M15 - M86 0.07680 0.00003 0.07053 0.07700 0.00627

M15 - M87 0.02906 0.00017 0.02819 0.02973 0.00087

M15 - M88 0.07979 0.00124 0.07816 0.07886 0.00163

M15 - M89 0.09914 0.00194 0.09858 0.09499 0.00056

M15 - M90 0.06781 0.00023 0.06267 0.06671 0.00514

M15 - M91 0.01855 0.00087 0.01542 0.01898 0.00312

M15 - M92 0.06647 0.00060 0.05901 0.06279 0.00746

M15 - M93 0.02457 0.00013 0.02272 0.02473 0.00185

M15 - M94 0.04664 0.00031 0.04511 0.04659 0.00153

M15 - M95 0.05912 0.00128 0.05883 0.05548 0.00029

M15 - M96 0.05309 0.00075 0.05282 0.05144 0.00027

M15 - M97 0.06312 0.00036 0.06229 0.05949 0.00082

M16 - M17 0.18080 0.01275 0.16029 0.17069 0.02051

M16 - M18 0.15038 0.01072 0.14408 0.13510 0.00631

M16 - M19 0.19626 0.02317 0.15846 0.17374 0.03780

M16 - M20 0.18065 0.00001 0.17624 0.18070 0.00441

M16 - M21 0.13163 0.01440 0.12651 0.11765 0.00512

M16 - M22 0.18075 0.02624 0.14220 0.16080 0.03855

M16 - M23 0.21490 0.02646 0.17872 0.18976 0.03618

M16 - M24 0.18022 0.01560 0.16926 0.16525 0.01096

M16 - M25 0.22564 0.00823 0.21108 0.21882 0.01455

M16 - M26 0.16883 0.00189 0.16104 0.16903 0.00778

M16 - M27 0.12840 0.00653 0.12240 0.12552 0.00600

M16 - M28 0.15425 0.00178 0.14601 0.15424 0.00825

M16 - M29 0.16055 0.00281 0.15458 0.16117 0.00597

M16 - M30 0.14445 0.00191 0.13843 0.14489 0.00602

M16 - M31 0.14841 0.00892 0.14241 0.14249 0.00600

M16 - M32 0.16423 0.00862 0.15652 0.15898 0.00771

M16 - M33 0.13382 0.00850 0.13020 0.12448 0.00362

M16 - M34 0.23738 0.02511 0.20009 0.21720 0.03730

M16 - M35 0.14642 0.00191 0.14262 0.14489 0.00380

M16 - M36 0.16295 0.00736 0.15855 0.15173 0.00440

M16 - M37 0.13904 0.00191 0.13552 0.13791 0.00352

M16 - M38 0.14991 0.00119 0.14486 0.14762 0.00504

M16 - M39 0.17898 0.00203 0.17158 0.17868 0.00741

M16 - M40 0.14035 0.00405 0.13575 0.13339 0.00459

M16 - M41 0.17911 0.00018 0.17494 0.17757 0.00416

M16 - M42 0.16698 0.00471 0.15818 0.16177 0.00880

M16 - M43 0.19300 0.01706 0.17127 0.17626 0.02173

M16 - M44 0.13235 0.00466 0.12588 0.12616 0.00647

M16 - M45 0.24446 0.02744 0.20300 0.22103 0.04145

M16 - M46 0.17404 0.00131 0.16659 0.17581 0.00745

M16 - M47 0.17999 0.00223 0.17515 0.17732 0.00484

M16 - M48 0.20075 0.00278 0.19508 0.19798 0.00567

M16 - M49 0.20064 0.00507 0.19050 0.19218 0.01015

M16 - M50 0.17777 0.01604 0.17382 0.15969 0.00395

M16 - M51 0.10706 0.00188 0.10372 0.10393 0.00334

M16 - M52 0.11065 0.00236 0.10751 0.10773 0.00314

M16 - M53 0.17022 0.00323 0.15687 0.16861 0.01334

M16 - M54 0.15033 0.00112 0.14640 0.14785 0.00393

M16 - M55 0.13149 0.01088 0.12802 0.12205 0.00347

M16 - M56 0.16017 0.01405 0.15686 0.14261 0.00330

M16 - M57 0.11479 0.00869 0.11069 0.10846 0.00410

M16 - M58 0.16229 0.00110 0.15666 0.15803 0.00563

M16 - M59 0.14104 0.00619 0.13604 0.13285 0.00501

M16 - M60 0.16544 0.00954 0.16127 0.14868 0.00417

M16 - M61 0.14377 0.00410 0.13634 0.13924 0.00743

M16 - M62 0.15884 0.00358 0.15211 0.15640 0.00673

M16 - M63 0.15851 0.00334 0.15115 0.15700 0.00737

M16 - M64 0.12693 0.00393 0.12347 0.12408 0.00346

M16 - M65 0.16289 0.00501 0.15494 0.15793 0.00794

M16 - M66 0.16357 0.00435 0.15128 0.16005 0.01229

M16 - M67 0.16533 0.01115 0.15670 0.15339 0.00863

M16 - M68 0.12393 0.01015 0.11971 0.11643 0.00423

M16 - M69 0.15615 0.00701 0.15177 0.14301 0.00438

M16 - M70 0.14204 0.00258 0.13356 0.14123 0.00848

M16 - M71 0.12371 0.00108 0.11950 0.12481 0.00421

M16 - M72 0.14010 0.01268 0.13642 0.12374 0.00368

M16 - M73 0.16970 0.00037 0.16271 0.17285 0.00700

M16 - M74 0.16614 0.00078 0.15861 0.16652 0.00754

M16 - M75 0.14824 0.00302 0.14410 0.14848 0.00414

M16 - M76 0.22203 0.02712 0.18445 0.19950 0.03758

M16 - M77 0.16132 0.00935 0.15568 0.15198 0.00563

M16 - M78 0.17335 0.00335 0.16622 0.16713 0.00713

M16 - M79 0.17258 0.00946 0.16158 0.16495 0.01100

M16 - M80 0.12844 0.00835 0.12552 0.12204 0.00292

M16 - M81 0.14994 0.00499 0.14564 0.14126 0.00430

M16 - M82 0.15143 0.00808 0.14406 0.14514 0.00737

M16 - M83 0.16694 0.01115 0.16155 0.15478 0.00539

M16 - M84 0.12918 0.00970 0.12416 0.12059 0.00502

M16 - M85 0.16797 0.00143 0.16400 0.16427 0.00398

M16 - M86 0.20025 0.00213 0.19331 0.19629 0.00693

M16 - M87 0.16752 0.00213 0.16325 0.16705 0.00427

M16 - M88 0.16799 0.01124 0.16298 0.15990 0.00502

M16 - M89 0.15614 0.00120 0.15011 0.15778 0.00603

M16 - M90 0.15574 0.00333 0.14273 0.15391 0.01301

M16 - M91 0.14152 0.00159 0.13540 0.13936 0.00611

M16 - M92 0.15350 0.00227 0.14609 0.14935 0.00741

M16 - M93 0.17269 0.00159 0.16070 0.17381 0.01199

M16 - M94 0.15515 0.00564 0.14890 0.15346 0.00625

M16 - M95 0.14513 0.00578 0.14173 0.13895 0.00339

M16 - M96 0.13140 0.00463 0.12836 0.12585 0.00304

M16 - M97 0.14713 0.00433 0.14334 0.14483 0.00379

M17 - M18 0.06677 0.00718 0.06133 0.05981 0.00544

M17 - M19 0.09027 0.02177 0.06369 0.07595 0.02659

M17 - M20 0.04061 0.00000 0.03756 0.04061 0.00305

M17 - M21 0.04580 0.00608 0.04325 0.04052 0.00256

M17 - M22 0.08007 0.02143 0.05438 0.06728 0.02570

M17 - M23 0.10188 0.02266 0.06650 0.09110 0.03539

M17 - M24 0.09341 0.00401 0.08361 0.09308 0.00980

M17 - M25 0.15850 0.00554 0.14420 0.14926 0.01430

M17 - M26 0.04310 0.00099 0.03665 0.04163 0.00646

M17 - M27 0.06151 0.00238 0.05725 0.05404 0.00426

M17 - M28 0.07990 0.00239 0.07258 0.07442 0.00733

M17 - M29 0.04464 0.00145 0.04041 0.04559 0.00423

M17 - M30 0.06050 0.00201 0.05721 0.05988 0.00330

M17 - M31 0.06032 0.00267 0.05626 0.06002 0.00406

M17 - M32 0.08914 0.00246 0.08331 0.08436 0.00583

M17 - M33 0.05273 0.00563 0.04981 0.04102 0.00292

M17 - M34 0.09549 0.01958 0.06839 0.09637 0.02709

M17 - M35 0.06652 0.00489 0.06331 0.06682 0.00321

M17 - M36 0.05383 0.00159 0.05105 0.05486 0.00278

M17 - M37 0.06692 0.00444 0.06238 0.06572 0.00454

M17 - M38 0.08075 0.00796 0.07653 0.07849 0.00422

M17 - M39 0.03602 0.00045 0.03150 0.03761 0.00451

M17 - M40 0.04977 0.00252 0.04410 0.04340 0.00567

M17 - M41 0.04033 0.00015 0.03734 0.04001 0.00299

M17 - M42 0.06117 0.00845 0.05381 0.05550 0.00736

M17 - M43 0.09812 0.01248 0.08006 0.09434 0.01806

M17 - M44 0.05348 0.01109 0.04373 0.04161 0.00975

M17 - M45 0.10493 0.02660 0.07244 0.10231 0.03249

M17 - M46 0.03130 0.00008 0.02785 0.03239 0.00345

M17 - M47 0.09903 0.00266 0.09589 0.09403 0.00313

M17 - M48 0.11078 0.00285 0.10528 0.10969 0.00550

M17 - M49 0.11276 0.00310 0.10082 0.10865 0.01194

M17 - M50 0.08851 0.01147 0.08605 0.08247 0.00246

M17 - M51 0.03474 0.00626 0.03178 0.02501 0.00296

M17 - M52 0.03837 0.00393 0.03625 0.03482 0.00212

M17 - M53 0.09445 0.00272 0.08624 0.09203 0.00821

M17 - M54 0.07550 0.00378 0.07338 0.07137 0.00212

M17 - M55 0.04900 0.00540 0.04648 0.04405 0.00253

M17 - M56 0.04987 0.00584 0.04755 0.04308 0.00232

M17 - M57 0.03840 0.00311 0.03599 0.03403 0.00241

M17 - M58 0.04136 0.00068 0.03433 0.04199 0.00703

M17 - M59 0.05695 0.00312 0.05258 0.05325 0.00436

M17 - M60 0.05605 0.00172 0.05135 0.05247 0.00469

M17 - M61 0.06731 0.00310 0.05756 0.06061 0.00976

M17 - M62 0.03978 0.00029 0.03600 0.04037 0.00378

M17 - M63 0.03924 0.00026 0.03522 0.03982 0.00401

M17 - M64 0.04827 0.00285 0.04613 0.04479 0.00214

M17 - M65 0.07819 0.00432 0.06764 0.07205 0.01055

M17 - M66 0.07109 0.00145 0.06820 0.07492 0.00289

M17 - M67 0.07770 0.00381 0.07390 0.07827 0.00381

M17 - M68 0.04255 0.00390 0.04018 0.03886 0.00237

M17 - M69 0.06056 0.00535 0.05692 0.05546 0.00363

M17 - M70 0.07754 0.00648 0.06690 0.06568 0.01064

M17 - M71 0.05046 0.00158 0.04649 0.05162 0.00397

M17 - M72 0.05031 0.00630 0.04745 0.03921 0.00287

M17 - M73 0.04538 0.00265 0.04104 0.04567 0.00434

M17 - M74 0.04334 0.00288 0.03850 0.04376 0.00483

M17 - M75 0.04050 0.00278 0.03754 0.03790 0.00296

M17 - M76 0.10064 0.02399 0.07621 0.09578 0.02444

M17 - M77 0.06407 0.00529 0.06126 0.05287 0.00281

M17 - M78 0.04839 0.00308 0.04506 0.04352 0.00333

M17 - M79 0.05752 0.00445 0.05066 0.05994 0.00685

M17 - M80 0.04587 0.00276 0.04367 0.04167 0.00220

M17 - M81 0.04586 0.00187 0.04192 0.04380 0.00393

M17 - M82 0.07692 0.00784 0.06860 0.06877 0.00832

M17 - M83 0.07975 0.00243 0.07581 0.07590 0.00394

M17 - M84 0.05094 0.00865 0.04709 0.04354 0.00385

M17 - M85 0.04018 0.00183 0.03684 0.04119 0.00334

M17 - M86 0.07910 0.00152 0.07276 0.07889 0.00634

M17 - M87 0.04378 0.00226 0.04021 0.04340 0.00357

M17 - M88 0.10269 0.01509 0.09936 0.08286 0.00333

M17 - M89 0.08885 0.00537 0.08471 0.09120 0.00413

M17 - M90 0.09676 0.00888 0.08721 0.07817 0.00955

M17 - M91 0.04257 0.00319 0.03314 0.04069 0.00943

M17 - M92 0.06607 0.00252 0.05927 0.06710 0.00680

M17 - M93 0.05374 0.00270 0.04559 0.04823 0.00815

M17 - M94 0.06666 0.00455 0.06249 0.05913 0.00417

M17 - M95 0.06735 0.00800 0.06314 0.05699 0.00421

M17 - M96 0.05845 0.00439 0.05631 0.05171 0.00215

M17 - M97 0.07322 0.00672 0.07062 0.06329 0.00260

M18 - M19 0.09712 0.01541 0.07949 0.07469 0.01763

M18 - M20 0.08590 0.01519 0.08427 0.06936 0.00163

M18 - M21 0.06133 0.01250 0.05751 0.04849 0.00381

M18 - M22 0.08189 0.01107 0.07375 0.06600 0.00814

M18 - M23 0.09847 0.01127 0.09066 0.08523 0.00781

M18 - M24 0.08806 0.00444 0.07933 0.08272 0.00874

M18 - M25 0.13418 0.00285 0.11520 0.13299 0.01899

M18 - M26 0.06488 0.00168 0.06285 0.06312 0.00203

M18 - M27 0.06755 0.01063 0.06659 0.05944 0.00096

M18 - M28 0.07591 0.00731 0.07087 0.07283 0.00504

M18 - M29 0.08166 0.01601 0.08118 0.06302 0.00049

M18 - M30 0.06142 0.00907 0.06037 0.06009 0.00106

M18 - M31 0.06315 0.00985 0.06088 0.05989 0.00227

M18 - M32 0.08449 0.00942 0.08084 0.08190 0.00366

M18 - M33 0.05944 0.01110 0.05926 0.05022 0.00019

M18 - M34 0.09940 0.00876 0.09117 0.09047 0.00822

M18 - M35 0.08110 0.00677 0.07850 0.07938 0.00261

M18 - M36 0.08279 0.01859 0.08060 0.06130 0.00220

M18 - M37 0.07758 0.00786 0.07614 0.07142 0.00143

M18 - M38 0.08375 0.00900 0.08269 0.07690 0.00106

M18 - M39 0.06108 0.00120 0.06095 0.06019 0.00012

M18 - M40 0.08199 0.01906 0.07318 0.05868 0.00880

M18 - M41 0.08478 0.01551 0.08269 0.06717 0.00209

M18 - M42 0.07834 0.00828 0.06860 0.07087 0.00975

M18 - M43 0.11038 0.01285 0.09816 0.09744 0.01222

M18 - M44 0.05485 0.00801 0.05414 0.04823 0.00072

M18 - M45 0.10412 0.00985 0.09379 0.09322 0.01033

M18 - M46 0.06752 0.00059 0.06731 0.06399 0.00021

M18 - M47 0.09225 0.00535 0.09048 0.08755 0.00177

M18 - M48 0.10522 0.00201 0.10366 0.10258 0.00156

M18 - M49 0.11183 0.00479 0.10914 0.10346 0.00269

M18 - M50 0.08011 0.00468 0.07965 0.07263 0.00046

M18 - M51 0.05642 0.00372 0.05361 0.05305 0.00282

M18 - M52 0.06759 0.00608 0.06563 0.06106 0.00196

M18 - M53 0.12711 0.00973 0.12040 0.11401 0.00671

M18 - M54 0.10656 0.00970 0.10306 0.09721 0.00350

M18 - M55 0.07161 0.00557 0.07002 0.06763 0.00159

M18 - M56 0.07548 0.00334 0.07420 0.07006 0.00128

M18 - M57 0.05190 0.00939 0.05047 0.04446 0.00143

M18 - M58 0.06076 0.00151 0.05983 0.05948 0.00093

M18 - M59 0.05799 0.00440 0.05631 0.05602 0.00167

M18 - M60 0.06601 0.00513 0.06517 0.05798 0.00085

M18 - M61 0.06813 0.00657 0.06419 0.06576 0.00394

M18 - M62 0.06148 0.00151 0.06024 0.06154 0.00124

M18 - M63 0.06035 0.00141 0.05921 0.06134 0.00114

M18 - M64 0.06755 0.01255 0.06337 0.05800 0.00418

M18 - M65 0.07938 0.00214 0.07206 0.07725 0.00732

M18 - M66 0.07677 0.00446 0.07579 0.07236 0.00099

M18 - M67 0.08818 0.00871 0.08744 0.07489 0.00073

M18 - M68 0.05166 0.00780 0.05127 0.04854 0.00038

M18 - M69 0.05789 0.00225 0.05618 0.05754 0.00171

M18 - M70 0.06716 0.00229 0.06308 0.06314 0.00408

M18 - M71 0.06480 0.00986 0.06363 0.05784 0.00117

M18 - M72 0.05384 0.00859 0.05254 0.04550 0.00129

M18 - M73 0.07069 0.00416 0.07004 0.06603 0.00065

M18 - M74 0.06351 0.00086 0.06320 0.06097 0.00031

M18 - M75 0.06381 0.00509 0.06317 0.06267 0.00064

M18 - M76 0.10647 0.01412 0.08732 0.08972 0.01915

M18 - M77 0.07242 0.00171 0.06958 0.07318 0.00285

M18 - M78 0.06639 0.00233 0.06560 0.05830 0.00079

M18 - M79 0.09607 0.00344 0.09205 0.09729 0.00401

M18 - M80 0.06619 0.00230 0.06519 0.06359 0.00100

M18 - M81 0.07418 0.00831 0.07266 0.06470 0.00152

M18 - M82 0.07841 0.00936 0.07476 0.06894 0.00364

M18 - M83 0.08855 0.00791 0.08494 0.08355 0.00362

M18 - M84 0.05658 0.00800 0.05483 0.05179 0.00174

M18 - M85 0.07774 0.00445 0.07671 0.07361 0.00103

M18 - M86 0.10434 0.00506 0.10057 0.10243 0.00377

M18 - M87 0.07578 0.00440 0.07467 0.07337 0.00111

M18 - M88 0.08318 0.00774 0.07916 0.07907 0.00402

M18 - M89 0.09825 0.00738 0.09810 0.09151 0.00015

M18 - M90 0.07098 0.00343 0.06842 0.06674 0.00256

M18 - M91 0.05387 0.00366 0.05281 0.05349 0.00106

M18 - M92 0.07885 0.00910 0.07190 0.07232 0.00695

M18 - M93 0.06036 0.00087 0.05984 0.06257 0.00052

M18 - M94 0.07241 0.00435 0.07169 0.06702 0.00072

M18 - M95 0.07141 0.00715 0.07117 0.06575 0.00023

M18 - M96 0.06387 0.00675 0.06343 0.06276 0.00044

M18 - M97 0.07110 0.00560 0.06921 0.06909 0.00190

M19 - M20 0.08654 0.00380 0.08546 0.07776 0.00108

M19 - M21 0.08858 0.02713 0.08208 0.05307 0.00649

M19 - M22 0.09980 0.03134 0.06579 0.07249 0.03401

M19 - M23 0.11910 0.03222 0.08002 0.09146 0.03907

M19 - M24 0.09353 0.00976 0.08573 0.08772 0.00780

M19 - M25 0.15140 0.00797 0.13995 0.14222 0.01145

M19 - M26 0.06578 0.00064 0.06281 0.06402 0.00297

M19 - M27 0.07730 0.00602 0.07575 0.06767 0.00155

M19 - M28 0.09050 0.00535 0.08520 0.08792 0.00530

M19 - M29 0.08270 0.00443 0.08197 0.07620 0.00073

M19 - M30 0.10144 0.01787 0.09961 0.07491 0.00183

M19 - M31 0.10286 0.02082 0.09837 0.07066 0.00450

M19 - M32 0.13824 0.02456 0.13013 0.09527 0.00812

M19 - M33 0.08437 0.02134 0.08366 0.05610 0.00071

M19 - M34 0.12000 0.02969 0.08642 0.10575 0.03358

M19 - M35 0.08922 0.00738 0.08755 0.08165 0.00167

M19 - M36 0.08395 0.00680 0.08219 0.07717 0.00176

M19 - M37 0.08874 0.00688 0.08757 0.07837 0.00117

M19 - M38 0.10626 0.01951 0.10449 0.08102 0.00177

M19 - M39 0.06046 0.00161 0.05791 0.06382 0.00256

M19 - M40 0.07305 0.00867 0.06988 0.06501 0.00318

M19 - M41 0.08311 0.00393 0.08187 0.07508 0.00123

M19 - M42 0.08903 0.01548 0.07849 0.07670 0.01054

M19 - M43 0.14340 0.03191 0.10506 0.10972 0.03834

M19 - M44 0.06720 0.01172 0.06581 0.05718 0.00138

M19 - M45 0.12988 0.03704 0.08992 0.11021 0.03996

M19 - M46 0.05909 0.00030 0.05782 0.06275 0.00127

M19 - M47 0.10110 0.00440 0.09846 0.09380 0.00265

M19 - M48 0.11286 0.00683 0.11057 0.11039 0.00229

M19 - M49 0.11972 0.00681 0.11566 0.10850 0.00406

M19 - M50 0.09304 0.00891 0.09242 0.08291 0.00062

M19 - M51 0.06971 0.02038 0.06734 0.04504 0.00237

M19 - M52 0.07593 0.02297 0.07297 0.05194 0.00295

M19 - M53 0.10135 0.00517 0.09771 0.09049 0.00364

M19 - M54 0.07928 0.00425 0.07799 0.07274 0.00129

M19 - M55 0.08619 0.02549 0.08271 0.06361 0.00348

M19 - M56 0.09722 0.03304 0.09325 0.06790 0.00398

M19 - M57 0.06145 0.01753 0.06019 0.04862 0.00126

M19 - M58 0.06238 0.00167 0.06092 0.06334 0.00146

M19 - M59 0.06818 0.00735 0.06614 0.05591 0.00204

M19 - M60 0.07956 0.00724 0.07829 0.06308 0.00128

M19 - M61 0.07242 0.00433 0.06766 0.06497 0.00477

M19 - M62 0.06635 0.00575 0.06403 0.06444 0.00232

M19 - M63 0.06652 0.00572 0.06388 0.06441 0.00264

M19 - M64 0.08835 0.01891 0.08430 0.06118 0.00405

M19 - M65 0.08454 0.00452 0.07899 0.08225 0.00556

M19 - M66 0.07787 0.00335 0.07731 0.07969 0.00056

M19 - M67 0.08321 0.00836 0.08225 0.07802 0.00096

M19 - M68 0.07838 0.01774 0.07640 0.05281 0.00198

M19 - M69 0.07674 0.00705 0.07497 0.06370 0.00177

M19 - M70 0.07326 0.00454 0.06794 0.06621 0.00531

M19 - M71 0.08644 0.02026 0.08081 0.06453 0.00564

M19 - M72 0.07796 0.02031 0.07673 0.05508 0.00123

M19 - M73 0.08103 0.00556 0.07966 0.07812 0.00137

M19 - M74 0.06178 0.00120 0.05978 0.06559 0.00200

M19 - M75 0.06927 0.00485 0.06815 0.06344 0.00112

M19 - M76 0.14194 0.04071 0.10842 0.11010 0.03352

M19 - M77 0.11082 0.02183 0.10962 0.07951 0.00119

M19 - M78 0.07504 0.00190 0.07412 0.06759 0.00092

M19 - M79 0.07936 0.00498 0.07858 0.07694 0.00078

M19 - M80 0.09130 0.02285 0.09118 0.06373 0.00012

M19 - M81 0.08535 0.00667 0.08012 0.06908 0.00523

M19 - M82 0.09466 0.01336 0.09126 0.08247 0.00340

M19 - M83 0.09074 0.00540 0.08906 0.09176 0.00168

M19 - M84 0.06400 0.00902 0.06276 0.05900 0.00124

M19 - M85 0.08199 0.00484 0.08073 0.07484 0.00126

M19 - M86 0.13464 0.01569 0.12889 0.10334 0.00575

M19 - M87 0.08524 0.00506 0.08402 0.07508 0.00121

M19 - M88 0.13748 0.03090 0.13583 0.09480 0.00165

M19 - M89 0.11608 0.01916 0.11453 0.10256 0.00155

M19 - M90 0.08607 0.00479 0.08239 0.07888 0.00368

M19 - M91 0.06225 0.00471 0.05987 0.05758 0.00238

M19 - M92 0.11536 0.01934 0.10654 0.08133 0.00882

M19 - M93 0.07480 0.00171 0.07226 0.07352 0.00254

M19 - M94 0.07383 0.00375 0.07257 0.06897 0.00125

M19 - M95 0.10670 0.02610 0.10541 0.07257 0.00129

M19 - M96 0.09247 0.02314 0.09189 0.06559 0.00058

M19 - M97 0.10485 0.01855 0.10302 0.08137 0.00183

M20 - M21 0.02874 0.00382 0.02760 0.03165 0.00114

M20 - M22 0.05419 0.00000 0.05329 0.05416 0.00091

M20 - M23 0.08663 0.00000 0.08247 0.08657 0.00415

M20 - M24 0.07648 0.00002 0.06567 0.07637 0.01081

M20 - M25 0.15378 0.00002 0.13624 0.15374 0.01753

M20 - M26 0.02435 0.00000 0.02236 0.02436 0.00199

M20 - M27 0.05465 0.00382 0.05318 0.05485 0.00147

M20 - M28 0.06614 0.00382 0.05908 0.07314 0.00706

M20 - M29 0.02722 0.01519 0.02618 0.03635 0.00104

M20 - M30 0.04254 0.00382 0.04097 0.04945 0.00158

M20 - M31 0.04299 0.00382 0.04013 0.04978 0.00287

M20 - M32 0.08422 0.00382 0.07915 0.08949 0.00507

M20 - M33 0.04734 0.00381 0.04534 0.04008 0.00199

M20 - M34 0.09247 0.00000 0.09132 0.09232 0.00115

M20 - M35 0.09158 0.00381 0.08808 0.08441 0.00350

M20 - M36 0.04896 0.01520 0.04798 0.04874 0.00098

M20 - M37 0.07635 0.00381 0.07537 0.07469 0.00098

M20 - M38 0.07635 0.00381 0.07509 0.08134 0.00125

M20 - M39 0.02079 0.00000 0.01961 0.02079 0.00118

M20 - M40 0.04729 0.01520 0.04093 0.04233 0.00635

M20 - M41 0.01734 0.01519 0.01626 0.03105 0.00108

M20 - M42 0.07563 0.00380 0.07288 0.06272 0.00275

M20 - M43 0.12160 0.00381 0.11812 0.11667 0.00348

M20 - M44 0.04361 0.00380 0.04256 0.03620 0.00106

M20 - M45 0.10003 0.00000 0.09870 0.09991 0.00133

M20 - M46 0.01775 0.00000 0.01658 0.01773 0.00117

M20 - M47 0.07122 0.00000 0.06991 0.07123 0.00130

M20 - M48 0.08808 0.00000 0.08586 0.08807 0.00222

M20 - M49 0.09222 0.00000 0.08842 0.09220 0.00380

M20 - M50 0.06700 0.00001 0.06609 0.06700 0.00091

M20 - M51 0.00994 0.00001 0.00942 0.00984 0.00052

M20 - M52 0.02468 0.00001 0.02466 0.02465 0.00001

M20 - M53 0.08809 0.00001 0.08362 0.08820 0.00447

M20 - M54 0.05942 0.00001 0.05940 0.05951 0.00002

M20 - M55 0.03326 0.00000 0.03248 0.03334 0.00078

M20 - M56 0.02939 0.00000 0.02907 0.02938 0.00031

M20 - M57 0.02809 0.00381 0.02724 0.02744 0.00085

M20 - M58 0.02412 0.00000 0.02269 0.02402 0.00142

M20 - M59 0.03439 0.00001 0.03211 0.03419 0.00228

M20 - M60 0.03552 0.00000 0.03423 0.03544 0.00129

M20 - M61 0.04644 0.00001 0.04067 0.04616 0.00577

M20 - M62 0.02900 0.00000 0.02809 0.02899 0.00091

M20 - M63 0.02792 0.00000 0.02701 0.02790 0.00091

M20 - M64 0.03715 0.00382 0.03608 0.04086 0.00107

M20 - M65 0.06305 0.00001 0.05583 0.06325 0.00722

M20 - M66 0.06826 0.00000 0.06691 0.06831 0.00135

M20 - M67 0.06445 0.00001 0.06180 0.06452 0.00265

M20 - M68 0.02560 0.00380 0.02468 0.02785 0.00092

M20 - M69 0.03774 0.00000 0.03609 0.03781 0.00165

M20 - M70 0.04687 0.00001 0.04186 0.04693 0.00501

M20 - M71 0.05277 0.00381 0.05104 0.05244 0.00173

M20 - M72 0.03935 0.00382 0.03692 0.03055 0.00243

M20 - M73 0.05080 0.00380 0.04932 0.03380 0.00147

M20 - M74 0.02929 0.00000 0.02826 0.02929 0.00103

M20 - M75 0.04658 0.00380 0.04323 0.03101 0.00335

M20 - M76 0.11265 0.00380 0.11181 0.10087 0.00084

M20 - M77 0.03473 0.00000 0.03306 0.03476 0.00167

M20 - M78 0.03040 0.00000 0.02805 0.03039 0.00235

M20 - M79 0.04206 0.00000 0.04188 0.04208 0.00017

M20 - M80 0.02754 0.00001 0.02751 0.02739 0.00003

M20 - M81 0.06177 0.00380 0.05658 0.04931 0.00519

M20 - M82 0.07299 0.00380 0.06933 0.07178 0.00366

M20 - M83 0.09228 0.00380 0.09072 0.09216 0.00156

M20 - M84 0.04078 0.00381 0.03958 0.03983 0.00120

M20 - M85 0.05653 0.00380 0.05291 0.03995 0.00362

M20 - M86 0.06420 0.00380 0.05879 0.07721 0.00541

M20 - M87 0.05624 0.00380 0.05285 0.03965 0.00339

M20 - M88 0.07769 0.00381 0.07609 0.08123 0.00160

M20 - M89 0.11656 0.00381 0.11557 0.10956 0.00098

M20 - M90 0.05801 0.00001 0.05475 0.05789 0.00325

M20 - M91 0.02048 0.00000 0.01900 0.02050 0.00148

M20 - M92 0.05551 0.00380 0.05020 0.06388 0.00531

M20 - M93 0.02961 0.00000 0.02859 0.02961 0.00102

M20 - M94 0.04818 0.00000 0.04710 0.04824 0.00109

M20 - M95 0.05337 0.00382 0.05221 0.05642 0.00116

M20 - M96 0.05679 0.00381 0.05537 0.05589 0.00141

M20 - M97 0.06199 0.00380 0.06102 0.06527 0.00098

M21 - M22 0.07931 0.02761 0.07699 0.04657 0.00232

M21 - M23 0.09259 0.02122 0.08962 0.06886 0.00297

M21 - M24 0.07513 0.00913 0.06697 0.06351 0.00816

M21 - M25 0.11273 0.00890 0.10172 0.10528 0.01101

M21 - M26 0.03203 0.00175 0.03033 0.03212 0.00169

M21 - M27 0.04897 0.00822 0.04803 0.04283 0.00094

M21 - M28 0.05839 0.00852 0.05346 0.05338 0.00492

M21 - M29 0.02957 0.00499 0.02936 0.03152 0.00021

M21 - M30 0.06221 0.02096 0.05983 0.03993 0.00238

M21 - M31 0.05914 0.02093 0.05636 0.03978 0.00278

M21 - M32 0.07940 0.02229 0.07306 0.06124 0.00634

M21 - M33 0.05839 0.02339 0.05798 0.03240 0.00041

M21 - M34 0.08726 0.00920 0.08568 0.07107 0.00158

M21 - M35 0.07505 0.01585 0.06971 0.06238 0.00534

M21 - M36 0.03916 0.00655 0.03847 0.03678 0.00069

M21 - M37 0.07181 0.01746 0.06469 0.05495 0.00713

M21 - M38 0.10123 0.03657 0.09591 0.06291 0.00532

M21 - M39 0.03452 0.00169 0.03446 0.02989 0.00006

M21 - M40 0.03732 0.00737 0.03402 0.03655 0.00330

M21 - M41 0.02710 0.00404 0.02610 0.03076 0.00100

M21 - M42 0.04859 0.00758 0.04787 0.04640 0.00071

M21 - M43 0.11244 0.02858 0.10677 0.07607 0.00567

M21 - M44 0.03922 0.01064 0.03857 0.03321 0.00064

M21 - M45 0.09405 0.01093 0.09226 0.07580 0.00179

M21 - M46 0.02877 0.00053 0.02875 0.02810 0.00002

M21 - M47 0.06367 0.00450 0.06284 0.06065 0.00083

M21 - M48 0.07515 0.00472 0.07343 0.07084 0.00172

M21 - M49 0.07762 0.00783 0.07464 0.07253 0.00298

M21 - M50 0.06404 0.00750 0.06343 0.05645 0.00061

M21 - M51 0.03578 0.02718 0.03506 0.01413 0.00072

M21 - M52 0.05004 0.02768 0.04866 0.02157 0.00138

M21 - M53 0.06543 0.00775 0.06301 0.05621 0.00242

M21 - M54 0.05297 0.01217 0.05225 0.03925 0.00072

M21 - M55 0.05710 0.02983 0.05659 0.02743 0.00051

M21 - M56 0.05932 0.03093 0.05893 0.02683 0.00038

M21 - M57 0.03901 0.01487 0.03853 0.02634 0.00048

M21 - M58 0.03346 0.00077 0.03254 0.03243 0.00092

M21 - M59 0.04579 0.00884 0.04326 0.03825 0.00254

M21 - M60 0.04403 0.00679 0.04275 0.03886 0.00128

M21 - M61 0.05257 0.01137 0.04728 0.04521 0.00529

M21 - M62 0.03842 0.00196 0.03780 0.03539 0.00062

M21 - M63 0.03657 0.00202 0.03597 0.03515 0.00060

M21 - M64 0.06463 0.02774 0.05545 0.03601 0.00918

M21 - M65 0.05425 0.00320 0.04936 0.05431 0.00489

M21 - M66 0.06915 0.01358 0.06905 0.05712 0.00009

M21 - M67 0.06296 0.00954 0.06198 0.05381 0.00098

M21 - M68 0.05084 0.02305 0.05031 0.02610 0.00053

M21 - M69 0.04464 0.00598 0.04338 0.03827 0.00127

M21 - M70 0.05208 0.00745 0.04836 0.04538 0.00373

M21 - M71 0.07303 0.02511 0.07079 0.04155 0.00223

M21 - M72 0.05698 0.02161 0.05268 0.02810 0.00430

M21 - M73 0.03150 0.00533 0.02960 0.03227 0.00189

M21 - M74 0.03481 0.00202 0.03456 0.03353 0.00025

M21 - M75 0.03539 0.01264 0.03353 0.02794 0.00186

M21 - M76 0.08277 0.01508 0.08215 0.06658 0.00062

M21 - M77 0.05074 0.02832 0.04958 0.02789 0.00117

M21 - M78 0.03684 0.00482 0.03623 0.03378 0.00062

M21 - M79 0.04362 0.00927 0.04323 0.03329 0.00039

M21 - M80 0.05044 0.02998 0.04975 0.02331 0.00070

M21 - M81 0.03818 0.00571 0.03694 0.03701 0.00124

M21 - M82 0.05907 0.00965 0.05576 0.04943 0.00331

M21 - M83 0.06808 0.00692 0.06632 0.06345 0.00176

M21 - M84 0.04593 0.01503 0.04422 0.03199 0.00171

M21 - M85 0.03881 0.01055 0.03642 0.03486 0.00240

M21 - M86 0.07270 0.02043 0.06303 0.05973 0.00968

M21 - M87 0.03974 0.01049 0.03698 0.03662 0.00277

M21 - M88 0.08249 0.02324 0.07558 0.05805 0.00690

M21 - M89 0.10603 0.02076 0.10549 0.07794 0.00055

M21 - M90 0.05848 0.00568 0.05617 0.05093 0.00231

M21 - M91 0.04070 0.01089 0.03958 0.02951 0.00112

M21 - M92 0.07264 0.02524 0.06512 0.04868 0.00752

M21 - M93 0.03667 0.00282 0.03578 0.03555 0.00089

M21 - M94 0.05461 0.00780 0.05009 0.04371 0.00452

M21 - M95 0.06964 0.02420 0.06751 0.04273 0.00213

M21 - M96 0.06866 0.02109 0.06572 0.04253 0.00295

M21 - M97 0.06641 0.01850 0.06464 0.04834 0.00177

M22 - M23 0.14876 0.05991 0.07486 0.09335 0.07389

M22 - M24 0.10263 0.00986 0.09514 0.09894 0.00749

M22 - M25 0.15514 0.00895 0.14328 0.14823 0.01185

M22 - M26 0.06623 0.00890 0.05872 0.05334 0.00751

M22 - M27 0.06898 0.00420 0.06637 0.06052 0.00261

M22 - M28 0.08109 0.00216 0.07553 0.08022 0.00555

M22 - M29 0.05715 0.00208 0.05671 0.05770 0.00044

M22 - M30 0.07212 0.00225 0.06972 0.06594 0.00239

M22 - M31 0.07755 0.00871 0.07416 0.06283 0.00339

M22 - M32 0.10766 0.01124 0.10189 0.08489 0.00576

M22 - M33 0.05193 0.00194 0.05149 0.04696 0.00044

M22 - M34 0.10516 0.03218 0.07202 0.09344 0.03314

M22 - M35 0.08824 0.01193 0.08644 0.07246 0.00180

M22 - M36 0.06287 0.00380 0.06245 0.05556 0.00042

M22 - M37 0.08326 0.01129 0.08106 0.07108 0.00220

M22 - M38 0.08575 0.01142 0.08290 0.07976 0.00285

M22 - M39 0.04294 0.00170 0.04143 0.04898 0.00151

M22 - M40 0.05383 0.00234 0.05196 0.05174 0.00187

M22 - M41 0.05280 0.00014 0.05177 0.05282 0.00103

M22 - M42 0.06458 0.00759 0.05791 0.06385 0.00667

M22 - M43 0.11714 0.02033 0.09507 0.09962 0.02206

M22 - M44 0.05410 0.01036 0.04804 0.04394 0.00606

M22 - M45 0.11641 0.04056 0.07697 0.09935 0.03944

M22 - M46 0.04628 0.00123 0.04495 0.04861 0.00133

M22 - M47 0.10345 0.00294 0.09959 0.10081 0.00387

M22 - M48 0.11969 0.00182 0.11403 0.11795 0.00566

M22 - M49 0.12846 0.00514 0.11301 0.11658 0.01545

M22 - M50 0.10073 0.01390 0.10001 0.08527 0.00072

M22 - M51 0.04892 0.01086 0.04827 0.03328 0.00065

M22 - M52 0.04937 0.00910 0.04735 0.04112 0.00202

M22 - M53 0.10983 0.00809 0.10601 0.09918 0.00382

M22 - M54 0.08951 0.01152 0.08822 0.07816 0.00129

M22 - M55 0.06101 0.01066 0.05840 0.04961 0.00262

M22 - M56 0.06539 0.00884 0.06359 0.04781 0.00180

M22 - M57 0.04787 0.00778 0.04734 0.04118 0.00053

M22 - M58 0.05310 0.00126 0.05108 0.05266 0.00203

M22 - M59 0.07549 0.01039 0.07364 0.05989 0.00185

M22 - M60 0.07817 0.00998 0.07706 0.05867 0.00111

M22 - M61 0.07659 0.00704 0.07125 0.06822 0.00534

M22 - M62 0.05377 0.00126 0.05306 0.05370 0.00071

M22 - M63 0.05367 0.00123 0.05287 0.05341 0.00080

M22 - M64 0.07124 0.01734 0.07085 0.05420 0.00039

M22 - M65 0.08279 0.00521 0.07637 0.07965 0.00641

M22 - M66 0.08754 0.00998 0.08436 0.08391 0.00317

M22 - M67 0.09235 0.01112 0.09032 0.08334 0.00203

M22 - M68 0.05232 0.00739 0.04947 0.04574 0.00285

M22 - M69 0.08959 0.01302 0.08793 0.06408 0.00165

M22 - M70 0.08299 0.00633 0.07279 0.07201 0.01020

M22 - M71 0.06605 0.00775 0.06035 0.05735 0.00570

M22 - M72 0.06263 0.01252 0.06204 0.04220 0.00059

M22 - M73 0.05217 0.00091 0.05135 0.05553 0.00082

M22 - M74 0.05647 0.00318 0.05409 0.05698 0.00237

M22 - M75 0.05068 0.00987 0.04907 0.04847 0.00160

M22 - M76 0.11173 0.03744 0.08407 0.09121 0.02766

M22 - M77 0.06323 0.00609 0.06214 0.05930 0.00108

M22 - M78 0.06477 0.00151 0.06390 0.05940 0.00087

M22 - M79 0.07125 0.00756 0.06842 0.06903 0.00283

M22 - M80 0.04872 0.00285 0.04745 0.04829 0.00128

M22 - M81 0.05919 0.00322 0.05458 0.05736 0.00461

M22 - M82 0.07612 0.00375 0.07149 0.07255 0.00463

M22 - M83 0.08619 0.00317 0.08328 0.08225 0.00292

M22 - M84 0.05261 0.00514 0.04886 0.04752 0.00376

M22 - M85 0.05842 0.00442 0.05736 0.05830 0.00106

M22 - M86 0.09659 0.00392 0.09132 0.09405 0.00528

M22 - M87 0.05589 0.00504 0.05446 0.05742 0.00143

M22 - M88 0.10156 0.02156 0.09891 0.08618 0.00265

M22 - M89 0.09722 0.00504 0.09631 0.09265 0.00091

M22 - M90 0.09344 0.01114 0.08677 0.07928 0.00668

M22 - M91 0.05958 0.01166 0.05198 0.05038 0.00760

M22 - M92 0.07959 0.00820 0.07411 0.07346 0.00548

M22 - M93 0.05354 0.00175 0.05084 0.05492 0.00270

M22 - M94 0.08225 0.01113 0.08088 0.06764 0.00137

M22 - M95 0.07129 0.00718 0.07092 0.06110 0.00037

M22 - M96 0.06680 0.00948 0.06636 0.05848 0.00044

M22 - M97 0.07799 0.00473 0.07517 0.06984 0.00282

M23 - M24 0.12709 0.01142 0.11577 0.11859 0.01131

M23 - M25 0.17735 0.00743 0.16053 0.16807 0.01682

M23 - M26 0.11292 0.02167 0.08049 0.08917 0.03243

M23 - M27 0.08257 0.00289 0.07685 0.07959 0.00572

M23 - M28 0.09874 0.00128 0.09122 0.09969 0.00752

M23 - M29 0.08391 0.00073 0.08057 0.08321 0.00333

M23 - M30 0.09176 0.00196 0.08799 0.08679 0.00377

M23 - M31 0.09053 0.00423 0.08572 0.08372 0.00481

M23 - M32 0.12093 0.00804 0.11372 0.10398 0.00721

M23 - M33 0.07922 0.00397 0.07632 0.07230 0.00290

M23 - M34 0.14455 0.03941 0.08766 0.13003 0.05688

M23 - M35 0.11263 0.01175 0.10334 0.10197 0.00928

M23 - M36 0.07830 0.00148 0.07512 0.07294 0.00318

M23 - M37 0.10531 0.00991 0.09569 0.09549 0.00962

M23 - M38 0.12062 0.01128 0.10883 0.11370 0.01179

M23 - M39 0.07443 0.00173 0.06634 0.08015 0.00810

M23 - M40 0.07137 0.00174 0.06509 0.07411 0.00628

M23 - M41 0.08457 0.00006 0.08065 0.08439 0.00392

M23 - M42 0.09215 0.00955 0.07801 0.09066 0.01415

M23 - M43 0.14208 0.02241 0.10837 0.12438 0.03372

M23 - M44 0.06969 0.00857 0.05758 0.06648 0.01211

M23 - M45 0.15727 0.04902 0.09114 0.13615 0.06613

M23 - M46 0.07547 0.00074 0.06930 0.07883 0.00617

M23 - M47 0.12230 0.00213 0.11675 0.11989 0.00555

M23 - M48 0.14323 0.00341 0.13540 0.13858 0.00783

M23 - M49 0.14871 0.00673 0.13058 0.13527 0.01813

M23 - M50 0.11638 0.01260 0.11288 0.10602 0.00350

M23 - M51 0.05680 0.00453 0.05357 0.05227 0.00323

M23 - M52 0.07045 0.00903 0.06590 0.05927 0.00455

M23 - M53 0.13352 0.00715 0.12748 0.12443 0.00604

M23 - M54 0.10713 0.00928 0.10409 0.09592 0.00305

M23 - M55 0.07885 0.00781 0.07559 0.06954 0.00326

M23 - M56 0.09079 0.00797 0.08774 0.07396 0.00304

M23 - M57 0.06291 0.00527 0.05963 0.05832 0.00328

M23 - M58 0.07831 0.00046 0.07198 0.07733 0.00634

M23 - M59 0.09046 0.00840 0.08590 0.07946 0.00456

M23 - M60 0.10101 0.00916 0.09668 0.08115 0.00433

M23 - M61 0.09542 0.00567 0.08563 0.08811 0.00979

M23 - M62 0.07830 0.00112 0.07424 0.07739 0.00406

M23 - M63 0.07821 0.00107 0.07394 0.07716 0.00427

M23 - M64 0.09052 0.01516 0.08672 0.07406 0.00380

M23 - M65 0.10038 0.00519 0.09078 0.09768 0.00960

M23 - M66 0.11302 0.00984 0.10626 0.10979 0.00677

M23 - M67 0.10502 0.00931 0.09833 0.09977 0.00669

M23 - M68 0.07021 0.00786 0.06636 0.06236 0.00385

M23 - M69 0.10523 0.01246 0.10121 0.08329 0.00402

M23 - M70 0.10018 0.00509 0.08702 0.09168 0.01316

M23 - M71 0.09017 0.00624 0.08056 0.07949 0.00961

M23 - M72 0.07886 0.01013 0.07573 0.06034 0.00313

M23 - M73 0.07806 0.00146 0.07205 0.08347 0.00601

M23 - M74 0.08220 0.00204 0.07610 0.08350 0.00610

M23 - M75 0.07519 0.00949 0.06937 0.07513 0.00582

M23 - M76 0.14367 0.04315 0.09640 0.12027 0.04727

M23 - M77 0.08376 0.00441 0.07987 0.08154 0.00389

M23 - M78 0.09079 0.00158 0.08563 0.08535 0.00516

M23 - M79 0.09350 0.00558 0.08294 0.09453 0.01055

M23 - M80 0.07087 0.00315 0.06665 0.06871 0.00423

M23 - M81 0.08231 0.00298 0.07749 0.07911 0.00482

M23 - M82 0.09913 0.00576 0.09061 0.09199 0.00852

M23 - M83 0.11403 0.00344 0.10930 0.11039 0.00472

M23 - M84 0.07040 0.00551 0.06264 0.06381 0.00776

M23 - M85 0.08210 0.00407 0.07698 0.08390 0.00512

M23 - M86 0.12155 0.00282 0.11508 0.12280 0.00647

M23 - M87 0.08297 0.00471 0.07662 0.08570 0.00635

M23 - M88 0.12393 0.02400 0.11545 0.10515 0.00848

M23 - M89 0.12900 0.00644 0.12338 0.12823 0.00563

M23 - M90 0.10801 0.00640 0.09765 0.09864 0.01036

M23 - M91 0.07906 0.01035 0.06768 0.07179 0.01138

M23 - M92 0.10483 0.00780 0.09702 0.09675 0.00782

M23 - M93 0.08089 0.00162 0.07262 0.08284 0.00827

M23 - M94 0.10080 0.00878 0.09714 0.08696 0.00365

M23 - M95 0.08583 0.00362 0.08294 0.08021 0.00289

M23 - M96 0.08466 0.00562 0.08126 0.08018 0.00341

M23 - M97 0.09455 0.00460 0.08867 0.08887 0.00588

M24 - M25 0.28018 0.11914 0.13230 0.15095 0.14788

M24 - M26 0.09851 0.00484 0.07515 0.09134 0.02336

M24 - M27 0.07968 0.00619 0.07176 0.07459 0.00792

M24 - M28 0.08470 0.00560 0.07143 0.08522 0.01328

M24 - M29 0.07840 0.00174 0.06679 0.07265 0.01161

M24 - M30 0.08428 0.01265 0.07610 0.07418 0.00818

M24 - M31 0.07811 0.00537 0.06831 0.07328 0.00980

M24 - M32 0.10088 0.00854 0.08975 0.09219 0.01114

M24 - M33 0.07598 0.00776 0.06782 0.06626 0.00816

M24 - M34 0.12513 0.00364 0.11679 0.12365 0.00834

M24 - M35 0.09354 0.00143 0.08584 0.09303 0.00769

M24 - M36 0.08421 0.00523 0.07050 0.07361 0.01372

M24 - M37 0.08901 0.00314 0.08134 0.08556 0.00767

M24 - M38 0.09504 0.00179 0.08640 0.09033 0.00864

M24 - M39 0.08972 0.00098 0.07702 0.09087 0.01270

M24 - M40 0.07213 0.00846 0.06315 0.07011 0.00898

M24 - M41 0.07318 0.00033 0.06287 0.07265 0.01031

M24 - M42 0.08465 0.00463 0.07710 0.08317 0.00755

M24 - M43 0.12321 0.00855 0.11283 0.11248 0.01038

M24 - M44 0.06813 0.00821 0.06046 0.06455 0.00767

M24 - M45 0.12981 0.00528 0.12147 0.12733 0.00833

M24 - M46 0.09227 0.00225 0.08125 0.09007 0.01102

M24 - M47 0.11705 0.00688 0.10911 0.10919 0.00794

M24 - M48 0.12729 0.00475 0.11851 0.11997 0.00878

M24 - M49 0.12910 0.00636 0.11922 0.12121 0.00988

M24 - M50 0.11832 0.01221 0.11108 0.10481 0.00725

M24 - M51 0.06411 0.01189 0.05668 0.05162 0.00743

M24 - M52 0.07096 0.01165 0.06386 0.05851 0.00710

M24 - M53 0.11671 0.00436 0.10169 0.11329 0.01502

M24 - M54 0.10467 0.01074 0.09757 0.09632 0.00710

M24 - M55 0.08028 0.01140 0.06815 0.06590 0.01214

M24 - M56 0.07675 0.00484 0.06690 0.06751 0.00986

M24 - M57 0.06308 0.00576 0.05599 0.05880 0.00708

M24 - M58 0.09033 0.00234 0.07798 0.08747 0.01235

M24 - M59 0.10037 0.01107 0.08653 0.08619 0.01384

M24 - M60 0.10208 0.00543 0.08741 0.09025 0.01467

M24 - M61 0.10902 0.01249 0.08988 0.09287 0.01914

M24 - M62 0.10137 0.00871 0.09370 0.09109 0.00766

M24 - M63 0.10010 0.00713 0.09197 0.09072 0.00813

M24 - M64 0.07504 0.00462 0.06759 0.06916 0.00745

M24 - M65 0.10975 0.00370 0.08750 0.10338 0.02225

M24 - M66 0.12051 0.01331 0.11014 0.10617 0.01037

M24 - M67 0.11594 0.01430 0.10836 0.10175 0.00757

M24 - M68 0.06477 0.00433 0.05689 0.06005 0.00788

M24 - M69 0.09136 0.00358 0.08037 0.09045 0.01099

M24 - M70 0.09987 0.00593 0.08612 0.09344 0.01375

M24 - M71 0.07634 0.00252 0.06804 0.07397 0.00830

M24 - M72 0.06594 0.00416 0.05812 0.06149 0.00782

M24 - M73 0.07220 0.00054 0.06254 0.07264 0.00967

M24 - M74 0.09707 0.00179 0.07913 0.08977 0.01795

M24 - M75 0.06662 0.00358 0.05679 0.06854 0.00982

M24 - M76 0.10771 0.00624 0.10032 0.10198 0.00739

M24 - M77 0.06542 0.00514 0.05715 0.06808 0.00827

M24 - M78 0.09775 0.00248 0.08220 0.09181 0.01554

M24 - M79 0.09629 0.00286 0.08505 0.09597 0.01124

M24 - M80 0.06605 0.01096 0.05710 0.06029 0.00895

M24 - M81 0.08118 0.00276 0.06964 0.07370 0.01154

M24 - M82 0.09524 0.01525 0.08581 0.08170 0.00943

M24 - M83 0.09738 0.00548 0.08949 0.09486 0.00789

M24 - M84 0.08081 0.01620 0.07071 0.06430 0.01010

M24 - M85 0.08087 0.00430 0.06825 0.07603 0.01262

M24 - M86 0.10225 0.00234 0.08991 0.09789 0.01233

M24 - M87 0.08365 0.00402 0.07182 0.07678 0.01183

M24 - M88 0.09347 0.00583 0.08437 0.09032 0.00910

M24 - M89 0.10759 0.00395 0.09941 0.10728 0.00818

M24 - M90 0.10511 0.00830 0.09329 0.09945 0.01183

M24 - M91 0.08628 0.00648 0.07584 0.08491 0.01044

M24 - M92 0.08955 0.00740 0.07794 0.08362 0.01161

M24 - M93 0.09637 0.00169 0.08100 0.09432 0.01536

M24 - M94 0.09567 0.00434 0.08260 0.09533 0.01307

M24 - M95 0.08203 0.00485 0.07366 0.07581 0.00837

M24 - M96 0.07916 0.00407 0.07198 0.07560 0.00718

M24 - M97 0.08677 0.00479 0.07871 0.08246 0.00806

M25 - M26 0.17014 0.00523 0.13614 0.16206 0.03399

M25 - M27 0.12141 0.00454 0.10921 0.11578 0.01221

M25 - M28 0.12730 0.00424 0.10932 0.12913 0.01798

M25 - M29 0.14633 0.00131 0.12827 0.14315 0.01806

M25 - M30 0.12155 0.00719 0.10887 0.11780 0.01269

M25 - M31 0.12104 0.00669 0.10626 0.11519 0.01478

M25 - M32 0.14125 0.00778 0.12037 0.13676 0.02088

M25 - M33 0.11387 0.00638 0.10240 0.10527 0.01147

M25 - M34 0.18874 0.00436 0.17509 0.18008 0.01365

M25 - M35 0.14002 0.00343 0.12812 0.13814 0.01191

M25 - M36 0.14051 0.00393 0.12188 0.13261 0.01863

M25 - M37 0.13488 0.00425 0.12247 0.13127 0.01241

M25 - M38 0.13948 0.00416 0.12707 0.13102 0.01240

M25 - M39 0.16188 0.00065 0.14294 0.16168 0.01893

M25 - M40 0.12342 0.00453 0.10814 0.12556 0.01527

M25 - M41 0.15034 0.00033 0.13349 0.14974 0.01685

M25 - M42 0.14740 0.00505 0.13578 0.13920 0.01163

M25 - M43 0.16600 0.00401 0.15235 0.16281 0.01365

M25 - M44 0.11088 0.00461 0.09841 0.10710 0.01247

M25 - M45 0.18760 0.00401 0.17441 0.18121 0.01320

M25 - M46 0.16614 0.00172 0.14570 0.16248 0.02044

M25 - M47 0.16593 0.00719 0.15339 0.15849 0.01254

M25 - M48 0.18162 0.00503 0.16804 0.17549 0.01359

M25 - M49 0.18012 0.00618 0.16564 0.17592 0.01449

M25 - M50 0.15510 0.01003 0.14400 0.14550 0.01111

M25 - M51 0.10277 0.01093 0.09118 0.09097 0.01160

M25 - M52 0.10890 0.00980 0.09775 0.09907 0.01115

M25 - M53 0.16354 0.00551 0.14656 0.15733 0.01698

M25 - M54 0.14922 0.01131 0.13811 0.13824 0.01111

M25 - M55 0.12028 0.00912 0.10737 0.11164 0.01291

M25 - M56 0.11539 0.00408 0.10413 0.11433 0.01127

M25 - M57 0.10422 0.00477 0.09248 0.09957 0.01174

M25 - M58 0.15467 0.00180 0.13313 0.15222 0.02154

M25 - M59 0.14135 0.00829 0.12007 0.13203 0.02129

M25 - M60 0.14778 0.00494 0.12940 0.14271 0.01838

M25 - M61 0.16137 0.01204 0.12895 0.14491 0.03243

M25 - M62 0.16622 0.00643 0.14995 0.15501 0.01626

M25 - M63 0.16760 0.00642 0.15121 0.15581 0.01639

M25 - M64 0.11862 0.00333 0.10640 0.11610 0.01222

M25 - M65 0.16862 0.00290 0.13584 0.16145 0.03278

M25 - M66 0.15484 0.00695 0.14107 0.14656 0.01376

M25 - M67 0.15802 0.01235 0.14671 0.14712 0.01131

M25 - M68 0.11809 0.00610 0.10657 0.10667 0.01152

M25 - M69 0.13201 0.00332 0.11831 0.13632 0.01371

M25 - M70 0.14795 0.00589 0.12720 0.13658 0.02075

M25 - M71 0.11327 0.00124 0.10101 0.11432 0.01226

M25 - M72 0.10591 0.00167 0.09416 0.10624 0.01175

M25 - M73 0.14756 0.00045 0.13201 0.14792 0.01554

M25 - M74 0.16781 0.00221 0.13930 0.15736 0.02851

M25 - M75 0.13680 0.00343 0.12185 0.13784 0.01496

M25 - M76 0.16423 0.00530 0.15294 0.15607 0.01128

M25 - M77 0.10690 0.00312 0.09537 0.11250 0.01153

M25 - M78 0.15151 0.00242 0.13461 0.15070 0.01689

M25 - M79 0.14885 0.00192 0.13249 0.15021 0.01635

M25 - M80 0.10039 0.00704 0.08957 0.09940 0.01082

M25 - M81 0.13301 0.00300 0.11795 0.12729 0.01506

M25 - M82 0.13173 0.00958 0.11854 0.12526 0.01320

M25 - M83 0.13989 0.00479 0.12777 0.13958 0.01212

M25 - M84 0.11734 0.01209 0.10379 0.10465 0.01355

M25 - M85 0.15724 0.00410 0.13852 0.15301 0.01872

M25 - M86 0.17860 0.00207 0.16153 0.17496 0.01708

M25 - M87 0.16071 0.00413 0.14249 0.15226 0.01822

M25 - M88 0.15218 0.00583 0.13002 0.14394 0.02216

M25 - M89 0.15764 0.00510 0.14496 0.15789 0.01268

M25 - M90 0.14723 0.00231 0.12875 0.14657 0.01848

M25 - M91 0.15612 0.00415 0.13787 0.15499 0.01825

M25 - M92 0.14511 0.00612 0.12838 0.14493 0.01673

M25 - M93 0.17671 0.00148 0.15172 0.17156 0.02499

M25 - M94 0.14944 0.00309 0.12821 0.15014 0.02124

M25 - M95 0.12965 0.00398 0.11733 0.12550 0.01232

M25 - M96 0.12419 0.00364 0.11288 0.12072 0.01131

M25 - M97 0.13375 0.00372 0.12155 0.12778 0.01220

M26 - M27 0.04970 0.00029 0.04678 0.05029 0.00292

M26 - M28 0.06544 0.00018 0.05678 0.06640 0.00866

M26 - M29 0.02882 0.00018 0.02689 0.02884 0.00192

M26 - M30 0.04823 0.00063 0.04514 0.04806 0.00310

M26 - M31 0.04792 0.00036 0.04272 0.04718 0.00520

M26 - M32 0.08308 0.00031 0.07438 0.08322 0.00870

M26 - M33 0.03953 0.00188 0.03791 0.03618 0.00162

M26 - M34 0.08433 0.00027 0.08094 0.08415 0.00339

M26 - M35 0.07716 0.00125 0.07523 0.07600 0.00193

M26 - M36 0.04058 0.00037 0.03917 0.03962 0.00141

M26 - M37 0.06788 0.00108 0.06529 0.06879 0.00259

M26 - M38 0.08162 0.00138 0.07953 0.07811 0.00209

M26 - M39 0.01799 0.00011 0.01585 0.01755 0.00214

M26 - M40 0.03515 0.00081 0.02844 0.03723 0.00672

M26 - M41 0.02380 0.00001 0.02174 0.02367 0.00207

M26 - M42 0.05043 0.00038 0.04874 0.05060 0.00169

M26 - M43 0.10010 0.00063 0.09475 0.10239 0.00535

M26 - M44 0.02896 0.00081 0.02534 0.03131 0.00362

M26 - M45 0.09205 0.00043 0.08836 0.09103 0.00368

M26 - M46 0.01645 0.00067 0.01387 0.01458 0.00258

M26 - M47 0.09429 0.00153 0.09178 0.09313 0.00251

M26 - M48 0.11706 0.00224 0.11362 0.11181 0.00344

M26 - M49 0.11774 0.00205 0.11228 0.11328 0.00546

M26 - M50 0.07754 0.00130 0.07613 0.07703 0.00141

M26 - M51 0.01789 0.00220 0.01629 0.01510 0.00160

M26 - M52 0.03050 0.00202 0.02918 0.02882 0.00132

M26 - M53 0.09366 0.00105 0.08871 0.09054 0.00494

M26 - M54 0.06247 0.00082 0.06105 0.06204 0.00142

M26 - M55 0.03898 0.00134 0.03663 0.03587 0.00235

M26 - M56 0.03866 0.00172 0.03689 0.03535 0.00176

M26 - M57 0.02673 0.00138 0.02422 0.02623 0.00252

M26 - M58 0.02702 0.00077 0.02226 0.02703 0.00475

M26 - M59 0.04518 0.00101 0.04123 0.04442 0.00395

M26 - M60 0.03913 0.00132 0.03418 0.03931 0.00495

M26 - M61 0.05746 0.00170 0.04545 0.05686 0.01200

M26 - M62 0.03524 0.00102 0.03209 0.03090 0.00315

M26 - M63 0.03345 0.00097 0.03019 0.02931 0.00326

M26 - M64 0.04330 0.00116 0.04173 0.04158 0.00157

M26 - M65 0.06986 0.00071 0.05672 0.06892 0.01314

M26 - M66 0.07257 0.00153 0.07098 0.07411 0.00159

M26 - M67 0.07502 0.00148 0.07337 0.07474 0.00164

M26 - M68 0.03011 0.00185 0.02797 0.02758 0.00214

M26 - M69 0.04314 0.00123 0.03858 0.04412 0.00456

M26 - M70 0.06034 0.00072 0.05210 0.05791 0.00824

M26 - M71 0.05234 0.00142 0.04867 0.04835 0.00367

M26 - M72 0.02486 0.00051 0.02316 0.02198 0.00170

M26 - M73 0.02691 0.00009 0.02507 0.02630 0.00184

M26 - M74 0.02667 0.00083 0.02402 0.02584 0.00266

M26 - M75 0.02665 0.00056 0.02471 0.02503 0.00194

M26 - M76 0.08770 0.00088 0.08586 0.08502 0.00184

M26 - M77 0.03694 0.00063 0.03469 0.03646 0.00225

M26 - M78 0.02451 0.00070 0.02267 0.02349 0.00184

M26 - M79 0.04568 0.00186 0.04360 0.04779 0.00208

M26 - M80 0.03190 0.00030 0.03042 0.03191 0.00148

M26 - M81 0.03394 0.00102 0.03137 0.03809 0.00257

M26 - M82 0.06415 0.00062 0.05839 0.06413 0.00576

M26 - M83 0.08997 0.00224 0.08766 0.08693 0.00231

M26 - M84 0.04447 0.00154 0.04124 0.03753 0.00322

M26 - M85 0.03140 0.00008 0.02949 0.03113 0.00191

M26 - M86 0.07882 0.00008 0.07288 0.07799 0.00594

M26 - M87 0.03075 0.00009 0.02893 0.02993 0.00183

M26 - M88 0.07321 0.00124 0.07094 0.07166 0.00226

M26 - M89 0.10002 0.00115 0.09873 0.09652 0.00129

M26 - M90 0.07009 0.00170 0.06358 0.06853 0.00650

M26 - M91 0.02895 0.00137 0.02388 0.02498 0.00508

M26 - M92 0.06372 0.00045 0.05639 0.06244 0.00733

M26 - M93 0.03164 0.00043 0.02781 0.03011 0.00383

M26 - M94 0.05434 0.00043 0.05071 0.05324 0.00364

M26 - M95 0.05590 0.00039 0.05446 0.05598 0.00144

M26 - M96 0.05562 0.00083 0.05427 0.05356 0.00135

M26 - M97 0.06812 0.00150 0.06595 0.06309 0.00218

M27 - M28 0.11580 0.04158 0.10653 0.06556 0.00927

M27 - M29 0.05984 0.00738 0.05871 0.05186 0.00113

M27 - M30 0.06566 0.01524 0.06341 0.05492 0.00225

M27 - M31 0.05700 0.00692 0.05329 0.05343 0.00371

M27 - M32 0.07529 0.00722 0.07027 0.07371 0.00502

M27 - M33 0.06261 0.01739 0.05517 0.04301 0.00745

M27 - M34 0.08953 0.00043 0.08564 0.08768 0.00389

M27 - M35 0.10272 0.02138 0.09784 0.07188 0.00488

M27 - M36 0.05633 0.00785 0.05474 0.05133 0.00159

M27 - M37 0.08874 0.01892 0.08347 0.06513 0.00528

M27 - M38 0.07796 0.00834 0.07460 0.07138 0.00336

M27 - M39 0.05181 0.00201 0.04833 0.05062 0.00349

M27 - M40 0.05104 0.00972 0.04838 0.04967 0.00266

M27 - M41 0.04910 0.00411 0.04767 0.05152 0.00143

M27 - M42 0.08698 0.01998 0.08448 0.06309 0.00250

M27 - M43 0.09130 0.00572 0.08806 0.08827 0.00324

M27 - M44 0.06662 0.01863 0.05986 0.04482 0.00676

M27 - M45 0.09435 0.00097 0.08961 0.09178 0.00474

M27 - M46 0.04885 0.00212 0.04746 0.04796 0.00139

M27 - M47 0.07700 0.00068 0.07481 0.07497 0.00219

M27 - M48 0.09114 0.00204 0.08823 0.08734 0.00291

M27 - M49 0.09087 0.00280 0.08622 0.08737 0.00464

M27 - M50 0.07252 0.00484 0.07143 0.06598 0.00109

M27 - M51 0.06105 0.02963 0.05544 0.02738 0.00561

M27 - M52 0.06169 0.02799 0.06015 0.03436 0.00153

M27 - M53 0.08088 0.00749 0.07812 0.06759 0.00276

M27 - M54 0.05563 0.00609 0.05461 0.05070 0.00101

M27 - M55 0.06837 0.02633 0.06437 0.04036 0.00400

M27 - M56 0.06286 0.02175 0.06188 0.04341 0.00098

M27 - M57 0.06253 0.02329 0.05902 0.03645 0.00351

M27 - M58 0.05297 0.00249 0.05052 0.04861 0.00245

M27 - M59 0.05998 0.00749 0.05475 0.04789 0.00522

M27 - M60 0.06093 0.00444 0.05879 0.05179 0.00215

M27 - M61 0.06138 0.00558 0.05411 0.05489 0.00727

M27 - M62 0.05083 0.00226 0.04854 0.05183 0.00228

M27 - M63 0.05260 0.00332 0.04990 0.05173 0.00270

M27 - M64 0.05499 0.01165 0.05394 0.05007 0.00105

M27 - M65 0.07363 0.00365 0.06768 0.06844 0.00595

M27 - M66 0.07765 0.01185 0.07646 0.06699 0.00119

M27 - M67 0.07686 0.00978 0.07476 0.06457 0.00210

M27 - M68 0.04174 0.00625 0.04044 0.03786 0.00131

M27 - M69 0.05595 0.00348 0.05338 0.05183 0.00257

M27 - M70 0.06150 0.00423 0.05604 0.05578 0.00546

M27 - M71 0.05669 0.00679 0.05400 0.05116 0.00268

M27 - M72 0.04741 0.01012 0.04615 0.03934 0.00126

M27 - M73 0.07774 0.01572 0.07183 0.05422 0.00590

M27 - M74 0.05446 0.00099 0.05190 0.05159 0.00256

M27 - M75 0.06937 0.01620 0.06634 0.04585 0.00302

M27 - M76 0.10598 0.01628 0.10132 0.08240 0.00466

M27 - M77 0.06878 0.02373 0.06554 0.04471 0.00324

M27 - M78 0.05910 0.00465 0.05300 0.05116 0.00610

M27 - M79 0.05783 0.00614 0.05378 0.04901 0.00405

M27 - M80 0.06281 0.02630 0.06174 0.03665 0.00107

M27 - M81 0.07318 0.02202 0.07075 0.04967 0.00243

M27 - M82 0.08191 0.02111 0.07761 0.05973 0.00430

M27 - M83 0.09072 0.01902 0.08857 0.07203 0.00215

M27 - M84 0.06826 0.02628 0.06225 0.04271 0.00601

M27 - M85 0.08365 0.01753 0.08041 0.05434 0.00324

M27 - M86 0.08632 0.00525 0.08169 0.07900 0.00462

M27 - M87 0.08156 0.01670 0.07764 0.05511 0.00392

M27 - M88 0.07474 0.00679 0.07083 0.06951 0.00391

M27 - M89 0.09405 0.00688 0.09290 0.08663 0.00115

M27 - M90 0.06477 0.00368 0.06079 0.06283 0.00398

M27 - M91 0.05388 0.00608 0.05144 0.04647 0.00245

M27 - M92 0.08422 0.01439 0.07984 0.06427 0.00438

M27 - M93 0.06209 0.00597 0.05824 0.05521 0.00385

M27 - M94 0.05549 0.00155 0.05417 0.05576 0.00132

M27 - M95 0.07407 0.01283 0.07284 0.05610 0.00123

M27 - M96 0.06485 0.01377 0.06361 0.05323 0.00124

M27 - M97 0.07306 0.00862 0.07091 0.06141 0.00215

M28 - M29 0.06484 0.00940 0.05887 0.07068 0.00596

M28 - M30 0.09104 0.01991 0.08041 0.07089 0.01063

M28 - M31 0.09028 0.01950 0.07978 0.06949 0.01050

M28 - M32 0.11436 0.02087 0.10077 0.08925 0.01358

M28 - M33 0.06765 0.01238 0.06212 0.05911 0.00553

M28 - M34 0.11544 0.00177 0.10848 0.11346 0.00697

M28 - M35 0.11581 0.02007 0.10776 0.08241 0.00805

M28 - M36 0.06781 0.00580 0.06193 0.06978 0.00588

M28 - M37 0.10516 0.02186 0.09969 0.07839 0.00547

M28 - M38 0.09625 0.00644 0.09041 0.08668 0.00584

M28 - M39 0.07077 0.00070 0.06085 0.07175 0.00992

M28 - M40 0.06175 0.00778 0.05546 0.06272 0.00629

M28 - M41 0.06103 0.00418 0.05504 0.06776 0.00600

M28 - M42 0.11135 0.02077 0.10297 0.08161 0.00838

M28 - M43 0.10756 0.00646 0.10018 0.10585 0.00738

M28 - M44 0.08837 0.02024 0.07655 0.05909 0.01182

M28 - M45 0.12013 0.00090 0.11299 0.11803 0.00713

M28 - M46 0.06656 0.00070 0.05729 0.06808 0.00926

M28 - M47 0.08762 0.00268 0.08157 0.08597 0.00605

M28 - M48 0.09841 0.00330 0.09164 0.10019 0.00677

M28 - M49 0.09760 0.00394 0.08970 0.10016 0.00790

M28 - M50 0.08246 0.00317 0.07699 0.07736 0.00547

M28 - M51 0.07395 0.03089 0.06737 0.03494 0.00659

M28 - M52 0.06733 0.02410 0.06353 0.04295 0.00380

M28 - M53 0.07721 0.00397 0.07144 0.07485 0.00577

M28 - M54 0.06394 0.00550 0.06059 0.05992 0.00335

M28 - M55 0.07096 0.02594 0.06724 0.04777 0.00372

M28 - M56 0.06228 0.02560 0.05852 0.05155 0.00377

M28 - M57 0.07043 0.02210 0.06477 0.04885 0.00566

M28 - M58 0.07080 0.00137 0.06079 0.06942 0.01001

M28 - M59 0.07322 0.00795 0.06217 0.06239 0.01105

M28 - M60 0.07370 0.00263 0.06614 0.07313 0.00756

M28 - M61 0.08039 0.00753 0.06913 0.06867 0.01126

M28 - M62 0.07091 0.00099 0.06051 0.07062 0.01040

M28 - M63 0.07063 0.00086 0.06070 0.07058 0.00993

M28 - M64 0.06431 0.00771 0.05857 0.06045 0.00574

M28 - M65 0.08773 0.00254 0.07620 0.08382 0.01153

M28 - M66 0.08684 0.00930 0.08187 0.08014 0.00497

M28 - M67 0.08604 0.00825 0.08084 0.07647 0.00519

M28 - M68 0.05783 0.00706 0.05224 0.04928 0.00559

M28 - M69 0.06767 0.00360 0.06064 0.06732 0.00703

M28 - M70 0.08213 0.01026 0.07335 0.06815 0.00877

M28 - M71 0.06836 0.00812 0.06232 0.06289 0.00604

M28 - M72 0.05694 0.00775 0.05099 0.05224 0.00596

M28 - M73 0.10791 0.01592 0.09741 0.07316 0.01050

M28 - M74 0.07650 0.00112 0.06823 0.07203 0.00827

M28 - M75 0.09634 0.01628 0.08359 0.06250 0.01276

M28 - M76 0.14027 0.01639 0.13247 0.10921 0.00780

M28 - M77 0.08879 0.02063 0.07784 0.05675 0.01095

M28 - M78 0.09171 0.00636 0.08200 0.07333 0.00971

M28 - M79 0.06502 0.00395 0.06182 0.05873 0.00320

M28 - M80 0.07093 0.02108 0.06527 0.04628 0.00566

M28 - M81 0.09744 0.01701 0.08535 0.06927 0.01209

M28 - M82 0.09754 0.01960 0.09019 0.07615 0.00735

M28 - M83 0.10505 0.01773 0.09647 0.08426 0.00858

M28 - M84 0.07350 0.01919 0.06727 0.05308 0.00623

M28 - M85 0.10537 0.01674 0.09419 0.06833 0.01118

M28 - M86 0.10204 0.00585 0.09010 0.08913 0.01194

M28 - M87 0.10532 0.01630 0.09237 0.06842 0.01295

M28 - M88 0.09808 0.00529 0.08983 0.08884 0.00825

M28 - M89 0.10950 0.00708 0.10349 0.10196 0.00601

M28 - M90 0.07960 0.00303 0.07077 0.07776 0.00883

M28 - M91 0.06014 0.00305 0.05346 0.06040 0.00668

M28 - M92 0.08212 0.01099 0.07314 0.07904 0.00898

M28 - M93 0.07267 0.00136 0.06322 0.07327 0.00946

M28 - M94 0.06963 0.00351 0.06426 0.06564 0.00537

M28 - M95 0.07903 0.00737 0.07041 0.06875 0.00863

M28 - M96 0.06978 0.00899 0.06417 0.06386 0.00562

M28 - M97 0.08290 0.00701 0.07737 0.07359 0.00552

M29 - M30 0.04375 0.00742 0.04034 0.05107 0.00341

M29 - M31 0.05148 0.00582 0.04885 0.05053 0.00263

M29 - M32 0.08058 0.00475 0.07651 0.08366 0.00407

M29 - M33 0.04650 0.00622 0.04568 0.03910 0.00082

M29 - M34 0.09174 0.00016 0.09107 0.09122 0.00068

M29 - M35 0.07713 0.00507 0.07385 0.07148 0.00328

M29 - M36 0.04968 0.01573 0.04834 0.04690 0.00134

M29 - M37 0.07331 0.00671 0.07272 0.06836 0.00059

M29 - M38 0.07558 0.00578 0.07464 0.07688 0.00094

M29 - M39 0.02421 0.00002 0.02391 0.02397 0.00029

M29 - M40 0.05729 0.01700 0.05202 0.04514 0.00527

M29 - M41 0.02979 0.01531 0.02892 0.03667 0.00087

M29 - M42 0.07647 0.00442 0.07453 0.06259 0.00194

M29 - M43 0.10810 0.00489 0.10566 0.10257 0.00244

M29 - M44 0.04703 0.00527 0.04627 0.03702 0.00076

M29 - M45 0.09924 0.00028 0.09840 0.09814 0.00083

M29 - M46 0.02091 0.00001 0.02061 0.02086 0.00031

M29 - M47 0.07767 0.00232 0.07645 0.07232 0.00123

M29 - M48 0.09566 0.00213 0.09390 0.08930 0.00176

M29 - M49 0.09802 0.00234 0.09520 0.09107 0.00282

M29 - M50 0.06956 0.00161 0.06823 0.06234 0.00133

M29 - M51 0.01629 0.00185 0.01563 0.01627 0.00066

M29 - M52 0.03220 0.00145 0.03153 0.03139 0.00067

M29 - M53 0.08402 0.00094 0.07957 0.08477 0.00444

M29 - M54 0.06142 0.00151 0.06032 0.06278 0.00110

M29 - M55 0.04003 0.00267 0.03891 0.03415 0.00112

M29 - M56 0.03124 0.00140 0.03063 0.03066 0.00061

M29 - M57 0.02912 0.00657 0.02838 0.02806 0.00074

M29 - M58 0.02755 0.00022 0.02624 0.02794 0.00130

M29 - M59 0.03683 0.00184 0.03528 0.03553 0.00155

M29 - M60 0.03816 0.00079 0.03713 0.03684 0.00103

M29 - M61 0.05171 0.00237 0.04627 0.04727 0.00545

M29 - M62 0.03182 0.00064 0.03141 0.03024 0.00040

M29 - M63 0.03105 0.00063 0.03066 0.02951 0.00040

M29 - M64 0.03985 0.00472 0.03939 0.04364 0.00046

M29 - M65 0.06624 0.00074 0.05922 0.06388 0.00702

M29 - M66 0.06472 0.00157 0.06465 0.06195 0.00007

M29 - M67 0.06375 0.00199 0.06148 0.06170 0.00227

M29 - M68 0.02942 0.00574 0.02864 0.03047 0.00078

M29 - M69 0.04688 0.00333 0.04554 0.04195 0.00134

M29 - M70 0.04562 0.00096 0.04210 0.04674 0.00352

M29 - M71 0.04836 0.00647 0.04700 0.04688 0.00136

M29 - M72 0.04237 0.00486 0.03947 0.03158 0.00290

M29 - M73 0.05792 0.00398 0.05763 0.04119 0.00029

M29 - M74 0.03224 0.00074 0.03205 0.03502 0.00020

M29 - M75 0.04952 0.00415 0.04792 0.03562 0.00160

M29 - M76 0.11160 0.00524 0.11138 0.09961 0.00023

M29 - M77 0.04089 0.00128 0.03938 0.04122 0.00151

M29 - M78 0.03679 0.00038 0.03624 0.03524 0.00054

M29 - M79 0.05784 0.00271 0.05730 0.04843 0.00053

M29 - M80 0.03229 0.00093 0.03159 0.03266 0.00069

M29 - M81 0.05768 0.00418 0.05460 0.04966 0.00309

M29 - M82 0.07209 0.00582 0.06952 0.06978 0.00257

M29 - M83 0.08026 0.00443 0.07905 0.08146 0.00121

M29 - M84 0.04525 0.00558 0.04435 0.03951 0.00090

M29 - M85 0.05743 0.00544 0.05637 0.04515 0.00106

M29 - M86 0.06780 0.00424 0.06368 0.07735 0.00411

M29 - M87 0.05622 0.00495 0.05475 0.04204 0.00147

M29 - M88 0.07922 0.00555 0.07802 0.08118 0.00120

M29 - M89 0.10385 0.00444 0.10341 0.09803 0.00044

M29 - M90 0.06090 0.00139 0.05857 0.05861 0.00233

M29 - M91 0.02834 0.00127 0.02748 0.02525 0.00086

M29 - M92 0.05677 0.00446 0.05311 0.06123 0.00367

M29 - M93 0.03288 0.00032 0.03236 0.03158 0.00052

M29 - M94 0.04760 0.00069 0.04691 0.04996 0.00068

M29 - M95 0.05271 0.00649 0.05167 0.05374 0.00104

M29 - M96 0.05035 0.00503 0.05014 0.05004 0.00020

M29 - M97 0.05768 0.00460 0.05612 0.06059 0.00156

M30 - M31 0.11687 0.07072 0.05216 0.05495 0.06471

M30 - M32 0.15040 0.07591 0.07337 0.07538 0.07703

M30 - M33 0.11374 0.04702 0.07426 0.04647 0.03948

M30 - M34 0.09973 0.00118 0.09785 0.09610 0.00188

M30 - M35 0.06720 0.00794 0.06496 0.06730 0.00224

M30 - M36 0.05834 0.00831 0.05713 0.05368 0.00121

M30 - M37 0.07182 0.00644 0.06880 0.06567 0.00303

M30 - M38 0.08512 0.01890 0.07861 0.07427 0.00652

M30 - M39 0.04813 0.00021 0.04499 0.04777 0.00314

M30 - M40 0.04833 0.00664 0.04558 0.04770 0.00275

M30 - M41 0.04160 0.00417 0.03994 0.04596 0.00166

M30 - M42 0.06373 0.00588 0.06213 0.06474 0.00160

M30 - M43 0.11432 0.01756 0.11046 0.09282 0.00386

M30 - M44 0.04946 0.00710 0.04772 0.04578 0.00174

M30 - M45 0.10710 0.00198 0.10508 0.10110 0.00202

M30 - M46 0.04652 0.00078 0.04413 0.04535 0.00239

M30 - M47 0.07662 0.00284 0.07372 0.07352 0.00291

M30 - M48 0.09011 0.00504 0.08658 0.08605 0.00353

M30 - M49 0.09014 0.00501 0.08547 0.08649 0.00467

M30 - M50 0.07518 0.00385 0.07179 0.07029 0.00338

M30 - M51 0.03797 0.02234 0.03425 0.02192 0.00371

M30 - M52 0.05654 0.02398 0.05248 0.03194 0.00406

M30 - M53 0.06087 0.00096 0.05804 0.06192 0.00283

M30 - M54 0.04865 0.00260 0.04778 0.04693 0.00086

M30 - M55 0.06017 0.02308 0.05499 0.03494 0.00518

M30 - M56 0.06542 0.02160 0.06220 0.03610 0.00322

M30 - M57 0.04611 0.00803 0.04486 0.03761 0.00125

M30 - M58 0.05741 0.00276 0.05538 0.05189 0.00203

M30 - M59 0.05360 0.00417 0.04789 0.05012 0.00571

M30 - M60 0.05970 0.00323 0.05793 0.05838 0.00177

M30 - M61 0.06520 0.00548 0.05830 0.05661 0.00690

M30 - M62 0.05363 0.00146 0.05126 0.05116 0.00237

M30 - M63 0.05347 0.00151 0.05108 0.05063 0.00240

M30 - M64 0.06382 0.01990 0.06216 0.04676 0.00166

M30 - M65 0.06862 0.00147 0.06244 0.06992 0.00618

M30 - M66 0.06852 0.00605 0.06717 0.06821 0.00134

M30 - M67 0.06650 0.00322 0.06499 0.06460 0.00151

M30 - M68 0.05877 0.02183 0.05520 0.03676 0.00357

M30 - M69 0.05366 0.00148 0.05109 0.05354 0.00256

M30 - M70 0.05922 0.00344 0.05286 0.05576 0.00637

M30 - M71 0.07312 0.01811 0.07007 0.05114 0.00305

M30 - M72 0.07087 0.01824 0.06331 0.04061 0.00755

M30 - M73 0.04696 0.00411 0.04571 0.05093 0.00124

M30 - M74 0.05097 0.00130 0.04935 0.05251 0.00162

M30 - M75 0.04020 0.00437 0.03823 0.04512 0.00197

M30 - M76 0.09652 0.00649 0.09435 0.09473 0.00217

M30 - M77 0.05135 0.02290 0.04787 0.04288 0.00348

M30 - M78 0.06358 0.00473 0.06059 0.05223 0.00298

M30 - M79 0.06259 0.00662 0.06177 0.04605 0.00082

M30 - M80 0.05277 0.02106 0.05012 0.03633 0.00264

M30 - M81 0.05281 0.00489 0.05020 0.05455 0.00262

M30 - M82 0.07348 0.00673 0.06717 0.06874 0.00631

M30 - M83 0.08621 0.00643 0.08417 0.07659 0.00205

M30 - M84 0.04997 0.01030 0.04605 0.04612 0.00392

M30 - M85 0.04652 0.00486 0.04458 0.04982 0.00194

M30 - M86 0.07653 0.01565 0.07177 0.07133 0.00476

M30 - M87 0.04322 0.00462 0.04157 0.04928 0.00165

M30 - M88 0.08858 0.01844 0.08561 0.07207 0.00297

M30 - M89 0.11857 0.01720 0.11304 0.08902 0.00553

M30 - M90 0.07643 0.00975 0.06985 0.06578 0.00658

M30 - M91 0.04573 0.00233 0.04307 0.04029 0.00265

M30 - M92 0.07158 0.02220 0.06346 0.06033 0.00813

M30 - M93 0.05137 0.00132 0.04800 0.04875 0.00336

M30 - M94 0.05829 0.00229 0.05587 0.05602 0.00242

M30 - M95 0.07456 0.01948 0.07016 0.05614 0.00441

M30 - M96 0.07453 0.01834 0.07285 0.05212 0.00169

M30 - M97 0.07818 0.01772 0.07585 0.06116 0.00233

M31 - M32 0.18647 0.11426 0.07348 0.07475 0.11299

M31 - M33 0.09763 0.03355 0.07827 0.04548 0.01937

M31 - M34 0.09834 0.00651 0.09549 0.08834 0.00285

M31 - M35 0.06857 0.00866 0.06486 0.06754 0.00371

M31 - M36 0.05686 0.00745 0.05451 0.05409 0.00235

M31 - M37 0.07702 0.01125 0.07313 0.06584 0.00388

M31 - M38 0.08553 0.02149 0.07512 0.07323 0.01041

M31 - M39 0.05219 0.00142 0.04656 0.04683 0.00562

M31 - M40 0.04429 0.00617 0.04024 0.04599 0.00405

M31 - M41 0.04302 0.00420 0.03967 0.04807 0.00335

M31 - M42 0.06389 0.00755 0.06134 0.06000 0.00255

M31 - M43 0.11828 0.02164 0.11362 0.08961 0.00466

M31 - M44 0.04721 0.00949 0.04440 0.04312 0.00281

M31 - M45 0.10369 0.00555 0.10068 0.09346 0.00301

M31 - M46 0.04735 0.00052 0.04211 0.04485 0.00524

M31 - M47 0.08151 0.00565 0.07852 0.07122 0.00299

M31 - M48 0.09072 0.00395 0.08678 0.08354 0.00394

M31 - M49 0.08812 0.00317 0.08330 0.08439 0.00483

M31 - M50 0.07827 0.00949 0.07591 0.06863 0.00237

M31 - M51 0.04284 0.02368 0.03899 0.02178 0.00385

M31 - M52 0.05494 0.02426 0.05234 0.03054 0.00260

M31 - M53 0.06670 0.00485 0.06265 0.06300 0.00406

M31 - M54 0.05154 0.00431 0.05025 0.04707 0.00129

M31 - M55 0.06350 0.02775 0.05781 0.03488 0.00569

M31 - M56 0.06674 0.02519 0.06245 0.03461 0.00429

M31 - M57 0.04508 0.00957 0.04260 0.03557 0.00248

M31 - M58 0.04759 0.00201 0.04280 0.04723 0.00479

M31 - M59 0.05344 0.00713 0.04768 0.04792 0.00577

M31 - M60 0.05425 0.00880 0.05101 0.05307 0.00324

M31 - M61 0.05931 0.00474 0.04924 0.05364 0.01007

M31 - M62 0.05248 0.00212 0.04670 0.04728 0.00578

M31 - M63 0.05243 0.00241 0.04662 0.04674 0.00582

M31 - M64 0.05920 0.01713 0.05693 0.04511 0.00227

M31 - M65 0.06961 0.00243 0.06184 0.06639 0.00778

M31 - M66 0.07715 0.01058 0.07103 0.06847 0.00612

M31 - M67 0.07394 0.00857 0.07116 0.06463 0.00278

M31 - M68 0.06446 0.02811 0.05818 0.03698 0.00628

M31 - M69 0.05823 0.00445 0.05375 0.05208 0.00448

M31 - M70 0.05717 0.00200 0.05063 0.05539 0.00654

M31 - M71 0.07538 0.02159 0.07033 0.05149 0.00505

M31 - M72 0.06863 0.01987 0.06402 0.04130 0.00461

M31 - M73 0.04673 0.00421 0.04419 0.04999 0.00254

M31 - M74 0.05347 0.00045 0.04891 0.05292 0.00456

M31 - M75 0.04400 0.00436 0.04030 0.04637 0.00370

M31 - M76 0.09434 0.01063 0.09080 0.08849 0.00353

M31 - M77 0.04830 0.02057 0.04551 0.04126 0.00278

M31 - M78 0.06043 0.00567 0.05586 0.05249 0.00457

M31 - M79 0.05059 0.00317 0.04895 0.04543 0.00164

M31 - M80 0.05183 0.02274 0.05005 0.03353 0.00179

M31 - M81 0.05976 0.00714 0.05444 0.05356 0.00532

M31 - M82 0.07686 0.00972 0.06971 0.06573 0.00715

M31 - M83 0.07922 0.00625 0.07612 0.07326 0.00310

M31 - M84 0.05758 0.01670 0.05380 0.04315 0.00378

M31 - M85 0.04367 0.00436 0.04027 0.04985 0.00340

M31 - M86 0.07641 0.01577 0.07041 0.07218 0.00600

M31 - M87 0.04461 0.00442 0.04144 0.05031 0.00317

M31 - M88 0.09330 0.02148 0.09010 0.07174 0.00320

M31 - M89 0.12215 0.01881 0.11605 0.08732 0.00610

M31 - M90 0.07057 0.00709 0.06034 0.06288 0.01023

M31 - M91 0.04464 0.00273 0.04025 0.03912 0.00440

M31 - M92 0.06887 0.01757 0.06292 0.06295 0.00595

M31 - M93 0.04991 0.00197 0.04605 0.04795 0.00387

M31 - M94 0.06029 0.00323 0.05509 0.05598 0.00520

M31 - M95 0.07181 0.01952 0.06398 0.05404 0.00783

M31 - M96 0.07547 0.01846 0.07122 0.05221 0.00425

M31 - M97 0.08229 0.02149 0.07962 0.05865 0.00267

M32 - M33 0.09946 0.02478 0.08581 0.06326 0.01365

M32 - M34 0.12886 0.00590 0.12392 0.11420 0.00493

M32 - M35 0.09246 0.01105 0.08214 0.08573 0.01032

M32 - M36 0.08687 0.00668 0.08279 0.08423 0.00408

M32 - M37 0.10388 0.01136 0.09459 0.08764 0.00929

M32 - M38 0.10402 0.02123 0.09218 0.08984 0.01185

M32 - M39 0.08919 0.00146 0.07967 0.08488 0.00951

M32 - M40 0.06986 0.00729 0.06390 0.07161 0.00596

M32 - M41 0.08298 0.00430 0.07753 0.08564 0.00545

M32 - M42 0.10032 0.00884 0.09588 0.08900 0.00444

M32 - M43 0.14124 0.02412 0.13465 0.10695 0.00659

M32 - M44 0.07322 0.01119 0.06858 0.06468 0.00464

M32 - M45 0.13862 0.00923 0.13346 0.11640 0.00516

M32 - M46 0.08615 0.00090 0.07832 0.08280 0.00783

M32 - M47 0.11196 0.00609 0.10744 0.09794 0.00452

M32 - M48 0.11900 0.00470 0.11349 0.11447 0.00551

M32 - M49 0.11528 0.00388 0.10880 0.11387 0.00648

M32 - M50 0.09995 0.00979 0.09515 0.08663 0.00480

M32 - M51 0.06129 0.02278 0.05458 0.04205 0.00671

M32 - M52 0.07853 0.02599 0.07276 0.05075 0.00577

M32 - M53 0.08377 0.00220 0.07895 0.08204 0.00482

M32 - M54 0.07013 0.00367 0.06727 0.06696 0.00286

M32 - M55 0.08749 0.02959 0.07777 0.05620 0.00972

M32 - M56 0.08817 0.02477 0.08189 0.05779 0.00628

M32 - M57 0.06513 0.01009 0.06162 0.05632 0.00351

M32 - M58 0.08658 0.00167 0.07958 0.08564 0.00700

M32 - M59 0.08594 0.00835 0.07635 0.07409 0.00958

M32 - M60 0.08615 0.00770 0.08115 0.08564 0.00500

M32 - M61 0.09414 0.00476 0.07977 0.08250 0.01438

M32 - M62 0.08860 0.00207 0.07959 0.08441 0.00901

M32 - M63 0.08878 0.00237 0.07980 0.08409 0.00898

M32 - M64 0.08615 0.01713 0.08224 0.06850 0.00391

M32 - M65 0.10068 0.00252 0.08941 0.09829 0.01127

M32 - M66 0.09181 0.00954 0.08701 0.08559 0.00480

M32 - M67 0.09390 0.00799 0.08975 0.08634 0.00414

M32 - M68 0.07594 0.01812 0.07092 0.05800 0.00502

M32 - M69 0.07094 0.00377 0.06611 0.07111 0.00483

M32 - M70 0.08419 0.00418 0.07470 0.07709 0.00949

M32 - M71 0.09175 0.02200 0.08549 0.07001 0.00626

M32 - M72 0.09213 0.01968 0.08391 0.06319 0.00822

M32 - M73 0.08584 0.00420 0.08140 0.08872 0.00444

M32 - M74 0.08770 0.00096 0.08121 0.08940 0.00649

M32 - M75 0.08046 0.00433 0.07386 0.08242 0.00660

M32 - M76 0.12992 0.01450 0.12592 0.11077 0.00400

M32 - M77 0.07356 0.02060 0.06960 0.06324 0.00395

M32 - M78 0.08126 0.00273 0.07600 0.07678 0.00526

M32 - M79 0.09926 0.00626 0.09567 0.07719 0.00359

M32 - M80 0.07542 0.02373 0.07248 0.05410 0.00294

M32 - M81 0.08602 0.00753 0.07868 0.08066 0.00734

M32 - M82 0.09476 0.01057 0.08738 0.08422 0.00738

M32 - M83 0.09671 0.00567 0.09182 0.09226 0.00489

M32 - M84 0.07369 0.01433 0.06859 0.06230 0.00510

M32 - M85 0.08567 0.00536 0.07923 0.08819 0.00644

M32 - M86 0.11816 0.01627 0.10961 0.11034 0.00855

M32 - M87 0.08479 0.00475 0.07881 0.08874 0.00598

M32 - M88 0.12262 0.02455 0.11798 0.09224 0.00464

M32 - M89 0.13390 0.01753 0.12762 0.10300 0.00628

M32 - M90 0.08582 0.00492 0.07686 0.08178 0.00896

M32 - M91 0.07315 0.00270 0.06607 0.06952 0.00709

M32 - M92 0.09574 0.01942 0.08743 0.08830 0.00831

M32 - M93 0.08045 0.00127 0.07313 0.08023 0.00733

M32 - M94 0.09798 0.00494 0.08722 0.08378 0.01075

M32 - M95 0.10042 0.01938 0.09470 0.08259 0.00572

M32 - M96 0.10028 0.01870 0.09648 0.07483 0.00380

M32 - M97 0.09299 0.01669 0.08834 0.07716 0.00465

M33 - M34 0.07643 0.00207 0.07570 0.07181 0.00073

M33 - M35 0.06115 0.00430 0.06027 0.05779 0.00088

M33 - M36 0.05186 0.00801 0.04961 0.03973 0.00226

M33 - M37 0.05686 0.00460 0.05636 0.05499 0.00049

M33 - M38 0.09228 0.01692 0.08906 0.06445 0.00323

M33 - M39 0.03238 0.00052 0.03222 0.03239 0.00016

M33 - M40 0.04090 0.00876 0.03911 0.03563 0.00179

M33 - M41 0.04523 0.00406 0.04336 0.03836 0.00187

M33 - M42 0.06722 0.00885 0.06495 0.05072 0.00227

M33 - M43 0.09889 0.01965 0.09577 0.07800 0.00312

M33 - M44 0.04077 0.00768 0.04007 0.03314 0.00069

M33 - M45 0.08274 0.00206 0.08190 0.07622 0.00084

M33 - M46 0.03000 0.00061 0.02961 0.03050 0.00039

M33 - M47 0.06925 0.00258 0.06831 0.06806 0.00094

M33 - M48 0.08223 0.00525 0.08004 0.08221 0.00219

M33 - M49 0.08205 0.00462 0.07885 0.08135 0.00320

M33 - M50 0.06693 0.00942 0.06670 0.05785 0.00022

M33 - M51 0.05292 0.02504 0.05103 0.02429 0.00189

M33 - M52 0.05213 0.02128 0.04850 0.03148 0.00363

M33 - M53 0.07438 0.00376 0.07106 0.06566 0.00331

M33 - M54 0.05375 0.00356 0.05301 0.04816 0.00074

M33 - M55 0.06120 0.02700 0.05515 0.03629 0.00605

M33 - M56 0.05338 0.02337 0.05081 0.03931 0.00257

M33 - M57 0.03617 0.01171 0.03447 0.02702 0.00170

M33 - M58 0.04027 0.00229 0.03918 0.03235 0.00110

M33 - M59 0.04841 0.00821 0.04523 0.03704 0.00318

M33 - M60 0.04884 0.00469 0.04782 0.03810 0.00102

M33 - M61 0.05338 0.00659 0.04926 0.04285 0.00413

M33 - M62 0.03289 0.00160 0.03234 0.03376 0.00055

M33 - M63 0.03344 0.00242 0.03290 0.03301 0.00054

M33 - M64 0.06136 0.02165 0.05957 0.03840 0.00179

M33 - M65 0.05717 0.00255 0.05183 0.05308 0.00534

M33 - M66 0.06429 0.00548 0.06414 0.05800 0.00015

M33 - M67 0.06281 0.00575 0.06244 0.05461 0.00037

M33 - M68 0.05929 0.02168 0.05382 0.02901 0.00546

M33 - M69 0.04031 0.00554 0.03915 0.03849 0.00116

M33 - M70 0.04905 0.00353 0.04543 0.04530 0.00362

M33 - M71 0.06467 0.01930 0.06268 0.04203 0.00199

M33 - M72 0.04633 0.01819 0.04583 0.02791 0.00050

M33 - M73 0.04657 0.00528 0.04620 0.03958 0.00037

M33 - M74 0.03914 0.00238 0.03893 0.03694 0.00021

M33 - M75 0.04190 0.00508 0.04073 0.03630 0.00117

M33 - M76 0.08582 0.00645 0.08483 0.07371 0.00098

M33 - M77 0.06625 0.02004 0.06265 0.04121 0.00361

M33 - M78 0.03861 0.00223 0.03818 0.03580 0.00042

M33 - M79 0.04690 0.00308 0.04623 0.04896 0.00067

M33 - M80 0.05615 0.02110 0.05493 0.03561 0.00122

M33 - M81 0.04903 0.00622 0.04714 0.04075 0.00190

M33 - M82 0.05722 0.01024 0.05288 0.05415 0.00434

M33 - M83 0.07764 0.01249 0.07642 0.06593 0.00122

M33 - M84 0.03896 0.00724 0.03785 0.03431 0.00111

M33 - M85 0.04715 0.00440 0.04408 0.04030 0.00307

M33 - M86 0.09945 0.01627 0.08951 0.06976 0.00995

M33 - M87 0.04803 0.00478 0.04474 0.04021 0.00329

M33 - M88 0.08659 0.01897 0.08316 0.05939 0.00342

M33 - M89 0.09591 0.01702 0.09163 0.07580 0.00428

M33 - M90 0.05652 0.00468 0.05379 0.05493 0.00273

M33 - M91 0.03324 0.00273 0.03219 0.03069 0.00105

M33 - M92 0.09356 0.02001 0.07952 0.05984 0.01404

M33 - M93 0.04064 0.00578 0.03986 0.03803 0.00078

M33 - M94 0.05326 0.00501 0.05119 0.04606 0.00207

M33 - M95 0.07618 0.01938 0.07552 0.04700 0.00067

M33 - M96 0.07449 0.02587 0.07355 0.04404 0.00094

M33 - M97 0.07832 0.02212 0.07661 0.05055 0.00172

M34 - M35 0.11338 0.00319 0.11180 0.10572 0.00158

M34 - M36 0.09015 0.00075 0.08930 0.08573 0.00085

M34 - M37 0.10767 0.00209 0.10499 0.10405 0.00268

M34 - M38 0.11582 0.00243 0.11344 0.11556 0.00238

M34 - M39 0.06900 0.00137 0.06582 0.07735 0.00318

M34 - M40 0.07938 0.00101 0.07611 0.08031 0.00327

M34 - M41 0.08836 0.00000 0.08723 0.08836 0.00113

M34 - M42 0.08924 0.00749 0.07947 0.09258 0.00977

M34 - M43 0.14842 0.01653 0.11084 0.13669 0.03758

M34 - M44 0.07150 0.00389 0.06332 0.07110 0.00818

M34 - M45 0.14591 0.04627 0.08626 0.14012 0.05965

M34 - M46 0.07388 0.00020 0.07236 0.07776 0.00153

M34 - M47 0.12905 0.00111 0.12492 0.12911 0.00413

M34 - M48 0.15446 0.00192 0.14865 0.15152 0.00581

M34 - M49 0.16236 0.00535 0.14718 0.14787 0.01518

M34 - M50 0.12294 0.00867 0.12228 0.11368 0.00066

M34 - M51 0.05388 0.00061 0.05303 0.05280 0.00084

M34 - M52 0.06253 0.00052 0.06117 0.06146 0.00137

M34 - M53 0.13809 0.00780 0.13108 0.12771 0.00701

M34 - M54 0.10775 0.00680 0.10685 0.09810 0.00090

M34 - M55 0.08155 0.00498 0.08054 0.07385 0.00100

M34 - M56 0.09692 0.00781 0.09610 0.07827 0.00082

M34 - M57 0.06233 0.00436 0.06153 0.06069 0.00080

M34 - M58 0.08023 0.00001 0.07680 0.08003 0.00342

M34 - M59 0.08780 0.00632 0.08522 0.07744 0.00258

M34 - M60 0.10645 0.00907 0.10468 0.08303 0.00177

M34 - M61 0.08769 0.00091 0.08166 0.08808 0.00602

M34 - M62 0.07790 0.00020 0.07626 0.08028 0.00164

M34 - M63 0.07782 0.00020 0.07609 0.08022 0.00173

M34 - M64 0.08443 0.00375 0.08362 0.08045 0.00081

M34 - M65 0.11073 0.00389 0.10294 0.10810 0.00779

M34 - M66 0.11538 0.00425 0.11471 0.11328 0.00067

M34 - M67 0.10563 0.00430 0.10411 0.10389 0.00153

M34 - M68 0.06696 0.00255 0.06592 0.06549 0.00104

M34 - M69 0.10522 0.00691 0.10362 0.08675 0.00160

M34 - M70 0.09469 0.00059 0.08789 0.09269 0.00680

M34 - M71 0.08580 0.00026 0.08083 0.08488 0.00497

M34 - M72 0.07556 0.00409 0.07456 0.06571 0.00100

M34 - M73 0.08594 0.00217 0.08392 0.09205 0.00201

M34 - M74 0.07803 0.00067 0.07614 0.08403 0.00188

M34 - M75 0.06979 0.00060 0.06867 0.07523 0.00112

M34 - M76 0.14215 0.04234 0.09705 0.13131 0.04510

M34 - M77 0.08882 0.00116 0.08750 0.09267 0.00132

M34 - M78 0.09763 0.00229 0.09677 0.08690 0.00086

M34 - M79 0.08985 0.00332 0.08654 0.09434 0.00331

M34 - M80 0.07532 0.00104 0.07453 0.07713 0.00078

M34 - M81 0.09042 0.00130 0.08612 0.08655 0.00430

M34 - M82 0.11125 0.00628 0.10312 0.10439 0.00814

M34 - M83 0.11918 0.00291 0.11137 0.11967 0.00781

M34 - M84 0.07831 0.00700 0.07144 0.07150 0.00688

M34 - M85 0.08930 0.00025 0.08795 0.09046 0.00135

M34 - M86 0.13317 0.00007 0.12615 0.13282 0.00701

M34 - M87 0.08735 0.00083 0.08603 0.09171 0.00131

M34 - M88 0.12610 0.01411 0.12387 0.11934 0.00223

M34 - M89 0.14308 0.00373 0.14207 0.13930 0.00101

M34 - M90 0.10511 0.00169 0.09847 0.10586 0.00664

M34 - M91 0.06917 0.00107 0.06571 0.07125 0.00345

M34 - M92 0.11304 0.00216 0.10477 0.10368 0.00828

M34 - M93 0.08464 0.00195 0.07977 0.08673 0.00486

M34 - M94 0.08744 0.00063 0.08635 0.08881 0.00109

M34 - M95 0.09606 0.00437 0.09536 0.08834 0.00070

M34 - M96 0.08501 0.00375 0.08438 0.08402 0.00063

M34 - M97 0.10134 0.00090 0.09933 0.10141 0.00201

M35 - M36 0.10936 0.02256 0.07497 0.06968 0.03440

M35 - M37 0.20113 0.09375 0.11660 0.08794 0.08452

M35 - M38 0.17087 0.09462 0.09126 0.09544 0.07961

M35 - M39 0.07983 0.00080 0.07762 0.07691 0.00221

M35 - M40 0.07552 0.00943 0.07303 0.07026 0.00249

M35 - M41 0.08993 0.00394 0.08525 0.08229 0.00468

M35 - M42 0.10157 0.01943 0.10039 0.08580 0.00118

M35 - M43 0.11564 0.00617 0.11230 0.11127 0.00334

M35 - M44 0.07927 0.02120 0.07303 0.06111 0.00624

M35 - M45 0.11344 0.00366 0.11125 0.10779 0.00219

M35 - M46 0.07838 0.00114 0.07740 0.07676 0.00097

M35 - M47 0.09510 0.00377 0.09171 0.09444 0.00338

M35 - M48 0.11158 0.00173 0.10504 0.11002 0.00655

M35 - M49 0.11171 0.00457 0.10478 0.11086 0.00693

M35 - M50 0.08433 0.00544 0.08358 0.08149 0.00075

M35 - M51 0.07186 0.02532 0.07008 0.04660 0.00178

M35 - M52 0.09118 0.03599 0.08933 0.05337 0.00185

M35 - M53 0.10497 0.01040 0.09807 0.09499 0.00691

M35 - M54 0.08552 0.01355 0.08361 0.07192 0.00192

M35 - M55 0.09810 0.02817 0.09460 0.06428 0.00350

M35 - M56 0.10470 0.02525 0.10126 0.06844 0.00344

M35 - M57 0.07720 0.02140 0.07452 0.05044 0.00268

M35 - M58 0.06869 0.00044 0.06689 0.06948 0.00180

M35 - M59 0.07776 0.00743 0.07569 0.06508 0.00207

M35 - M60 0.07792 0.00450 0.07638 0.06918 0.00154

M35 - M61 0.07679 0.00515 0.07218 0.07457 0.00462

M35 - M62 0.07535 0.00131 0.07431 0.07530 0.00104

M35 - M63 0.07559 0.00094 0.07458 0.07551 0.00102

M35 - M64 0.08086 0.01411 0.07872 0.06898 0.00214

M35 - M65 0.09645 0.00456 0.08981 0.08877 0.00664

M35 - M66 0.08946 0.00547 0.08821 0.08466 0.00125

M35 - M67 0.09118 0.00556 0.08911 0.08101 0.00207

M35 - M68 0.06433 0.01563 0.06302 0.05414 0.00131

M35 - M69 0.06509 0.00423 0.06323 0.06163 0.00186

M35 - M70 0.07691 0.00699 0.06951 0.07214 0.00740

M35 - M71 0.08351 0.01440 0.07524 0.06997 0.00827

M35 - M72 0.07152 0.01078 0.06809 0.05506 0.00343

M35 - M73 0.08989 0.01625 0.08920 0.07808 0.00069

M35 - M74 0.07564 0.00080 0.07325 0.07292 0.00239

M35 - M75 0.08431 0.02162 0.08295 0.07231 0.00136

M35 - M76 0.11191 0.01769 0.10984 0.09539 0.00207

M35 - M77 0.07645 0.02099 0.07395 0.06341 0.00249

M35 - M78 0.06229 0.00096 0.06123 0.06512 0.00106

M35 - M79 0.09497 0.00909 0.09177 0.07978 0.00320

M35 - M80 0.07531 0.02193 0.07377 0.05565 0.00154

M35 - M81 0.10195 0.02597 0.09828 0.07291 0.00367

M35 - M82 0.10639 0.02406 0.09939 0.07458 0.00700

M35 - M83 0.11692 0.01565 0.10845 0.09556 0.00847

M35 - M84 0.08043 0.02016 0.07694 0.05605 0.00348

M35 - M85 0.09385 0.01963 0.09194 0.08448 0.00190

M35 - M86 0.11281 0.01185 0.10810 0.11684 0.00471

M35 - M87 0.09839 0.01969 0.09595 0.08923 0.00244

M35 - M88 0.10637 0.01730 0.10431 0.09076 0.00206

M35 - M89 0.12437 0.01252 0.12267 0.11054 0.00170

M35 - M90 0.07997 0.00890 0.07532 0.07303 0.00465

M35 - M91 0.07743 0.01323 0.07621 0.06369 0.00123

M35 - M92 0.08901 0.00882 0.08336 0.08338 0.00565

M35 - M93 0.07589 0.00136 0.07500 0.07474 0.00089

M35 - M94 0.09294 0.00756 0.08622 0.08260 0.00672

M35 - M95 0.08984 0.00924 0.08899 0.07653 0.00085

M35 - M96 0.08517 0.01236 0.08414 0.07390 0.00103

M35 - M97 0.07799 0.00755 0.07661 0.07539 0.00139

M36 - M37 0.09841 0.02984 0.06680 0.06461 0.03162

M36 - M38 0.08128 0.01270 0.07568 0.06707 0.00560

M36 - M39 0.03979 0.00082 0.03973 0.03948 0.00006

M36 - M40 0.06063 0.01857 0.05786 0.04430 0.00277

M36 - M41 0.04622 0.01524 0.04510 0.04604 0.00112

M36 - M42 0.06267 0.00525 0.06086 0.05882 0.00181

M36 - M43 0.09413 0.00612 0.09127 0.09170 0.00285

M36 - M44 0.05493 0.00826 0.05248 0.04103 0.00245

M36 - M45 0.09537 0.00068 0.09439 0.09075 0.00098

M36 - M46 0.03687 0.00185 0.03680 0.03552 0.00006

M36 - M47 0.08709 0.00504 0.08577 0.07767 0.00131

M36 - M48 0.10271 0.00458 0.10080 0.09224 0.00191

M36 - M49 0.09837 0.00395 0.09423 0.09339 0.00414

M36 - M50 0.07282 0.00431 0.07266 0.06738 0.00016

M36 - M51 0.03478 0.00552 0.03416 0.02848 0.00062

M36 - M52 0.04297 0.00373 0.04219 0.03889 0.00078

M36 - M53 0.09861 0.00397 0.09145 0.09133 0.00716

M36 - M54 0.08046 0.00356 0.07979 0.07321 0.00067

M36 - M55 0.05092 0.00272 0.05018 0.04568 0.00074

M36 - M56 0.05078 0.00247 0.04971 0.04804 0.00106

M36 - M57 0.03373 0.00617 0.03327 0.03242 0.00046

M36 - M58 0.04251 0.00122 0.04154 0.04119 0.00097

M36 - M59 0.05406 0.00479 0.05244 0.04247 0.00163

M36 - M60 0.05086 0.00323 0.04861 0.04675 0.00225

M36 - M61 0.05632 0.00399 0.05161 0.05148 0.00471

M36 - M62 0.03939 0.00151 0.03872 0.04278 0.00067

M36 - M63 0.03841 0.00124 0.03782 0.04182 0.00059

M36 - M64 0.04607 0.00720 0.04582 0.04448 0.00025

M36 - M65 0.07024 0.00376 0.06409 0.06715 0.00614

M36 - M66 0.06788 0.00500 0.06720 0.06005 0.00068

M36 - M67 0.07727 0.00472 0.07559 0.06730 0.00168

M36 - M68 0.03608 0.00699 0.03565 0.03521 0.00042

M36 - M69 0.04586 0.00123 0.04467 0.04390 0.00118

M36 - M70 0.05248 0.00469 0.04825 0.05050 0.00422

M36 - M71 0.05383 0.00764 0.05016 0.04730 0.00367

M36 - M72 0.05399 0.01084 0.05350 0.03979 0.00048

M36 - M73 0.05968 0.00451 0.05941 0.05039 0.00027

M36 - M74 0.04278 0.00132 0.04253 0.04097 0.00025

M36 - M75 0.05318 0.00536 0.05209 0.04319 0.00109

M36 - M76 0.10189 0.00496 0.10154 0.09235 0.00034

M36 - M77 0.06430 0.00340 0.06210 0.05551 0.00220

M36 - M78 0.03523 0.00167 0.03489 0.03779 0.00034

M36 - M79 0.06610 0.00122 0.06555 0.06297 0.00055

M36 - M80 0.04855 0.00466 0.04814 0.04296 0.00041

M36 - M81 0.05957 0.00882 0.05816 0.04692 0.00141

M36 - M82 0.06729 0.00859 0.06322 0.06576 0.00407

M36 - M83 0.07762 0.00746 0.07546 0.07186 0.00217

M36 - M84 0.04663 0.00511 0.04588 0.04433 0.00075

M36 - M85 0.05685 0.00527 0.05569 0.04936 0.00116

M36 - M86 0.07206 0.00506 0.06820 0.07999 0.00386

M36 - M87 0.06014 0.00526 0.05823 0.05007 0.00191

M36 - M88 0.07152 0.00616 0.07041 0.07357 0.00111

M36 - M89 0.09599 0.00635 0.09285 0.08250 0.00314

M36 - M90 0.06708 0.00334 0.06469 0.06082 0.00239

M36 - M91 0.03801 0.00444 0.03717 0.03288 0.00084

M36 - M92 0.06317 0.00864 0.05939 0.06007 0.00378

M36 - M93 0.04335 0.00127 0.04273 0.04452 0.00061

M36 - M94 0.06723 0.00501 0.06656 0.05834 0.00067

M36 - M95 0.07574 0.00934 0.07567 0.06067 0.00007

M36 - M96 0.06942 0.01126 0.06922 0.05270 0.00019

M36 - M97 0.06442 0.00661 0.06402 0.06087 0.00040

M37 - M38 0.14975 0.06435 0.11892 0.08500 0.03083

M37 - M39 0.07058 0.00070 0.07005 0.06968 0.00053

M37 - M40 0.06559 0.00896 0.06359 0.06256 0.00200

M37 - M41 0.07586 0.00413 0.07492 0.07154 0.00094

M37 - M42 0.10149 0.01950 0.09910 0.07990 0.00238

M37 - M43 0.10670 0.00696 0.10344 0.09967 0.00326

M37 - M44 0.07666 0.02140 0.06797 0.05579 0.00869

M37 - M45 0.10834 0.00162 0.10502 0.10646 0.00332

M37 - M46 0.07264 0.00151 0.07181 0.06739 0.00084

M37 - M47 0.09532 0.00677 0.09392 0.08761 0.00140

M37 - M48 0.10752 0.00531 0.10523 0.10161 0.00230

M37 - M49 0.10772 0.00603 0.10428 0.10164 0.00344

M37 - M50 0.07804 0.00470 0.07670 0.07622 0.00134

M37 - M51 0.06327 0.02778 0.06294 0.03930 0.00032

M37 - M52 0.07928 0.03157 0.07909 0.04604 0.00019

M37 - M53 0.08987 0.00640 0.08452 0.08171 0.00535

M37 - M54 0.07659 0.01042 0.07475 0.06282 0.00184

M37 - M55 0.08607 0.02726 0.08485 0.05354 0.00121

M37 - M56 0.08995 0.02576 0.08953 0.05726 0.00043

M37 - M57 0.07527 0.02359 0.07357 0.04745 0.00171

M37 - M58 0.06362 0.00113 0.06215 0.06664 0.00147

M37 - M59 0.07043 0.00621 0.06787 0.06217 0.00255

M37 - M60 0.07338 0.00313 0.07207 0.06691 0.00131

M37 - M61 0.07277 0.00620 0.06716 0.07092 0.00562

M37 - M62 0.06718 0.00063 0.06598 0.06892 0.00120

M37 - M63 0.06770 0.00059 0.06645 0.06895 0.00124

M37 - M64 0.08358 0.01738 0.07835 0.06129 0.00523

M37 - M65 0.08719 0.00698 0.08194 0.08245 0.00525

M37 - M66 0.08963 0.00759 0.08852 0.07787 0.00111

M37 - M67 0.08800 0.00744 0.08606 0.07713 0.00194

M37 - M68 0.05654 0.01333 0.05583 0.04867 0.00071

M37 - M69 0.06362 0.00554 0.06112 0.05898 0.00250

M37 - M70 0.07156 0.00838 0.06422 0.06688 0.00734

M37 - M71 0.06912 0.01149 0.06658 0.06208 0.00254

M37 - M72 0.06020 0.01106 0.05857 0.05227 0.00163

M37 - M73 0.09289 0.01561 0.09064 0.07262 0.00225

M37 - M74 0.07213 0.00093 0.07174 0.06863 0.00039

M37 - M75 0.09386 0.02120 0.09045 0.06477 0.00342

M37 - M76 0.11643 0.01701 0.11350 0.09519 0.00294

M37 - M77 0.08193 0.02197 0.07781 0.05737 0.00412

M37 - M78 0.06158 0.00127 0.06041 0.06404 0.00117

M37 - M79 0.08675 0.00835 0.08632 0.06721 0.00043

M37 - M80 0.06989 0.02234 0.06888 0.04728 0.00101

M37 - M81 0.11212 0.03174 0.11032 0.06512 0.00180

M37 - M82 0.10248 0.02370 0.09931 0.07076 0.00317

M37 - M83 0.10983 0.01840 0.09735 0.08530 0.01248

M37 - M84 0.07896 0.02177 0.07588 0.05266 0.00308

M37 - M85 0.09722 0.01725 0.09409 0.07502 0.00314

M37 - M86 0.10959 0.00794 0.10459 0.10199 0.00501

M37 - M87 0.10074 0.01721 0.09669 0.07821 0.00405

M37 - M88 0.08751 0.00765 0.08566 0.08058 0.00186

M37 - M89 0.11161 0.01151 0.10649 0.09783 0.00512

M37 - M90 0.07999 0.01192 0.07477 0.06940 0.00522

M37 - M91 0.07031 0.01245 0.06889 0.05813 0.00142

M37 - M92 0.08461 0.00955 0.08016 0.07438 0.00445

M37 - M93 0.06461 0.00110 0.06215 0.06846 0.00246

M37 - M94 0.08166 0.00460 0.07952 0.07642 0.00214

M37 - M95 0.09044 0.01183 0.08998 0.07071 0.00046

M37 - M96 0.07792 0.00928 0.07701 0.06711 0.00091

M37 - M97 0.07543 0.00690 0.07369 0.06897 0.00173

M38 - M39 0.08002 0.00088 0.07769 0.07593 0.00234

M38 - M40 0.07198 0.00750 0.06881 0.07226 0.00317

M38 - M41 0.07924 0.00418 0.07777 0.08119 0.00147

M38 - M42 0.08676 0.01154 0.08551 0.08065 0.00125

M38 - M43 0.12755 0.01733 0.12425 0.10818 0.00330

M38 - M44 0.07139 0.01378 0.06623 0.06602 0.00516

M38 - M45 0.12029 0.00488 0.11686 0.12008 0.00343

M38 - M46 0.07749 0.00104 0.07583 0.07384 0.00166

M38 - M47 0.09150 0.00383 0.08969 0.08735 0.00181

M38 - M48 0.10450 0.00255 0.10167 0.10059 0.00283

M38 - M49 0.10282 0.00353 0.09763 0.10093 0.00519

M38 - M50 0.08534 0.00511 0.08372 0.08384 0.00162

M38 - M51 0.07215 0.03191 0.07152 0.04124 0.00063

M38 - M52 0.08292 0.03348 0.08228 0.04877 0.00064

M38 - M53 0.08844 0.00808 0.08543 0.08372 0.00301

M38 - M54 0.07737 0.01390 0.07711 0.06477 0.00026

M38 - M55 0.08382 0.02638 0.08113 0.05350 0.00269

M38 - M56 0.08694 0.02285 0.08573 0.05607 0.00121

M38 - M57 0.05808 0.00725 0.05666 0.05158 0.00142

M38 - M58 0.07232 0.00070 0.07013 0.07219 0.00218

M38 - M59 0.07351 0.00572 0.07119 0.06750 0.00233

M38 - M60 0.07842 0.00236 0.07688 0.07572 0.00154

M38 - M61 0.08246 0.00426 0.07771 0.07504 0.00475

M38 - M62 0.07444 0.00127 0.07306 0.07360 0.00138

M38 - M63 0.07500 0.00093 0.07364 0.07404 0.00136

M38 - M64 0.08772 0.02329 0.08334 0.06699 0.00438

M38 - M65 0.08949 0.00227 0.08413 0.08711 0.00536

M38 - M66 0.08881 0.00279 0.08775 0.08714 0.00106

M38 - M67 0.08130 0.00269 0.08037 0.07906 0.00094

M38 - M68 0.07648 0.02369 0.07518 0.05357 0.00130

M38 - M69 0.07517 0.00413 0.07332 0.07236 0.00185

M38 - M70 0.07877 0.00732 0.07268 0.07311 0.00609

M38 - M71 0.10987 0.02906 0.10357 0.06995 0.00630

M38 - M72 0.08280 0.01934 0.07939 0.05623 0.00341

M38 - M73 0.07898 0.00516 0.07809 0.07834 0.00089

M38 - M74 0.07781 0.00034 0.07625 0.07706 0.00155

M38 - M75 0.06748 0.00887 0.06572 0.07167 0.00176

M38 - M76 0.10243 0.00822 0.09909 0.10523 0.00334

M38 - M77 0.06932 0.02085 0.06691 0.06174 0.00241

M38 - M78 0.08223 0.00081 0.08048 0.07961 0.00175

M38 - M79 0.07818 0.00738 0.07774 0.06940 0.00043

M38 - M80 0.07224 0.02140 0.07061 0.05411 0.00163

M38 - M81 0.07332 0.00647 0.06868 0.07283 0.00464

M38 - M82 0.08320 0.00643 0.07694 0.07868 0.00626

M38 - M83 0.09576 0.00683 0.09364 0.09325 0.00212

M38 - M84 0.06458 0.00805 0.06068 0.05760 0.00391

M38 - M85 0.07025 0.00752 0.06871 0.07494 0.00154

M38 - M86 0.10585 0.02058 0.09983 0.10011 0.00602

M38 - M87 0.07019 0.00763 0.06823 0.07786 0.00196

M38 - M88 0.11789 0.02450 0.11196 0.09268 0.00592

M38 - M89 0.15377 0.02670 0.15302 0.12017 0.00075

M38 - M90 0.08547 0.00589 0.08242 0.07905 0.00304

M38 - M91 0.06856 0.00672 0.06724 0.06255 0.00131

M38 - M92 0.10035 0.01938 0.09618 0.08378 0.00418

M38 - M93 0.07212 0.00164 0.07104 0.07008 0.00108

M38 - M94 0.08251 0.00575 0.07721 0.07340 0.00530

M38 - M95 0.08596 0.01805 0.08478 0.06805 0.00117

M38 - M96 0.09421 0.01939 0.09102 0.06903 0.00318

M38 - M97 0.09962 0.01987 0.09859 0.07619 0.00102

M39 - M40 0.03145 0.00039 0.02920 0.03136 0.00225

M39 - M41 0.01965 0.00000 0.01848 0.01964 0.00117

M39 - M42 0.04853 0.00013 0.04815 0.04941 0.00038

M39 - M43 0.10069 0.00080 0.09423 0.10372 0.00647

M39 - M44 0.02554 0.00128 0.02419 0.02869 0.00134

M39 - M45 0.07750 0.00136 0.07391 0.08546 0.00359

M39 - M46 0.00887 0.00032 0.00885 0.00935 0.00002

M39 - M47 0.09163 0.00089 0.08793 0.08982 0.00370

M39 - M48 0.11144 0.00048 0.10615 0.10956 0.00530

M39 - M49 0.11042 0.00053 0.10367 0.11107 0.00675

M39 - M50 0.07888 0.00150 0.07885 0.07465 0.00004

M39 - M51 0.01364 0.00078 0.01336 0.01211 0.00028

M39 - M52 0.02798 0.00090 0.02793 0.02352 0.00005

M39 - M53 0.08887 0.00046 0.08428 0.08974 0.00459

M39 - M54 0.06104 0.00086 0.06100 0.06030 0.00004

M39 - M55 0.03836 0.00189 0.03758 0.03508 0.00078

M39 - M56 0.03774 0.00102 0.03741 0.03328 0.00033

M39 - M57 0.02592 0.00117 0.02568 0.02270 0.00024

M39 - M58 0.01931 0.00027 0.01737 0.01920 0.00194

M39 - M59 0.03882 0.00030 0.03565 0.03935 0.00317

M39 - M60 0.03733 0.00062 0.03591 0.03417 0.00143

M39 - M61 0.05259 0.00154 0.04417 0.04942 0.00842

M39 - M62 0.02495 0.00042 0.02411 0.02371 0.00084

M39 - M63 0.02297 0.00022 0.02228 0.02241 0.00069

M39 - M64 0.03878 0.00057 0.03824 0.03749 0.00054

M39 - M65 0.06665 0.00054 0.05819 0.06495 0.00846

M39 - M66 0.07404 0.00109 0.07403 0.07217 0.00001

M39 - M67 0.07175 0.00166 0.07152 0.07383 0.00023

M39 - M68 0.02599 0.00047 0.02471 0.02518 0.00128

M39 - M69 0.03611 0.00050 0.03469 0.03752 0.00142

M39 - M70 0.05213 0.00081 0.04433 0.05363 0.00781

M39 - M71 0.04568 0.00072 0.04075 0.04575 0.00493

M39 - M72 0.02167 0.00053 0.02122 0.02207 0.00046

M39 - M73 0.02288 0.00017 0.02281 0.02346 0.00007

M39 - M74 0.02007 0.00052 0.02005 0.02165 0.00002

M39 - M75 0.02107 0.00013 0.02008 0.02107 0.00099

M39 - M76 0.07614 0.00137 0.07481 0.08269 0.00132

M39 - M77 0.03601 0.00028 0.03410 0.03634 0.00191

M39 - M78 0.02153 0.00010 0.02087 0.02110 0.00066

M39 - M79 0.04382 0.00024 0.04372 0.04506 0.00010

M39 - M80 0.02919 0.00088 0.02910 0.02923 0.00009

M39 - M81 0.03701 0.00054 0.03447 0.03777 0.00253

M39 - M82 0.06362 0.00026 0.05901 0.06549 0.00461

M39 - M83 0.08351 0.00055 0.08124 0.08204 0.00227

M39 - M84 0.03480 0.00060 0.03292 0.03357 0.00188

M39 - M85 0.02748 0.00086 0.02649 0.02847 0.00099

M39 - M86 0.07712 0.00077 0.07054 0.07885 0.00658

M39 - M87 0.02816 0.00072 0.02720 0.02915 0.00096

M39 - M88 0.07925 0.00085 0.07750 0.07731 0.00174

M39 - M89 0.09183 0.00028 0.09178 0.09324 0.00005

M39 - M90 0.07043 0.00143 0.06624 0.06512 0.00419

M39 - M91 0.01832 0.00080 0.01694 0.01832 0.00139

M39 - M92 0.06386 0.00055 0.05491 0.06245 0.00895

M39 - M93 0.01992 0.00062 0.01855 0.02254 0.00137

M39 - M94 0.04611 0.00047 0.04552 0.04439 0.00059

M39 - M95 0.05493 0.00052 0.05488 0.05492 0.00005

M39 - M96 0.05302 0.00068 0.05296 0.05120 0.00006

M39 - M97 0.05991 0.00055 0.05896 0.05919 0.00095

M40 - M41 0.04907 0.01612 0.03926 0.04376 0.00980

M40 - M42 0.06910 0.00748 0.06722 0.05538 0.00188

M40 - M43 0.09357 0.00850 0.08930 0.08875 0.00428

M40 - M44 0.15804 0.10455 0.06625 0.04009 0.09179

M40 - M45 0.08399 0.00070 0.08076 0.08547 0.00323

M40 - M46 0.03055 0.00050 0.02857 0.03068 0.00198

M40 - M47 0.07377 0.00240 0.07139 0.07281 0.00237

M40 - M48 0.08712 0.00192 0.08379 0.08660 0.00333

M40 - M49 0.08835 0.00176 0.08380 0.08869 0.00455

M40 - M50 0.06592 0.00583 0.06416 0.06216 0.00176

M40 - M51 0.03629 0.00532 0.03127 0.03025 0.00503

M40 - M52 0.04410 0.00471 0.03726 0.04121 0.00684

M40 - M53 0.09706 0.00786 0.08963 0.09181 0.00743

M40 - M54 0.07686 0.00359 0.07253 0.07441 0.00433

M40 - M55 0.04324 0.00358 0.03858 0.04313 0.00467

M40 - M56 0.04589 0.00377 0.04089 0.04333 0.00500

M40 - M57 0.04462 0.01601 0.04041 0.03035 0.00421

M40 - M58 0.03646 0.00126 0.03271 0.03775 0.00376

M40 - M59 0.04141 0.00525 0.03859 0.04047 0.00282

M40 - M60 0.04103 0.00242 0.03825 0.04086 0.00278

M40 - M61 0.05404 0.00424 0.04858 0.05228 0.00547

M40 - M62 0.04402 0.00225 0.04017 0.04215 0.00384

M40 - M63 0.04380 0.00225 0.03958 0.04214 0.00422

M40 - M64 0.05246 0.00830 0.04866 0.04764 0.00379

M40 - M65 0.06703 0.00156 0.05833 0.06717 0.00870

M40 - M66 0.06931 0.00487 0.06735 0.06008 0.00196

M40 - M67 0.06750 0.00703 0.06555 0.05983 0.00196

M40 - M68 0.03685 0.00938 0.03255 0.03200 0.00430

M40 - M69 0.04473 0.00274 0.03998 0.04424 0.00475

M40 - M70 0.05289 0.00433 0.04757 0.04874 0.00532

M40 - M71 0.04843 0.00514 0.04548 0.04643 0.00295

M40 - M72 0.04273 0.01257 0.04063 0.03165 0.00210

M40 - M73 0.05495 0.00426 0.05300 0.04438 0.00195

M40 - M74 0.03846 0.00055 0.03609 0.03870 0.00237

M40 - M75 0.04503 0.00491 0.04296 0.03502 0.00206

M40 - M76 0.08445 0.00498 0.08275 0.07826 0.00170

M40 - M77 0.04810 0.00216 0.04254 0.04836 0.00557

M40 - M78 0.04050 0.00148 0.03866 0.03957 0.00183

M40 - M79 0.06561 0.00274 0.05969 0.06405 0.00591

M40 - M80 0.04276 0.00287 0.03800 0.04303 0.00477

M40 - M81 0.05932 0.00811 0.05620 0.04659 0.00311

M40 - M82 0.08090 0.01387 0.07284 0.05931 0.00806

M40 - M83 0.08469 0.00537 0.08209 0.07643 0.00260

M40 - M84 0.05203 0.01418 0.04737 0.04220 0.00466

M40 - M85 0.05777 0.00594 0.05542 0.04671 0.00236

M40 - M86 0.07169 0.00599 0.06520 0.07588 0.00649

M40 - M87 0.05551 0.00587 0.05313 0.04539 0.00238

M40 - M88 0.07274 0.00705 0.06977 0.07092 0.00298

M40 - M89 0.09542 0.00696 0.09374 0.09139 0.00168

M40 - M90 0.06418 0.00572 0.05494 0.05740 0.00924

M40 - M91 0.03596 0.00208 0.03283 0.03501 0.00313

M40 - M92 0.05271 0.00559 0.04729 0.05694 0.00541

M40 - M93 0.04536 0.00259 0.03809 0.04073 0.00727

M40 - M94 0.04871 0.00227 0.04490 0.04965 0.00381

M40 - M95 0.04914 0.00597 0.04739 0.05088 0.00174

M40 - M96 0.05798 0.01101 0.05632 0.04761 0.00166

M40 - M97 0.06261 0.00698 0.06032 0.06073 0.00229

M41 - M42 0.07348 0.00431 0.07138 0.06050 0.00210

M41 - M43 0.11330 0.00384 0.11021 0.10960 0.00309

M41 - M44 0.04606 0.00401 0.04492 0.03798 0.00114

M41 - M45 0.09561 0.00000 0.09432 0.09561 0.00129

M41 - M46 0.01689 0.00000 0.01571 0.01684 0.00117

M41 - M47 0.06950 0.00009 0.06808 0.06986 0.00142

M41 - M48 0.08562 0.00011 0.08315 0.08614 0.00247

M41 - M49 0.09083 0.00017 0.08657 0.09112 0.00426

M41 - M50 0.06834 0.00064 0.06709 0.06474 0.00125

M41 - M51 0.01030 0.00016 0.00946 0.01030 0.00083

M41 - M52 0.02620 0.00031 0.02613 0.02521 0.00007

M41 - M53 0.08695 0.00032 0.08111 0.08594 0.00584

M41 - M54 0.05860 0.00048 0.05859 0.05898 0.00001

M41 - M55 0.02903 0.00013 0.02861 0.03019 0.00042

M41 - M56 0.02723 0.00005 0.02669 0.02723 0.00053

M41 - M57 0.02701 0.00433 0.02614 0.02533 0.00087

M41 - M58 0.02286 0.00011 0.02148 0.02275 0.00139

M41 - M59 0.03466 0.00033 0.03273 0.03438 0.00194

M41 - M60 0.03273 0.00016 0.03147 0.03336 0.00126

M41 - M61 0.04662 0.00016 0.04133 0.04658 0.00528

M41 - M62 0.02774 0.00010 0.02685 0.02762 0.00089

M41 - M63 0.02676 0.00010 0.02587 0.02659 0.00088

M41 - M64 0.03775 0.00398 0.03695 0.04107 0.00079

M41 - M65 0.06337 0.00022 0.05759 0.06224 0.00577

M41 - M66 0.07298 0.00103 0.07165 0.06662 0.00134

M41 - M67 0.06249 0.00016 0.06058 0.06281 0.00191

M41 - M68 0.02541 0.00440 0.02463 0.02725 0.00079

M41 - M69 0.03647 0.00013 0.03482 0.03646 0.00165

M41 - M70 0.04420 0.00026 0.03962 0.04509 0.00458

M41 - M71 0.05095 0.00404 0.04912 0.04999 0.00183

M41 - M72 0.03677 0.00408 0.03480 0.02926 0.00197

M41 - M73 0.04947 0.00392 0.04821 0.03379 0.00126

M41 - M74 0.03020 0.00006 0.02926 0.03059 0.00094

M41 - M75 0.04818 0.00397 0.04476 0.03202 0.00341

M41 - M76 0.10764 0.00382 0.10665 0.09639 0.00099

M41 - M77 0.03423 0.00078 0.03299 0.03434 0.00124

M41 - M78 0.03079 0.00007 0.02855 0.03126 0.00224

M41 - M79 0.04289 0.00040 0.04276 0.04449 0.00013

M41 - M80 0.02441 0.00033 0.02429 0.02586 0.00012

M41 - M81 0.06144 0.00400 0.05601 0.04822 0.00542

M41 - M82 0.06799 0.00403 0.06378 0.06861 0.00420

M41 - M83 0.08899 0.00432 0.08758 0.08647 0.00141

M41 - M84 0.03861 0.00412 0.03742 0.03825 0.00118

M41 - M85 0.05819 0.00387 0.05449 0.04092 0.00371

M41 - M86 0.06687 0.00388 0.06160 0.07811 0.00527

M41 - M87 0.05675 0.00386 0.05308 0.03961 0.00367

M41 - M88 0.07633 0.00400 0.07477 0.07971 0.00156

M41 - M89 0.11227 0.00402 0.11138 0.10684 0.00089

M41 - M90 0.05571 0.00009 0.05262 0.05574 0.00309

M41 - M91 0.02131 0.00009 0.01987 0.02101 0.00145

M41 - M92 0.05722 0.00455 0.05208 0.06208 0.00514

M41 - M93 0.02795 0.00003 0.02694 0.02821 0.00101

M41 - M94 0.04541 0.00033 0.04439 0.04628 0.00102

M41 - M95 0.04933 0.00396 0.04869 0.05339 0.00063

M41 - M96 0.05656 0.00434 0.05592 0.05373 0.00064

M41 - M97 0.06196 0.00445 0.06109 0.06079 0.00087

M42 - M43 0.12185 0.01168 0.10275 0.10963 0.01909

M42 - M44 0.06534 0.02149 0.05801 0.04573 0.00733

M42 - M45 0.09605 0.01168 0.08287 0.09886 0.01317

M42 - M46 0.04663 0.00057 0.04623 0.05041 0.00040

M42 - M47 0.08969 0.00479 0.08839 0.08395 0.00131

M42 - M48 0.10185 0.00274 0.09806 0.10140 0.00379

M42 - M49 0.10592 0.00284 0.09883 0.10069 0.00710

M42 - M50 0.07723 0.00395 0.07687 0.07301 0.00036

M42 - M51 0.06220 0.02431 0.06094 0.04100 0.00127

M42 - M52 0.07196 0.02228 0.06943 0.04801 0.00254

M42 - M53 0.10019 0.00833 0.09671 0.09270 0.00349

M42 - M54 0.06982 0.00386 0.06857 0.06893 0.00125

M42 - M55 0.08568 0.02521 0.08181 0.05954 0.00388

M42 - M56 0.09316 0.02215 0.09061 0.05900 0.00255

M42 - M57 0.06726 0.02069 0.06378 0.04100 0.00349

M42 - M58 0.04943 0.00042 0.04818 0.04827 0.00125

M42 - M59 0.05887 0.00570 0.05687 0.04814 0.00200

M42 - M60 0.05544 0.00216 0.05426 0.05070 0.00118

M42 - M61 0.07277 0.00722 0.06767 0.05768 0.00510

M42 - M62 0.04910 0.00097 0.04814 0.05057 0.00096

M42 - M63 0.04911 0.00097 0.04822 0.05044 0.00089

M42 - M64 0.05360 0.00594 0.05319 0.05485 0.00041

M42 - M65 0.07560 0.00388 0.07015 0.07335 0.00546

M42 - M66 0.07677 0.00783 0.07585 0.07941 0.00091

M42 - M67 0.07059 0.00450 0.06989 0.07210 0.00070

M42 - M68 0.04595 0.00727 0.04459 0.04368 0.00136

M42 - M69 0.05623 0.00254 0.05353 0.05239 0.00270

M42 - M70 0.06898 0.00862 0.06295 0.05886 0.00602

M42 - M71 0.06308 0.00885 0.06099 0.05971 0.00209

M42 - M72 0.05293 0.00569 0.05130 0.04365 0.00163

M42 - M73 0.06115 0.01549 0.06016 0.06156 0.00099

M42 - M74 0.05603 0.00096 0.05521 0.05521 0.00081

M42 - M75 0.05631 0.01551 0.05537 0.05252 0.00094

M42 - M76 0.10180 0.02632 0.08173 0.09671 0.02007

M42 - M77 0.07459 0.02484 0.07218 0.06109 0.00242

M42 - M78 0.06256 0.00159 0.06083 0.05733 0.00173

M42 - M79 0.06880 0.00411 0.06767 0.06917 0.00114

M42 - M80 0.07134 0.02504 0.06629 0.05022 0.00505

M42 - M81 0.07216 0.01635 0.07024 0.06533 0.00192

M42 - M82 0.10021 0.01951 0.09252 0.07583 0.00770

M42 - M83 0.11695 0.01696 0.11004 0.09040 0.00692

M42 - M84 0.06960 0.02326 0.06769 0.04638 0.00191

M42 - M85 0.07287 0.01629 0.07195 0.06906 0.00092

M42 - M86 0.09450 0.00411 0.08958 0.09990 0.00491

M42 - M87 0.07844 0.01696 0.07757 0.06895 0.00087

M42 - M88 0.09781 0.00974 0.09591 0.09031 0.00190

M42 - M89 0.10536 0.00593 0.10491 0.10320 0.00045

M42 - M90 0.07380 0.00335 0.07031 0.06553 0.00349

M42 - M91 0.04680 0.00221 0.04584 0.04533 0.00096

M42 - M92 0.07715 0.00615 0.07280 0.07890 0.00435

M42 - M93 0.06172 0.00282 0.06101 0.05818 0.00071

M42 - M94 0.06148 0.00518 0.06058 0.05862 0.00090

M42 - M95 0.06458 0.00760 0.06359 0.06463 0.00099

M42 - M96 0.07360 0.00839 0.07187 0.06386 0.00173

M42 - M97 0.07551 0.00859 0.07428 0.07125 0.00123

M43 - M44 0.08566 0.01268 0.07854 0.07651 0.00712

M43 - M45 0.15772 0.02230 0.11481 0.14090 0.04291

M43 - M46 0.10269 0.00115 0.09877 0.10363 0.00392

M43 - M47 0.12617 0.00675 0.12290 0.11315 0.00327

M43 - M48 0.14237 0.00722 0.13775 0.12968 0.00462

M43 - M49 0.15498 0.01193 0.14676 0.12863 0.00822

M43 - M50 0.12323 0.01551 0.11857 0.10314 0.00467

M43 - M51 0.08809 0.02384 0.08541 0.06325 0.00269

M43 - M52 0.09565 0.02497 0.09236 0.07053 0.00329

M43 - M53 0.11757 0.00500 0.11394 0.11328 0.00363

M43 - M54 0.09151 0.00531 0.08967 0.08863 0.00184

M43 - M55 0.10710 0.02483 0.10095 0.08403 0.00615

M43 - M56 0.12740 0.02906 0.11939 0.09361 0.00801

M43 - M57 0.07194 0.00752 0.06878 0.06948 0.00317

M43 - M58 0.09077 0.00060 0.08636 0.09326 0.00440

M43 - M59 0.08561 0.00694 0.08176 0.07770 0.00385

M43 - M60 0.10056 0.00706 0.09738 0.08867 0.00318

M43 - M61 0.08791 0.00489 0.08171 0.08713 0.00621

M43 - M62 0.09857 0.00117 0.09441 0.09667 0.00416

M43 - M63 0.09967 0.00126 0.09505 0.09708 0.00463

M43 - M64 0.10676 0.01882 0.10396 0.08609 0.00281

M43 - M65 0.10685 0.00265 0.09745 0.10234 0.00940

M43 - M66 0.10465 0.00571 0.10114 0.10118 0.00351

M43 - M67 0.10016 0.00581 0.09709 0.09530 0.00307

M43 - M68 0.09897 0.02133 0.09612 0.07394 0.00285

M43 - M69 0.10528 0.01161 0.10191 0.08721 0.00337

M43 - M70 0.08959 0.00361 0.08211 0.08592 0.00748

M43 - M71 0.10808 0.02120 0.10110 0.08650 0.00699

M43 - M72 0.09883 0.02053 0.09391 0.07204 0.00491

M43 - M73 0.10589 0.00435 0.10219 0.10531 0.00369

M43 - M74 0.08606 0.00141 0.08256 0.09069 0.00350

M43 - M75 0.09693 0.00493 0.09395 0.09433 0.00298

M43 - M76 0.15727 0.02582 0.12542 0.13253 0.03185

M43 - M77 0.11821 0.02203 0.11617 0.09234 0.00204

M43 - M78 0.09761 0.00141 0.09497 0.09752 0.00264

M43 - M79 0.08795 0.00186 0.08646 0.08897 0.00149

M43 - M80 0.10379 0.02339 0.10247 0.07630 0.00131

M43 - M81 0.10607 0.00678 0.10207 0.09536 0.00401

M43 - M82 0.11476 0.01160 0.10992 0.09833 0.00483

M43 - M83 0.11898 0.00668 0.11220 0.11523 0.00678

M43 - M84 0.08509 0.01140 0.08148 0.07689 0.00361

M43 - M85 0.11750 0.00649 0.11308 0.11186 0.00442

M43 - M86 0.16984 0.01968 0.16360 0.14179 0.00624

M43 - M87 0.12069 0.00679 0.11431 0.11357 0.00638

M43 - M88 0.14751 0.03245 0.14385 0.11427 0.00366

M43 - M89 0.14686 0.01823 0.14245 0.12829 0.00441

M43 - M90 0.09655 0.00479 0.09057 0.09471 0.00599

M43 - M91 0.08217 0.00323 0.07775 0.08388 0.00441

M43 - M92 0.12967 0.01871 0.11732 0.10184 0.01235

M43 - M93 0.10584 0.00168 0.09874 0.10582 0.00711

M43 - M94 0.09169 0.00224 0.08879 0.09168 0.00290

M43 - M95 0.11887 0.02241 0.11589 0.09161 0.00298

M43 - M96 0.11588 0.02328 0.11244 0.08877 0.00344

M43 - M97 0.13068 0.01960 0.12584 0.10339 0.00484

M44 - M45 0.07917 0.00768 0.06912 0.07647 0.01005

M44 - M46 0.02870 0.00108 0.02822 0.02605 0.00047

M44 - M47 0.06816 0.00339 0.06676 0.06675 0.00140

M44 - M48 0.08059 0.00267 0.07775 0.07938 0.00284

M44 - M49 0.08004 0.00249 0.07558 0.07966 0.00445

M44 - M50 0.06424 0.01195 0.06364 0.05821 0.00060

M44 - M51 0.05401 0.03344 0.05219 0.02275 0.00182

M44 - M52 0.05654 0.02570 0.05126 0.03011 0.00529

M44 - M53 0.06868 0.00909 0.06586 0.06308 0.00282

M44 - M54 0.05063 0.00512 0.04853 0.04741 0.00210

M44 - M55 0.05998 0.02580 0.05511 0.03531 0.00487

M44 - M56 0.06794 0.02562 0.06315 0.03792 0.00479

M44 - M57 0.05777 0.02909 0.05720 0.02586 0.00057

M44 - M58 0.03146 0.00146 0.02935 0.03222 0.00211

M44 - M59 0.03808 0.00438 0.03581 0.03572 0.00227

M44 - M60 0.03822 0.00174 0.03612 0.03792 0.00209

M44 - M61 0.04680 0.00430 0.04227 0.04321 0.00453

M44 - M62 0.03621 0.00298 0.03457 0.03514 0.00164

M44 - M63 0.03600 0.00295 0.03408 0.03502 0.00192

M44 - M64 0.04079 0.00758 0.03987 0.03924 0.00092

M44 - M65 0.05813 0.00303 0.05050 0.05764 0.00762

M44 - M66 0.05725 0.00315 0.05654 0.05616 0.00071

M44 - M67 0.05994 0.00668 0.05907 0.05396 0.00087

M44 - M68 0.03855 0.01215 0.03691 0.02721 0.00165

M44 - M69 0.04267 0.00312 0.04066 0.03916 0.00202

M44 - M70 0.05807 0.01153 0.05249 0.04450 0.00558

M44 - M71 0.04738 0.00801 0.04540 0.04126 0.00198

M44 - M72 0.03941 0.01333 0.03748 0.02829 0.00193

M44 - M73 0.04783 0.01521 0.04436 0.03851 0.00348

M44 - M74 0.03188 0.00097 0.03070 0.03257 0.00118

M44 - M75 0.03886 0.01633 0.03632 0.02892 0.00254

M44 - M76 0.08754 0.02297 0.07576 0.07069 0.01178

M44 - M77 0.06078 0.02228 0.05802 0.04535 0.00277

M44 - M78 0.03391 0.00098 0.03316 0.03355 0.00075

M44 - M79 0.04651 0.00315 0.04596 0.04674 0.00055

M44 - M80 0.05788 0.02387 0.05527 0.03953 0.00261

M44 - M81 0.05722 0.02040 0.05344 0.03964 0.00378

M44 - M82 0.08642 0.02116 0.07191 0.05589 0.01451

M44 - M83 0.09083 0.01750 0.08422 0.06754 0.00661

M44 - M84 0.06694 0.02508 0.05222 0.03776 0.01472

M44 - M85 0.04752 0.01732 0.04471 0.03593 0.00281

M44 - M86 0.06171 0.00561 0.05722 0.06451 0.00449

M44 - M87 0.04805 0.01690 0.04498 0.03665 0.00307

M44 - M88 0.06984 0.01249 0.06658 0.06321 0.00325

M44 - M89 0.08912 0.01029 0.08808 0.08086 0.00104

M44 - M90 0.06370 0.00896 0.05548 0.05540 0.00822

M44 - M91 0.03173 0.00270 0.03009 0.02941 0.00164

M44 - M92 0.05198 0.00672 0.04744 0.05272 0.00454

M44 - M93 0.04052 0.00210 0.03607 0.03623 0.00445

M44 - M94 0.04430 0.00397 0.04276 0.03971 0.00154

M44 - M95 0.04516 0.00483 0.04437 0.04640 0.00079

M44 - M96 0.05427 0.01086 0.05340 0.04210 0.00087

M44 - M97 0.06082 0.00933 0.05980 0.05339 0.00102

M45 - M46 0.08154 0.00020 0.07970 0.08532 0.00185

M45 - M47 0.13214 0.00071 0.12891 0.13278 0.00324

M45 - M48 0.15773 0.00242 0.15180 0.15511 0.00592

M45 - M49 0.16697 0.00585 0.15030 0.15121 0.01667

M45 - M50 0.13009 0.01177 0.12922 0.11777 0.00087

M45 - M51 0.06133 0.00221 0.06027 0.05732 0.00107

M45 - M52 0.06736 0.00071 0.06565 0.06564 0.00171

M45 - M53 0.13837 0.00493 0.13207 0.13195 0.00629

M45 - M54 0.10781 0.00403 0.10688 0.10227 0.00093

M45 - M55 0.08615 0.00518 0.08503 0.07844 0.00113

M45 - M56 0.10273 0.00802 0.10174 0.08352 0.00098

M45 - M57 0.06531 0.00324 0.06420 0.06495 0.00111

M45 - M58 0.08688 0.00001 0.08330 0.08664 0.00358

M45 - M59 0.09263 0.00607 0.08936 0.08172 0.00327

M45 - M60 0.11287 0.00922 0.11101 0.08892 0.00186

M45 - M61 0.09356 0.00172 0.08620 0.09187 0.00736

M45 - M62 0.08446 0.00026 0.08265 0.08632 0.00181

M45 - M63 0.08453 0.00026 0.08261 0.08641 0.00192

M45 - M64 0.08678 0.00274 0.08570 0.08446 0.00109

M45 - M65 0.11504 0.00406 0.10795 0.11187 0.00709

M45 - M66 0.11707 0.00325 0.11592 0.11746 0.00115

M45 - M67 0.10972 0.00500 0.10781 0.10700 0.00191

M45 - M68 0.06955 0.00132 0.06833 0.06977 0.00122

M45 - M69 0.11414 0.00945 0.11227 0.09143 0.00187

M45 - M70 0.10080 0.00259 0.09249 0.09631 0.00831

M45 - M71 0.08986 0.00107 0.08338 0.08805 0.00647

M45 - M72 0.07978 0.00497 0.07873 0.07060 0.00104

M45 - M73 0.09257 0.00242 0.09014 0.09926 0.00243

M45 - M74 0.08461 0.00066 0.08254 0.09055 0.00206

M45 - M75 0.07583 0.00062 0.07452 0.08134 0.00132

M45 - M76 0.15416 0.05197 0.10073 0.13766 0.05343

M45 - M77 0.09644 0.00197 0.09496 0.09742 0.00148

M45 - M78 0.09986 0.00106 0.09864 0.09368 0.00121

M45 - M79 0.09437 0.00316 0.09082 0.10013 0.00355

M45 - M80 0.07856 0.00142 0.07703 0.08107 0.00153

M45 - M81 0.09732 0.00177 0.09269 0.09142 0.00464

M45 - M82 0.11466 0.00579 0.10665 0.10872 0.00801

M45 - M83 0.12195 0.00087 0.11550 0.12346 0.00645

M45 - M84 0.08025 0.00504 0.07411 0.07562 0.00614

M45 - M85 0.09521 0.00038 0.09376 0.09696 0.00145

M45 - M86 0.13831 0.00002 0.13156 0.13856 0.00675

M45 - M87 0.09422 0.00083 0.09271 0.09841 0.00151

M45 - M88 0.13503 0.02167 0.13204 0.12375 0.00298

M45 - M89 0.14294 0.00513 0.14123 0.14065 0.00171

M45 - M90 0.10960 0.00270 0.10170 0.10951 0.00790

M45 - M91 0.07389 0.00106 0.07029 0.07583 0.00360

M45 - M92 0.11813 0.00259 0.10964 0.10752 0.00848

M45 - M93 0.08886 0.00156 0.08369 0.09251 0.00517

M45 - M94 0.09338 0.00161 0.09213 0.09383 0.00125

M45 - M95 0.10000 0.00454 0.09902 0.09173 0.00097

M45 - M96 0.09005 0.00329 0.08926 0.08693 0.00079

M45 - M97 0.10925 0.00277 0.10680 0.10556 0.00245

M46 - M47 0.09234 0.00130 0.08894 0.08856 0.00339

M46 - M48 0.11174 0.00133 0.10934 0.10844 0.00240

M46 - M49 0.10989 0.00177 0.10558 0.11052 0.00431

M46 - M50 0.07560 0.00218 0.07557 0.07066 0.00003

M46 - M51 0.01267 0.00139 0.01218 0.01055 0.00049

M46 - M52 0.02895 0.00178 0.02879 0.02378 0.00016

M46 - M53 0.09074 0.00216 0.08382 0.08680 0.00692

M46 - M54 0.06105 0.00097 0.06098 0.05937 0.00006

M46 - M55 0.03806 0.00284 0.03753 0.03157 0.00053

M46 - M56 0.03631 0.00176 0.03613 0.03021 0.00018

M46 - M57 0.02218 0.00046 0.02158 0.02177 0.00060

M46 - M58 0.01567 0.00014 0.01360 0.01573 0.00207

M46 - M59 0.04062 0.00150 0.03705 0.03817 0.00358

M46 - M60 0.03169 0.00041 0.03047 0.03017 0.00122

M46 - M61 0.05077 0.00141 0.04202 0.04876 0.00875

M46 - M62 0.01863 0.00137 0.01685 0.02028 0.00178

M46 - M63 0.01868 0.00101 0.01732 0.01972 0.00136

M46 - M64 0.04045 0.00090 0.04039 0.03674 0.00006

M46 - M65 0.06757 0.00158 0.06019 0.06332 0.00738

M46 - M66 0.07164 0.00093 0.07143 0.07091 0.00021

M46 - M67 0.07278 0.00154 0.07244 0.07186 0.00034

M46 - M68 0.02378 0.00085 0.02254 0.02318 0.00124

M46 - M69 0.03550 0.00060 0.03413 0.03496 0.00137

M46 - M70 0.05278 0.00062 0.04670 0.05167 0.00608

M46 - M71 0.05143 0.00137 0.04867 0.04538 0.00276

M46 - M72 0.02131 0.00039 0.02070 0.01970 0.00061

M46 - M73 0.01901 0.00029 0.01894 0.01923 0.00007

M46 - M74 0.01972 0.00009 0.01967 0.01903 0.00005

M46 - M75 0.01784 0.00025 0.01657 0.01761 0.00127

M46 - M76 0.07771 0.00031 0.07727 0.08129 0.00045

M46 - M77 0.03246 0.00032 0.02974 0.03216 0.00271

M46 - M78 0.01880 0.00048 0.01838 0.01919 0.00043

M46 - M79 0.04098 0.00096 0.04087 0.04228 0.00011

M46 - M80 0.02481 0.00042 0.02479 0.02574 0.00003

M46 - M81 0.03764 0.00085 0.03541 0.03704 0.00223

M46 - M82 0.05867 0.00061 0.05508 0.06139 0.00359

M46 - M83 0.08306 0.00055 0.08129 0.08277 0.00177

M46 - M84 0.03776 0.00189 0.03628 0.03217 0.00147

M46 - M85 0.02973 0.00029 0.02804 0.02700 0.00169

M46 - M86 0.08175 0.00047 0.07376 0.07762 0.00799

M46 - M87 0.02998 0.00037 0.02865 0.02747 0.00132

M46 - M88 0.07197 0.00057 0.07052 0.07297 0.00145

M46 - M89 0.09450 0.00094 0.09446 0.09335 0.00004

M46 - M90 0.06394 0.00080 0.06015 0.06149 0.00379

M46 - M91 0.01877 0.00067 0.01739 0.01676 0.00138

M46 - M92 0.06329 0.00086 0.05603 0.06107 0.00726

M46 - M93 0.02511 0.00087 0.02437 0.02095 0.00074

M46 - M94 0.04947 0.00072 0.04852 0.04495 0.00095

M46 - M95 0.05424 0.00073 0.05406 0.05193 0.00018

M46 - M96 0.05248 0.00064 0.05233 0.05068 0.00015

M46 - M97 0.06562 0.00170 0.06482 0.05842 0.00080

M47 - M48 0.20305 0.07964 0.10054 0.12327 0.10251

M47 - M49 0.22020 0.09791 0.10127 0.12523 0.11893

M47 - M50 0.11152 0.00338 0.10895 0.10794 0.00258

M47 - M51 0.05493 0.00480 0.05298 0.05088 0.00194

M47 - M52 0.06721 0.00600 0.06590 0.06073 0.00131

M47 - M53 0.11231 0.00540 0.10351 0.10950 0.00881

M47 - M54 0.09906 0.00419 0.09719 0.09650 0.00187

M47 - M55 0.06670 0.00470 0.06545 0.06376 0.00124

M47 - M56 0.06834 0.00233 0.06735 0.06612 0.00100

M47 - M57 0.06777 0.00617 0.06618 0.05970 0.00159

M47 - M58 0.09574 0.00209 0.09187 0.09241 0.00387

M47 - M59 0.09674 0.00522 0.09326 0.08924 0.00348

M47 - M60 0.09857 0.00320 0.09385 0.09460 0.00473

M47 - M61 0.10845 0.00682 0.10160 0.09829 0.00685

M47 - M62 0.09769 0.00223 0.09554 0.09535 0.00215

M47 - M63 0.09678 0.00216 0.09474 0.09508 0.00204

M47 - M64 0.07044 0.00327 0.06850 0.06894 0.00194

M47 - M65 0.11922 0.00240 0.11382 0.11344 0.00540

M47 - M66 0.11412 0.00622 0.11054 0.10824 0.00358

M47 - M67 0.10988 0.00645 0.10245 0.10614 0.00742

M47 - M68 0.06184 0.00646 0.06056 0.05842 0.00128

M47 - M69 0.09762 0.00160 0.09517 0.09322 0.00245

M47 - M70 0.09816 0.00336 0.09245 0.09414 0.00572

M47 - M71 0.07766 0.00793 0.07439 0.07299 0.00327

M47 - M72 0.06285 0.00105 0.06038 0.06062 0.00247

M47 - M73 0.07305 0.00025 0.07210 0.07243 0.00094

M47 - M74 0.09361 0.00181 0.09248 0.09305 0.00113

M47 - M75 0.07001 0.00209 0.06859 0.06678 0.00142

M47 - M76 0.10757 0.00104 0.10649 0.10797 0.00109

M47 - M77 0.07483 0.00381 0.07287 0.06744 0.00196

M47 - M78 0.09465 0.00190 0.09235 0.09114 0.00230

M47 - M79 0.10297 0.00262 0.09620 0.10051 0.00677

M47 - M80 0.07434 0.00877 0.07279 0.06303 0.00155

M47 - M81 0.08200 0.00370 0.07874 0.07478 0.00327

M47 - M82 0.09766 0.00809 0.09427 0.08687 0.00338

M47 - M83 0.10663 0.00529 0.10460 0.10044 0.00203

M47 - M84 0.07575 0.00719 0.07419 0.06910 0.00156

M47 - M85 0.07546 0.00160 0.07390 0.07440 0.00156

M47 - M86 0.09313 0.00096 0.08822 0.09248 0.00491

M47 - M87 0.07302 0.00142 0.07150 0.07237 0.00152

M47 - M88 0.09801 0.00648 0.09239 0.08753 0.00562

M47 - M89 0.10964 0.00393 0.10820 0.10652 0.00144

M47 - M90 0.10701 0.00326 0.10351 0.10310 0.00350

M47 - M91 0.09105 0.00428 0.08695 0.08475 0.00409

M47 - M92 0.08692 0.00379 0.08233 0.08004 0.00459

M47 - M93 0.09769 0.00285 0.09504 0.09403 0.00265

M47 - M94 0.11427 0.00660 0.11213 0.10093 0.00213

M47 - M95 0.08234 0.00316 0.07950 0.08088 0.00284

M47 - M96 0.08533 0.00361 0.08157 0.07799 0.00376

M47 - M97 0.08523 0.00216 0.08297 0.08563 0.00226

M48 - M49 0.21588 0.08597 0.11476 0.13785 0.10112

M48 - M50 0.12885 0.00320 0.12067 0.12142 0.00818

M48 - M51 0.06640 0.00305 0.06425 0.06228 0.00215

M48 - M52 0.07459 0.00433 0.07285 0.07330 0.00174

M48 - M53 0.12207 0.00347 0.11595 0.12085 0.00612

M48 - M54 0.10879 0.00175 0.10574 0.10870 0.00305

M48 - M55 0.08271 0.00499 0.08062 0.07537 0.00209

M48 - M56 0.08565 0.00368 0.08380 0.07788 0.00185

M48 - M57 0.07345 0.00252 0.07110 0.07055 0.00235

M48 - M58 0.11531 0.00177 0.11038 0.11076 0.00493

M48 - M59 0.10644 0.00404 0.10215 0.10269 0.00429

M48 - M60 0.11610 0.00301 0.11064 0.11164 0.00546

M48 - M61 0.11815 0.00479 0.11037 0.11317 0.00778

M48 - M62 0.11549 0.00371 0.11268 0.11341 0.00281

M48 - M63 0.11587 0.00395 0.11301 0.11318 0.00286

M48 - M64 0.08212 0.00381 0.08020 0.08032 0.00192

M48 - M65 0.13535 0.00209 0.12933 0.13132 0.00602

M48 - M66 0.12744 0.00299 0.12051 0.12306 0.00692

M48 - M67 0.12295 0.00423 0.11279 0.12022 0.01015

M48 - M68 0.07169 0.00460 0.06940 0.06895 0.00229

M48 - M69 0.11760 0.00416 0.11474 0.10804 0.00286

M48 - M70 0.10892 0.00420 0.10003 0.10668 0.00889

M48 - M71 0.09052 0.00544 0.08608 0.08467 0.00444

M48 - M72 0.07629 0.00370 0.07213 0.07302 0.00416

M48 - M73 0.09004 0.00069 0.08830 0.08915 0.00174

M48 - M74 0.11392 0.00202 0.11112 0.11305 0.00281

M48 - M75 0.08382 0.00213 0.08124 0.08298 0.00257

M48 - M76 0.12921 0.00363 0.12713 0.12785 0.00208

M48 - M77 0.08339 0.00271 0.08058 0.08070 0.00281

M48 - M78 0.11296 0.00142 0.10798 0.11050 0.00498

M48 - M79 0.12067 0.00175 0.11391 0.11948 0.00676

M48 - M80 0.07859 0.00356 0.07639 0.07584 0.00220

M48 - M81 0.09842 0.00312 0.09356 0.09103 0.00486

M48 - M82 0.10341 0.00244 0.09902 0.10165 0.00440

M48 - M83 0.11351 0.00271 0.10984 0.11537 0.00368

M48 - M84 0.09376 0.00741 0.09084 0.08207 0.00293

M48 - M85 0.09165 0.00152 0.08898 0.09146 0.00266

M48 - M86 0.10620 0.00108 0.10060 0.10651 0.00560

M48 - M87 0.08783 0.00137 0.08503 0.08866 0.00281

M48 - M88 0.11235 0.01225 0.10558 0.09919 0.00677

M48 - M89 0.12885 0.00296 0.12551 0.12257 0.00335

M48 - M90 0.12095 0.00325 0.11424 0.11629 0.00671

M48 - M91 0.10466 0.00246 0.09942 0.10054 0.00524

M48 - M92 0.10186 0.00432 0.09548 0.09244 0.00638

M48 - M93 0.11216 0.00074 0.10798 0.11123 0.00418

M48 - M94 0.12586 0.00360 0.12073 0.11855 0.00513

M48 - M95 0.09463 0.00322 0.09048 0.09472 0.00414

M48 - M96 0.09630 0.00588 0.09429 0.09157 0.00201

M48 - M97 0.10216 0.00275 0.09911 0.09842 0.00305

M49 - M50 0.12702 0.00341 0.12010 0.12018 0.00692

M49 - M51 0.06576 0.00250 0.06269 0.06338 0.00307

M49 - M52 0.07780 0.00521 0.07460 0.07349 0.00321

M49 - M53 0.12362 0.00312 0.11653 0.12137 0.00708

M49 - M54 0.11240 0.00450 0.10742 0.10922 0.00498

M49 - M55 0.08366 0.00558 0.08045 0.07586 0.00322

M49 - M56 0.09017 0.00523 0.08718 0.07898 0.00299

M49 - M57 0.07357 0.00339 0.07049 0.07063 0.00309

M49 - M58 0.11636 0.00229 0.10953 0.11035 0.00683

M49 - M59 0.10565 0.00555 0.10083 0.10249 0.00483

M49 - M60 0.11798 0.00484 0.11198 0.11007 0.00601

M49 - M61 0.11656 0.00602 0.10763 0.11322 0.00893

M49 - M62 0.11419 0.00269 0.11071 0.11351 0.00347

M49 - M63 0.11460 0.00303 0.11103 0.11330 0.00357

M49 - M64 0.08244 0.00247 0.07889 0.08256 0.00355

M49 - M65 0.13617 0.00271 0.12895 0.13131 0.00722

M49 - M66 0.12484 0.00375 0.11637 0.12204 0.00847

M49 - M67 0.12497 0.00543 0.11498 0.12077 0.01000

M49 - M68 0.07674 0.00591 0.07353 0.07096 0.00321

M49 - M69 0.11660 0.00373 0.11250 0.10818 0.00409

M49 - M70 0.11066 0.00491 0.10207 0.10719 0.00858

M49 - M71 0.08823 0.00555 0.08376 0.08571 0.00447

M49 - M72 0.07432 0.00128 0.06865 0.07352 0.00567

M49 - M73 0.09150 0.00018 0.08788 0.09165 0.00362

M49 - M74 0.11120 0.00143 0.10610 0.11370 0.00511

M49 - M75 0.08770 0.00242 0.08298 0.08635 0.00472

M49 - M76 0.13768 0.00537 0.12900 0.12401 0.00868

M49 - M77 0.09032 0.00430 0.08608 0.08293 0.00424

M49 - M78 0.11755 0.00174 0.11017 0.11119 0.00738

M49 - M79 0.12380 0.00194 0.11185 0.11995 0.01195

M49 - M80 0.08712 0.00809 0.08426 0.07652 0.00286

M49 - M81 0.09709 0.00222 0.09193 0.09171 0.00517

M49 - M82 0.10229 0.00438 0.09705 0.09889 0.00524

M49 - M83 0.11306 0.00338 0.10934 0.11458 0.00372

M49 - M84 0.09017 0.00561 0.08665 0.08151 0.00352

M49 - M85 0.09284 0.00114 0.08864 0.09438 0.00420

M49 - M86 0.11113 0.00055 0.10435 0.11116 0.00678

M49 - M87 0.08951 0.00095 0.08544 0.09182 0.00406

M49 - M88 0.10396 0.00717 0.09936 0.10016 0.00459

M49 - M89 0.12987 0.00564 0.12292 0.12225 0.00695

M49 - M90 0.11453 0.00289 0.10724 0.11468 0.00729

M49 - M91 0.10946 0.00444 0.10191 0.10190 0.00755

M49 - M92 0.10377 0.00375 0.09658 0.09543 0.00719

M49 - M93 0.11601 0.00208 0.11038 0.11136 0.00563

M49 - M94 0.12209 0.00385 0.11654 0.11894 0.00556

M49 - M95 0.09519 0.00495 0.08846 0.09511 0.00673

M49 - M96 0.09711 0.00545 0.09315 0.09210 0.00396

M49 - M97 0.10285 0.00299 0.09889 0.09910 0.00396

M50 - M51 0.05624 0.01156 0.05472 0.04269 0.00152

M50 - M52 0.05117 0.00254 0.05113 0.04996 0.00004

M50 - M53 0.11163 0.00690 0.10925 0.10399 0.00238

M50 - M54 0.09217 0.00735 0.09055 0.08554 0.00162

M50 - M55 0.06476 0.00537 0.06396 0.05742 0.00080

M50 - M56 0.07096 0.00618 0.06939 0.06399 0.00157

M50 - M57 0.05908 0.00724 0.05879 0.05189 0.00029

M50 - M58 0.07840 0.00135 0.07734 0.07766 0.00106

M50 - M59 0.07930 0.00528 0.07282 0.07633 0.00648

M50 - M60 0.08305 0.00338 0.08156 0.08240 0.00149

M50 - M61 0.08216 0.00180 0.07731 0.08282 0.00484

M50 - M62 0.08329 0.00351 0.08292 0.07911 0.00037

M50 - M63 0.08319 0.00365 0.08282 0.07847 0.00037

M50 - M64 0.06404 0.00678 0.06396 0.06215 0.00008

M50 - M65 0.10224 0.00640 0.09757 0.09354 0.00466

M50 - M66 0.10312 0.01131 0.10306 0.09184 0.00006

M50 - M67 0.09564 0.00443 0.09481 0.09202 0.00083

M50 - M68 0.05628 0.00553 0.05449 0.05189 0.00179

M50 - M69 0.09303 0.01158 0.09196 0.07779 0.00108

M50 - M70 0.08744 0.00691 0.08215 0.08236 0.00528

M50 - M71 0.06520 0.00471 0.06365 0.06463 0.00154

M50 - M72 0.05798 0.01033 0.05428 0.05004 0.00370

M50 - M73 0.06588 0.00041 0.06502 0.06487 0.00086

M50 - M74 0.06731 0.00148 0.06728 0.07020 0.00002

M50 - M75 0.05544 0.00161 0.05484 0.05695 0.00060

M50 - M76 0.11369 0.01376 0.11327 0.09919 0.00042

M50 - M77 0.06567 0.00154 0.06417 0.06321 0.00150

M50 - M78 0.07210 0.00238 0.07176 0.07058 0.00034

M50 - M79 0.08004 0.00337 0.07990 0.07780 0.00015

M50 - M80 0.05827 0.00590 0.05812 0.05506 0.00015

M50 - M81 0.07020 0.00824 0.06888 0.06130 0.00132

M50 - M82 0.09092 0.01661 0.08835 0.07472 0.00257

M50 - M83 0.10410 0.01406 0.10113 0.08679 0.00296

M50 - M84 0.08183 0.02269 0.08103 0.05779 0.00080

M50 - M85 0.06203 0.00093 0.06129 0.06084 0.00075

M50 - M86 0.09199 0.00079 0.08738 0.08945 0.00461

M50 - M87 0.06422 0.00102 0.06361 0.06217 0.00061

M50 - M88 0.09364 0.01034 0.09139 0.08397 0.00225

M50 - M89 0.09887 0.00357 0.09835 0.09383 0.00052

M50 - M90 0.09336 0.00345 0.08861 0.09121 0.00476

M50 - M91 0.07021 0.00271 0.06865 0.06612 0.00156

M50 - M92 0.07987 0.00335 0.07550 0.07423 0.00438

M50 - M93 0.07728 0.00094 0.07629 0.07783 0.00099

M50 - M94 0.08042 0.00491 0.07823 0.07786 0.00218

M50 - M95 0.07315 0.00427 0.07294 0.07190 0.00021

M50 - M96 0.07329 0.00843 0.07227 0.06650 0.00103

M50 - M97 0.08167 0.00461 0.07602 0.07750 0.00566

M51 - M52 0.08690 0.06467 0.04111 0.01869 0.04579

M51 - M53 0.05394 0.00399 0.05088 0.05024 0.00306

M51 - M54 0.05058 0.01504 0.05031 0.03517 0.00028

M51 - M55 0.06819 0.04309 0.06705 0.02206 0.00114

M51 - M56 0.05486 0.03213 0.05437 0.02112 0.00049

M51 - M57 0.04073 0.02818 0.03519 0.01288 0.00554

M51 - M58 0.01911 0.00319 0.01784 0.01505 0.00127

M51 - M59 0.03628 0.01340 0.03363 0.02367 0.00265

M51 - M60 0.02587 0.00514 0.02454 0.02216 0.00132

M51 - M61 0.04854 0.01674 0.04245 0.02995 0.00609

M51 - M62 0.02034 0.00261 0.01940 0.01714 0.00093

M51 - M63 0.01952 0.00239 0.01875 0.01654 0.00078

M51 - M64 0.04160 0.02667 0.04093 0.01885 0.00067

M51 - M65 0.04028 0.00371 0.03490 0.03909 0.00538

M51 - M66 0.05745 0.01013 0.05607 0.04287 0.00138

M51 - M67 0.05436 0.01082 0.05365 0.04149 0.00071

M51 - M68 0.03758 0.02635 0.03725 0.01183 0.00033

M51 - M69 0.03037 0.00339 0.02900 0.02497 0.00137

M51 - M70 0.04140 0.00913 0.03675 0.03141 0.00465

M51 - M71 0.04695 0.02264 0.04591 0.02584 0.00104

M51 - M72 0.04858 0.02402 0.04356 0.01949 0.00502

M51 - M73 0.04088 0.02014 0.03980 0.02881 0.00109

M51 - M74 0.01822 0.00106 0.01782 0.01818 0.00040

M51 - M75 0.03988 0.02219 0.03804 0.02545 0.00184

M51 - M76 0.07872 0.02262 0.07678 0.06105 0.00193

M51 - M77 0.03733 0.02220 0.03510 0.02456 0.00223

M51 - M78 0.02467 0.00440 0.02394 0.01836 0.00073

M51 - M79 0.03075 0.00443 0.03034 0.03001 0.00041

M51 - M80 0.03827 0.02468 0.03633 0.02026 0.00194

M51 - M81 0.04702 0.02235 0.04388 0.03341 0.00314

M51 - M82 0.06075 0.02595 0.05866 0.03796 0.00209

M51 - M83 0.07523 0.02747 0.07343 0.04778 0.00180

M51 - M84 0.04155 0.02131 0.03930 0.01993 0.00225

M51 - M85 0.04689 0.02266 0.04514 0.03099 0.00175

M51 - M86 0.05261 0.02385 0.05001 0.03486 0.00260

M51 - M87 0.04654 0.02300 0.04480 0.03061 0.00173

M51 - M88 0.06146 0.02689 0.05992 0.03969 0.00154

M51 - M89 0.09944 0.03036 0.09685 0.06192 0.00258

M51 - M90 0.06225 0.01721 0.05800 0.03960 0.00425

M51 - M91 0.02269 0.00824 0.02152 0.01540 0.00117

M51 - M92 0.05184 0.02905 0.04932 0.03073 0.00253

M51 - M93 0.02175 0.00139 0.02075 0.01875 0.00100

M51 - M94 0.03124 0.00433 0.03027 0.02833 0.00098

M51 - M95 0.04867 0.02503 0.04330 0.02661 0.00537

M51 - M96 0.05645 0.02950 0.05538 0.02804 0.00107

M51 - M97 0.05314 0.02515 0.05182 0.03107 0.00131

M52 - M53 0.06631 0.01022 0.06318 0.05696 0.00313

M52 - M54 0.06395 0.02089 0.06361 0.04236 0.00035

M52 - M55 0.06946 0.04184 0.05611 0.02938 0.01335

M52 - M56 0.05341 0.02529 0.04715 0.02909 0.00625

M52 - M57 0.04965 0.02924 0.04768 0.02079 0.00197

M52 - M58 0.03373 0.00375 0.03283 0.02737 0.00090

M52 - M59 0.03984 0.00731 0.03827 0.03158 0.00157

M52 - M60 0.03930 0.00524 0.03846 0.03133 0.00084

M52 - M61 0.05332 0.00972 0.04852 0.03941 0.00480

M52 - M62 0.02954 0.00323 0.02872 0.02870 0.00082

M52 - M63 0.02765 0.00272 0.02700 0.02808 0.00065

M52 - M64 0.05928 0.03088 0.05908 0.02879 0.00020

M52 - M65 0.05479 0.00299 0.05029 0.04873 0.00449

M52 - M66 0.05303 0.00309 0.05292 0.04952 0.00011

M52 - M67 0.05130 0.00195 0.05106 0.04899 0.00024

M52 - M68 0.05252 0.03088 0.04658 0.02036 0.00594

M52 - M69 0.04494 0.00737 0.04369 0.03568 0.00126

M52 - M70 0.04978 0.00998 0.04597 0.03881 0.00381

M52 - M71 0.06529 0.03076 0.05816 0.03390 0.00714

M52 - M72 0.05398 0.02491 0.05265 0.02787 0.00133

M52 - M73 0.06458 0.01937 0.06232 0.04345 0.00226

M52 - M74 0.02835 0.00187 0.02826 0.03040 0.00010

M52 - M75 0.06417 0.02490 0.06234 0.03869 0.00183

M52 - M76 0.09112 0.01984 0.09110 0.06994 0.00002

M52 - M77 0.06043 0.02390 0.05840 0.03639 0.00203

M52 - M78 0.03704 0.00317 0.03632 0.03101 0.00072

M52 - M79 0.04442 0.00689 0.04330 0.03884 0.00112

M52 - M80 0.05468 0.02701 0.05387 0.02932 0.00080

M52 - M81 0.06861 0.02366 0.06271 0.04273 0.00590

M52 - M82 0.07090 0.02487 0.06947 0.04396 0.00142

M52 - M83 0.08090 0.02503 0.07931 0.05493 0.00159

M52 - M84 0.05286 0.02521 0.05227 0.02781 0.00059

M52 - M85 0.07055 0.02641 0.06816 0.04408 0.00239

M52 - M86 0.07633 0.02906 0.07261 0.04721 0.00372

M52 - M87 0.07155 0.02631 0.06947 0.04280 0.00208

M52 - M88 0.07756 0.03123 0.07359 0.04700 0.00397

M52 - M89 0.09377 0.02297 0.09173 0.07006 0.00204

M52 - M90 0.05519 0.00778 0.05275 0.04556 0.00244

M52 - M91 0.03897 0.00940 0.03815 0.02435 0.00082

M52 - M92 0.06464 0.02719 0.06083 0.04060 0.00381

M52 - M93 0.03370 0.00111 0.03248 0.03078 0.00123

M52 - M94 0.05543 0.01221 0.05280 0.03920 0.00264

M52 - M95 0.06000 0.02558 0.05971 0.03366 0.00029

M52 - M96 0.05770 0.02345 0.05728 0.03525 0.00043

M52 - M97 0.06517 0.02367 0.06349 0.04094 0.00169

M53 - M54 0.19012 0.08716 0.13156 0.09451 0.05856

M53 - M55 0.10519 0.03110 0.08205 0.06727 0.02315

M53 - M56 0.09671 0.01962 0.08390 0.07143 0.01281

M53 - M57 0.06288 0.00999 0.06032 0.05270 0.00256

M53 - M58 0.08713 0.00183 0.07963 0.08194 0.00750

M53 - M59 0.09175 0.00490 0.08318 0.08364 0.00857

M53 - M60 0.09715 0.00560 0.08987 0.08486 0.00728

M53 - M61 0.09769 0.00699 0.08618 0.08989 0.01151

M53 - M62 0.08679 0.00155 0.08063 0.08427 0.00616

M53 - M63 0.08655 0.00176 0.08000 0.08428 0.00655

M53 - M64 0.06592 0.00692 0.06363 0.05962 0.00230

M53 - M65 0.10245 0.00647 0.09411 0.09757 0.00834

M53 - M66 0.11404 0.00659 0.11040 0.10430 0.00364

M53 - M67 0.11031 0.00967 0.10625 0.10006 0.00406

M53 - M68 0.05581 0.00330 0.05303 0.05339 0.00278

M53 - M69 0.09561 0.01406 0.09219 0.08488 0.00342

M53 - M70 0.09659 0.00704 0.09059 0.09101 0.00600

M53 - M71 0.06937 0.00469 0.06630 0.06744 0.00308

M53 - M72 0.06822 0.00600 0.06447 0.06153 0.00374

M53 - M73 0.07769 0.00081 0.07412 0.07883 0.00356

M53 - M74 0.08528 0.00127 0.07914 0.08351 0.00614

M53 - M75 0.07473 0.00407 0.07105 0.07407 0.00368

M53 - M76 0.11679 0.00515 0.11419 0.10977 0.00259

M53 - M77 0.07030 0.00502 0.06717 0.06886 0.00314

M53 - M78 0.08772 0.00140 0.08152 0.08590 0.00620

M53 - M79 0.09924 0.00457 0.09349 0.09262 0.00576

M53 - M80 0.06090 0.00388 0.05837 0.05907 0.00254

M53 - M81 0.08573 0.00710 0.08106 0.07553 0.00467

M53 - M82 0.08281 0.00550 0.07708 0.07693 0.00573

M53 - M83 0.09531 0.00283 0.09265 0.09040 0.00267

M53 - M84 0.06718 0.00901 0.06444 0.05815 0.00274

M53 - M85 0.09025 0.00658 0.08589 0.08396 0.00437

M53 - M86 0.09472 0.00719 0.08995 0.08978 0.00477

M53 - M87 0.09278 0.00702 0.08788 0.08672 0.00490

M53 - M88 0.09585 0.01327 0.09175 0.08125 0.00410

M53 - M89 0.11821 0.00861 0.11240 0.10574 0.00581

M53 - M90 0.10025 0.00538 0.09441 0.09709 0.00584

M53 - M91 0.08487 0.00550 0.08016 0.08143 0.00472

M53 - M92 0.07661 0.00343 0.07147 0.07150 0.00513

M53 - M93 0.09680 0.00188 0.08914 0.09415 0.00766

M53 - M94 0.10125 0.00587 0.09354 0.09286 0.00771

M53 - M95 0.07348 0.00368 0.07080 0.06813 0.00268

M53 - M96 0.08620 0.01691 0.08356 0.07073 0.00264

M53 - M97 0.08400 0.01270 0.08067 0.07205 0.00333

M54 - M55 0.09210 0.03679 0.05255 0.04770 0.03955

M54 - M56 0.07094 0.01444 0.06360 0.04837 0.00734

M54 - M57 0.05134 0.01228 0.05130 0.03770 0.00003

M54 - M58 0.06483 0.00191 0.06364 0.06049 0.00119

M54 - M59 0.07429 0.00748 0.07251 0.06660 0.00178

M54 - M60 0.07163 0.00329 0.07074 0.06482 0.00090

M54 - M61 0.08401 0.01003 0.07937 0.07291 0.00465

M54 - M62 0.06728 0.00210 0.06684 0.06143 0.00043

M54 - M63 0.06577 0.00190 0.06533 0.06122 0.00044

M54 - M64 0.05492 0.00816 0.05481 0.04426 0.00011

M54 - M65 0.08634 0.00454 0.08034 0.08260 0.00600

M54 - M66 0.09860 0.01014 0.09216 0.08594 0.00644

M54 - M67 0.09297 0.00887 0.08858 0.08589 0.00439

M54 - M68 0.04519 0.00652 0.04484 0.03867 0.00036

M54 - M69 0.08318 0.01595 0.08082 0.06920 0.00236

M54 - M70 0.08268 0.00705 0.07545 0.07528 0.00724

M54 - M71 0.05544 0.00572 0.05496 0.04957 0.00048

M54 - M72 0.04952 0.00519 0.04495 0.04536 0.00457

M54 - M73 0.06105 0.00038 0.05864 0.06124 0.00241

M54 - M74 0.06699 0.00100 0.06659 0.06477 0.00040

M54 - M75 0.06217 0.00640 0.06085 0.05611 0.00132

M54 - M76 0.09452 0.00423 0.09254 0.08912 0.00197

M54 - M77 0.05799 0.00716 0.05643 0.05230 0.00156

M54 - M78 0.06609 0.00120 0.06583 0.06572 0.00026

M54 - M79 0.07946 0.00267 0.07909 0.07495 0.00037

M54 - M80 0.05465 0.01237 0.04941 0.04500 0.00524

M54 - M81 0.07111 0.00771 0.06719 0.05949 0.00392

M54 - M82 0.07537 0.01340 0.07178 0.06265 0.00359

M54 - M83 0.07891 0.00456 0.07704 0.07184 0.00187

M54 - M84 0.06152 0.01564 0.06017 0.04495 0.00134

M54 - M85 0.07607 0.00978 0.07430 0.06348 0.00177

M54 - M86 0.08057 0.01165 0.07797 0.06758 0.00261

M54 - M87 0.07523 0.00977 0.07344 0.06342 0.00179

M54 - M88 0.08408 0.01282 0.08333 0.06622 0.00075

M54 - M89 0.09288 0.00847 0.09273 0.08581 0.00015

M54 - M90 0.09014 0.00622 0.08637 0.08323 0.00377

M54 - M91 0.07431 0.00862 0.07327 0.06229 0.00104

M54 - M92 0.06919 0.00870 0.06682 0.05833 0.00237

M54 - M93 0.07265 0.00301 0.07153 0.06844 0.00112

M54 - M94 0.09008 0.01050 0.08883 0.07603 0.00125

M54 - M95 0.05882 0.00552 0.05780 0.05169 0.00102

M54 - M96 0.05937 0.00756 0.05910 0.05273 0.00027

M54 - M97 0.06215 0.00691 0.06201 0.05671 0.00014

M55 - M56 0.11771 0.07630 0.04056 0.04063 0.07715

M55 - M57 0.05476 0.02993 0.05445 0.02620 0.00031

M55 - M58 0.03391 0.00214 0.03263 0.03441 0.00128

M55 - M59 0.04746 0.01052 0.04376 0.03718 0.00370

M55 - M60 0.04270 0.00636 0.04098 0.03942 0.00172

M55 - M61 0.05501 0.01173 0.04955 0.04353 0.00546

M55 - M62 0.04167 0.00222 0.04081 0.03587 0.00086

M55 - M63 0.04209 0.00260 0.04105 0.03560 0.00105

M55 - M64 0.05558 0.02114 0.05483 0.03172 0.00075

M55 - M65 0.05746 0.00810 0.05268 0.05300 0.00477

M55 - M66 0.06718 0.01022 0.06528 0.05504 0.00190

M55 - M67 0.06713 0.01292 0.06658 0.05411 0.00055

M55 - M68 0.04897 0.02625 0.04668 0.02553 0.00229

M55 - M69 0.04195 0.00631 0.04062 0.03832 0.00134

M55 - M70 0.04815 0.00533 0.04409 0.04379 0.00406

M55 - M71 0.06442 0.02580 0.05859 0.03906 0.00584

M55 - M72 0.05308 0.02220 0.05102 0.03294 0.00206

M55 - M73 0.07347 0.01926 0.07217 0.04869 0.00130

M55 - M74 0.03395 0.00129 0.03364 0.03275 0.00031

M55 - M75 0.06836 0.02129 0.06560 0.04325 0.00276

M55 - M76 0.11177 0.02550 0.11095 0.08043 0.00082

M55 - M77 0.06817 0.02526 0.06508 0.03925 0.00309

M55 - M78 0.03823 0.00391 0.03736 0.03404 0.00087

M55 - M79 0.04677 0.00439 0.04607 0.04179 0.00070

M55 - M80 0.05967 0.02623 0.05360 0.03156 0.00607

M55 - M81 0.07890 0.02362 0.07493 0.04585 0.00397

M55 - M82 0.07365 0.02607 0.07140 0.05032 0.00225

M55 - M83 0.08215 0.02136 0.08149 0.06127 0.00066

M55 - M84 0.05764 0.02441 0.05715 0.03152 0.00049

M55 - M85 0.07667 0.02166 0.07283 0.05073 0.00384

M55 - M86 0.07990 0.02186 0.07632 0.05478 0.00358

M55 - M87 0.07817 0.02150 0.07542 0.05213 0.00274

M55 - M88 0.08500 0.02713 0.07515 0.05643 0.00986

M55 - M89 0.10328 0.02641 0.10242 0.07655 0.00086

M55 - M90 0.06172 0.01237 0.05859 0.05131 0.00313

M55 - M91 0.03542 0.00461 0.03439 0.03054 0.00104

M55 - M92 0.07413 0.02583 0.06746 0.04318 0.00668

M55 - M93 0.04179 0.00125 0.04064 0.04109 0.00114

M55 - M94 0.05185 0.00608 0.05054 0.04445 0.00131

M55 - M95 0.06882 0.02455 0.06768 0.04126 0.00114

M55 - M96 0.07387 0.02832 0.07171 0.04216 0.00217

M55 - M97 0.07032 0.02234 0.06882 0.04624 0.00151

M56 - M57 0.05506 0.03283 0.05390 0.02584 0.00116

M56 - M58 0.03441 0.00174 0.03331 0.03286 0.00110

M56 - M59 0.03656 0.00484 0.03456 0.03487 0.00199

M56 - M60 0.04021 0.00583 0.03855 0.03915 0.00165

M56 - M61 0.04212 0.00369 0.03795 0.04099 0.00417

M56 - M62 0.03646 0.00155 0.03588 0.03594 0.00058

M56 - M63 0.03599 0.00172 0.03542 0.03507 0.00057

M56 - M64 0.05973 0.02060 0.05910 0.03190 0.00062

M56 - M65 0.05749 0.00317 0.05289 0.05500 0.00460

M56 - M66 0.07036 0.00903 0.06803 0.06104 0.00233

M56 - M67 0.06552 0.01164 0.06515 0.05520 0.00037

M56 - M68 0.05456 0.02690 0.05180 0.02423 0.00275

M56 - M69 0.04707 0.00675 0.04582 0.03938 0.00125

M56 - M70 0.04535 0.00370 0.04150 0.04427 0.00385

M56 - M71 0.06694 0.02166 0.06030 0.04218 0.00664

M56 - M72 0.05008 0.02551 0.04902 0.03202 0.00106

M56 - M73 0.08378 0.01955 0.08048 0.04619 0.00330

M56 - M74 0.03235 0.00095 0.03148 0.03063 0.00088

M56 - M75 0.07744 0.02059 0.07149 0.03965 0.00594

M56 - M76 0.13309 0.02841 0.13194 0.08721 0.00114

M56 - M77 0.07776 0.02397 0.07575 0.04064 0.00201

M56 - M78 0.03438 0.00188 0.03336 0.03182 0.00102

M56 - M79 0.04804 0.00381 0.04717 0.04276 0.00087

M56 - M80 0.06766 0.02513 0.06684 0.03521 0.00082

M56 - M81 0.08003 0.02145 0.07449 0.04675 0.00554

M56 - M82 0.08232 0.02504 0.08021 0.05887 0.00212

M56 - M83 0.09498 0.02306 0.09454 0.07233 0.00044

M56 - M84 0.06255 0.02429 0.06174 0.03658 0.00081

M56 - M85 0.08361 0.01963 0.07732 0.04698 0.00629

M56 - M86 0.08834 0.01975 0.08225 0.05321 0.00610

M56 - M87 0.08676 0.02002 0.08108 0.04873 0.00568

M56 - M88 0.09062 0.02148 0.08557 0.06105 0.00505

M56 - M89 0.11005 0.02700 0.10971 0.08262 0.00034

M56 - M90 0.06782 0.01268 0.06471 0.05655 0.00311

M56 - M91 0.03368 0.00397 0.03254 0.02784 0.00115

M56 - M92 0.07904 0.02272 0.07251 0.04466 0.00653

M56 - M93 0.04601 0.00111 0.04511 0.04267 0.00090

M56 - M94 0.04601 0.00220 0.04424 0.04278 0.00177

M56 - M95 0.07944 0.03019 0.07890 0.04491 0.00054

M56 - M96 0.07016 0.02396 0.06982 0.04513 0.00035

M56 - M97 0.08484 0.02259 0.08337 0.05249 0.00148

M57 - M58 0.03514 0.00929 0.02950 0.02538 0.00564

M57 - M59 0.04064 0.00999 0.03361 0.03187 0.00703

M57 - M60 0.04078 0.00755 0.03935 0.03097 0.00143

M57 - M61 0.04526 0.00708 0.03843 0.03872 0.00683

M57 - M62 0.03252 0.00452 0.03063 0.02784 0.00189

M57 - M63 0.03177 0.00444 0.03006 0.02722 0.00171

M57 - M64 0.04158 0.00872 0.04137 0.03195 0.00020

M57 - M65 0.04961 0.00294 0.04433 0.04720 0.00528

M57 - M66 0.05894 0.00873 0.05875 0.04872 0.00020

M57 - M67 0.06611 0.01777 0.06527 0.04970 0.00084

M57 - M68 0.03148 0.01045 0.03074 0.02350 0.00074

M57 - M69 0.04251 0.00903 0.04147 0.03373 0.00103

M57 - M70 0.04746 0.00646 0.04322 0.03986 0.00424

M57 - M71 0.03810 0.00653 0.03666 0.03505 0.00144

M57 - M72 0.03323 0.01313 0.03261 0.02277 0.00061

M57 - M73 0.04779 0.01544 0.04641 0.02729 0.00138

M57 - M74 0.02567 0.00085 0.02521 0.02599 0.00046

M57 - M75 0.05318 0.02047 0.05239 0.02443 0.00080

M57 - M76 0.08156 0.01920 0.08089 0.06104 0.00067

M57 - M77 0.05295 0.02334 0.04933 0.02659 0.00362

M57 - M78 0.02783 0.00129 0.02690 0.02705 0.00093

M57 - M79 0.03528 0.00389 0.03519 0.03016 0.00010

M57 - M80 0.04679 0.02408 0.04544 0.02106 0.00135

M57 - M81 0.06065 0.02150 0.05649 0.03226 0.00416

M57 - M82 0.07375 0.02302 0.07063 0.04395 0.00311

M57 - M83 0.08365 0.02127 0.08162 0.05573 0.00203

M57 - M84 0.06306 0.02910 0.06225 0.02857 0.00080

M57 - M85 0.05816 0.02072 0.05728 0.03058 0.00088

M57 - M86 0.06415 0.00882 0.06003 0.05649 0.00412

M57 - M87 0.05769 0.02042 0.05681 0.03024 0.00089

M57 - M88 0.05677 0.00707 0.05536 0.05091 0.00141

M57 - M89 0.07100 0.00760 0.07084 0.06577 0.00016

M57 - M90 0.05289 0.00372 0.04957 0.04628 0.00331

M57 - M91 0.03542 0.00895 0.03449 0.02581 0.00093

M57 - M92 0.05873 0.01315 0.05498 0.04700 0.00376

M57 - M93 0.03986 0.00398 0.03937 0.03294 0.00050

M57 - M94 0.04113 0.00322 0.03953 0.03800 0.00160

M57 - M95 0.05147 0.01477 0.04850 0.03916 0.00297

M57 - M96 0.04533 0.01265 0.04480 0.03714 0.00054

M57 - M97 0.05243 0.00861 0.05167 0.04482 0.00076

M58 - M59 0.05527 0.01200 0.03405 0.04567 0.02122

M58 - M60 0.06629 0.02935 0.02515 0.04483 0.04114

M58 - M61 0.08082 0.02219 0.03691 0.05718 0.04391

M58 - M62 0.03915 0.00182 0.03616 0.03490 0.00299

M58 - M63 0.03878 0.00191 0.03651 0.03353 0.00227

M58 - M64 0.04213 0.00181 0.04122 0.04070 0.00091

M58 - M65 0.07804 0.00223 0.06361 0.07404 0.01443

M58 - M66 0.06514 0.00107 0.06386 0.06723 0.00128

M58 - M67 0.07646 0.00308 0.07520 0.07555 0.00125

M58 - M68 0.02832 0.00274 0.02700 0.02627 0.00131

M58 - M69 0.04233 0.00125 0.03865 0.04350 0.00369

M58 - M70 0.05721 0.00132 0.04852 0.05779 0.00869

M58 - M71 0.04537 0.00135 0.04173 0.04410 0.00364

M58 - M72 0.02264 0.00105 0.02137 0.02266 0.00127

M58 - M73 0.02920 0.00038 0.02809 0.02820 0.00112

M58 - M74 0.02931 0.00035 0.02773 0.02777 0.00158

M58 - M75 0.02437 0.00055 0.02292 0.02383 0.00145

M58 - M76 0.08214 0.00037 0.08046 0.08197 0.00168

M58 - M77 0.03731 0.00123 0.03447 0.03562 0.00284

M58 - M78 0.02791 0.00139 0.02669 0.02497 0.00122

M58 - M79 0.04532 0.00068 0.04408 0.04684 0.00124

M58 - M80 0.03026 0.00190 0.02940 0.03209 0.00086

M58 - M81 0.04035 0.00094 0.03787 0.03743 0.00248

M58 - M82 0.05921 0.00165 0.05368 0.06161 0.00553

M58 - M83 0.07836 0.00109 0.07609 0.07734 0.00228

M58 - M84 0.03965 0.00244 0.03633 0.03833 0.00331

M58 - M85 0.02992 0.00043 0.02837 0.02863 0.00155

M58 - M86 0.07391 0.00056 0.06801 0.07222 0.00590

M58 - M87 0.02929 0.00034 0.02785 0.02834 0.00144

M58 - M88 0.06867 0.00172 0.06659 0.06710 0.00208

M58 - M89 0.09131 0.00092 0.09046 0.08766 0.00085

M58 - M90 0.07267 0.00290 0.06350 0.06799 0.00917

M58 - M91 0.02689 0.00153 0.02177 0.02672 0.00511

M58 - M92 0.06540 0.00165 0.06092 0.06103 0.00448

M58 - M93 0.03006 0.00072 0.02728 0.03074 0.00278

M58 - M94 0.05456 0.00223 0.05309 0.05134 0.00148

M58 - M95 0.06129 0.00220 0.06014 0.05752 0.00115

M58 - M96 0.05227 0.00138 0.05117 0.05067 0.00111

M58 - M97 0.06565 0.00149 0.06384 0.06116 0.00182

M59 - M60 0.08189 0.02939 0.04546 0.05343 0.03643

M59 - M61 0.16625 0.09421 0.05211 0.06483 0.11414

M59 - M62 0.05493 0.01114 0.03896 0.04755 0.01597

M59 - M63 0.05410 0.01074 0.03826 0.04680 0.01584

M59 - M64 0.04770 0.00700 0.04319 0.04342 0.00450

M59 - M65 0.07794 0.00309 0.06839 0.07390 0.00955

M59 - M66 0.09155 0.01725 0.08943 0.07239 0.00212

M59 - M67 0.10834 0.02226 0.10441 0.07807 0.00393

M59 - M68 0.04194 0.00763 0.03627 0.03395 0.00567

M59 - M69 0.06014 0.00776 0.05556 0.05366 0.00458

M59 - M70 0.07071 0.00488 0.06060 0.06549 0.01011

M59 - M71 0.05069 0.00849 0.04778 0.04567 0.00292

M59 - M72 0.03992 0.01012 0.03513 0.03120 0.00479

M59 - M73 0.03928 0.00167 0.03765 0.03598 0.00163

M59 - M74 0.05238 0.00357 0.04884 0.04626 0.00354

M59 - M75 0.04263 0.00445 0.04001 0.03512 0.00261

M59 - M76 0.07900 0.00677 0.07723 0.06794 0.00177

M59 - M77 0.04165 0.00446 0.03580 0.03739 0.00585

M59 - M78 0.04603 0.00226 0.04173 0.04259 0.00430

M59 - M79 0.06115 0.00155 0.05864 0.05947 0.00251

M59 - M80 0.03528 0.00472 0.03248 0.03310 0.00280

M59 - M81 0.04864 0.00266 0.04624 0.04240 0.00240

M59 - M82 0.05709 0.00379 0.05345 0.05512 0.00364

M59 - M83 0.07938 0.00719 0.07712 0.06731 0.00226

M59 - M84 0.04121 0.00440 0.03941 0.03833 0.00180

M59 - M85 0.04380 0.00355 0.04166 0.03896 0.00214

M59 - M86 0.07539 0.00190 0.06888 0.07201 0.00651

M59 - M87 0.04346 0.00371 0.04151 0.03921 0.00195

M59 - M88 0.06972 0.00615 0.06717 0.06326 0.00255

M59 - M89 0.07832 0.00233 0.07635 0.07665 0.00197

M59 - M90 0.06969 0.00357 0.06498 0.06918 0.00471

M59 - M91 0.04877 0.00577 0.04661 0.04351 0.00216

M59 - M92 0.07035 0.00774 0.06548 0.06061 0.00486

M59 - M93 0.04629 0.00096 0.04445 0.04547 0.00184

M59 - M94 0.07899 0.00868 0.07192 0.06541 0.00707

M59 - M95 0.06575 0.00868 0.06132 0.05356 0.00443

M59 - M96 0.07263 0.01613 0.06707 0.05038 0.00556

M59 - M97 0.06143 0.00681 0.05481 0.05440 0.00662

M60 - M61 0.09837 0.03623 0.04468 0.06220 0.05369

M60 - M62 0.04582 0.00386 0.03928 0.04478 0.00654

M60 - M63 0.04507 0.00331 0.03973 0.04377 0.00535

M60 - M64 0.04363 0.00127 0.04286 0.04418 0.00076

M60 - M65 0.08108 0.00168 0.06818 0.07797 0.01291

M60 - M66 0.07791 0.00329 0.07686 0.07098 0.00105

M60 - M67 0.09346 0.00649 0.09175 0.07887 0.00171

M60 - M68 0.03557 0.00271 0.03415 0.03225 0.00142

M60 - M69 0.05385 0.00535 0.04985 0.05172 0.00400

M60 - M70 0.06462 0.00265 0.05401 0.06403 0.01060

M60 - M71 0.05369 0.00603 0.04878 0.04690 0.00491

M60 - M72 0.03056 0.00670 0.02889 0.02874 0.00166

M60 - M73 0.04038 0.00026 0.03955 0.03894 0.00082

M60 - M74 0.04269 0.00095 0.04135 0.03992 0.00134

M60 - M75 0.03878 0.00167 0.03726 0.03454 0.00152

M60 - M76 0.10341 0.00978 0.10252 0.08261 0.00089

M60 - M77 0.04632 0.00283 0.04328 0.04184 0.00304

M60 - M78 0.04149 0.00209 0.04016 0.03674 0.00133

M60 - M79 0.05590 0.00081 0.05447 0.05479 0.00143

M60 - M80 0.03941 0.00340 0.03843 0.03800 0.00098

M60 - M81 0.04552 0.00312 0.04373 0.04306 0.00180

M60 - M82 0.06971 0.00486 0.06290 0.06356 0.00681

M60 - M83 0.08117 0.00279 0.07826 0.07418 0.00292

M60 - M84 0.04551 0.00523 0.03801 0.04201 0.00750

M60 - M85 0.03625 0.00058 0.03466 0.03547 0.00159

M60 - M86 0.07511 0.00116 0.06940 0.07466 0.00571

M60 - M87 0.03559 0.00027 0.03416 0.03596 0.00143

M60 - M88 0.07593 0.00582 0.07340 0.06757 0.00253

M60 - M89 0.08894 0.00215 0.08798 0.08487 0.00096

M60 - M90 0.07971 0.00605 0.07317 0.07378 0.00654

M60 - M91 0.03616 0.00146 0.03335 0.03631 0.00281

M60 - M92 0.07387 0.00415 0.07014 0.06699 0.00373

M60 - M93 0.04354 0.00161 0.04174 0.04124 0.00180

M60 - M94 0.06101 0.00190 0.05927 0.06071 0.00173

M60 - M95 0.07026 0.00579 0.06653 0.06017 0.00373

M60 - M96 0.05983 0.00452 0.05812 0.05288 0.00171

M60 - M97 0.07064 0.00376 0.06623 0.06219 0.00441

M61 - M62 0.06582 0.00853 0.04261 0.05991 0.02321

M61 - M63 0.06291 0.00677 0.04196 0.05924 0.02095

M61 - M64 0.05435 0.00454 0.05036 0.05321 0.00399

M61 - M65 0.09265 0.00365 0.07682 0.08661 0.01583

M61 - M66 0.10011 0.01855 0.09576 0.07947 0.00435

M61 - M67 0.10940 0.02190 0.10521 0.08628 0.00419

M61 - M68 0.04339 0.00408 0.03799 0.04078 0.00539

M61 - M69 0.06395 0.00313 0.05752 0.06210 0.00643

M61 - M70 0.08310 0.00761 0.07080 0.07286 0.01229

M61 - M71 0.06315 0.00852 0.05652 0.05295 0.00663

M61 - M72 0.04375 0.00562 0.03789 0.03788 0.00586

M61 - M73 0.04865 0.00068 0.04351 0.04775 0.00514

M61 - M74 0.06387 0.00341 0.05378 0.05728 0.01009

M61 - M75 0.04903 0.00361 0.04435 0.04512 0.00468

M61 - M76 0.08013 0.00383 0.07516 0.07634 0.00497

M61 - M77 0.04432 0.00223 0.03891 0.04382 0.00541

M61 - M78 0.05833 0.00365 0.04976 0.05136 0.00857

M61 - M79 0.06988 0.00288 0.06396 0.06819 0.00592

M61 - M80 0.04013 0.00481 0.03662 0.03943 0.00351

M61 - M81 0.05509 0.00328 0.05061 0.05168 0.00448

M61 - M82 0.06623 0.00793 0.06049 0.06193 0.00574

M61 - M83 0.08348 0.00349 0.07877 0.07695 0.00470

M61 - M84 0.04660 0.00241 0.04206 0.04571 0.00454

M61 - M85 0.05831 0.00377 0.05388 0.05183 0.00442

M61 - M86 0.08864 0.00218 0.08123 0.08438 0.00741

M61 - M87 0.05887 0.00428 0.05450 0.05099 0.00437

M61 - M88 0.08236 0.00676 0.07678 0.07114 0.00558

M61 - M89 0.08948 0.00424 0.08510 0.08751 0.00437

M61 - M90 0.08475 0.00679 0.06942 0.07620 0.01533

M61 - M91 0.06031 0.00491 0.05414 0.05472 0.00618

M61 - M92 0.07378 0.00611 0.06661 0.06830 0.00717

M61 - M93 0.05778 0.00159 0.05062 0.05606 0.00717

M61 - M94 0.08974 0.01078 0.07816 0.07299 0.01158

M61 - M95 0.06735 0.00524 0.06350 0.06240 0.00385

M61 - M96 0.07456 0.01002 0.07077 0.05894 0.00379

M61 - M97 0.07010 0.00467 0.06420 0.06359 0.00590

M62 - M63 0.07037 0.05132 0.01739 0.03880 0.05297

M62 - M64 0.04740 0.00170 0.04693 0.04478 0.00047

M62 - M65 0.08374 0.00243 0.07292 0.07698 0.01082

M62 - M66 0.07486 0.00578 0.07391 0.07108 0.00096

M62 - M67 0.07851 0.00287 0.07783 0.07749 0.00068

M62 - M68 0.03095 0.00188 0.02847 0.02843 0.00248

M62 - M69 0.05187 0.00435 0.04683 0.04650 0.00504

M62 - M70 0.06439 0.00540 0.05752 0.05989 0.00687

M62 - M71 0.04988 0.00255 0.04538 0.04721 0.00449

M62 - M72 0.02552 0.00132 0.02424 0.02433 0.00129

M62 - M73 0.03106 0.00025 0.03063 0.03004 0.00042

M62 - M74 0.03542 0.00113 0.03503 0.03024 0.00040

M62 - M75 0.02709 0.00052 0.02572 0.02539 0.00137

M62 - M76 0.07551 0.00037 0.07493 0.07798 0.00059

M62 - M77 0.03767 0.00085 0.03459 0.03647 0.00308

M62 - M78 0.02584 0.00041 0.02499 0.02668 0.00085

M62 - M79 0.04825 0.00123 0.04783 0.04830 0.00042

M62 - M80 0.03562 0.00212 0.03528 0.03286 0.00034

M62 - M81 0.04385 0.00230 0.04228 0.04127 0.00157

M62 - M82 0.06654 0.00305 0.06256 0.06161 0.00397

M62 - M83 0.08723 0.00181 0.08538 0.08215 0.00186

M62 - M84 0.04829 0.00521 0.04739 0.04117 0.00090

M62 - M85 0.03834 0.00095 0.03694 0.03440 0.00140

M62 - M86 0.08481 0.00142 0.07809 0.07950 0.00672

M62 - M87 0.03646 0.00073 0.03518 0.03370 0.00128

M62 - M88 0.07505 0.00365 0.07359 0.06775 0.00147

M62 - M89 0.09064 0.00100 0.08983 0.09064 0.00081

M62 - M90 0.07635 0.00297 0.07058 0.06917 0.00578

M62 - M91 0.03937 0.00326 0.03786 0.03112 0.00151

M62 - M92 0.06410 0.00180 0.05631 0.06246 0.00779

M62 - M93 0.03743 0.00045 0.03503 0.03558 0.00240

M62 - M94 0.05944 0.00175 0.05870 0.05517 0.00074

M62 - M95 0.05921 0.00212 0.05839 0.05932 0.00083

M62 - M96 0.05894 0.00279 0.05852 0.05341 0.00042

M62 - M97 0.06892 0.00369 0.06762 0.06543 0.00130

M63 - M64 0.04728 0.00165 0.04679 0.04450 0.00049

M63 - M65 0.08245 0.00181 0.07094 0.07655 0.01151

M63 - M66 0.07178 0.00371 0.07111 0.07058 0.00067

M63 - M67 0.07765 0.00219 0.07699 0.07691 0.00066

M63 - M68 0.02958 0.00168 0.02736 0.02796 0.00221

M63 - M69 0.05037 0.00406 0.04504 0.04567 0.00533

M63 - M70 0.06302 0.00448 0.05554 0.05929 0.00748

M63 - M71 0.04719 0.00168 0.04331 0.04679 0.00388

M63 - M72 0.02535 0.00143 0.02416 0.02387 0.00119

M63 - M73 0.03026 0.00025 0.02984 0.02915 0.00043

M63 - M74 0.03382 0.00101 0.03341 0.02917 0.00041

M63 - M75 0.02553 0.00055 0.02421 0.02403 0.00132

M63 - M76 0.07525 0.00044 0.07463 0.07763 0.00063

M63 - M77 0.03637 0.00087 0.03351 0.03547 0.00286

M63 - M78 0.02489 0.00039 0.02403 0.02600 0.00086

M63 - M79 0.04663 0.00104 0.04624 0.04727 0.00039

M63 - M80 0.03556 0.00244 0.03522 0.03207 0.00034

M63 - M81 0.04375 0.00236 0.04213 0.04058 0.00162

M63 - M82 0.06762 0.00336 0.06323 0.06141 0.00439

M63 - M83 0.08784 0.00204 0.08599 0.08175 0.00186

M63 - M84 0.04734 0.00496 0.04638 0.04035 0.00096

M63 - M85 0.03648 0.00073 0.03494 0.03354 0.00154

M63 - M86 0.08314 0.00120 0.07607 0.07886 0.00707

M63 - M87 0.03580 0.00073 0.03455 0.03296 0.00125

M63 - M88 0.07571 0.00315 0.07419 0.06949 0.00153

M63 - M89 0.09109 0.00122 0.09015 0.09112 0.00093

M63 - M90 0.07479 0.00265 0.06902 0.06858 0.00578

M63 - M91 0.03836 0.00316 0.03672 0.03031 0.00164

M63 - M92 0.06288 0.00155 0.05544 0.06182 0.00744

M63 - M93 0.03609 0.00038 0.03369 0.03442 0.00240

M63 - M94 0.05719 0.00139 0.05643 0.05399 0.00075

M63 - M95 0.05794 0.00195 0.05706 0.05861 0.00088

M63 - M96 0.05791 0.00257 0.05748 0.05274 0.00044

M63 - M97 0.06808 0.00335 0.06696 0.06492 0.00111

M64 - M65 0.06569 0.00637 0.05789 0.06512 0.00780

M64 - M66 0.07306 0.01245 0.07262 0.06395 0.00044

M64 - M67 0.06599 0.01041 0.06530 0.06070 0.00069

M64 - M68 0.05937 0.02415 0.05724 0.03223 0.00213

M64 - M69 0.05488 0.00577 0.05356 0.04767 0.00132

M64 - M70 0.05380 0.00462 0.04970 0.05036 0.00410

M64 - M71 0.07712 0.02386 0.07572 0.04742 0.00140

M64 - M72 0.08117 0.03571 0.06266 0.03294 0.01851

M64 - M73 0.03879 0.00400 0.03842 0.04192 0.00037

M64 - M74 0.04528 0.00151 0.04474 0.04274 0.00054

M64 - M75 0.04817 0.01436 0.04701 0.03643 0.00116

M64 - M76 0.07802 0.00709 0.07775 0.07507 0.00027

M64 - M77 0.04934 0.02169 0.04724 0.03494 0.00209

M64 - M78 0.04434 0.00193 0.04371 0.04441 0.00064

M64 - M79 0.04537 0.00859 0.04457 0.04024 0.00080

M64 - M80 0.05159 0.02367 0.04931 0.02975 0.00228

M64 - M81 0.05279 0.00901 0.05149 0.04685 0.00130

M64 - M82 0.06822 0.00988 0.06428 0.05606 0.00394

M64 - M83 0.08237 0.01108 0.07940 0.07315 0.00297

M64 - M84 0.05171 0.00937 0.05102 0.04005 0.00069

M64 - M85 0.04656 0.00813 0.04490 0.04720 0.00166

M64 - M86 0.08343 0.01910 0.07722 0.07123 0.00621

M64 - M87 0.04547 0.00811 0.04361 0.04612 0.00186

M64 - M88 0.08468 0.02170 0.07760 0.06252 0.00709

M64 - M89 0.11050 0.01905 0.10985 0.08595 0.00065

M64 - M90 0.07248 0.00937 0.06952 0.05765 0.00296

M64 - M91 0.05227 0.01307 0.05122 0.03891 0.00105

M64 - M92 0.07349 0.02304 0.06676 0.05570 0.00673

M64 - M93 0.04686 0.00191 0.04616 0.04544 0.00070

M64 - M94 0.06285 0.00971 0.06075 0.05290 0.00210

M64 - M95 0.06881 0.01869 0.06764 0.04983 0.00117

M64 - M96 0.07059 0.02200 0.06652 0.04945 0.00407

M64 - M97 0.08092 0.02100 0.07820 0.05878 0.00272

M65 - M66 0.10393 0.01137 0.09931 0.08830 0.00462

M65 - M67 0.11311 0.01542 0.10567 0.09634 0.00745

M65 - M68 0.05372 0.00646 0.04653 0.04895 0.00719

M65 - M69 0.08175 0.01031 0.05891 0.07250 0.02284

M65 - M70 0.08701 0.00681 0.07449 0.08133 0.01252

M65 - M71 0.06869 0.00279 0.06308 0.06196 0.00562

M65 - M72 0.05275 0.00783 0.04495 0.04624 0.00780

M65 - M73 0.06878 0.00024 0.06137 0.06714 0.00741

M65 - M74 0.07184 0.00098 0.06313 0.07042 0.00871

M65 - M75 0.05579 0.00074 0.04996 0.05673 0.00583

M65 - M76 0.10040 0.00417 0.09562 0.09675 0.00478

M65 - M77 0.05774 0.00117 0.05248 0.05681 0.00525

M65 - M78 0.06420 0.00172 0.05658 0.06305 0.00762

M65 - M79 0.07616 0.00202 0.06977 0.07618 0.00639

M65 - M80 0.05509 0.00344 0.05034 0.05258 0.00475

M65 - M81 0.06933 0.00412 0.06294 0.06289 0.00639

M65 - M82 0.08149 0.00248 0.07263 0.07687 0.00885

M65 - M83 0.09246 0.00288 0.08692 0.08997 0.00554

M65 - M84 0.06445 0.00278 0.05911 0.05817 0.00534

M65 - M85 0.06809 0.00346 0.06126 0.06547 0.00683

M65 - M86 0.10207 0.00286 0.09387 0.09829 0.00820

M65 - M87 0.06677 0.00253 0.06070 0.06471 0.00607

M65 - M88 0.09449 0.00491 0.08781 0.08513 0.00668

M65 - M89 0.11270 0.00429 0.10824 0.10107 0.00447

M65 - M90 0.09193 0.00191 0.08030 0.09046 0.01163

M65 - M91 0.06564 0.00228 0.05310 0.06577 0.01254

M65 - M92 0.07628 0.00262 0.06835 0.07836 0.00794

M65 - M93 0.07550 0.00214 0.06382 0.07025 0.01167

M65 - M94 0.08629 0.00240 0.07967 0.08102 0.00662

M65 - M95 0.08850 0.00695 0.08412 0.07851 0.00438

M65 - M96 0.07688 0.00591 0.07252 0.06978 0.00436

M65 - M97 0.09266 0.00736 0.08705 0.07875 0.00560

M66 - M67 0.13657 0.04323 0.08431 0.09118 0.05226

M66 - M68 0.05606 0.00539 0.05304 0.05177 0.00302

M66 - M69 0.08240 0.01284 0.08135 0.07215 0.00105

M66 - M70 0.08768 0.00667 0.08406 0.08192 0.00362

M66 - M71 0.09411 0.02349 0.08335 0.06440 0.01075

M66 - M72 0.07449 0.01492 0.06529 0.05206 0.00920

M66 - M73 0.07204 0.00153 0.07183 0.06486 0.00021

M66 - M74 0.07670 0.00154 0.07661 0.07142 0.00008

M66 - M75 0.06078 0.00420 0.06017 0.05885 0.00061

M66 - M76 0.09642 0.00372 0.09617 0.09721 0.00025

M66 - M77 0.06874 0.00465 0.06685 0.06273 0.00189

M66 - M78 0.06695 0.00393 0.06489 0.06995 0.00206

M66 - M79 0.08396 0.00498 0.08313 0.08027 0.00082

M66 - M80 0.05643 0.00522 0.05635 0.05160 0.00008

M66 - M81 0.07403 0.00603 0.07259 0.06735 0.00144

M66 - M82 0.10181 0.02662 0.09744 0.07426 0.00437

M66 - M83 0.10135 0.00693 0.10015 0.08919 0.00120

M66 - M84 0.07530 0.01894 0.07404 0.05629 0.00126

M66 - M85 0.07372 0.00609 0.07292 0.06940 0.00080

M66 - M86 0.10594 0.00361 0.10111 0.10292 0.00483

M66 - M87 0.07709 0.00583 0.07602 0.07098 0.00107

M66 - M88 0.09092 0.00628 0.08961 0.08410 0.00131

M66 - M89 0.10713 0.00714 0.10697 0.09733 0.00016

M66 - M90 0.09115 0.00451 0.08491 0.08542 0.00624

M66 - M91 0.06252 0.00541 0.06082 0.06405 0.00170

M66 - M92 0.08060 0.01194 0.07690 0.07466 0.00370

M66 - M93 0.08316 0.00480 0.08243 0.07335 0.00072

M66 - M94 0.09442 0.00761 0.09329 0.08352 0.00113

M66 - M95 0.07499 0.00563 0.07419 0.07044 0.00079

M66 - M96 0.07713 0.01329 0.07681 0.06665 0.00031

M66 - M97 0.08130 0.00745 0.07966 0.07211 0.00164

M67 - M68 0.06230 0.01122 0.06131 0.05208 0.00098

M67 - M69 0.08405 0.00860 0.08197 0.07454 0.00209

M67 - M70 0.10272 0.01557 0.09428 0.08308 0.00844

M67 - M71 0.07026 0.00830 0.06453 0.06212 0.00573

M67 - M72 0.07691 0.01829 0.06899 0.05139 0.00792

M67 - M73 0.06383 0.00054 0.06238 0.06450 0.00145

M67 - M74 0.07971 0.00204 0.07932 0.07714 0.00039

M67 - M75 0.06194 0.00458 0.06014 0.06163 0.00181

M67 - M76 0.09164 0.00496 0.09114 0.08935 0.00050

M67 - M77 0.06022 0.00270 0.05918 0.05691 0.00104

M67 - M78 0.06987 0.00157 0.06931 0.07019 0.00056

M67 - M79 0.08905 0.00728 0.08796 0.08456 0.00108

M67 - M80 0.05323 0.00345 0.05297 0.05012 0.00026

M67 - M81 0.08345 0.00887 0.08085 0.06359 0.00260

M67 - M82 0.08412 0.01197 0.08137 0.07161 0.00275

M67 - M83 0.09544 0.01001 0.09405 0.08264 0.00139

M67 - M84 0.06122 0.00658 0.06039 0.05471 0.00083

M67 - M85 0.06626 0.00169 0.06451 0.06631 0.00175

M67 - M86 0.09256 0.00142 0.08713 0.09397 0.00543

M67 - M87 0.06633 0.00176 0.06506 0.06647 0.00128

M67 - M88 0.08206 0.00522 0.08078 0.07976 0.00128

M67 - M89 0.09148 0.00272 0.09104 0.08876 0.00044

M67 - M90 0.09270 0.00774 0.08796 0.08678 0.00473

M67 - M91 0.06911 0.00419 0.06810 0.06891 0.00100

M67 - M92 0.08954 0.01000 0.08446 0.07593 0.00508

M67 - M93 0.07521 0.00265 0.07434 0.07375 0.00087

M67 - M94 0.09732 0.00590 0.09640 0.09214 0.00092

M67 - M95 0.08742 0.00912 0.08630 0.07177 0.00111

M67 - M96 0.08160 0.01220 0.08044 0.06721 0.00115

M67 - M97 0.07382 0.00324 0.07048 0.07034 0.00335

M68 - M69 0.04203 0.00906 0.03993 0.03596 0.00210

M68 - M70 0.05710 0.01458 0.05318 0.04076 0.00393

M68 - M71 0.06184 0.02204 0.05752 0.03621 0.00432

M68 - M72 0.05880 0.02477 0.05251 0.02473 0.00628

M68 - M73 0.02762 0.00406 0.02692 0.02913 0.00069

M68 - M74 0.03046 0.00133 0.02876 0.02945 0.00171

M68 - M75 0.03264 0.00835 0.03154 0.02674 0.00110

M68 - M76 0.06467 0.00512 0.06372 0.06557 0.00095

M68 - M77 0.04443 0.02307 0.03989 0.02720 0.00455

M68 - M78 0.03712 0.00498 0.03414 0.03076 0.00298

M68 - M79 0.04029 0.00558 0.03986 0.03140 0.00042

M68 - M80 0.04438 0.02404 0.04255 0.02164 0.00184

M68 - M81 0.03598 0.00880 0.03443 0.03313 0.00154

M68 - M82 0.05198 0.00792 0.04883 0.04738 0.00316

M68 - M83 0.06739 0.01242 0.06549 0.05720 0.00189

M68 - M84 0.03860 0.00917 0.03750 0.02938 0.00110

M68 - M85 0.03586 0.00640 0.03488 0.03247 0.00098

M68 - M86 0.07512 0.01875 0.07051 0.05684 0.00461

M68 - M87 0.03508 0.00614 0.03418 0.03203 0.00090

M68 - M88 0.07888 0.01960 0.07719 0.05704 0.00169

M68 - M89 0.09382 0.01973 0.09096 0.06690 0.00287

M68 - M90 0.05222 0.00441 0.04910 0.04803 0.00312

M68 - M91 0.03350 0.00678 0.03231 0.02721 0.00118

M68 - M92 0.07124 0.02254 0.06764 0.04674 0.00360

M68 - M93 0.03601 0.00226 0.03530 0.03198 0.00071

M68 - M94 0.04642 0.00756 0.04520 0.04009 0.00122

M68 - M95 0.05933 0.01976 0.05699 0.03932 0.00234

M68 - M96 0.06772 0.02669 0.06617 0.03863 0.00155

M68 - M97 0.06701 0.01944 0.06486 0.04515 0.00214

M69 - M70 0.08082 0.01548 0.06116 0.06329 0.01966

M69 - M71 0.05647 0.00996 0.05193 0.04743 0.00453

M69 - M72 0.04284 0.01083 0.04068 0.03204 0.00216

M69 - M73 0.04039 0.00023 0.03930 0.03984 0.00109

M69 - M74 0.04442 0.00140 0.04308 0.04289 0.00134

M69 - M75 0.03896 0.00400 0.03669 0.03524 0.00227

M69 - M76 0.10357 0.01013 0.10101 0.08026 0.00256

M69 - M77 0.04168 0.00374 0.03937 0.04336 0.00231

M69 - M78 0.04532 0.00118 0.04325 0.04421 0.00207

M69 - M79 0.05636 0.00347 0.05539 0.05476 0.00097

M69 - M80 0.03968 0.00356 0.03880 0.03716 0.00088

M69 - M81 0.05111 0.00323 0.04843 0.04500 0.00268

M69 - M82 0.07622 0.00878 0.07233 0.06179 0.00389

M69 - M83 0.07758 0.00276 0.07510 0.07304 0.00248

M69 - M84 0.05287 0.00888 0.05137 0.04329 0.00150

M69 - M85 0.04284 0.00262 0.04105 0.04119 0.00179

M69 - M86 0.07729 0.00227 0.07214 0.07644 0.00515

M69 - M87 0.03931 0.00257 0.03777 0.03880 0.00155

M69 - M88 0.08291 0.00998 0.07986 0.07071 0.00304

M69 - M89 0.09061 0.01017 0.08608 0.08341 0.00453

M69 - M90 0.07891 0.00418 0.07218 0.07586 0.00673

M69 - M91 0.04733 0.00607 0.04196 0.04391 0.00537

M69 - M92 0.06470 0.00554 0.06030 0.06247 0.00440

M69 - M93 0.04909 0.00212 0.04694 0.04810 0.00215

M69 - M94 0.07052 0.00572 0.06908 0.06168 0.00144

M69 - M95 0.05831 0.00285 0.05643 0.05286 0.00188

M69 - M96 0.04956 0.00299 0.04642 0.04812 0.00314

M69 - M97 0.06467 0.00796 0.06007 0.06188 0.00461

M70 - M71 0.06652 0.01372 0.04828 0.05278 0.01824

M70 - M72 0.04987 0.00776 0.04512 0.03995 0.00475

M70 - M73 0.04813 0.00062 0.04399 0.04722 0.00413

M70 - M74 0.05936 0.00105 0.05363 0.05893 0.00573

M70 - M75 0.04631 0.00416 0.04085 0.04344 0.00546

M70 - M76 0.08211 0.00334 0.07768 0.07826 0.00444

M70 - M77 0.04732 0.00150 0.04283 0.04612 0.00448

M70 - M78 0.05944 0.00095 0.05352 0.05883 0.00592

M70 - M79 0.07214 0.00375 0.06711 0.06894 0.00502

M70 - M80 0.04649 0.00540 0.04256 0.04044 0.00393

M70 - M81 0.06031 0.00918 0.05511 0.05004 0.00519

M70 - M82 0.06929 0.00445 0.06285 0.06215 0.00645

M70 - M83 0.08033 0.00447 0.07546 0.07409 0.00487

M70 - M84 0.04895 0.00245 0.04426 0.04544 0.00469

M70 - M85 0.05219 0.00304 0.04731 0.04882 0.00488

M70 - M86 0.08377 0.00469 0.07627 0.07801 0.00750

M70 - M87 0.05180 0.00258 0.04571 0.04949 0.00610

M70 - M88 0.07930 0.00797 0.07446 0.07056 0.00484

M70 - M89 0.09163 0.00651 0.08374 0.08646 0.00789

M70 - M90 0.08650 0.00645 0.07473 0.07998 0.01177

M70 - M91 0.06749 0.00547 0.05705 0.05883 0.01044

M70 - M92 0.07484 0.00760 0.06643 0.06771 0.00841

M70 - M93 0.05991 0.00094 0.04999 0.06311 0.00992

M70 - M94 0.07967 0.00701 0.07357 0.06802 0.00610

M70 - M95 0.06774 0.01097 0.06325 0.05865 0.00450

M70 - M96 0.06429 0.00583 0.05994 0.05596 0.00435

M70 - M97 0.06685 0.00742 0.06163 0.06094 0.00522

M71 - M72 0.06529 0.02030 0.06369 0.03855 0.00160

M71 - M73 0.05106 0.00410 0.04998 0.04805 0.00108

M71 - M74 0.04267 0.00131 0.04152 0.04485 0.00115

M71 - M75 0.04836 0.00739 0.04610 0.04316 0.00226

M71 - M76 0.07992 0.00512 0.07824 0.07968 0.00168

M71 - M77 0.06984 0.02429 0.06817 0.04581 0.00167

M71 - M78 0.04758 0.00111 0.04566 0.04478 0.00192

M71 - M79 0.05304 0.00405 0.05249 0.04672 0.00056

M71 - M80 0.06125 0.02485 0.05882 0.03598 0.00242

M71 - M81 0.05174 0.00523 0.04916 0.05010 0.00258

M71 - M82 0.06829 0.01268 0.06458 0.05861 0.00371

M71 - M83 0.07780 0.00852 0.07570 0.07257 0.00209

M71 - M84 0.05250 0.01101 0.05059 0.04271 0.00192

M71 - M85 0.05632 0.00760 0.05466 0.05142 0.00167

M71 - M86 0.09668 0.01731 0.09161 0.07834 0.00507

M71 - M87 0.05996 0.00762 0.05836 0.05378 0.00160

M71 - M88 0.09021 0.02177 0.08707 0.06555 0.00314

M71 - M89 0.10645 0.01592 0.10068 0.08456 0.00577

M71 - M90 0.06799 0.00759 0.06278 0.05834 0.00521

M71 - M91 0.04673 0.00538 0.04370 0.04070 0.00304

M71 - M92 0.08521 0.02153 0.07595 0.05802 0.00926

M71 - M93 0.05564 0.00191 0.05240 0.05067 0.00324

M71 - M94 0.05830 0.00410 0.05641 0.05341 0.00189

M71 - M95 0.07540 0.01832 0.07429 0.05235 0.00112

M71 - M96 0.08080 0.02170 0.07930 0.05127 0.00150

M71 - M97 0.08608 0.02095 0.08082 0.05767 0.00526

M72 - M73 0.04262 0.00441 0.04020 0.03228 0.00242

M72 - M74 0.03178 0.00503 0.03148 0.02850 0.00030

M72 - M75 0.04712 0.00918 0.04107 0.02679 0.00605

M72 - M76 0.08297 0.00924 0.08165 0.06783 0.00131

M72 - M77 0.07744 0.02581 0.07186 0.04080 0.00557

M72 - M78 0.02477 0.00206 0.02424 0.02704 0.00053

M72 - M79 0.05228 0.00464 0.04987 0.04452 0.00241

M72 - M80 0.05616 0.02552 0.05305 0.02852 0.00311

M72 - M81 0.05198 0.01215 0.04761 0.03532 0.00438

M72 - M82 0.06156 0.01722 0.05882 0.04615 0.00274

M72 - M83 0.06897 0.01316 0.06766 0.05721 0.00131

M72 - M84 0.03936 0.01371 0.03844 0.02743 0.00093

M72 - M85 0.04766 0.00835 0.04524 0.03380 0.00243

M72 - M86 0.09720 0.01784 0.08815 0.06252 0.00906

M72 - M87 0.04973 0.00854 0.04761 0.03531 0.00212

M72 - M88 0.08825 0.02212 0.08323 0.05807 0.00503

M72 - M89 0.08803 0.01860 0.08756 0.07153 0.00047

M72 - M90 0.05412 0.00695 0.05138 0.04611 0.00274

M72 - M91 0.03347 0.00927 0.03212 0.02319 0.00134

M72 - M92 0.09263 0.02460 0.07927 0.05166 0.01336

M72 - M93 0.03038 0.00204 0.02942 0.03101 0.00096

M72 - M94 0.04548 0.00752 0.04338 0.03804 0.00211

M72 - M95 0.06671 0.01993 0.06616 0.04063 0.00055

M72 - M96 0.07665 0.03090 0.07434 0.03892 0.00231

M72 - M97 0.07446 0.01999 0.07178 0.04494 0.00268

M73 - M74 0.02858 0.00472 0.02819 0.03355 0.00039

M73 - M75 0.02684 0.01560 0.02565 0.03316 0.00119

M73 - M76 0.09576 0.01826 0.08850 0.10093 0.00726

M73 - M77 0.05386 0.01947 0.05216 0.05443 0.00170

M73 - M78 0.02980 0.00042 0.02936 0.03227 0.00044

M73 - M79 0.06417 0.00014 0.06331 0.06467 0.00086

M73 - M80 0.05860 0.02001 0.05481 0.04645 0.00380

M73 - M81 0.05014 0.01577 0.04875 0.04818 0.00138

M73 - M82 0.09587 0.01600 0.08759 0.07223 0.00828

M73 - M83 0.10561 0.01579 0.10326 0.08338 0.00235

M73 - M84 0.06009 0.01567 0.05534 0.03984 0.00475

M73 - M85 0.03505 0.01538 0.03401 0.04221 0.00104

M73 - M86 0.06431 0.00382 0.06006 0.07541 0.00424

M73 - M87 0.03360 0.01535 0.03258 0.04068 0.00102

M73 - M88 0.08034 0.00545 0.07902 0.08172 0.00133

M73 - M89 0.11189 0.00491 0.11144 0.10199 0.00045

M73 - M90 0.06113 0.00076 0.05788 0.05955 0.00325

M73 - M91 0.02358 0.00083 0.02232 0.02373 0.00126

M73 - M92 0.05557 0.00397 0.05167 0.06093 0.00390

M73 - M93 0.03087 0.00002 0.03029 0.03104 0.00058

M73 - M94 0.04999 0.00146 0.04882 0.04896 0.00116

M73 - M95 0.05424 0.00392 0.05362 0.05698 0.00061

M73 - M96 0.05422 0.00391 0.05383 0.05332 0.00038

M73 - M97 0.06146 0.00446 0.06066 0.06185 0.00080

M74 - M75 0.03278 0.00090 0.03198 0.02997 0.00079

M74 - M76 0.07931 0.00081 0.07862 0.08519 0.00069

M74 - M77 0.04451 0.00151 0.04162 0.04102 0.00289

M74 - M78 0.02954 0.00039 0.02924 0.03125 0.00030

M74 - M79 0.06204 0.00267 0.06193 0.05234 0.00010

M74 - M80 0.02960 0.00127 0.02952 0.03076 0.00008

M74 - M81 0.05172 0.00256 0.04815 0.04499 0.00357

M74 - M82 0.06311 0.00231 0.05773 0.06390 0.00538

M74 - M83 0.07166 0.00201 0.07067 0.07509 0.00099

M74 - M84 0.03304 0.00255 0.03078 0.03473 0.00226

M74 - M85 0.04137 0.00144 0.04046 0.03883 0.00092

M74 - M86 0.08205 0.00072 0.07612 0.08407 0.00593

M74 - M87 0.03999 0.00151 0.03904 0.03724 0.00096

M74 - M88 0.07363 0.00304 0.07256 0.07467 0.00107

M74 - M89 0.09197 0.00045 0.09129 0.09229 0.00068

M74 - M90 0.06490 0.00174 0.06215 0.06646 0.00275

M74 - M91 0.03530 0.00264 0.03359 0.02834 0.00171

M74 - M92 0.06740 0.00210 0.06187 0.06373 0.00553

M74 - M93 0.03056 0.00033 0.02997 0.02912 0.00059

M74 - M94 0.05176 0.00253 0.04843 0.05411 0.00332

M74 - M95 0.05821 0.00139 0.05816 0.05585 0.00005

M74 - M96 0.05303 0.00191 0.05299 0.05116 0.00004

M74 - M97 0.05440 0.00067 0.05375 0.05522 0.00065

M75 - M76 0.08362 0.01939 0.07866 0.08495 0.00496

M75 - M77 0.05139 0.02143 0.04891 0.04787 0.00249

M75 - M78 0.02672 0.00014 0.02483 0.02786 0.00189

M75 - M79 0.06113 0.00165 0.05853 0.06031 0.00260

M75 - M80 0.05493 0.02149 0.05174 0.03930 0.00319

M75 - M81 0.05500 0.01768 0.05308 0.04205 0.00193

M75 - M82 0.08518 0.01784 0.07399 0.06073 0.01119

M75 - M83 0.09662 0.01599 0.09299 0.07193 0.00363

M75 - M84 0.05131 0.01627 0.04590 0.03184 0.00542

M75 - M85 0.02870 0.01973 0.02690 0.03757 0.00180

M75 - M86 0.05727 0.00759 0.05294 0.07175 0.00434

M75 - M87 0.02759 0.01969 0.02593 0.03608 0.00165

M75 - M88 0.07180 0.00763 0.07029 0.06986 0.00151

M75 - M89 0.09341 0.00690 0.09260 0.08847 0.00081

M75 - M90 0.06041 0.00443 0.05641 0.05064 0.00400

M75 - M91 0.02579 0.01015 0.02410 0.02034 0.00169

M75 - M92 0.05154 0.01052 0.04749 0.05957 0.00405

M75 - M93 0.02442 0.00052 0.02345 0.02415 0.00097

M75 - M94 0.06027 0.00660 0.05900 0.04460 0.00128

M75 - M95 0.05302 0.00591 0.05244 0.05048 0.00058

M75 - M96 0.05508 0.00885 0.05385 0.05017 0.00122

M75 - M97 0.05065 0.00528 0.04972 0.05250 0.00093

M76 - M77 0.10806 0.02176 0.10532 0.10254 0.00275

M76 - M78 0.09407 0.00117 0.09363 0.08863 0.00044

M76 - M79 0.09751 0.00332 0.09716 0.09965 0.00035

M76 - M80 0.09549 0.02134 0.08808 0.08535 0.00741

M76 - M81 0.10700 0.01873 0.10549 0.08738 0.00151

M76 - M82 0.13353 0.02293 0.12207 0.10455 0.01146

M76 - M83 0.12771 0.01623 0.12122 0.11073 0.00649

M76 - M84 0.10069 0.02351 0.09144 0.07208 0.00926

M76 - M85 0.09642 0.01552 0.09461 0.09389 0.00181

M76 - M86 0.11692 0.00394 0.11280 0.12264 0.00412

M76 - M87 0.09315 0.01600 0.09070 0.09366 0.00244

M76 - M88 0.12617 0.02615 0.12062 0.11340 0.00555

M76 - M89 0.12878 0.00741 0.12779 0.12417 0.00098

M76 - M90 0.09492 0.00399 0.09040 0.09248 0.00452

M76 - M91 0.06282 0.00119 0.06163 0.06531 0.00119

M76 - M92 0.10541 0.00642 0.10090 0.09891 0.00451

M76 - M93 0.08204 0.00163 0.07969 0.08408 0.00234

M76 - M94 0.08305 0.00168 0.08232 0.08323 0.00074

M76 - M95 0.09328 0.00903 0.09305 0.08626 0.00023

M76 - M96 0.08702 0.00815 0.08644 0.08072 0.00058

M76 - M97 0.10227 0.00764 0.10165 0.09786 0.00062

M77 - M78 0.05082 0.00391 0.04587 0.03997 0.00495

M77 - M79 0.05077 0.00365 0.04976 0.05419 0.00101

M77 - M80 0.11384 0.07768 0.03938 0.03960 0.07446

M77 - M81 0.05695 0.02060 0.05350 0.05178 0.00345

M77 - M82 0.08998 0.02393 0.08446 0.06213 0.00551

M77 - M83 0.09450 0.02183 0.08959 0.06942 0.00491

M77 - M84 0.06190 0.02187 0.05901 0.03975 0.00289

M77 - M85 0.05394 0.02100 0.05141 0.05377 0.00254

M77 - M86 0.05619 0.02087 0.05281 0.05820 0.00337

M77 - M87 0.05385 0.02107 0.05142 0.05368 0.00243

M77 - M88 0.08108 0.02383 0.07943 0.06155 0.00165

M77 - M89 0.11438 0.02151 0.10813 0.08826 0.00625

M77 - M90 0.07109 0.00591 0.06780 0.05890 0.00330

M77 - M91 0.03798 0.00343 0.03527 0.03159 0.00272

M77 - M92 0.07075 0.02461 0.06756 0.05291 0.00319

M77 - M93 0.04758 0.00170 0.04447 0.04310 0.00311

M77 - M94 0.05776 0.00739 0.05628 0.04989 0.00148

M77 - M95 0.06530 0.02228 0.06317 0.04444 0.00214

M77 - M96 0.06311 0.02421 0.05914 0.04535 0.00397

M77 - M97 0.06331 0.02549 0.05992 0.05199 0.00339

M78 - M79 0.04727 0.00225 0.04681 0.04885 0.00046

M78 - M80 0.03189 0.00116 0.03131 0.03109 0.00057

M78 - M81 0.05003 0.00121 0.04448 0.04576 0.00555

M78 - M82 0.06682 0.00446 0.06246 0.06726 0.00436

M78 - M83 0.07491 0.00117 0.07373 0.07771 0.00118

M78 - M84 0.04136 0.00646 0.03921 0.03578 0.00216

M78 - M85 0.03728 0.00044 0.03532 0.03803 0.00196

M78 - M86 0.08723 0.00020 0.07805 0.08454 0.00917

M78 - M87 0.03688 0.00008 0.03448 0.03608 0.00240

M78 - M88 0.08077 0.00075 0.07793 0.08057 0.00283

M78 - M89 0.08827 0.00060 0.08789 0.09234 0.00039

M78 - M90 0.06931 0.00267 0.06414 0.06836 0.00517

M78 - M91 0.02871 0.00147 0.02692 0.02786 0.00179

M78 - M92 0.07217 0.00155 0.06380 0.06588 0.00837

M78 - M93 0.02957 0.00071 0.02879 0.03007 0.00078

M78 - M94 0.05810 0.00270 0.05703 0.05097 0.00107

M78 - M95 0.04928 0.00141 0.04867 0.04973 0.00061

M78 - M96 0.04701 0.00130 0.04675 0.04557 0.00026

M78 - M97 0.06261 0.00127 0.06172 0.05791 0.00089

M79 - M80 0.03907 0.00320 0.03887 0.03990 0.00020

M79 - M81 0.07594 0.00533 0.07074 0.06419 0.00520

M79 - M82 0.06712 0.00536 0.06543 0.06247 0.00170

M79 - M83 0.07087 0.00286 0.06997 0.06914 0.00089

M79 - M84 0.04255 0.00435 0.04214 0.03724 0.00042

M79 - M85 0.06538 0.00251 0.06313 0.06843 0.00225

M79 - M86 0.07042 0.00173 0.06678 0.07356 0.00364

M79 - M87 0.06341 0.00239 0.06124 0.06789 0.00216

M79 - M88 0.07376 0.00457 0.07317 0.06735 0.00059

M79 - M89 0.09788 0.00311 0.09626 0.09280 0.00162

M79 - M90 0.07670 0.00521 0.07126 0.07259 0.00544

M79 - M91 0.04121 0.00314 0.03962 0.04273 0.00159

M79 - M92 0.05744 0.00089 0.05494 0.05536 0.00250

M79 - M93 0.04696 0.00083 0.04631 0.04754 0.00065

M79 - M94 0.07011 0.00376 0.06915 0.06264 0.00096

M79 - M95 0.06229 0.00552 0.06055 0.05140 0.00174

M79 - M96 0.05349 0.00293 0.05308 0.05219 0.00041

M79 - M97 0.04986 0.00294 0.04950 0.04863 0.00035

M80 - M81 0.05834 0.02060 0.05502 0.04207 0.00332

M80 - M82 0.08199 0.02617 0.07764 0.05148 0.00435

M80 - M83 0.07831 0.02275 0.07504 0.05789 0.00326

M80 - M84 0.05800 0.02610 0.05691 0.03300 0.00109

M80 - M85 0.05204 0.02146 0.04883 0.04177 0.00320

M80 - M86 0.05592 0.02053 0.05281 0.04512 0.00311

M80 - M87 0.05405 0.02121 0.05182 0.04149 0.00223

M80 - M88 0.07332 0.02601 0.06653 0.05070 0.00679

M80 - M89 0.09595 0.02091 0.09231 0.07440 0.00364

M80 - M90 0.05965 0.00436 0.05752 0.05212 0.00213

M80 - M91 0.03125 0.00338 0.03034 0.02541 0.00090

M80 - M92 0.06565 0.02627 0.06131 0.04142 0.00434

M80 - M93 0.03614 0.00312 0.03475 0.03422 0.00139

M80 - M94 0.04339 0.00407 0.04280 0.03882 0.00060

M80 - M95 0.06348 0.02399 0.06275 0.03827 0.00073

M80 - M96 0.06048 0.02448 0.05893 0.03761 0.00156

M80 - M97 0.06341 0.02674 0.05878 0.04415 0.00463

M81 - M82 0.09151 0.01958 0.08462 0.06301 0.00689

M81 - M83 0.11106 0.02259 0.10883 0.07699 0.00224

M81 - M84 0.06513 0.02013 0.06016 0.04088 0.00497

M81 - M85 0.06093 0.01746 0.05862 0.05477 0.00230

M81 - M86 0.08514 0.00740 0.07839 0.08667 0.00675

M81 - M87 0.06110 0.01759 0.05888 0.05315 0.00222

M81 - M88 0.07203 0.00803 0.06656 0.06946 0.00547

M81 - M89 0.10066 0.00792 0.09485 0.09180 0.00581

M81 - M90 0.05534 0.00320 0.05195 0.05617 0.00339

M81 - M91 0.04382 0.00441 0.04092 0.03450 0.00290

M81 - M92 0.06682 0.00746 0.06188 0.06773 0.00495

M81 - M93 0.04263 0.00044 0.04023 0.04131 0.00240

M81 - M94 0.05233 0.00306 0.05024 0.05300 0.00209

M81 - M95 0.05984 0.00663 0.05819 0.05524 0.00165

M81 - M96 0.06882 0.00946 0.06514 0.05470 0.00368

M81 - M97 0.06176 0.00474 0.06012 0.06188 0.00163

M82 - M83 0.11338 0.02388 0.10875 0.08190 0.00463

M82 - M84 0.12606 0.06270 0.06164 0.05349 0.06442

M82 - M85 0.09543 0.01708 0.08749 0.06798 0.00794

M82 - M86 0.09785 0.00526 0.08882 0.09189 0.00904

M82 - M87 0.09319 0.01677 0.08506 0.06713 0.00813

M82 - M88 0.09772 0.01000 0.09384 0.08071 0.00388

M82 - M89 0.09486 0.00909 0.09105 0.08833 0.00381

M82 - M90 0.07595 0.00844 0.07094 0.07071 0.00501

M82 - M91 0.05765 0.00740 0.05453 0.05071 0.00313

M82 - M92 0.08199 0.00985 0.07476 0.07259 0.00723

M82 - M93 0.06385 0.00141 0.05912 0.06263 0.00472

M82 - M94 0.07238 0.00508 0.06885 0.06628 0.00353

M82 - M95 0.07723 0.00923 0.07292 0.06776 0.00431

M82 - M96 0.06702 0.00584 0.06446 0.06205 0.00256

M82 - M97 0.07607 0.00772 0.07023 0.07315 0.00584

M83 - M84 0.08953 0.02213 0.08510 0.06489 0.00442

M83 - M85 0.11007 0.01584 0.10739 0.08754 0.00268

M83 - M86 0.11746 0.00528 0.11266 0.11484 0.00480

M83 - M87 0.10834 0.01576 0.10411 0.08810 0.00423

M83 - M88 0.09381 0.00739 0.09173 0.09068 0.00208

M83 - M89 0.11969 0.00631 0.11857 0.11247 0.00111

M83 - M90 0.08714 0.00512 0.07985 0.08094 0.00729

M83 - M91 0.07378 0.00307 0.07003 0.06818 0.00374

M83 - M92 0.08814 0.00609 0.08344 0.08152 0.00471

M83 - M93 0.08863 0.00328 0.08612 0.08588 0.00251

M83 - M94 0.09471 0.00465 0.09300 0.08156 0.00171

M83 - M95 0.08850 0.00961 0.08751 0.07901 0.00099

M83 - M96 0.08563 0.00960 0.08465 0.07590 0.00098

M83 - M97 0.08838 0.00782 0.08676 0.08616 0.00162

M84 - M85 0.06489 0.02037 0.06062 0.03779 0.00426

M84 - M86 0.06878 0.00702 0.06348 0.06297 0.00529

M84 - M87 0.06234 0.02010 0.05724 0.03656 0.00510

M84 - M88 0.06877 0.00996 0.06545 0.05871 0.00332

M84 - M89 0.08247 0.01166 0.07903 0.07330 0.00344

M84 - M90 0.05964 0.00581 0.05440 0.05552 0.00524

M84 - M91 0.03388 0.00215 0.03220 0.03251 0.00168

M84 - M92 0.05717 0.00718 0.05279 0.05159 0.00438

M84 - M93 0.05045 0.00369 0.04826 0.04171 0.00219

M84 - M94 0.04832 0.00322 0.04665 0.04703 0.00168

M84 - M95 0.05160 0.00826 0.04801 0.04766 0.00360

M84 - M96 0.04883 0.00701 0.04792 0.04386 0.00091

M84 - M97 0.06534 0.00825 0.06362 0.05611 0.00172

M85 - M86 0.06419 0.02425 0.05681 0.08523 0.00738

M85 - M87 0.03499 0.03889 0.02634 0.04930 0.00865

M85 - M88 0.09298 0.01719 0.08891 0.08230 0.00407

M85 - M89 0.10433 0.00458 0.10326 0.09979 0.00108

M85 - M90 0.06182 0.00341 0.05769 0.05426 0.00413

M85 - M91 0.02951 0.00479 0.02741 0.02949 0.00210

M85 - M92 0.05850 0.00931 0.05385 0.06503 0.00465

M85 - M93 0.03183 0.00285 0.03056 0.03541 0.00127

M85 - M94 0.07710 0.00935 0.07521 0.05358 0.00189

M85 - M95 0.05566 0.00446 0.05488 0.05605 0.00077

M85 - M96 0.06407 0.00918 0.06337 0.05792 0.00071

M85 - M97 0.06196 0.00613 0.06063 0.06285 0.00133

M86 - M87 0.06097 0.02294 0.05528 0.08374 0.00569

M86 - M88 0.12340 0.02544 0.11600 0.10619 0.00739

M86 - M89 0.15569 0.01625 0.15002 0.13077 0.00566

M86 - M90 0.08686 0.00341 0.08057 0.08178 0.00629

M86 - M91 0.06957 0.00412 0.06490 0.06954 0.00467

M86 - M92 0.08998 0.01958 0.08254 0.08878 0.00744

M86 - M93 0.07660 0.00169 0.07191 0.08086 0.00468

M86 - M94 0.11100 0.00847 0.10362 0.08925 0.00738

M86 - M95 0.09440 0.01681 0.08998 0.07967 0.00442

M86 - M96 0.10450 0.01889 0.09870 0.08426 0.00580

M86 - M97 0.10066 0.01977 0.09496 0.08710 0.00569

M87 - M88 0.09152 0.01663 0.08823 0.08281 0.00329

M87 - M89 0.10889 0.00445 0.10806 0.10473 0.00082

M87 - M90 0.06278 0.00390 0.05858 0.05393 0.00420

M87 - M91 0.02972 0.00476 0.02739 0.02883 0.00233

M87 - M92 0.05685 0.00948 0.05251 0.06537 0.00435

M87 - M93 0.03283 0.00280 0.03164 0.03684 0.00120

M87 - M94 0.07134 0.00901 0.06924 0.05042 0.00210

M87 - M95 0.05518 0.00436 0.05435 0.05631 0.00083

M87 - M96 0.06759 0.00886 0.06649 0.05975 0.00110

M87 - M97 0.06238 0.00631 0.06120 0.06225 0.00118

M88 - M89 0.13277 0.01773 0.13006 0.10455 0.00271

M88 - M90 0.09652 0.00708 0.09335 0.08097 0.00318

M88 - M91 0.07321 0.00619 0.07154 0.06610 0.00166

M88 - M92 0.09753 0.02147 0.09260 0.08238 0.00493

M88 - M93 0.08058 0.00262 0.07909 0.07906 0.00148

M88 - M94 0.08779 0.00827 0.08361 0.07313 0.00418

M88 - M95 0.08598 0.01710 0.08377 0.06848 0.00221

M88 - M96 0.09735 0.02203 0.09620 0.06754 0.00115

M88 - M97 0.10493 0.02075 0.10301 0.07941 0.00192

M89 - M90 0.10186 0.01465 0.09813 0.09008 0.00373

M89 - M91 0.08079 0.00592 0.07519 0.08024 0.00560

M89 - M92 0.12790 0.01944 0.12104 0.10200 0.00686

M89 - M93 0.10227 0.00344 0.10176 0.09643 0.00051

M89 - M94 0.09137 0.00148 0.09081 0.08937 0.00056

M89 - M95 0.11135 0.01953 0.10506 0.08325 0.00628

M89 - M96 0.11205 0.02335 0.11099 0.08208 0.00106

M89 - M97 0.12232 0.01847 0.11984 0.09487 0.00248

M90 - M91 0.08357 0.01136 0.06810 0.06150 0.01546

M90 - M92 0.07415 0.00509 0.06797 0.07175 0.00618

M90 - M93 0.07465 0.00506 0.05584 0.07040 0.01881

M90 - M94 0.08404 0.00575 0.08087 0.07529 0.00317

M90 - M95 0.07280 0.00739 0.06903 0.06571 0.00376

M90 - M96 0.06470 0.00603 0.06183 0.05902 0.00287

M90 - M97 0.08050 0.00906 0.07543 0.07373 0.00508

M91 - M92 0.06161 0.01152 0.05644 0.05376 0.00518

M91 - M93 0.02820 0.00247 0.02405 0.02962 0.00415

M91 - M94 0.06453 0.00720 0.06290 0.04936 0.00163

M91 - M95 0.04877 0.00139 0.04759 0.04700 0.00118

M91 - M96 0.04925 0.00745 0.04828 0.04370 0.00097

M91 - M97 0.05080 0.00252 0.04962 0.05383 0.00117

M92 - M93 0.06538 0.00157 0.05447 0.06267 0.01091

M92 - M94 0.08225 0.00658 0.07832 0.07088 0.00393

M92 - M95 0.08996 0.02076 0.08622 0.06508 0.00374

M92 - M96 0.08519 0.01938 0.08005 0.06137 0.00515

M92 - M97 0.08012 0.01715 0.07441 0.06627 0.00571

M93 - M94 0.06373 0.00526 0.06278 0.05355 0.00095

M93 - M95 0.06126 0.00108 0.06061 0.05749 0.00064

M93 - M96 0.05750 0.00301 0.05611 0.05177 0.00139

M93 - M97 0.07240 0.00170 0.07146 0.06778 0.00094

M94 - M95 0.07443 0.00577 0.07298 0.06896 0.00145

M94 - M96 0.08490 0.00893 0.08177 0.06469 0.00313

M94 - M97 0.07568 0.00588 0.06962 0.06775 0.00605

M95 - M96 0.12844 0.05597 0.07795 0.06028 0.05049

M95 - M97 0.11119 0.03890 0.10238 0.06462 0.00881

M96 - M97 0.10356 0.03567 0.08816 0.05901 0.01540

Overall Average 0.08470 0.00815 0.07947 0.07660 0.00523

=====================================================================================
